# Iodoarene Activation: Take a Leap Forward toward Green and Sustainable Transformations

**DOI:** 10.1021/acs.chemrev.4c00808

**Published:** 2025-03-07

**Authors:** Toshifumi Dohi, Elghareeb E. Elboray, Kotaro Kikushima, Koji Morimoto, Yasuyuki Kita

**Affiliations:** 1 Graduate School of Pharmaceutical Sciences, Ritsumeikan University, 1-1-1, Nojihigashi, Kusatsu Shiga 525-8577, Japan; ‡ Research Organization of Science and Technology, Ritsumeikan University, 1-1-1, Nojihigashi, Kusatsu Shiga 525-8577, Japan; § Department of Chemistry, Faculty of Science, South Valley University, Qena 83523, Egypt

## Abstract

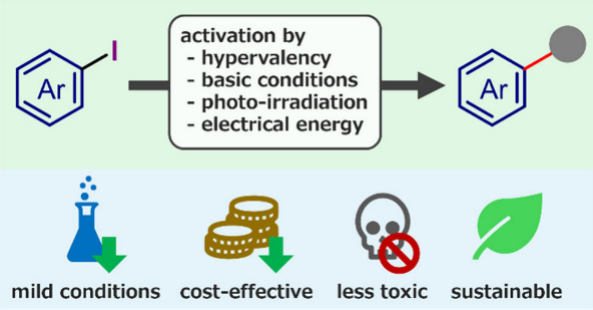

Constructing chemical bonds under green sustainable conditions has drawn attention from environmental and economic perspectives. The dissociation of (hetero)aryl–halide bonds is a crucial step of most arylations affording (hetero)arene derivatives. Herein, we summarize the (hetero)aryl halides activation enabling the direct (hetero)arylation of trapping reagents and construction of highly functionalized (hetero)arenes under benign conditions. The strategies for the activation of aryl iodides are classified into (a) hypervalent iodoarene activation followed by functionalization under thermal/photochemical conditions, (b) aryl–I bond dissociation in the presence of bases with/without organic catalysts and promoters, (c) photoinduced aryl–I bond dissociation in the presence/absence of organophotocatalysts, (d) electrochemical activation of aryl iodides by direct/indirect electrolysis mediated by organocatalysts and mediators acting as electron shuttles, and (e) electrophotochemical activation of aryl iodides mediated by redox-active organocatalysts. These activation modes result in aryl iodides exhibiting diverse reactivity as formal aryl cations/radicals/anions and aryne precursors. The coupling of these reactive intermediates with trapping reagents leads to the facile and selective formation of C–C and C–heteroatom bonds. These ecofriendly, inexpensive, and functional group-tolerant activation strategies offer green alternatives to transition metal-based catalysis.

## 1 Introduction

**Chart 1:**
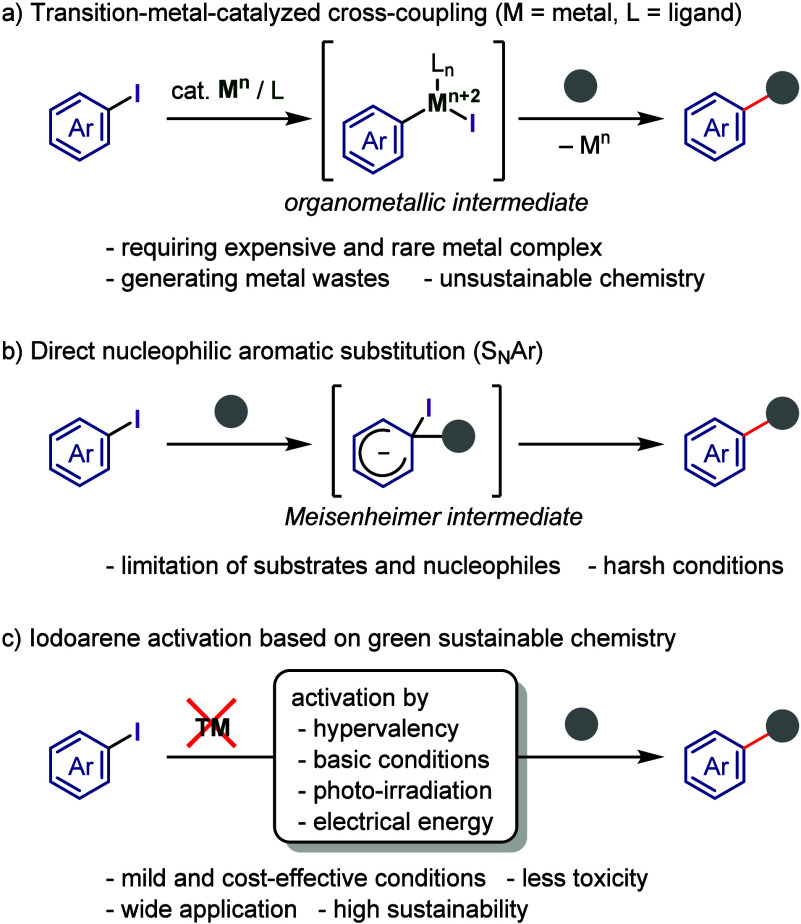
Possible Nucleophile–Organoiodine Couplings

The activation of aryl–halide bonds through transition metal–catalyzed oxidative addition (Chart 1a) has enabled the development of various cross-coupling techniques for constructing C–C, C–O, and C–N bonds.^1−8^ The importance of these transformations, which are crucial for the synthesis of natural products, pharmaceuticals, compounds used in optical devices, and industrially useful starting materials,^9,10^ is reflected by the Nobel Prize awarded to Heck, Negishi, and Suzuki in 2010.^4−6^

Transition metal-catalyzed cross-couplings provide access to a wide range of products but exhibit several drawbacks, including the generation of byproducts and metal-containing waste and the high cost and air/moisture sensitivity of most catalysts. The cost factor is particularly critical when large quantities are required. The generation of byproducts and metal-containing waste also increases environmental and economic costs. Additionally, the isolated products often contain metal impurities, therefore requiring additional purification using specialized equipment and having a limited scope of pharmaceutical and industrial applications.^11,12^ Furthermore, the production of palladium, the primary transition metal used to promote cross-coupling reactions, is strongly influenced by social and environmental factors,^13^ which, together with the scarcity of this metal, considerably affects the cost of the corresponding catalysts. Therefore, novel ecofriendly and sustainable transformation strategies aligning with the advancement of modern synthetic chemistry are urgently required. The nucleophilic aromatic substitution reactions of aryl iodides enable straightforward arene functionalization (Chart 1b) but feature several limitations, such as narrow substrate scope, functional group sensitivity, and harsh conditions.^14,15^

**Chart 2:**
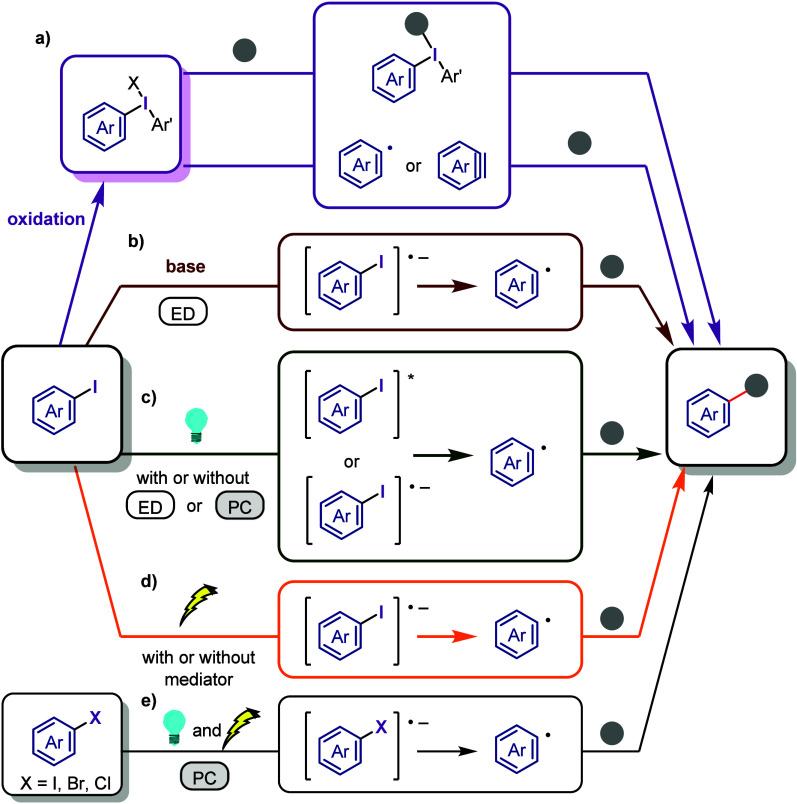
Iodoarene and Haloarene Activation Strategiesa

In this review, we explore the activation methods of organoiodines and discuss their applications in the conversion of aryl–iodide bonds, revealing that these methods enable arylation reactions that may not be achievable under conventional conditions (Chart 1c). We highlight recent pioneering works and outline five key iodoarene activation strategies, as listed below (Chart 2). Although this work mainly focuses on the activation of iodoarenes, the presented concepts can be applied to other haloarenes.

a) Oxidative activation of Ar–I bond (Section 2): Activation to hypervalent organoiodines (e.g., diaryliodonium salts) enables transition metal-free bond formation via ligand coupling, enabling the arylation of diverse nucleophiles and formation of valuable heterocycles. The relatively low oxidation potential of the iodine atom presents a significant advantage for the oxidative activation of iodoarenes in comparison to other halogen atoms, which exhibit greater resistance to oxidation. Diaryliodonium species can act not only as aryl cation synthons but also as aryne and aryl radical equivalents, depending on the applied conditions.
b) Base-promoted Ar–I bond dissociation (Section 3): The activation of aryl iodides by bases with or without organocatalysts is included to shed light on the importance of transition metal-free approaches for C–H arylation, aliphatic carbon activation, addition to unsaturated bonds, hydrodehalogenation, dehalogenative deuteration, hydroxylation, carbonylation, formylation, etc.
c) Photoinduced Ar–I bond dissociation (Section 4): Photochemical iodoarene activation is discussed to address the significance of this green approach in related recent pioneering works. Several strategies have been developed to make this photoactivation chemistry more desirable and accessible even under visible-light irradiation, as exemplified by the direct excitation of haloarenes or facilitation of iodoarene reduction through single electron transfer (SET) from photoexcited catalysts, reaction components, bases, and electron donor–acceptor (EDA) complexes.
d) Electrochemical dissociation of Ar–I bond (Section 5): The electrochemical activation of aryl halides is a promising yet underexplored green route to functionalized arenes.
e) Electrophotoinduced activation of Ar–I bond (Section 6): Electrophotochemical activation enables the dissociation of the difficult-to-activate aryl–halide bonds.

a ED = Electron Donor Species; PC = Photocatalyst.

Based on the above contents, the evaluations of iodoarene activations are discussed in Section 7 from the perspective of green sustainability. These evaluations include synthetic methods for iodoarenes, the design of diaryliodonium salts, and the comparison and assessment of reaction conditions in borylation and hydroxylation.

## 2 Oxidative Activation of Ar–I toward Arylation Reactions

The use of hypervalent iodine chemistry is a highly versatile and ecofriendly strategy enabling a wide range of transformations.^16−20^ This approach exploits readily available nontoxic materials and does not rely on transition metals or ligands, thus presenting a sustainable alternative to traditional methods. The reactivity of hypervalent iodine reagents is based on the strong electrophilicity of the central iodine and high leaving ability of the aryl iodine group, which is ∼10^6^ times greater than that of the triflate group.^21−23^ These properties resemble those of heavy metal-based oxidants and transition metal species, inspiring the development of new synthetic transformations. Hypervalent iodine reagents are therefore used as green alternatives to heavy metals in modern organic synthesis,^24−26^ and their ability to promote the formation of various bonds, such as C–C, C–N, C–X (X = halogen), and C–O, enables the construction of a wide range of organic skeletons.^27−37^ This high oxidative coupling reactivity is also important for the total synthesis of biologically active natural products and their related scaffolds.^38−41^ In addition to featuring excellent oxidant and electrophilic group-transfer abilities, hypervalent iodine reagents can promote different types of rearrangements,^42−44^ such as the Hofmann, Beckmann, [1,2]-migration, [3,3]-sigmatropic/iodonio-Claisen-type, ring contraction, and ring expansion rearrangements, which further enhances their versatility as a synthetic tool.

The importance of hypervalent iodine chemistry as an efficient transition metal-free approach was highlighted after the independent discovery by Kita and Ochiai in the 1980s. Hypervalent iodine reagents are required in stoichiometric or excess amounts, which could have a negative environmental impact and conflict with the principles of green and sustainable chemistry. In 2005, these researchers demonstrated catalytic oxidative transformations facilitated by hypervalent iodine reagents, which were generated in situ through the oxidation of aryl iodides in the presence of sacrificial oxidants.^45,46^ This method was promptly applied to asymmetric synthesis using catalytic chiral hypervalent organoiodines, which led to the development and testing of various strategies and numerous chiral organoiodines.^47−53^ This innovation has created new possibilities in the field of transition metal-free coupling.

**Figure 1:**
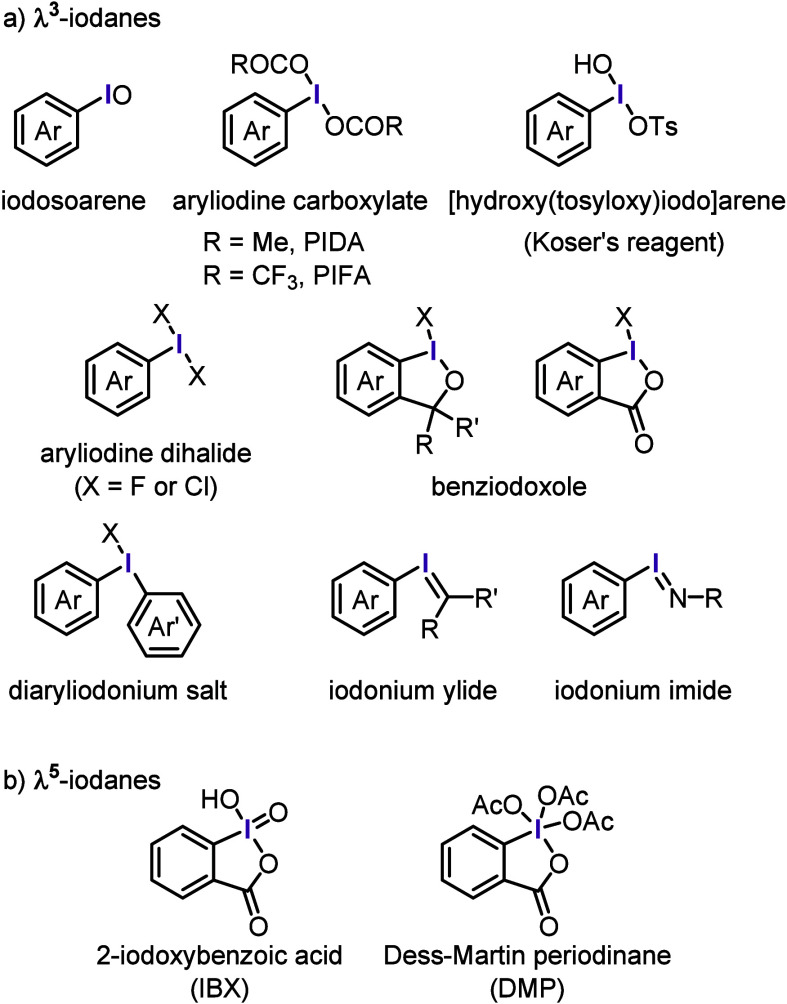
Hypervalent iodine reagents employed in organic synthesis.

Hypervalent organoiodines are categorized into two classes, λ^3^- and λ^5^-iodanes, based on the oxidation state of the central iodine atom (Figure 1). For instance, iodosoarenes, aryliodine carboxylates, and aryliodine organosulfonates are widely used as strong oxidizing reagents and are classified as λ^3^-iodanes. Among these compounds, phenyliodine diacetate (PIDA, PhI(OAc)_2_), phenyliodine bis(trifluoroacetate) (PIFA, PhI(OCOCF_3_)_2_), and [hydroxy(tosyloxy)iodo]benzene (HTIB, Koser’s reagent) are frequently employed in oxidative transformations. In contrast, aryliodine dihalides (X = F or Cl) are effective halogenating agents. Benziodoxoles are more stable than their acyclic counterparts and are valuable reagents for transferring functional (e.g., trifluoromethyl, ethynyl, cyano, and azido) groups. Diaryliodonium salts exhibit diverse reactivity because of the remarkable leaving ability of the iodoarene moiety and are, therefore, helpful aryl-group-transfer agents. Iodonium ylides and imides are excellent generators of carbene and nitrene species, respectively. The most essential λ^5^-iodanes in organic synthesis are 2-iodoxybenzoic acid (IBX) and the Dess–Martin periodinane (DMP), which are mild and highly selective reagents for the oxidation of alcohols.

Hypervalent iodine compounds, some of which are known for their explosive nature, have been discussed in review articles.^16−19^ Pentavalent iodine reagents, such as IBX, DMP, and iodylarenes (ArIO_2_), can explode upon heating or impact. Various analogs of IBX have been developed and are summarized in the literature.^18,19^ In contrast, trivalent iodine reagents are relatively stable and less explosive. The frequently used reagents, such as PhIO, PIDA, PIFA, and HTIB, can be stored over long-term and are commercially available. However, drying PhIO at high temperatures causes disproportionation to PhI and PhIO_2_, which has the possibility of severe explosion. *N*-Tosyliminoiodanes are stable and can be stored for extended periods, whereas trifluoroacetate and mesyl derivatives are thermally sensitive and explosive. Benziodoxoles have higher thermal stability, enabling the development of various stable atom-transferring reagents including even those containing the azide group. In addition, diaryliodonium salts generally exhibit high stability, allowing them to be stored for extended periods. To the best of our knowledge, no explosive properties of typically used diaryliodonium salts have been reported so far.

**Figure 2:**
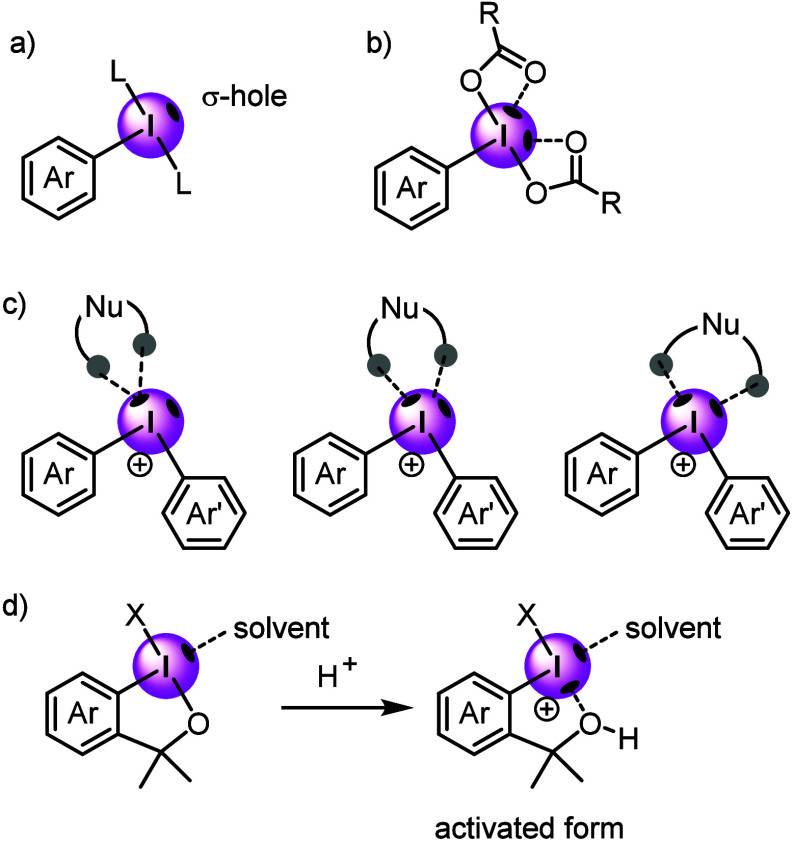
(a) Classical σ-hole of λ^3^-iodane, (b) nonclassical σ-hole with two maxima, (c) interaction with bidentate nucleophiles, (d) activation of benziodoxoles by acids.

Reactions involving hypervalent iodine reagents are initiated by the coordination of the nucleophile with the central iodine atom,^54^ which is highly Lewis-acidic and reactive owing to the presence of a region of positive electrostatic potential known as the σ-hole. This σ-hole not only affects the reactivity of hypervalent iodine but also facilitates molecular assembly through inter-/intramolecular secondary interactions, or halogen bonds, with nucleophilic regions. These anisotropic properties of iodine, particularly the electrophilic σ-holes, are essential for understanding the ability of hypervalent iodine species to engage in secondary interactions and halogen bonding. The number of σ-holes in λ^3^-iodanes can be one or two, whereas up to four are possible in λ^5^-iodanes (Figure 2).^55^

The classical σ-hole λ^3^-iodane is generated along with the extension of the primary σ-bond (Figure 2a). The σ-hole strength is determined by the nature of the primary two-center-two-electron σ-bond between the equatorial aryl group and the central I(III) atom, while the shape, direction, and splitting of the σ-hole are controlled by the three-center-four-electron hypervalent bond between the axial ligands and the I(III) atom. In contrast, the crystal structures of iodoarene dicarboxylates exhibit two additional secondary interactions (halogen bonds) between the carbonyl oxygens and the central iodine atom,^56−59^ which are attributed to the presence of a nonclassical σ-hole with two maxima on the I(III) atom surface (Figure 2b).^54,55^ More than one nucleophile can bind to either the same σ-hole or the less electronegative area between two σ-holes (Figure 2c).^60,61^ When the bite angle falls within the range of 34.3–46.1°, a bidentate nucleophile can bind to one of the two σ-holes on the iodonium(III) cation. If the bite angle is 54.9–77.6°, a bidentate nucleophile can bind to two σ-holes or even one σ-hole and the region between the two σ-holes. Benziodoxoles, including Togni reagents (X = CF_3_),^62^ feature a weak interaction between the moderately strong σ-hole on the I(III) atom and the solvent, such as acetonitrile (Figure 2d).^54^ Upon activation by a Brønsted acid, the characteristics of the hypervalent bond change to induce the formation of a highly electrophilic disubstituted iodonium cation with two localized σ-bonds that generate two strong σ-holes. One of these σ-holes forms an intramolecular halogen bond with the generated OH group, while the other can more tightly coordinate the incoming solvent with excellent proximity to the coupling partner X^+^.

The groups of Mayer and Legault developed a Lewis acidity scale for diaryliodonium salts based on the equilibrium constants for the complexation of a broad range of iodonium salts by Lewis bases, such as halides, carboxylates, phenolates, amines, and tris(*p*-anisyl)phosphine.^63,64^ Notably, cyclic diaryliodonium salts exhibited Lewis acidities approximately 2 orders of magnitude greater than those of their acyclic counterparts. The Lewis acidities of diaryliodonium salts were found to be comparable with that of Schreiner’s thiourea, while the theoretically predicted Lewis acidity of common cationic iodine(III) species (PhI^+^X, X = OH, Cl, F, OAc, OTs, OTf) was found to be similar or even stronger than those of widely used Lewis-acidic catalysts, such as BF_3_·OEt_2_ and TiCl_4_. The characteristics of λ^3^-iodanes explain the high electrophilicity/Lewis acidity of the central iodine, conservation of the configuration around the I(III) atom, and strong complexation in the crystal lattice and solution during the reaction, which considerably influences the susceptibility to reductive elimination.^65−69^ Consequently, diaryliodonium salts have been employed as arylation reagents or halogen bond–donor and/or Lewis-acidic organocatalysts^70−73^ to achieve diverse transformations.

This section focuses on the transition metal-free activation of aryl–iodide bonds through the oxidative activations of aryl iodides into λ^3^-iodanes. Among all possible λ^3^-iodanes, diaryliodonium salts can undergo transition metal-free dissociation of the Ar–I(III) bond and transfer aryl groups through the reductive elimination of hypernucleofuge aryl-λ^3^-iodane groups (hyperleaving groups).^21−23^ Aryliodonium ylides can also transfer aryl groups, as demonstrated by several examples in this section.

### 2.1 Breakthroughs in the Utilization of Diaryliodonium Salts

**Scheme 1:**
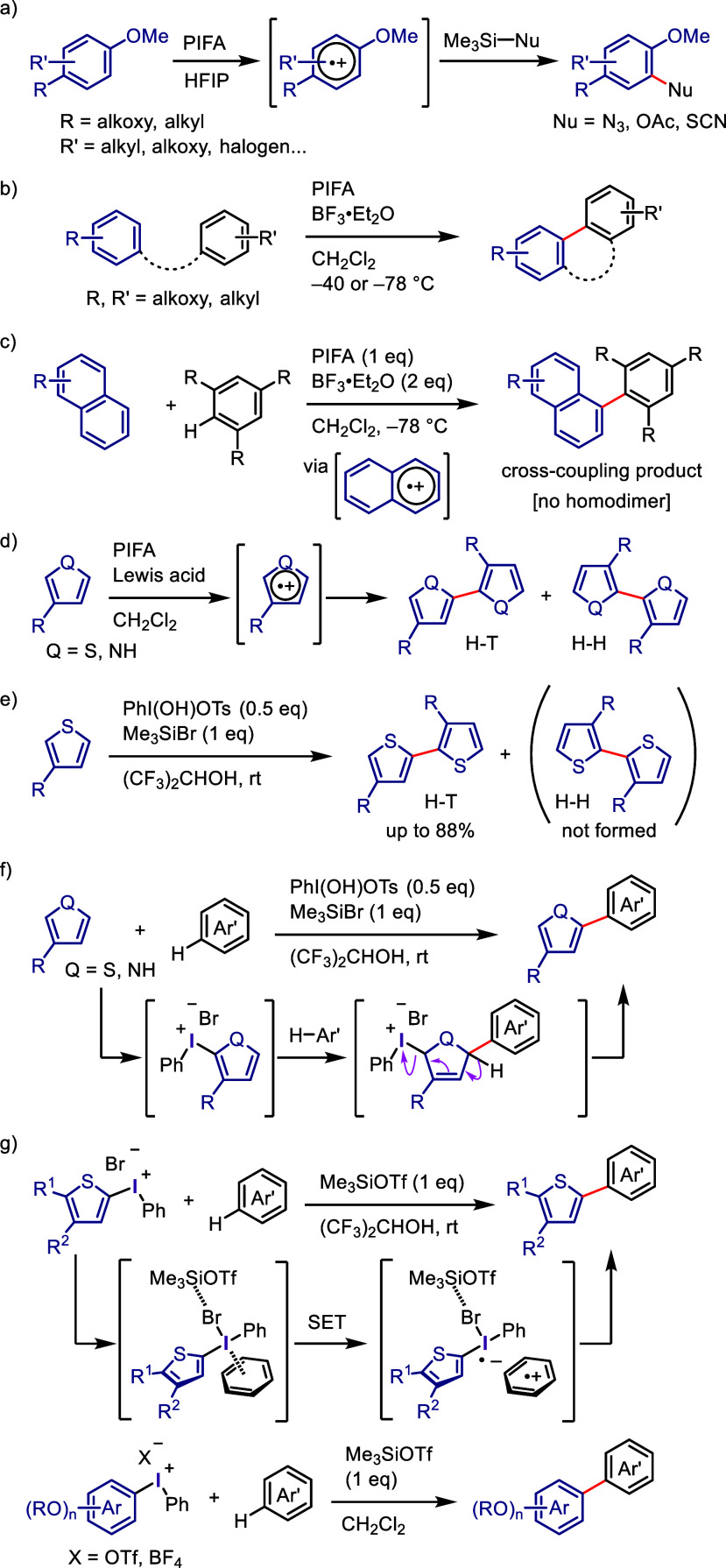
Evolution of Transition Metal-Free Aryl C–H Functionalization Involving Hypervalent Iodine by Kita et al.

Until about 35 years ago, hypervalent iodine reagents were reported to exhibit reactivity similar to that of many metal oxidants, and numerous organic synthetic reactions were reported using these reagents.^16,74−76^ In the mid-1980s, Kita et al. used hypervalent iodine reagents, particularly PIDA and PIFA, as environmentally friendly oxidants to replace toxic heavy metal oxidants, such as mercuric diacetate, thallium triacetate, and lead tetraacetate. They also revealed the unique reactivity of hypervalent iodine and its potential as an alternative to transition metal catalysts. Using these reactions, they synthesized numerous natural products, leading to a paradigm shift in the use of hypervalent iodine.^77,78^ Additionally, Kita et al. found that PIFA in 1,1,1,3,3,3-hexafluoroisopropanol (HFIP)^79^ enables the oxidation of aromatic compounds via SET-induced aryl C–H functionalization with various nucleophiles (Scheme 1a).^80−85^ The involvement of aromatic cation radicals was confirmed by ultraviolet (UV) and electron spin resonance (ESR) spectroscopies.^81,82^ The same group extended the metal-free oxidative coupling to intra- and intermolecular biaryl synthesis via C–H arylation using a combination of PIFA and a Lewis acid, such as boron trifluoride or trimethylsilyl bromide (Me_3_SiBr), in dichloromethane (Scheme 1b).^86−90^ Intermolecular biaryl cross-coupling was also achieved by choosing substrates that avoided homobiaryl formation (Scheme 1c).^91−93^

The combination of PIFA with a Lewis acid induced homocoupling of thiophenes and pyrroles. The resulting products were a mixture of regioisomers containing head-to-tail (H-T) and head-to-head (H-H) biaryls (Scheme 1d).^94−97^ After careful optimization of the reaction conditions, they disclosed that the use of HTIB and Me_3_SiBr in HFIP induced the regioselective homocoupling of 3-substituted thiophenes to generate H-T biaryls as a single isomer (Scheme 1e).^98,99^ Furthermore, the regioselective cross-coupling of unfunctionalized aromatic compounds was achieved to afford heterobiaryls (Scheme 1f).^100^ During the investigation of this reaction system, the authors noticed the generation of phenylthienyliodonium salts as the reaction intermediates, which react with the other arenes via S_N_2′-type substitution to furnish the coupling product. The isolated phenylthienyliodonium salts underwent biaryl cross-coupling in the presence of Me_3_SiBr to afford the same product. At nearly the same time, the authors discovered *ipso*-substitution of diaryliodonium bromides with electron-rich aromatic compounds in the presence of Me_3_SiOTf (Scheme 1g).^101,102^ In this reaction, the diaryliodonium salt serves as the SET oxidant to generate an aromatic cation radical, which was observed by ESR and UV spectroscopic measurements.

**Scheme 2:**
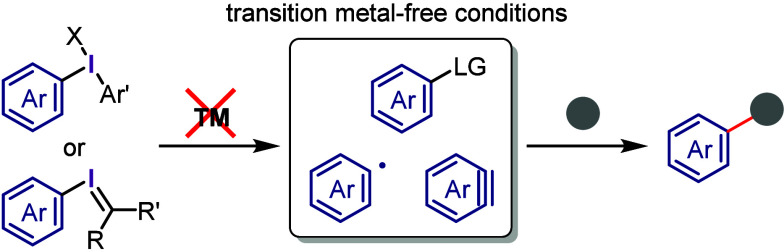
Diaryliodonium Salts Act as Aryl Cation-like Species, Aryl Radicals, or Aryne Precursors under Transition Metal-Free Conditions

Beringer initially reported research on the synthesis of diaryliodonium salts and their use in arylation in the 1950s.^103,104^ However, it received little attention for a long time. Around 2010, when Kita’s discoveries were reported in the literature, diaryliodonium salts saw renewed interest, and numerous studies on arylation reactions have since been conducted. Transition metal-catalyzed arylations of aromatic compounds were reported by Sanford and Gaunt, wherein diaryliodonium salts serve as both aryl source and oxidant for the catalysts to generate high-valent aryl–metal species via oxidative addition.^105−113^ By contrast, transition metal-free arylation using diaryliodonium salts involves various activated aryl species, such as aryl electrophiles, aryl radicals, and aryne equivalents, which can be captured by a range of nucleophiles to form C–C and C–heteroatom bonds, as demonstrated by several chemists (Scheme 2). Numerous reviews published in the past decade have covered topics related to transition metal-free and metal-catalyzed reactions, in which diaryliodonium salts act as efficient substrates,^114−117^ including the double functionalization of C–I(III) and *ortho* C–H bonds.^118,119^

The current section summarizes the latest progress in transition metal-free arylations with diaryliodonium salts as aryl cation-like species (Section 2.3), aryl radicals (Section 2.4), and aryne equivalents (Section 2.5). Arylation using diaryliodonium salts generates a stoichiometric amount of iodoarenes, thereby reducing the atom economy. We also introduce two strategies to minimize waste: the incorporation of the iodoarene into final products via intramolecular or sequential reactions (Section 2.6) and the recyclable use of iodoarene auxiliaries (Section 2.7).

### 2.2 Oxidative Activation of Aryl Iodide for Diaryliodonium Salt Synthesis

Diaryliodonium salt synthesis typically begins with the oxidative activation of aryl iodides followed by the coupling of the hypervalent iodine(III) species with arene (Ar-H) or organometallic arene (Ar-M) reagents. Various approaches have been developed to synthesize diaryliodonium salts under mild and accessible conditions.^115−117,120,121^ Hence, the following part describes the commonly used and updated strategies for producing diaryliodonium salts.

**Scheme 3:**
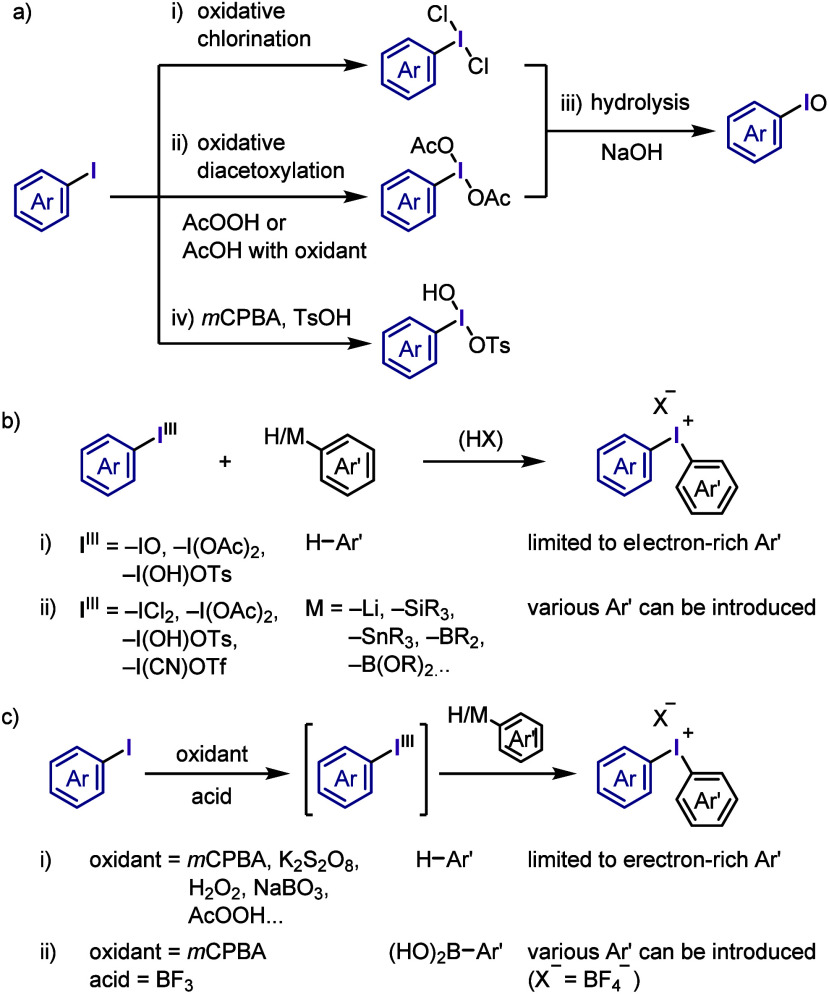
Oxidative Activation of Aryl Iodide and Preparation of Diaryliodonium Salts

The generation of hypervalent iodine from aryl iodide has been summarized by Zhdankin and Stang in previously reported reviews.^17,18^ Oxidation of iodoarenes has been classically performed via oxidative chlorination to generate aryliodine dichloride (Scheme 3a-i),^122^ which is an unstable yellow solid unsuitable for long-term storage at low temperatures. Relatively stable aryliodine diacetates are generally synthesized via oxidative diacetoxylation using peracetic acid in acetic acid (Scheme 3a-ii).^123^ Alternatively, the combination of acetic acid with various oxidants, such as sodium periodate,^124^ sodium percarbonate,^125^ potassium peroxodisulfate,^126^ sodium perborate,^127−129^ and *m*-chloroperbenzoic acid (*m*CPBA), is used.^130,131^ Hydrolysis of aryliodine dichloride and diacetate under basic conditions affords the corresponding iodosoarenes (Scheme 3a-iii).^132,133^ [Hydroxy(tosyloxy)iodo]arene, another common and essential hypervalent iodine(III) compound, has been classically synthesized via the generation of aryliodine diacetate followed by treatment with *p*-toluenesulfonic acid monohydrate (*p*TsOH·H_2_O).^134^ One-pot synthesis via oxidation of iodoarene by *m*CPBA in the presence of *p*TsOH·H_2_O provides a more convenient procedure (Scheme 3a-iv).^135,136^

Dehydrative condensation of aromatic compounds with these hypervalent iodine(III) compounds, such as iodosoarenes,^101,137,138^ (diacetoxyiodo)arenes,^139−141^ and [hydroxy(tosyloxy)iodo]arenes,^142−146^ affords diaryliodonium(III) salts, which is the simplest and cleanest synthesis (Scheme 3b-i). Given that these reactions involve electrophilic substitution, the starting materials are limited to electron-rich aromatic compounds. A metal–I(III) exchange strategy using organometallic aryl nucleophiles, such as aryllithiums, -silanes, -stannanes, -boronic acids, and -boronates, has been developed to prepare various diaryliodonium salts (Scheme 3b-ii).^147−155^

One-pot syntheses of diaryliodonium salts including the oxidation of the corresponding iodoarenes followed by dehydrative condensation have also been developed. The combination of an organic oxidant, such as *m*CPBA, and a strong acid (TfOH, TsOH, or TFA) was used to achieve the one-pot syntheses of a wide range of commonly used diaryliodonium salts in high yields (Scheme 3c-i).^141,156−162^ The choice of *m*CPBA as the organic oxidant enabled the purification of iodonium salts by simple trituration with diethyl ether. The K_2_S_2_O_8_/TFA combination was employed for the in situ generation of diaryliodonium trifluoroacetate followed by anion exchange with TfONa to afford the corresponding triflate.^163−165^ In addition, urea–H_2_O_2_/Tf_2_O and Oxone/TfOH (oxidant/acid) combinations were successfully used for the one-pot preparation of diaryliodonium triflates.^166,167^ NaBO_3_·H_2_O/Ac_2_O/H_2_SO_4_ and Oxone/H_2_SO_4_ combinations were employed for the one-pot synthesis of diaryliodonium bromides after anion exchange of the generated diaryliodonium hydrogen sulfates in the presence of KBr.^168,169^ Dohi et al. developed a practical synthesis of diaryliodonium acetates bearing the trimethoxyphenyl group as an aryl group, which involves sequential oxidation of iodoarene with peracetic acid followed by condensation with trimethoxybenzene in 2,2,2-trifluoroethanol (TFE).^170^ These one-pot strategies for the preparation of diaryliodonium salts, including the oxidation of iodoarenes and condensation with aromatic compounds, have been applied to flow and electrochemical syntheses.^171−178^ Olofsson et al. designed a one-pot reaction of iodoarenes with arylboronic acids in the presence of *m*-CPBA/BF_3_-OEt_2_ for the regiospecific synthesis of diaryliodonium salts (Scheme 3c-ii).^179^ They also updated the one- and two-pot protocols to be more tolerant of electron-rich arenes, electron-deficient iodoarenes/unactivated arenes, and electron-deficient iodoarenes/electron-deficient arylboronic acids in the syntheses of iodonium triflates, tosylates, and tetrafluoroborates, respectively.^180^ Additional anion exchange may be crucial for preparing difficult-to-access iodonium salts or for changing the counteranions of the iodonium salts to modify their physical and chemical properties.^181^ Cyclic diaryliodonium salts are typically prepared through oxidation of the *ortho*-iodoarene scaffold in the presence of an acid, generating a hypervalent iodine intermediate followed by an intramolecular electrophilic aromatic substitution reaction with the neighboring aryl moiety. This topic has been comprehensively discussed in several recent reviews.^118,182−185^

### 2.3 Arylation of Carbon and Heteroatom Nucleophiles via Ligand Coupling

The investigation of transition metal-free arylation can be traced back to the seminal report of Beringer in 1953, who used diaryliodonium salts to arylate various nucleophiles, such as hydroxide, alkoxides, phenoxides, benzoate, nitrite, sulfonamides, amines, diethyl oxalacetate, sulfite, sulfinate, and cyanide.^103,104^ This method enabled the formation of a wide range of C–C and C–heteroatom bonds from a limited range of substrates, and the reactions were conducted in refluxing polar or mixed solvents, affording arylation products in poor to good yields. The findings of this work provided new opportunities for transition metal-free cross-arylations based on the development of sustainable and ecofriendly methods for constructing diverse chemical bonds under benign conditions.

The related recent studies have primarily aimed to develop chemoselective iodonium salts using a cost-effective and widely available dummy group to decrease costs as well as recycle aryl iodide waste, conducting the process under mild conditions (base, solvent, and temperature) with stoichiometric amounts of reactants, and identify conditions compatible with a broad range of nucleophiles and iodonium salts. Furthermore, the significance of this chemistry for delivering organic compounds suitable for further functionalization, addressing challenges associated with previously reported strategies, and transferring the process from the synthetic community to the manufacturing society for multigram-scale reactions, synthesis of pharmaceutical drugs/ingredients, and late-stage functionalization of valuable compounds is also targeted. The following sections commence with a succinct introduction followed by a discussion of work published largely after 2017.

#### 2.3.1 Arylation via Ligand Exchange and Coupling Mechanism

**Scheme 4:**
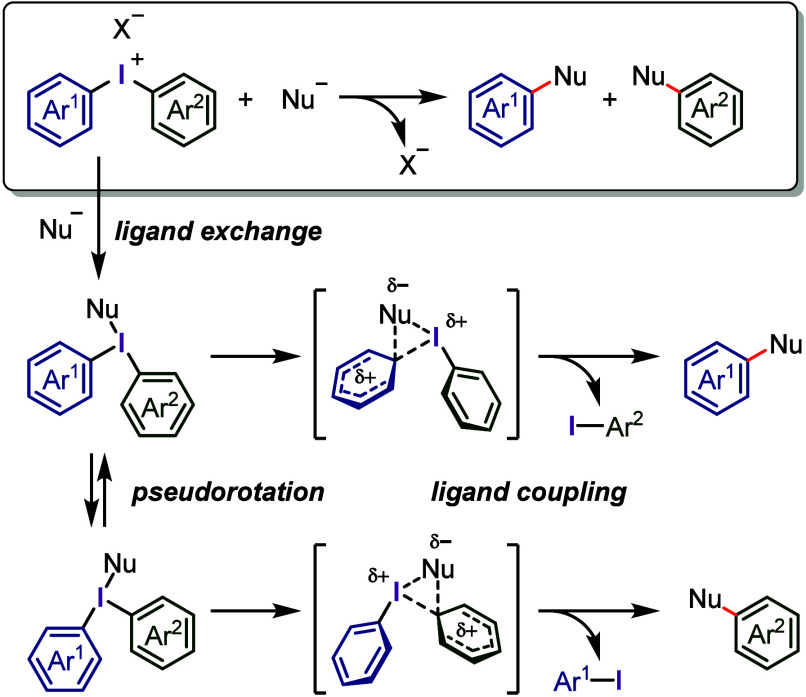
Possible Mechanism of the Reaction of Diaryliodonium Salts with Nucleophiles

The reactions between diaryliodonium salts and nucleophiles afford aryl–nucleophile bonds under transition metal-free conditions (Scheme 4).^15,117,186^ These reactions involve the exchange of the counteranion of the iodonium salt with the nucleophile, which affords two T-shaped Ar^1^(Ar^2^)I-Nu intermediates in equilibrium via pseudorotation. The nucleophile is transferred to the more approachable equatorial aryl group through a concerted *ipso*-substitution mechanism involving the formation of a three-center-four-electron transition state followed by the reductive elimination of the aryl iodide and coupling product formation.^187−189^ The aryl group selectivity is thought to be determined during the ligand coupling step, specifically through the migration of the nucleophile from the I(III) atom to the adjacent *ipso*-carbon of the equatorial aryl group.

The reactions of diaryliodonium salts with nucleophiles are sustainable alternatives to metal catalyst-promoted C–C and C–heteroatom bond formation. Considerable progress has been made in transition metal-free arylations using diaryliodonium salts under benign conditions, although this approach suffers from the production of stoichiometric amounts of aryl iodides as waste, which can be costly if expensive starting materials are used. The chemoselectivity of transition metal-free cross-coupling reactions involving diaryliodonium salts with two different aryl ligands can be challenging to predict and is primarily influenced by the nature of the nucleophile and diaryliodonium salt.^190^ Generally, electron-deficient aryl ligands are preferred for electrophilic transfer because of their ability to better stabilize the negative charge formed in the transition state of reductive elimination.^191^ In addition, other factors, such as steric effects, including the *ortho*- and *anti*-*ortho*-effects, should be considered when discussing the chemoselectivity of this process.^186,190,192^

**Scheme 5:**
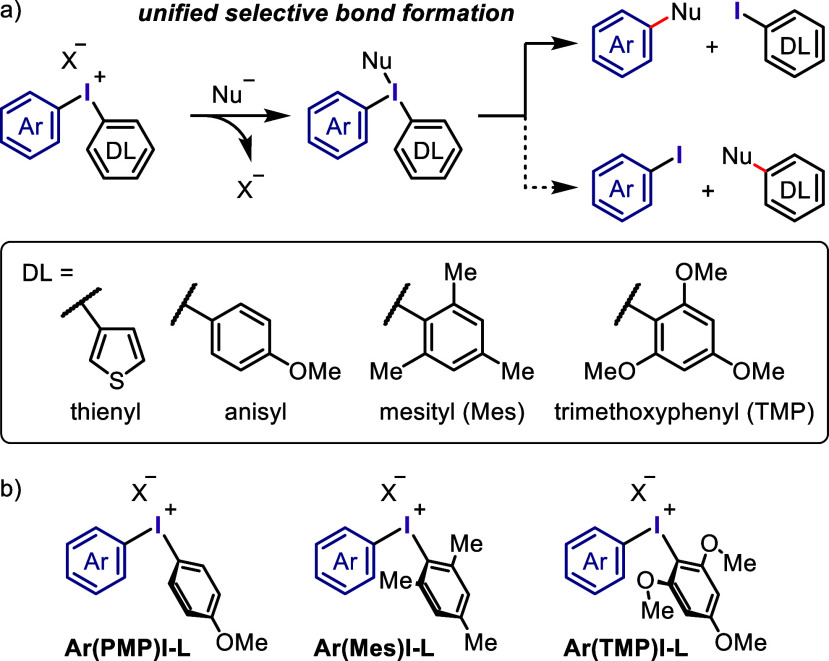
Dummy Ligand Strategy Used for the Unified Selective Arylation of Nucleophiles

Several auxiliary or dummy aryl groups have been used to increase chemoselectivity (Scheme 5a). The selection of dummy ligands depends on the other aryl or heteroaryl group of the diaryliodonium salt. To date, auxiliary diaryliodonium salts with one dummy aryl ligand, such as thienyl, *p*-methoxyphenyl (PMP), mesityl (Mes), and trimethoxyphenyl (TMP) groups, have been employed.^156,189,192−194^ Among then, TMP was found to be the most electron-rich and, hence, most suitable for enhancing chemoselectivity in the direct reactions of nucleophiles. This dummy ligand is derived from a commercially available and inexpensive compound, making diaryliodonium salt synthesis more facile and productive. Additionally, the dummy ligand allows for the chemoselective transfer of the aryl group during arylation to form the desired product in a controlled manner and produce an inexpensive dummy aryl iodide coproduct instead of a wasteful aryl iodide.

In this review, aryliodonium salts incorporating these dummy ligands are expressed using abbreviations to facilitate visual understanding (Scheme 5b). Specifically, aryl(4-phenyl)iodonium salt is represented as aryl(PMP)iodonium salt or **Ar(PMP)I-L**, aryl(2,4,6-trimethylphenyl)iodonium salt as aryl(Mes)iodonium salt or **Ar(Mes)I-L**, and aryl(2,4,6-trimethoxyphenyl)iodonium salt as aryl(TMP)iodonium salt or **Ar(TMP)I-L**, where L represents the counteranion.

#### 2.3.2 C–O Bond Formation

**Scheme 6:**
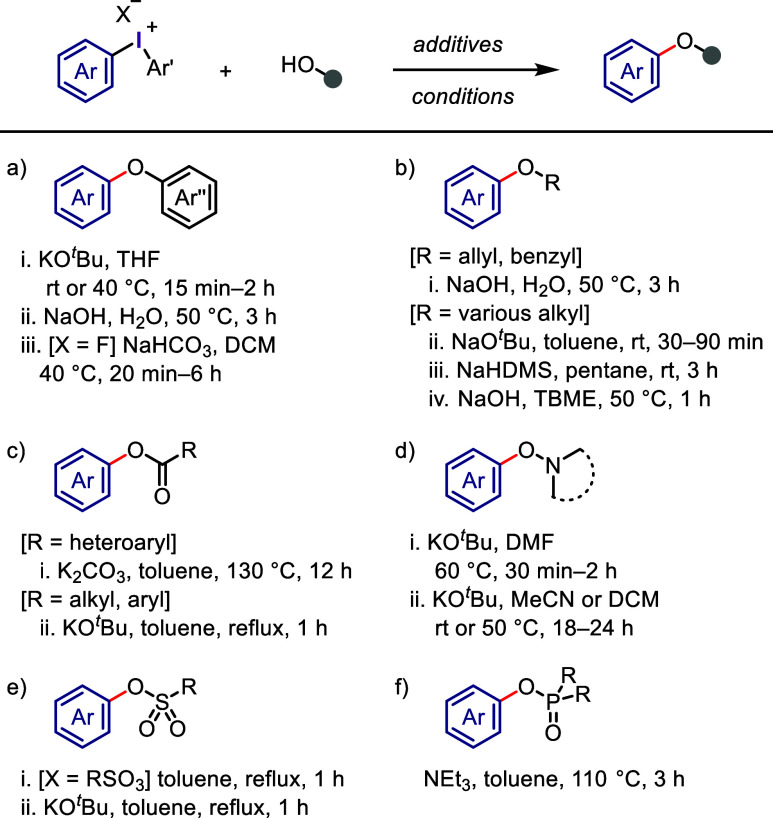
General Conditions for *O*-Arylations with Diaryliodonium Salts

Considerable progress has been achieved in the development of diaryliodonium salts as effective agents for the arylation of diverse OH groups under transition metal-free mild conditions. Olofsson et al. expanded the arylation scope to include phenols and iodonium salts under both aqueous and nonaqueous conditions (Schemes 6a-i and -ii).^195−197^ Gaunt et al. emphasized the essential role of the fluoride counteranion in the iodonium salt in activating the phenolic OH group through hydrogen bonding, which allows *O*-arylation reactions to be carried out using a weak base, NaHCO_3_ (Scheme 6a-iii).^198^ Olofsson et al. aimed to provide optimal aqueous and nonaqueous conditions for the OH-arylation of various types of alcohols, including primary, secondary, tertiary, benzylic, and allylic ones, as well as carbohydrates (Schemes 6b-i and -ii).^196,199,200^ Additionally, sodium hexamethyldisilazide (NaHMDS) was found to be a more effective base than KO^*t*^Bu for coupling highly sterically congested systems comprising both tertiary alcohols and iodonium salts with different aryl groups (Scheme 6b-iii).^201^ Stuart et al. used aryl(Mes)iodonium bromides or aryl(TMP)iodonium tosylates for the highly chemoselective arylation of diverse aliphatic and aromatic alcohols in the presence of NaH as a base (Scheme 6b-iv).^202,203^ The arylation of heteroaromatic carboxylic acids, such as indolecarboxylic acids, can be effectively achieved using simple iodonium salts at elevated temperatures to produce aryl esters (Scheme 6c-i).^204^ Additionally, KO^*t*^Bu was found to be an effective base for mediating the *O*-arylation of aromatic and aliphatic carboxylic acids, as well as *N*-hydroxysuccinimide and phthalimide (Schemes 6c-ii and -iii).^197,205,206^ This approach was later extended to include *N*-hydroxybenzotriazoles and 3-hydroxybenzotriazin-4-ones, enabling the [3,3]-sigmatropic rearrangements of the resulting *O*-arylated products (Scheme 6d).^207−209^ The transfer of the sulfonate counterion was successful in moving to the adjacent aryl moiety within the diaryliodonium sulfonate salt, resulting in the production of aryl sulfonate esters in high yields (Scheme 6e-i). In contrast, the two-component reaction of arylsulfonic acids with diaryliodonium triflates afforded coupling products in moderate yields (Scheme 6e-ii).^197^ Furthermore, diaryliodonium triflates were used to arylate various -P(O)-OH substrates, including phosphinic acid, hydrogen phosphate, and hydrogen phosphonate, which afforded the corresponding aryl esters in excellent yields (Scheme 6f).^210^

**Scheme 7:**
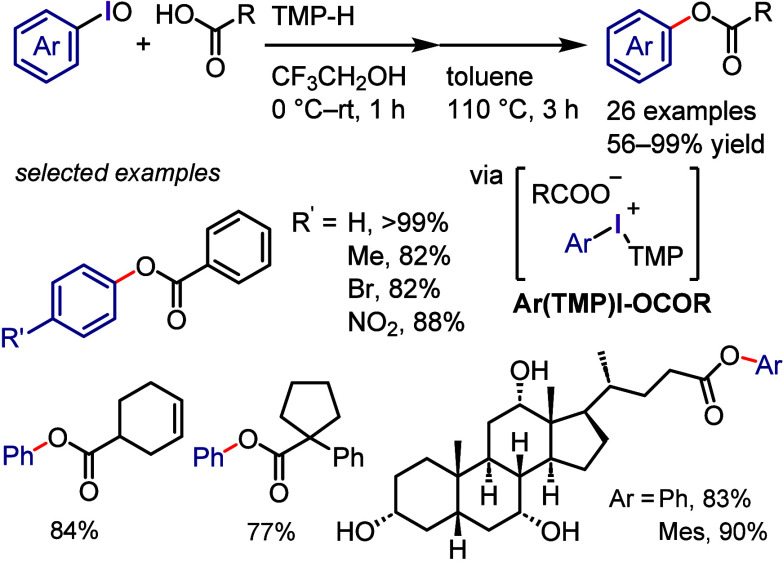
*O*-Arylation of Carboxylic Acids via the In Situ Generation of Iodonium Carboxylates

The Dohi and Kita group has developed a convenient base-free and step-economical strategy for the *O*-arylation of carboxylic acids involving the in situ generation of aryl(TMP)iodonium carboxylates (**Ar(TMP)I-OCOR**) via the one-pot reaction of iodosobenzene with 1,3,5-trimethoxybenzene (TMP-H) and a carboxylic acid (Scheme 7).^138^ The subsequent heating of the thus generated aryl(TMP)iodonium carboxylates led to ligand coupling and the formation of the desired arylcarboxylate ester. This process was applicable to a wide range of carboxylic acids, including electron-rich, electron-deficient, and sterically congested (hetero)aryl ones, as well as aliphatic carboxylic acids and natural products, such as cholic acid, even in the presence of multiple hydroxyl groups.

**Scheme 8:**
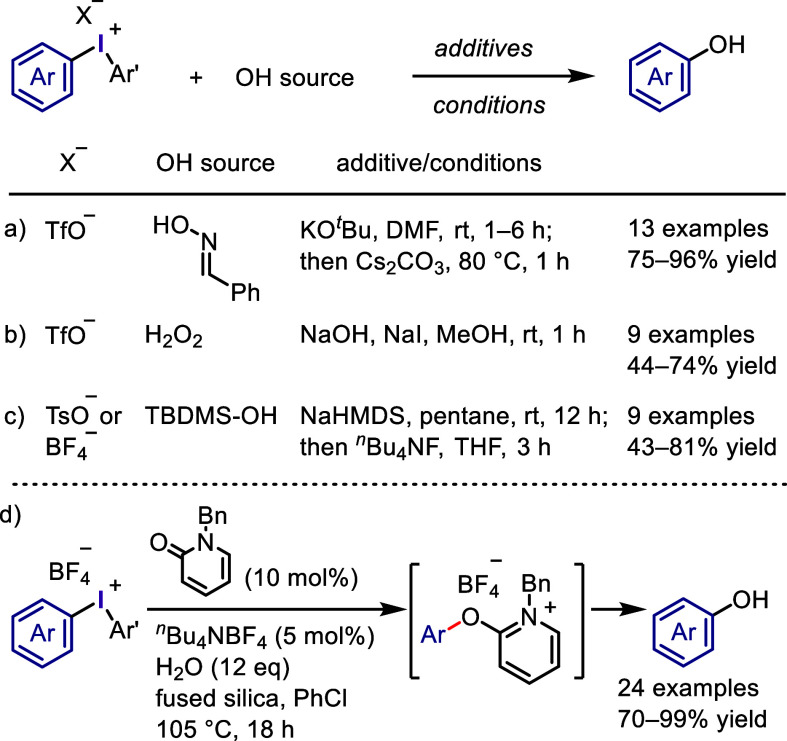
Hydroxide Equivalents Used for Coupling with Iodonium Salts and Phenol Formation

The direct arylation of hydroxide ions with diaryliodonium salts is challenged by the frequent formation of regioisomeric aryl ethers instead of the intended phenols.^187^ The highly basic conditions of the reaction facilitate competing processes, such as S_N_Ar reactions and the generation of aryne intermediates. This process is also accompanied by the formation of undesired aryl ethers due to the propensity of phenols to undergo further arylation under the employed conditions. To address this problem, Zhou et al. used oximes, which are nucleophiles commonly employed in one-pot, two-step cross-coupling reactions with diaryliodonium salts as a hydroxide surrogate (Scheme 8a).^211−216^ Initially, diaryliodonium salts (Ar = Ar′) were used in C–O couplings with oximes to generate *O*-arylated oximes in situ, with subsequent treatment with Cs_2_CO_3_ upon heating producing the desired phenols. The Olofsson group used hydrogen peroxide and silanol as hydroxide surrogates in coupling reactions with diaryliodonium salts to generate phenols (Schemes 8b and 8c).^217^ The reaction conditions were optimized to achieve exclusive regioselectivity for the desired phenols. When hydrogen peroxide was used, the generated phenoxide intermediate and resulting phenol could not undergo further arylation under the applied conditions. Only iodonium salts with moderately electron-donating substituents and electron-deficient iodonium salts with only one phenyl group were tolerated, whereas aryl transfer groups or electron-rich dummy ligands were not suitable.

Onomura and Kuriyama developed an efficient catalytic method for synthesizing phenols by coupling diaryliodonium salts with water in the presence of an organocatalyst, *N*-benzylpyridin-2-one, under mild conditions (Scheme 8d).^218^ This process commences with the *O*-arylation of the catalyst to form a 2-aryloxypyridinium intermediate, which is subsequently hydrolyzed by water to produce the desired phenol and regenerate the catalyst. The addition of fused silica is essential for rendering the reaction catalytic, and ^*n*^Bu_4_NBF_4_ is necessary for improving the product yield.

**Scheme 9:**
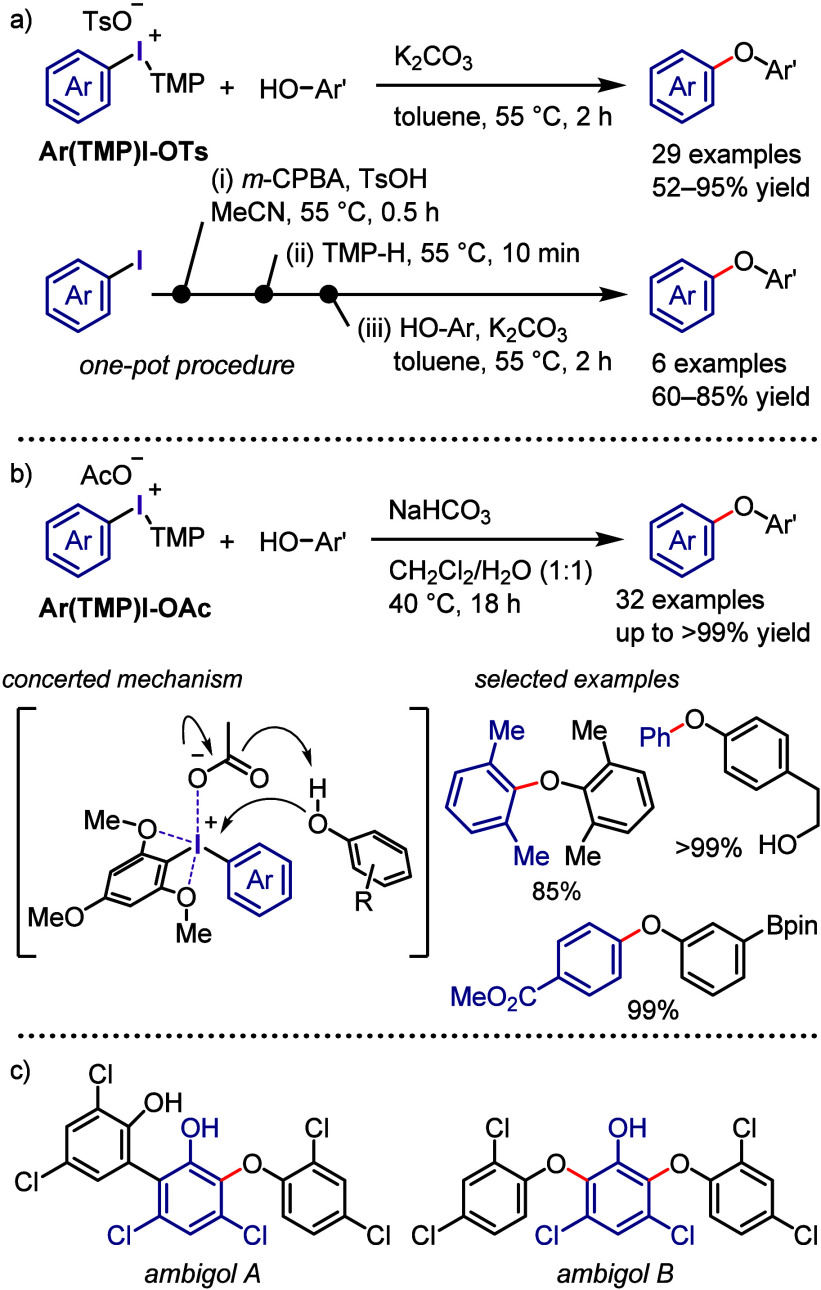
(a) Arylation of Phenols Using Ar(TMP)I-OTs, (b) *O*-Arylation of Phenols with Ar(TMP)I-OAc, (c) Ambigols Synthesized Using Diaryliodonium Salts

Aryl(TMP)iodonium tosylates (**Ar(TMP)I-OTs**) were utilized as effective reagents for the arylation of phenols, including a broad range of natural products (Scheme 9a).^219^ The TMP group in these salts facilitated their preparation and chemoselective aryl transfer during coupling, enabling the highly selective transfer of various electron-rich, electron-poor, sterically hindered, and heterocyclic aryl groups to produce the corresponding diaryl ethers. The one-pot sequential reaction, involving in situ aryl iodide oxidation, aryl(TMP)iodonium salt formation, and coupling with phenols, further expanded the versatility of this process.

Dohi and Kita reported the *O*-arylation of phenols with aryl(TMP)iodonium acetates (**Ar(TMP)I-OAc**), which acted as highly reactive aryl precursors.^220,221^ The TMP group, with its two *ortho* methoxy groups coordinating the central I(III) atom, facilitated the dissociation of the acetate counterion, enhancing acetate basicity and phenol nucleophilicity via deprotonation (Scheme 9b). The reaction demonstrated scalability, and substrates with various functional groups, including aliphatic alcohols and boronic esters, were well tolerated under the employed conditions. Gulder et al. used the *O*-arylation of phenols using TMP iodonium salts to prepare ambigols, which are natural products derived from cyanobacteria and exhibit intriguing biological activities (Scheme 9c).^222^

**Scheme 10:**
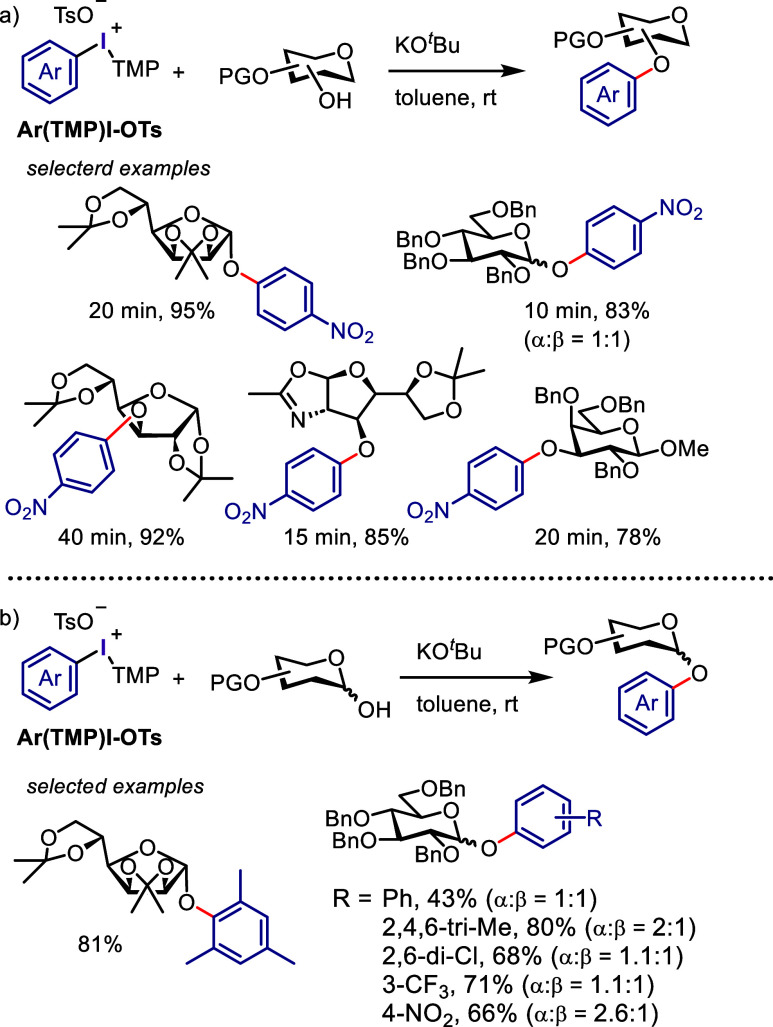
*O*-Arylation of Unactivated Carbohydrates with Diaryliodonium Salts

Olofsson et al. discovered a method for efficiently *O*-functionalizing carbohydrate derivatives under simple conditions using protected furanose and pyranose structures and successfully arylated them by aryl groups derived from diaryliodonium salts (Scheme 10a).^200,223^ The versatility of this process was showcased through the 2- and 7-fold arylations of glucose diol and cyclodextrin derivatives, respectively.

Gilmour et al. extensively researched the *O*-arylation of the anomeric hydroxyls of unactivated sugars (Scheme 10b).^224^ Specifically, diaryliodonium salts were used to arylate the lactol positions of mono-, di-, and trisaccharides, with preferences for α- and β-anomers observed for benzyl- and *p*-methoxybenzyl-protected substrates, respectively. The stereochemistry of the starting materials was transferred to the product with complete stereoretention.

**Scheme 11:**
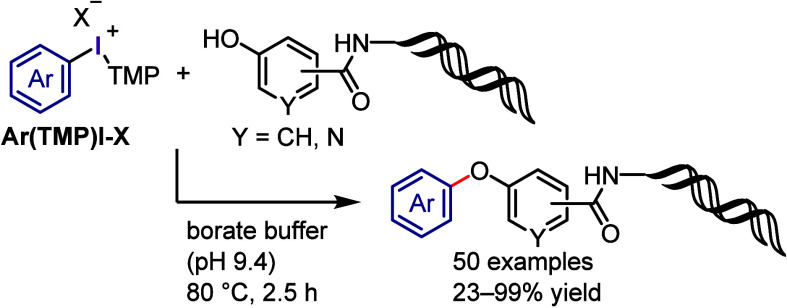
On-DNA *O*-Arylation of Phenols with Diaryliodonium Salts

Motivated by the high reactivity of diaryliodonium salts as transition metal-free arylating reagent, Xu et al. designed an effective process for arylating DNA-conjugated libraries of (hetero)aryl phenols and naphthols (Scheme 11).^225^ In this DNA-encoded library synthesis, the *O*-arylation of phenolic groups was accomplished in high yields and with high DNA fidelity in the presence of additional amidic groups. The scope of this method was expanded to include the late-stage *O*-arylation of tyrosine on DNA-conjugated peptides and synthesis of DNA-conjugated analogues of sorafenib.

**Scheme 12:**
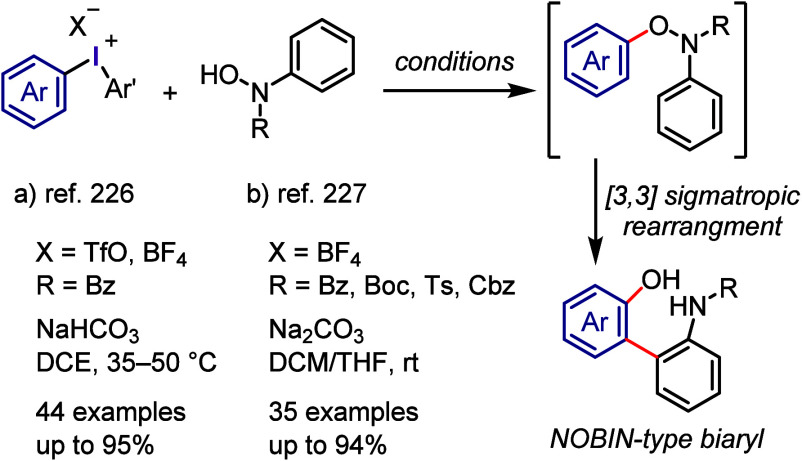
*O*-Arylation of *N*-Arylhydroxylamine and NOBIN-Type Biaryl Formation

Gao and Tan designed an *O*-arylation of *N*-arylhydroxylamines using diaryliodonium salts for the synthesis of 2-amino-2′-hydroxy-1,1′-binaphthyl (NOBIN)-type biaryls (Scheme 12).^226,227^ The *O*-arylation of *N*-arylhydroxylamines produced *N*,*O*-diarylhydroxylamines, which subsequently underwent a [3,3]-sigmatropic rearrangement and rearomatization to produce the corresponding biaryls.^228^

#### 2.3.3 C–N Bond Formation

**Scheme 13:**
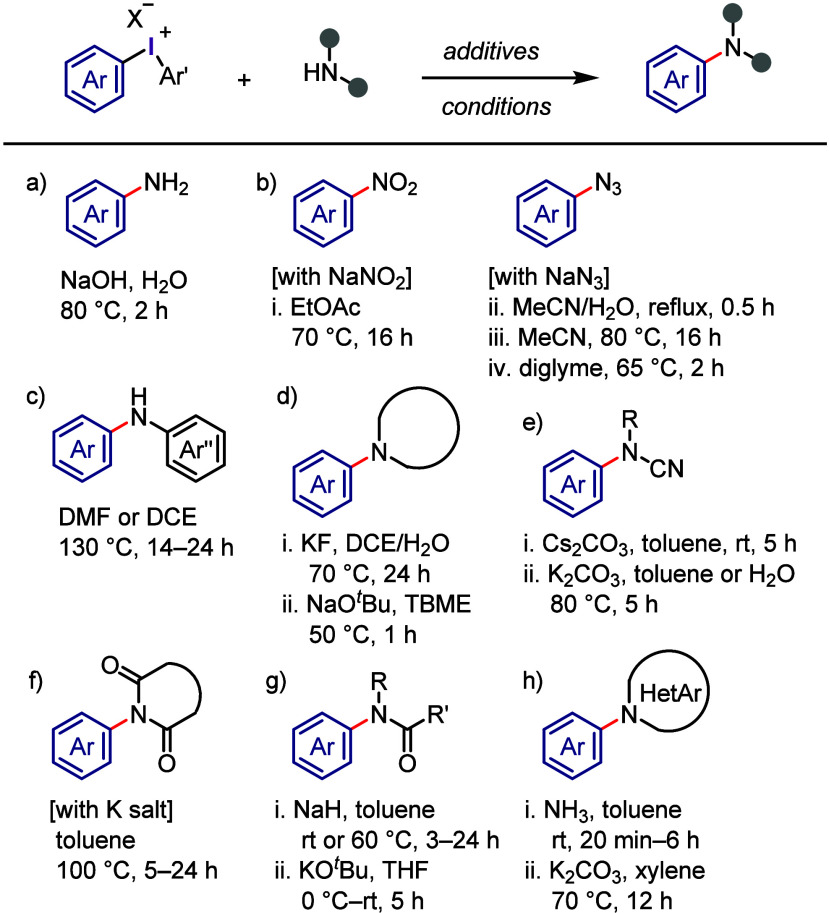
General Conditions for *N*-Arylations with Diaryliodonium Salts

Since the discovery of diaryliodonium salts by Beringer, their versatility as transition metal-free *N*-arylating agents has been significantly expanded to include a broad range of substrates and more convenient conditions.^104^ The arylation of aqueous ammonia under basic conditions was reported to produce only the related aniline and not di- or triarylation products (Scheme 13a). The nitrite anion was successfully *N*-arylated with a variety of diaryliodonium salts under base-free conditions (Scheme 13b-i).^229,230^ Additionally, useful aryl azides were prepared by the arylation of the azide anion with iodonium salts, such as electronically diverse aryl(TMP)iodonium tosylates, which acted as highly chemoselective arylating agents (Schemes 13b-ii–iv).^230−233^ The arylation of anilines with diaryliodonium salts can be achieved at high temperatures in the absence of bases (Scheme 13c).^234,235^ In contrast, the arylation of aliphatic cyclic secondary amines with aryl(TMP)iodonium trifluoroacetate can be accomplished under basic conditions, affording *N*-arylation products (Scheme 13d).^236,237^ Under basic conditions, aryl/alkyl cyanamides can be effectively arylated with diaryliodonium salts in aqueous or nonaqueous environments to generate diverse *N*-arylated cyanamides (Scheme 13e).^238,239^ Muñiz et al. used aryl(phenyl)iodonium salts and Mes_2_IOTf to arylate cyclic imides and amides and synthesize sterically hindered aniline derivatives (Scheme 13f).^240^ In this process, the bulky aryl group was transferred with excellent chemoselectivity. Additionally, Stuart et al. arylated phthalimide potassium salts with aryl(TMP)iodonium tosylates to yield *N*-arylation products with good chemoselectivity (Scheme 13F).^241^ Acyclic secondary amides were arylated by iodonium salts in the presence of bases to produce *N*-arylated amides (Scheme 13g).^242,243^ Specific *N*-heteroarenes, such as pyrazoles and 1,2,3-triazoles, were successfully reacted with diaryliodonium triflates under milder conditions to form the corresponding *N*-aryl heterocycles (Scheme 13h).^244,245^ According to density functional theory (DFT) calculations, the presence of a basic nitrogen atom adjacent to the arylated NH center is crucial for enhancing the reactivity required to carry out the reaction under mild basic conditions.^246^

**Scheme 14:**
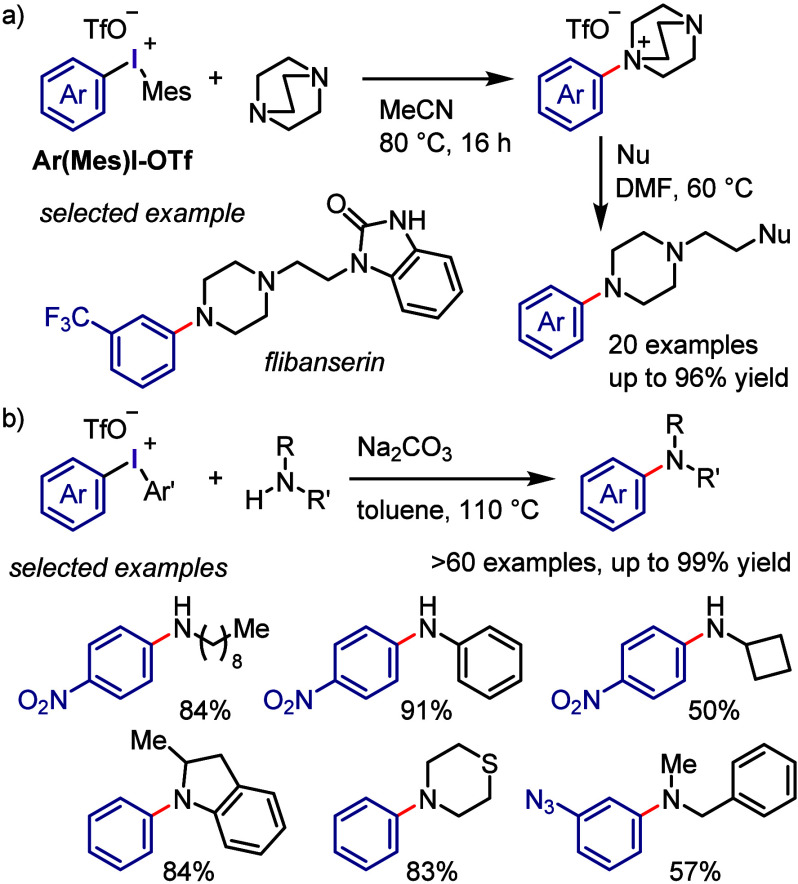
*N*-Arylation of DABCO and Formation of Quaternary Salts

Karchava et al. reported the successful arylation of a tertiary amine (DABCO) with electronically and sterically diverse aryl(Mes)iodonium triflates (Ar(Mes)I-OTf) (Scheme 14a).^247^ The obtained *N*-aryl-DABCO triflates afforded 1,4-disubstituted piperazines and flibaserin drugs when reacted with nucleophiles to induce ring opening. Olofsson et al. reported a method for the *N*-arylation of primary and secondary amines compatible with a wide range of diaryliodonium salts and amine nucleophiles (Scheme 14b).^248,249^

**Scheme 15:**
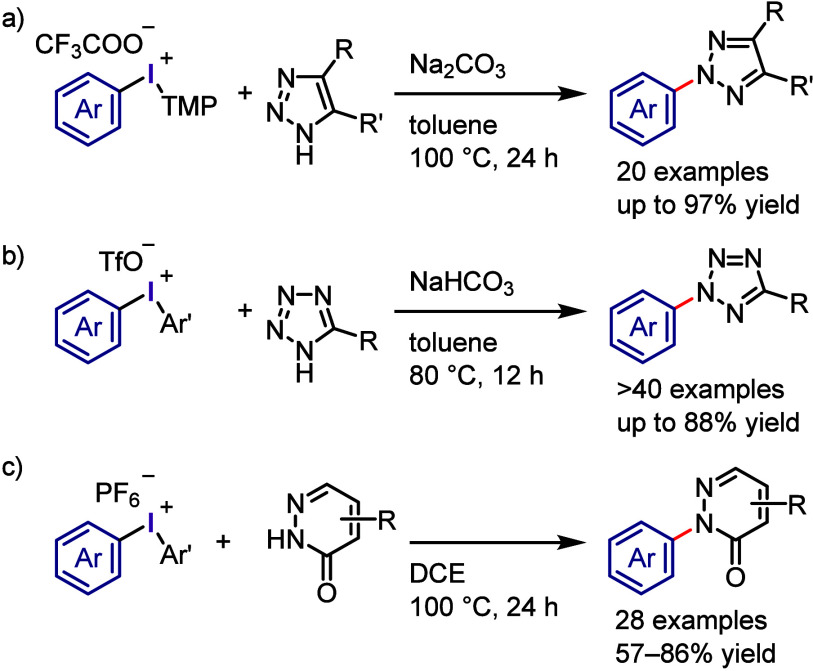
Regioselective *N*^2^-Arylation of Triazoles/Tetrazoles with Diaryliodonium Salts

Motivated by the reactivity of diaryliodonium salts as transition metal-free arylating reagents, Prakash et al. developed a method for the regioselective arylation of 1,2,3-triazoles at the challenging *N*(2) position (Scheme 15a).^250^ Under optimal conditions (aryl(TMP)iodonium salt, Na_2_CO_3_, toluene, and heating at 100 °C), diverse substituted and unsubstituted-1,2,3-triazoles were regioselectively arylated to produce *N*^2^-arylated products. The results of DFT calculations were in good agreement with the experimentally observed *N*^2^-regioselectivity. Similarly, *N*-tetrazoles were selectively arylated at the *N*^2^-position using diaryliodonium salts (Scheme 15b).^251^ The optimal conditions were compatible with a wide range of 5-substituted tetrazoles. Han and Wang reported a chemoselective *N*-arylation of pyridazin-3-ones under base-free conditions (Scheme 15c).^252^ The authors examined the reactivity of diaryliodonium hexafluorophosphate by demonstrating the remarkable selectivity of the *N*-arylation of a wide range of pyridazinones. Contrary to previous ionic mechanisms, these mechanistic investigations uncovered a free radical reaction pathway.

**Scheme 16:**
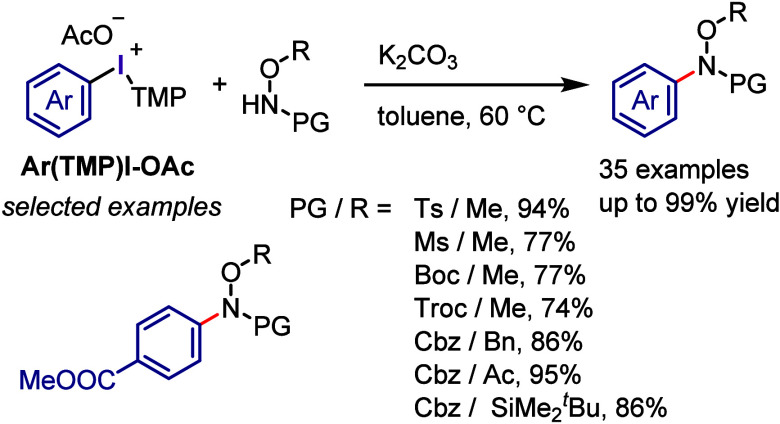
*N*-Arylation of Sulfonamides and Protected Hydroxylamines with Ar(TMP)I-OAc

The potential of aryl(TMP)iodonium acetates (Ar(TMP)I-OAc) for the *N*-arylation of *N*-methoxysulfonamides and *N*,*O*-protected hydroxylamines was investigated by Dohi and Kita (Scheme 16).^253^ The use of iodonium salts with TMP as the dummy ligand and acetate as the counteranion was critical for achieving high yields under mild conditions, while reactions with other counteranions, such as OTs, OTf, and OCOCF_3_, were unsuccessful or led to poor yields.

**Scheme 17:**
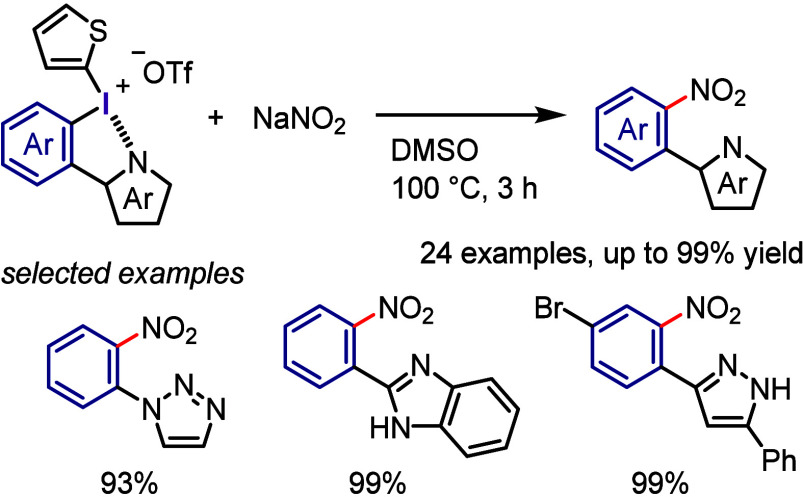
Nitration of Diaryliodonium Salts Bearing *N*-Heterocyclic Aryl Groups

Postnikov et al. examined the influence of *N*-heterocyclic substituents on the reactivity and chemoselectivity of *ortho*-azole-tethered diaryliodonium salts (Scheme 17).^254^ The gram-scale arylation of NaNO_2_ with a range of electronically diverse iodonium salts afforded *ortho*-nitroarene derivatives and could be carried out as a one-pot sequential reaction starting from the related iodoarenes. The azole iodonium salts demonstrated high reactivity and chemoselectivity when reacted with a variety of halogen, nitrogen, oxygen, and sulfur nucleophiles, which was attributed to the stabilization of these salts via coordination with the adjacent azole substituent. However, the reactivity and chemoselectivity of these salts were reduced when the *ortho*-azole group was subjected to *N*-methylation or *N*-protonation.

**Scheme 18:**
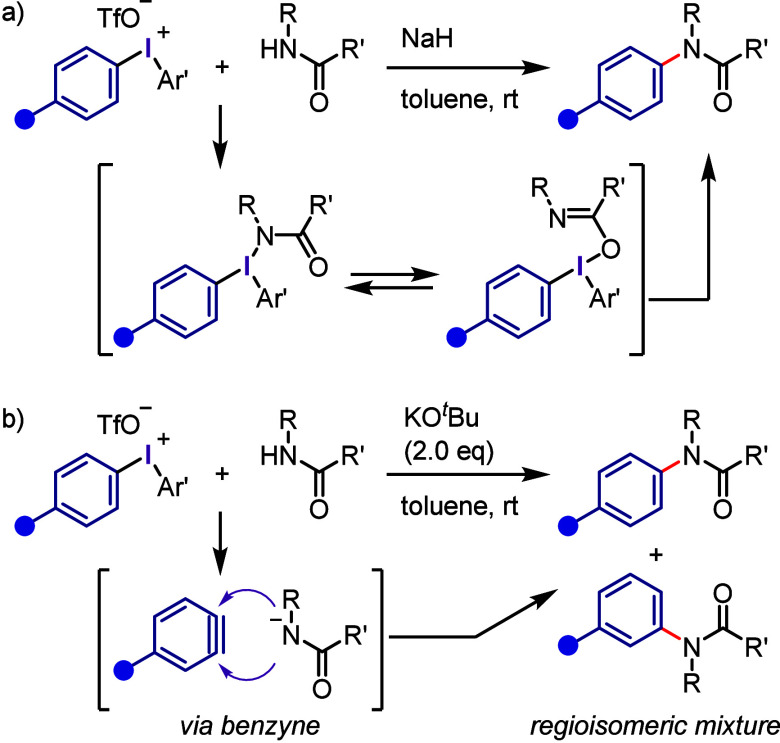
*N*-Arylation of Acyclic Amides with Diaryliodonium Salts

Olofsson et al. used diaryliodonium salts to achieve the *N*-arylation of secondary acyclic amides under mild conditions (Scheme 18a),^242^ suggesting that this reaction proceeds via ligand exchange and the formation of T-shaped intermediates, which undergo ligand coupling through [1,2]- and [2,3]-rearrangements to yield the desired *N*-arylated tertiary amide. Wang et al. prepared *N*-arylated secondary acyclic amides through the in situ generation of aryne intermediates from iodonium salts followed by nucleophilic attack by the deprotonated amide and protonation, which afforded the desired *N*-arylated products (Scheme 18b).^243^ This mechanism was supported by the formation of two regioisomeric *N*-arylated amides.

#### 2.3.4 *N*- *vs O*-Arylation of Ambident Nucleophiles

**Scheme 19:**
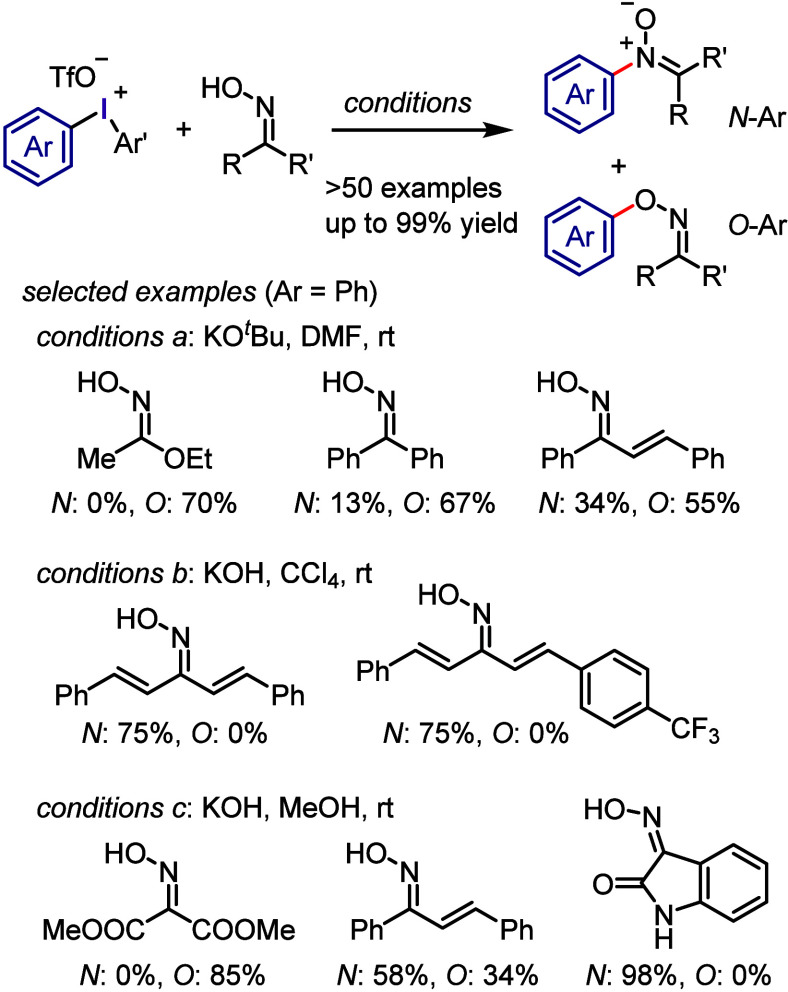
*N*- *vs O*-Arylations of Oximes with Diaryliodonium Salts

The arylation of aldoximes and ketoximes with diphenyliodonium triflates under basic conditions (Cs_2_CO_3_, MeCN, rt, 6 h) resulted in the exclusive formation of *O*-arylated oximes in high yields.^213^ This process was the subject of extensive studies conducted by Mo and colleagues to investigate the influence of the oxime structure and applied conditions for *N*- vs *O*-arylation (Scheme 19).^255,256^ The reaction of diaryliodonium salt with ketoxime in the presence of KO^*t*^Bu (conditions a) generated an *N*-arylation product (nitrone) and *O*-arylation product. The reaction of dibenzylideneacetone oximes with KOH as a base (conditions b) led to the formation of nitrone as the sole product. Similarly, oxindole oxime yielded the *N*-aryl oxindole nitrone as the only product under the optimum conditions.

**Scheme 20:**
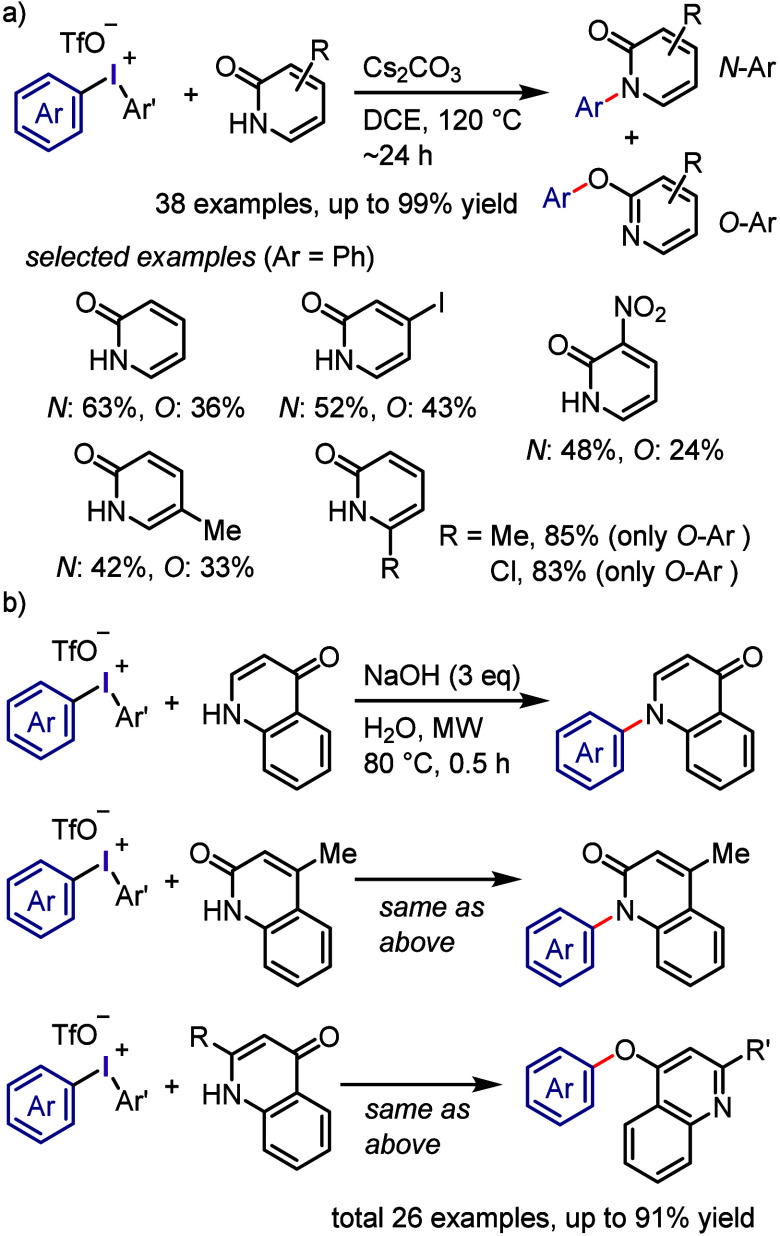
a) Substitutions-Controlled Chemoselective *N*- *vs O*-Arylation of Pyridin-2-ones. b) *N*- *vs O*-Arylation of Quinolones under MW Conditions

The impact of substituents on the 2-pyridone skeleton and the preference for *N*- or *O*-arylation with diaryliodonium salts was thoroughly examined (Scheme 20a).^257^ Under optimal conditions, unsubstituted and C3 to C5 electron-donor/acceptor substituted 2-pyridones displayed a preference for *N*-arylation over *O*-arylation. In contrast, C-6 substituted 2-pyridones exclusively produced *O*-arylated products. It is believed that the *O*-selectivity was due to the steric interaction that occurred during and after the *N*-attack of the 6-substituted-2-pyridone on the I(III) center of the iodonium salt, which was not the case with *O*-attack. Kumar investigated the *N*- *vsO*-arylation of quinolones and related substrates using conventional and microwave heating in aqueous NaOH solvent to produce the same selectivity but with improved productivity and shorter reaction times under microwave heating (Scheme 20b).^258,259^ The arylation of 4-quinolone and 4-methylquinolin-2(1H)-one with microwave heating resulted in the exclusive formation of *N*-arylated products. This *N*-arylation protocol is beneficial for large-scale synthesis and can be applied to structurally relevant substrates like acridin-9-one, quinoxaline-2-one, and benzoimidazol-2-one. Notably, 2-substituted quinoline-4-ones reacted under the same microwave conditions with diaryliodonium triflates produced only *O*-arylation products due to the sterically hindered nitrogen center.

**Scheme 21:**
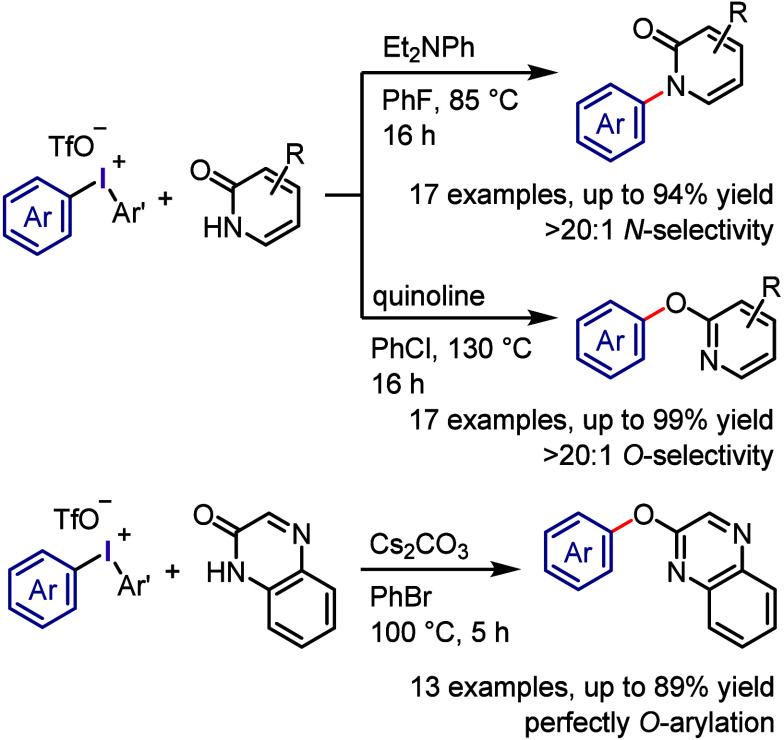
Chemoselective *N*-/*O*-Arylation of Pyridine-2-one and Quinoxaline-2-one

The Onomura and Kuriyama group emphasized the importance of selecting the optimal base for achieving chemoselective *N*- and *O*-arylation of pyridin-2-one using diaryliodonium triflate (Scheme 21).^260,261^ They discovered that *N*,*N*-diethylaniline and quinoline were the most suitable bases for *N*- and *O*-arylation of pyridin-2-one, respectively, affording exceptional chemoselectivity and high productivity. Quinoxalin-2-one also exhibited chemospecific *O*-arylation under various conditions, particularly in the presence of Cs_2_CO_3_ as a base. The diverse substitutions on both pyridin-2-one/quinoxalin-2-one and diaryliodonium salts were found to be well-tolerated. Although the X-ray crystallography structure of the diphenyliodonium salt with the amidate counteranion of 5-trifluoromethylpyridin-2-one was confirmed, the reaction mechanism of this alternative selectivity is still unclear.

**Scheme 22:**
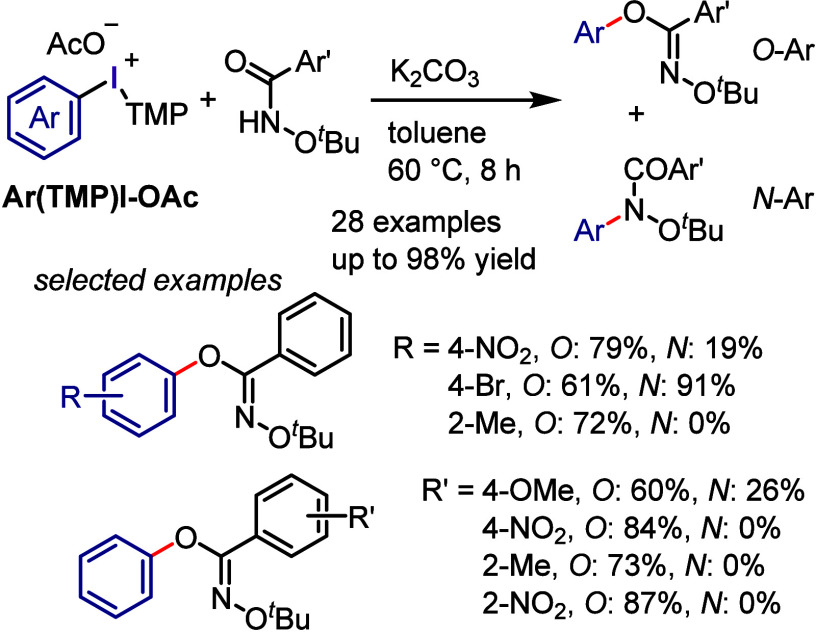
Chemoselective *O*-Arylation of Amides with Iodonium Acetates

Recently, the investigation of the *O*- versus *N*-arylation of *N*-alkoxyamides with diaryliodonium salts has been conducted (Scheme 22).^262^ Among the various *N*-substituted benzamides and iodonium salts, *N*-*tert*-butoxyamide and aryl(TMP)iodonium acetate (**Ar(TMP)I-OAc**) demonstrated the highest reactivity and *O*-arylation selectivity. The reaction conditions were suitable for gram-scale synthesis and could accommodate iodonium acetates and *N*-*tert*-butoxyamides with electron-donating/withdrawing and sterically congested groups. By adjusting the substituents on the starting materials, high-to-excellent *O*-selectivity could be achieved. Remarkably, *N*-methoxy-4-nitrobenzamide yielded only *O*-arylation products when reacted with sterically congested iodonium acetates.

#### 2.3.5 C–C Bond Formation via C–H Arylation

**Scheme 23:**
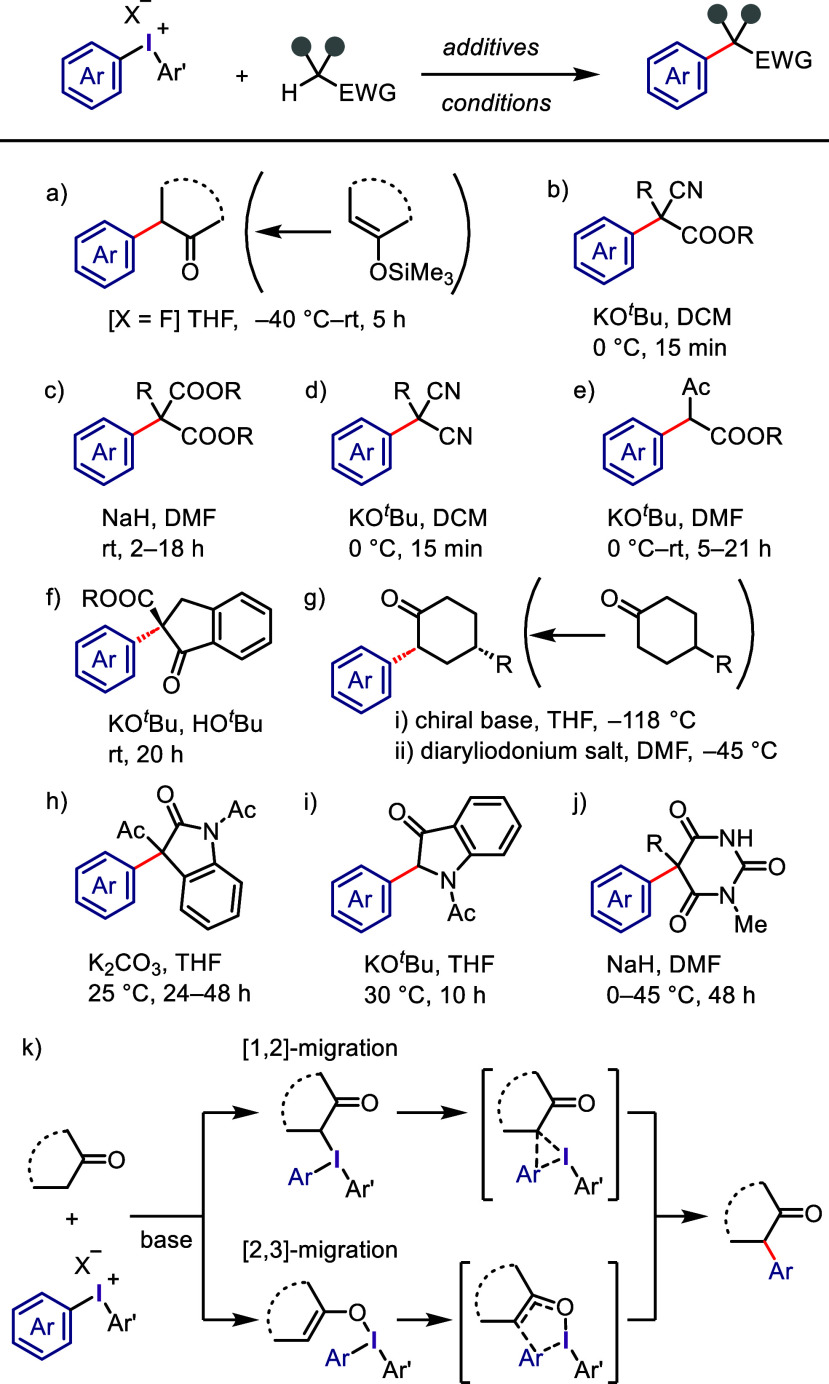
General Conditions for the Csp^3^-Arylation of Enolizable Substrates

Diaryliodonium salts have shown remarkable versatility as transition metal-free arylating reagents, capable of reacting with a range of functional groups, including Csp^3^ and Csp^2^ nucleophiles. Csp^2^–Csp^3^ bond formation has been achieved using silyl enol ethers of aliphatic ketones, which were coupled with diphenyliodonium fluoride to produce mono- or diphenyl ketones (Scheme 23a).^263,264^ Active methylene compounds, such as 2-substituted cyanoacetates,^265^ 2-substituted malonates,^190,266^ 2-substituted malononitriles,^267^ ethyl acetoacetates,^268^ and nitroalkanes,^269^ have been found to be highly effective in arylation reactions with diaryliodonium salts in the presence of KO^*t*^Bu or NaH as bases (Scheme 23b–23e). Shibata and colleagues utilized cyclic β-keto esters/amides as benchmark substrates for pentafluorophenylation, triflyl-(hetero)arylation, and pentafluorosulfanylarylation by related iodonium salts with high chemoselectivity.^270−272^

The study of asymmetric versions of arylation reactions was initially conducted by Ochiai and colleagues, who utilized a chiral iodonium salt ((*S*)-(−)-1,1-binaththyl-2-yl(phenyl)iodonium tetrafluoroborates) for arylation of 2-(alkoxycarbonyl)-1-indanone with moderate enantioselectivity (up to 53% ee) (Scheme 23f).^152^ Aggarwal and Olofsson employed Simpkins’ (*R*,*R*)-base for the desymmetrization of cyclohexanones, followed by coupling with dipyridyliodonium chloride to produce pyridyl derivatives with high enantioselectivity (up to 94% ee), which were subsequently used in the total synthesis of (−)-epibatidine alkaloid (Scheme 23g).^273^ The scope of Csp^3^-arylation with iodonium salts was extended to heterocycles; *N*-acetyl-3-acyloxy-2-oxindoles underwent C3-arylation with aryl(Mes)iodonium hexafluorophosphates,^274^ whereas *N*-acetyl-3-indolinones afforded a C2-arylated product in moderate yield (Scheme 23h and i).^275^ However, aryl(Mes)iodonium triflates showed that the Mes-group transferred more preferentially than the aryl group, resulting in a mixture of C2-aryl and C2-mesity products in moderate yields. C5-Arylation of 1-methyl-5-alkylbarbiturtaes was also achieved (Scheme 23j) and then applied to the synthesis of the general anesthetic mephobarbital drug.^276^ Other heterocycles, such as azlactones and 4-substituted pyrazolin-5-ones, were potently arylated by iodonium salts,^277,278^ wherein aryl(Mes)iodonium salts exhibited poor and excellent aryl-transfer selectivity with azlactones and pyrazolin-5-ones, respectively.

The Olofsson group conducted extensive density functional theory (DFT) studies to propose a plausible mechanism for Csp^3^-arylation using diaryliodonium salts (Scheme 23k).^190,269,279^ This process involves deprotonation of the activated methylene group of the substrate under basic conditions to generate an enolate intermediate, which undergoes ligand exchange with the anion of the iodonium salt to form *C*- and *O*-enolate iodonium intermediates, respectively. The reductive eliminations via [1,2]- and [2,3]-rearrangements through three- and five-membered transition states, respectively, result in the formation of the desired α-arylated product and the extrusion of aryl iodide. DFT calculations demonstrated that both pathways were viable for the formation of the product, and the [2,3]-rearrangement process was found to be more energetically favorable than the [1,2]-rearrangement.

**Scheme 24:**
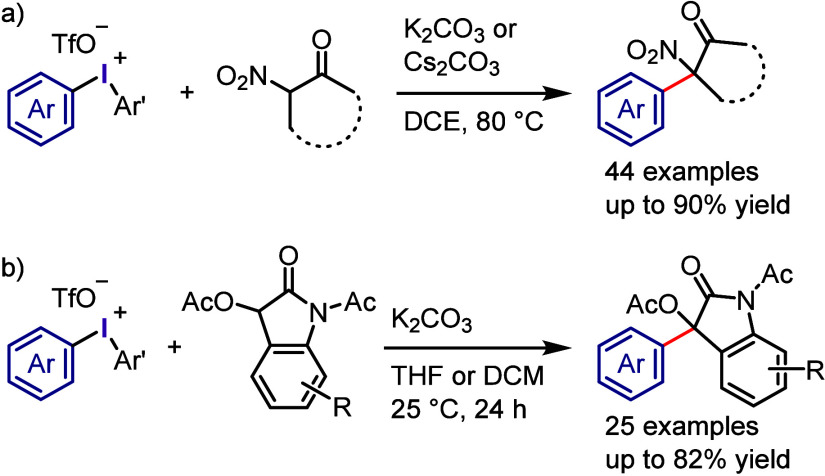
a) α-Arylation of α-Nitroketones with Iodonium Salts. b) C-3 Arylation of Oxindoles with Diaryliodonium Salts

The research conducted by Zhang and colleagues extended the range of *C*-arylation by employing 2-nitoketones for the synthesis of tertiary 2-aryl-2-nitoketones (Scheme 24a).^280^ This reaction was compatible with a range of 5-, 6-, 7-, and 12-membered cyclic 2-nitroketones, benzocyclic nitroketones, and acyclic aryl/alkyl nitroketones. Wang and Li proposed a mild approach for the C-3 arylation of 3-acetyloxy-2-oxindoles using diaryliodonium salts, resulting in the formation of 3-aryl-3-acetyloxy-2-oxindoles (Scheme 24b).^274^

**Scheme 25:**
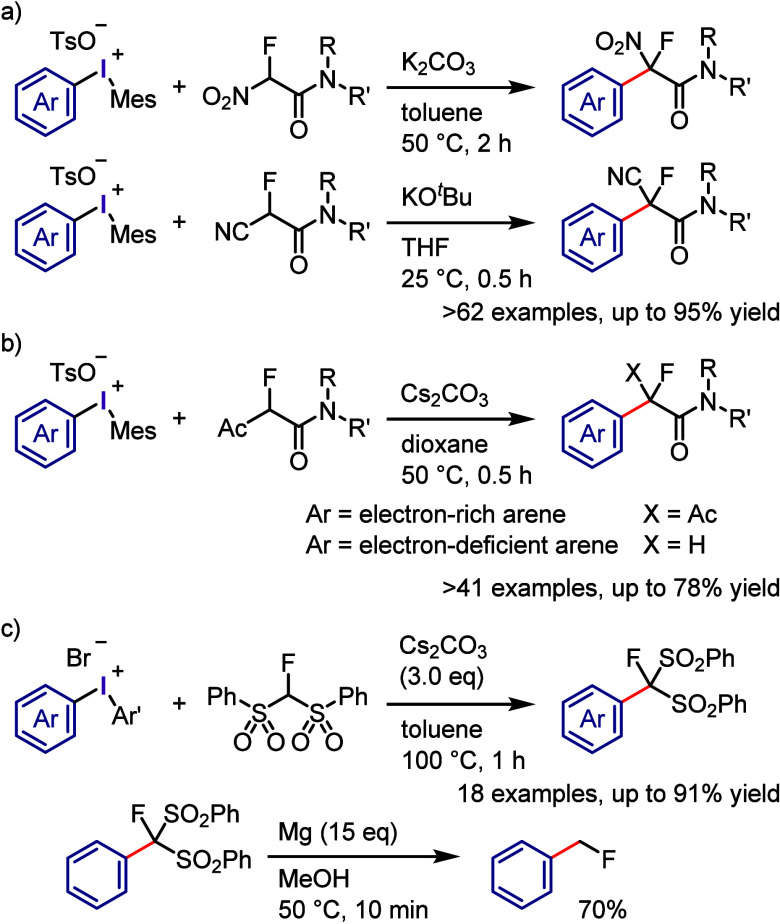
*C*-Arylation of Fluoroacetamides with Diaryliodonium Salts

The application of activated C–H substrates as *C*-nucleophiles in coupling reactions with iodonium salts under ambient conditions was investigated with the aim of forming Csp^3^–Csp^2^ bonds.^281−283^ The compounds, including tertiary amides of α-fluoro-α-nitroacetamides and α-fluoro-α-cyanoacetamides, were found to be effective in forming α-arylated products through α-arylation with electronically and sterically diverse aryl/heteroaryl groups derived from diaryliodonium tosylates with a mesityl dummy ligand (Scheme 25a).^281^ Although secondary amides of these fluorinated acetamides showed moderate reactivity, the reaction scope of α-substituted-α-fluoroacetamides was expanded to include secondary amides of α-fluoroacetoacetamides, which effectively coupled with electron-rich aryl groups to produce fully substituted benzylic products (Scheme 25b).^283^ However, a reaction with electron-deficient iodonium aryl groups resulted in the formation of α-arylated products containing an electrophilic α-acetyl group, which is prone to spontaneous deacetylation under basic conditions, leading to the formation of the related α-aryl-α-fluoroacetamide. Regrettably, the tertiary amide of α-fluoroacetoacetamide was not reactive under these conditions. Prakash et al. utilized fluorobis(phenylsulfonyl)methane, which comprised Csp^3^-nucleophiles with fluoro groups, for coupling with diaryliodonium salts (Scheme 25c).^284^ This led to the synthesis of fluorobis(phenylsulfonyl)methylarenes, which served as useful scaffolds for further reduction steps and the synthesis of biologically attractive Ar-CH_2_F and Ar-CD_2_F derivatives.

**Scheme 26:**
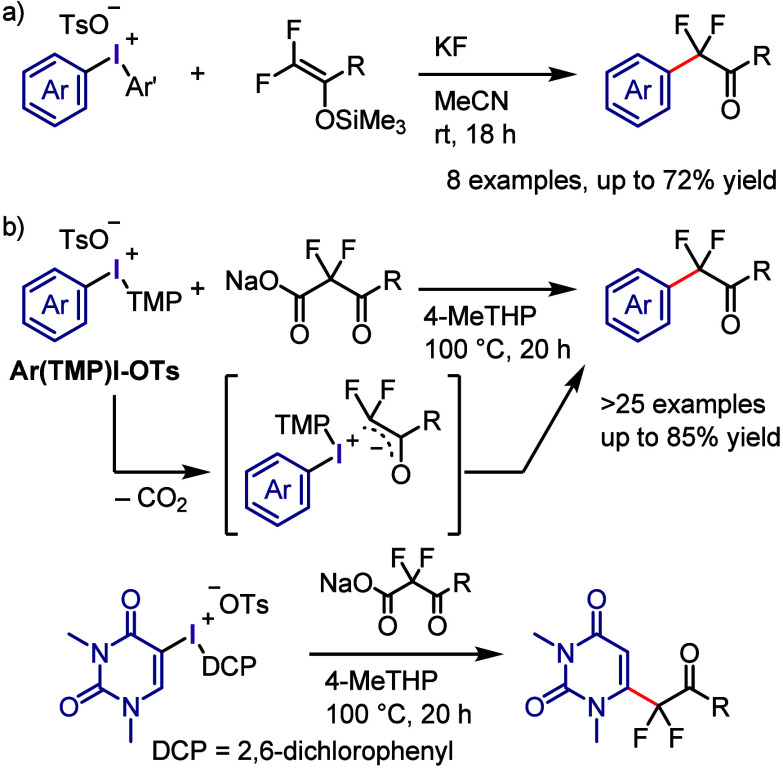
α-Arylation of α,α-Difluoromethyl Ketone

The difluoro enol silyl ethers, which were prepared by reacting trifluoromethylketones with TMSCl/Mg, have proven to be useful nucleophilic synthons for merging α,α-difluorocarbonyl moieties with a variety of electrophiles (Scheme 26a).^285^ Diaryliodonium salts effectively served as precursors for arylating a small library of difluoro enol ethers, resulting in the formation of corresponding α-arylated products.

The Dohi and Kita group first developed a transition metal-free method for the decarboxylative coupling of aryl(TMP)iodonium tosylate (Ar(TMP)I-OTs) with α,α-difluoro-β-ketoacid sodium salt, which afforded useful fluorine-containing scaffolds without requiring hazardous reagents (Scheme 26b).^286^ Mechanistic studies concluded that decarboxylation would occur to generate iodonium difluoroenolate salt. Further ligand coupling produced the aryldifluoromethyl ketone. Interestingly, the reaction of uracil iodonium salt resulted in C-6 difluoromethylation by cine substitution rather than the anticipated C-5 product.

**Scheme 27:**
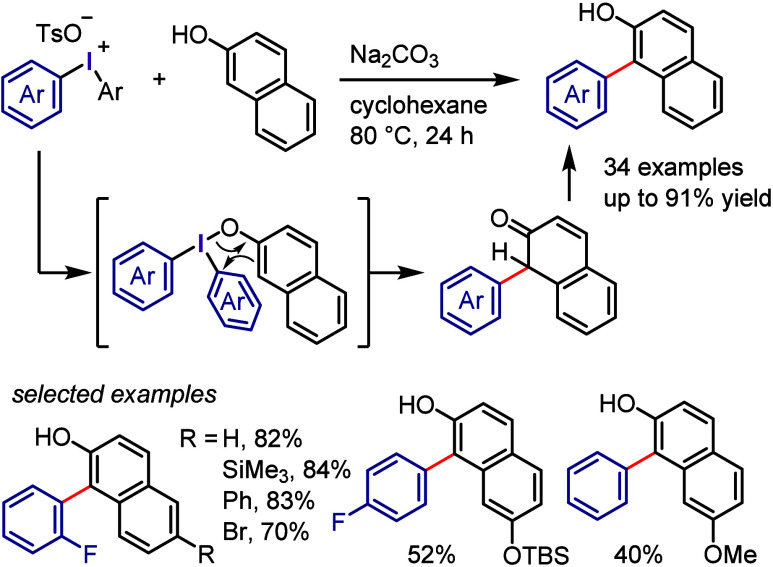
C2-Arylation of 2-Naphthols with Diaryliodonium Salts

Yorimitsu and his colleagues have disclosed a method for generating 2-hydroxy-2′-iodobiaryls through dehydrogenative coupling of phenols with electron-rich diacetoxyiodoarenes.^287^ The Kalek group reported a complementary process for coupling 2-naphthols with diaryliodonium salts, resulting in the formation of related biaryls (Scheme 27).^288^ By using cyclohexane and an inorganic base, the deprotonation of 2-naphthol was avoided, leading to the selective formation of C1-arylated 2-naphthols rather than *O*-arylation, and O-/C1-double arylation products as previously obtained by Quideau.^289^ Efficient coupling with substituted 2-naphthols required diaryliodonium salts with electron-deficient aryl groups. A plausible mechanism was proposed based on experimental and DFT studies, suggesting that the association of 2-naphthol with diaryliodonium salts formed diaryliodonium naphthoxide. C–C bond formation through rearrangement produced the dearomatized ketone, which spontaneously rearomatized via tautomerization to give C1-arylated naphthol. The other possible *O*-arylation product was found to be energetically unfavorable in the presence of protonated 2-naphthol.

**Scheme 28:**
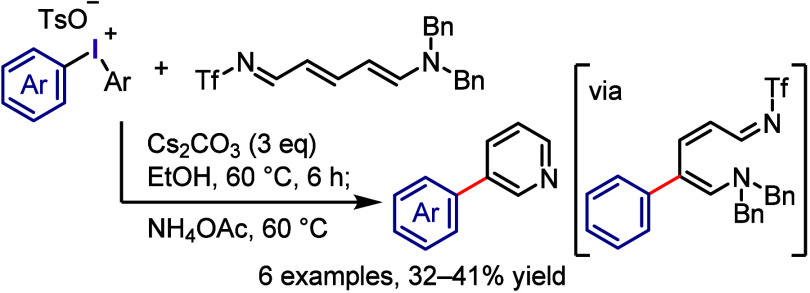
Regioselective Arylation of Pyridine with Diaryliodonium Salts

Treating pyridine with Tf_2_O and a secondary amine afforded azahexatrienes (Zincke imines), which were used by Greaney’s group as C-nucleophiles for the regiodivergent arylation of pyridines under transition metal-free and -catalyzed conditions.^290^ When symmetrical diaryliodonium salts were used as arylating reagents for coupling with a Zincke imine under transition metal-free conditions followed by cyclization, the related *meta*-arylated pyridines were obtained exclusively (Scheme 28).

#### 2.3.6 C–S Bond Formation

**Scheme 29:**
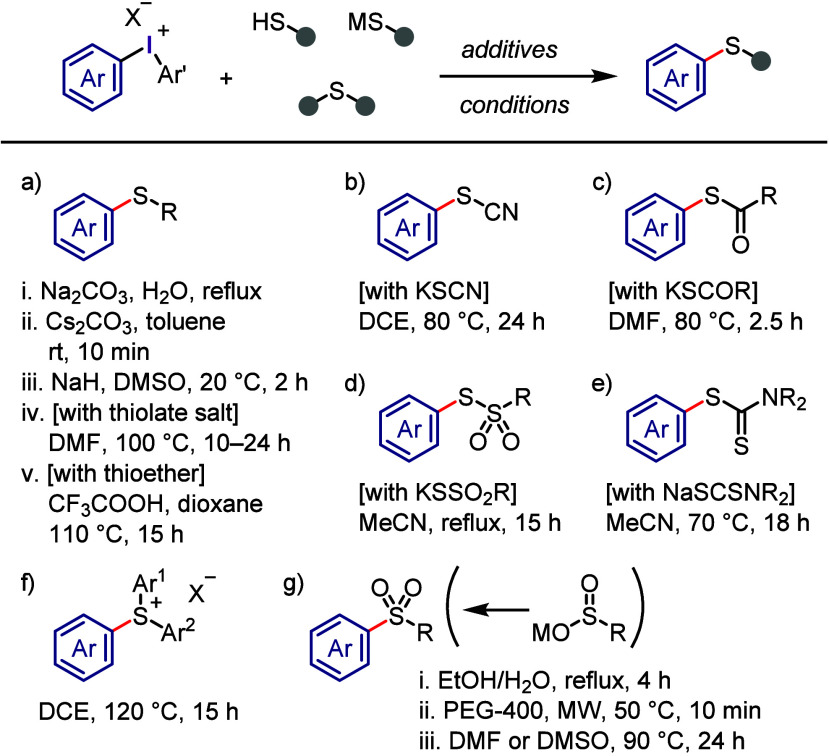
General Conditions for *S*-Arylations with Diaryliodonium Salts

Diaryliodonium salts have proven to be valuable reagents for the arylation of various sulfur-containing substrates, affording C–S bonds. Sandin and Beringer conducted the *S*-arylation of thioglycolic acid, thiophenol, cysteine, and sulfinate salt under aqueous conditions (Scheme 29a-i and g-i).^104,291^ The combination of Cs_2_CO_3_ and toluene promoted the completion of the reaction within 10 min (Scheme 29a-ii).^292^ Alkylthiol was converted in situ to the sodium salt, then reacted with diphenyliodonium triflates to give hexyl phenyl sulfide (Scheme 29a-iii).^293^ Polymer-bound diaryliodonium salts were used to S-arylate sodium benzenethiolate, and the resulting polymer-bound aryl iodide could be recycled (Scheme 29a-iv).^294^ Sanford reported a different approach for synthesizing aryl sulfides by reacting alkyl/aryl thiols and thioethers with diaryliodonium trifluoroacetates under acidic conditions (Scheme 29a-v).^295^ KSCN combined with diaryliodonium triflates led to the formation of aryl thiocyanates (Scheme 29b).^296,297^ Potassium and sodium derivatives of thiocarboxylic, thiosulfonic, and dithiocarbamic acid underwent S-arylation with diaryliodonium salts in moderate to satisfactory yields (Scheme 29c–e).^298−300^ Ciufolini and colleagues developed a copper-free method for synthesizing triarylsulfonium triflates by reacting diaryl sulfides with iodonium triflates (Scheme 29f).^301^ The reaction was effective, and the iodonium salts with two different aryl groups generated a mixture of arylation products with a preference for transferring electron-rich aryl groups to electron-poor and thienyl groups. The Dohi group investigated the use of diaryliodonium triflates containing a mesityl group as a dummy ligand for fully chemoselective S-arylation of diaryl sulfides and the formation of triarylsulfonium triflates under both copper-free and copper-catalyzed conditions.^302^ A study conducted by Kumar and Manolikakes successfully synthesized diaryl and aryl-heteroaryl sulfones by reacting (hetero)arylsulfinic acid sodium salts with diaryliodonium salts under thermal and microwave conditions (Scheme 29g-ii and -iii).^303−306^ Xu and co-workers employed readily available sodium glycosyl sulfinates for the glycosyl sulfonation of diaryliodonium salts and the synthesis of glycosyl aryl sulfones in the presence of DMSO or H_2_O as solvent.^307^

**Scheme 30:**
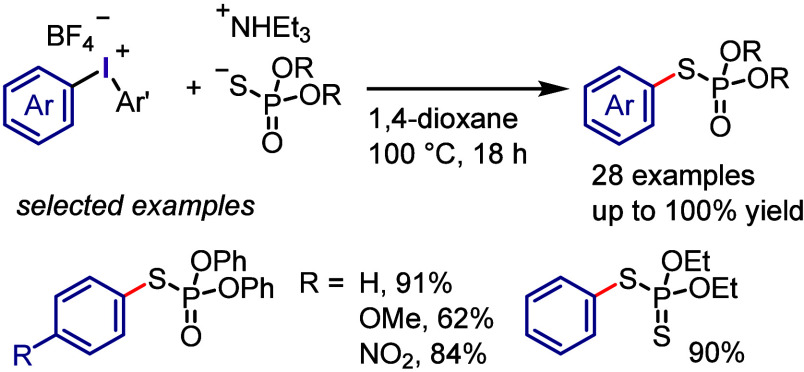
*S*-Arylation of Phosphorothioate Diesters with Iodonium Salts

The Kalek group utilized diaryliodonium salts for the *S*-arylation of phosphorothioate diesters, preserving the stereochemistry of the phosphorus atom (Scheme 30).^308^ A variety of *O*,*O*-diaryl and *O*,*O*-dialkyl phosphorothioate diesters reacted efficiently under the specified reaction conditions. Phosphorus substrates such as phosphorodithioates and phosphonothioates were also found to be effective to yield *S*-arylation products.

**Scheme 31:**
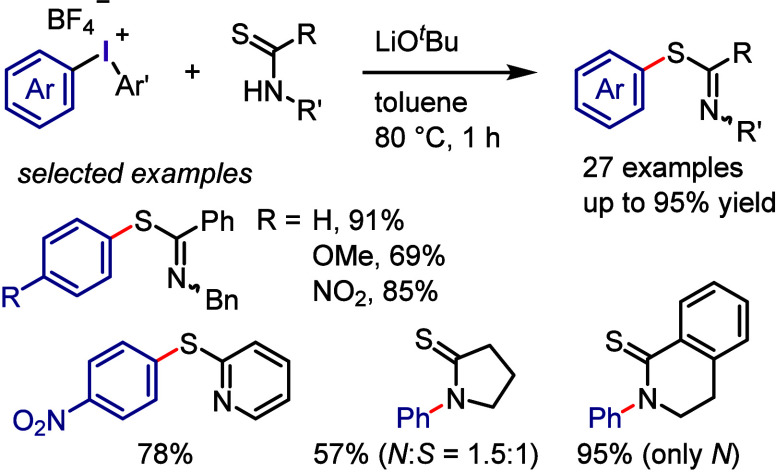
Arylation of Thioamides with Diaryliodonium Salts

A process for the *S*-arylation of thioamides under basic conditions was established by Olofsson and colleagues (Scheme 31).^309^ Aromatic thioamides and pyridine-2-thiol displayed excellent S-site selectivity during the reaction. While aliphatic thioamides and pyrrolidine-2-thione produced mixtures of *S*- and *N*-arylations, 3,4-dihydroisoquinoline-1(2*H*)-thione only yielded an *N*-arylation product. This selectivity was attributed to the conjugation within the thioamide group, where efficient conjugation in aromatic thioamides led to increased S-nucleophilicity. In contrast, less efficient conjugation, along with the constraints in cyclic thioamides, resulted in more *N*-nucleophilicity. Mechanistic studies ruled out the radical mechanism and the generation of aryne intermediates.

**Scheme 32:**
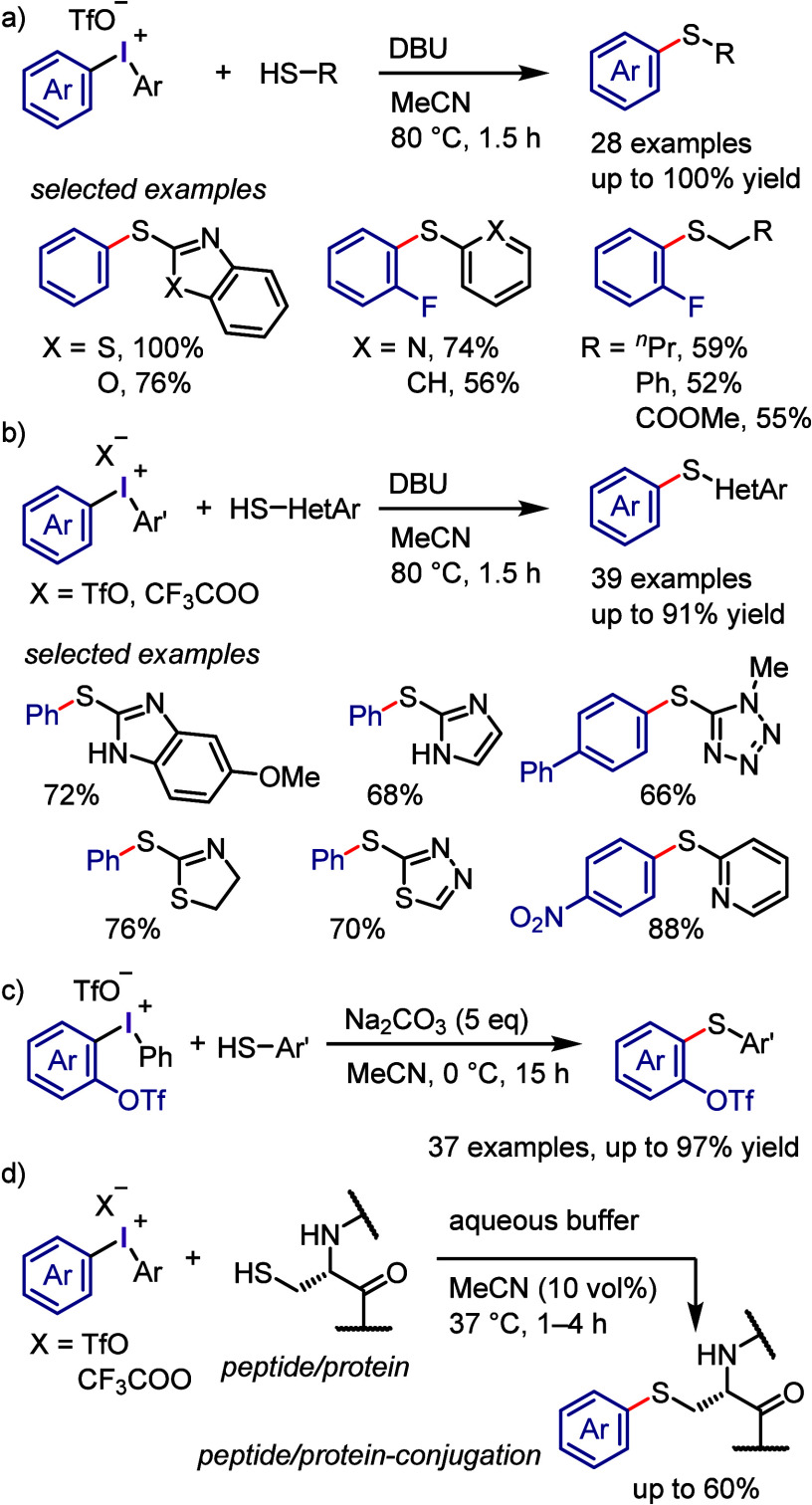
*S*-Arylation of Thiols Using Diaryliodonium Salts

Apart from the methods previously reported for arylation of thiols using diaryliodonium salts,^292,295,310^ Kalek et al. have developed the arylation of heterocyclic thiols under mild conditions (Scheme 32a).^311^ This S-arylation with a specific base demonstrated success with a wide range of 5-/6-membered heterocyclic thiols, aliphatic thiols, and diverse mercaptobenzazoles. A more effective protocol for the *S*-arylation of heterocyclic thiols using diaryliodonium salts was reported by the Thakur group (Scheme 32b).^312^ Notably, no interference from *N*-arylation was observed in the screened thiol substrates, even with acidic N–H groups, and the related *S*-arylation products were exclusively formed.

Wang, Han, and collaborators demonstrated that the reaction of *ortho*-OTf-substituted diaryliodonium salts with thiophenols afforded the corresponding diaryl sulfides (Scheme 32c), whereas this type of iodonium salts underwent intramolecular aryl migration in the absence of thiols.^313^ The obtained *ortho*-OTf-substituted diaryl sulfides were employed for further coupling reactions, wherein the OTf group served as the reaction point.

The early work of Sandin et al.^291^ on the *S*-arylation of Cys-amino acids with diphenyliodonium chloride prompted the Payne group to explore the chemoselective arylation of peptides and proteins at the Cys-position using more functionalized diaryliodonium salts (Scheme 32d).^314^ To accomplish the late-stage functionalization of MUC1 VNTR peptide, affibody zEGFR, and histone H2A proteins, alkyne-, keto-, and mPEG-derived diaryliodonium salts were synthesized.

**Scheme 33:**
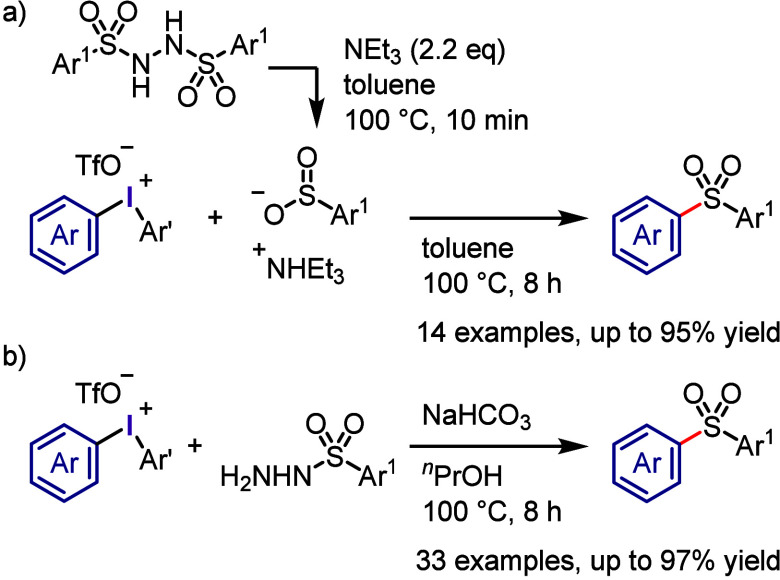
Aryl Sulfonyl Anion Equivalents for Coupling with Diaryliodonium Salts

In addition to aryl sulfinate sodium salt,^304^*N*,*N*′-disulfonylhydrazine has been used as an effective aryl sulfonyl anion precursor due to its stability and solubility in common solvents.^315^ In sequential one-pot reactions, disulfonylhydrazine with triethylamine generated ammonium sulfinate, which then reacted with added diaryliodonium salt to produce the related diaryl sulfone (Scheme 33a). This reaction was compatible with various *N*,*N*′-disulfonylhydrazines. Alternatively, treating sulfonyl hydrazides with base produced sulfonyl anions that reacted with diaryliodonium salts to similarly yield the corresponding diaryl sulfones (Scheme 33b).^316^

**Scheme 34:**
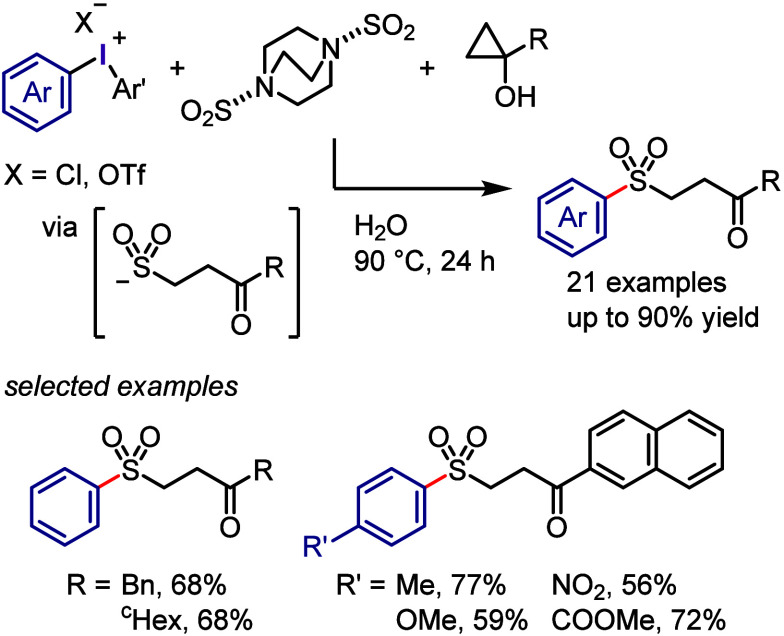
3-Component Reaction of Diaryliodonium Salt with DABSO and Cyclopropanol in Water

Ye, Wu, Chen, and co-workers developed a direct approach for the synthesis of γ-ketosulfones via a 3-component reaction of cyclopropanol, SO_2_-surrogate (DABSO), and diaryliodonium salt in H_2_O as the only solvent under additive-, catalyst-, and oxidant-free conditions (Scheme 34).^317^ Mechanistic investigations indicated the in situ generation of γ-ketosulfinate intermediate, which underwent ligand coupling with diaryliodonium salt to give substituted γ-keto sulfones. Preliminary antitumor activity of these aryl-substituted γ-ketosulfones showed potent inhibition of SBC-2 and 16HBE cell lines.

**Scheme 35:**
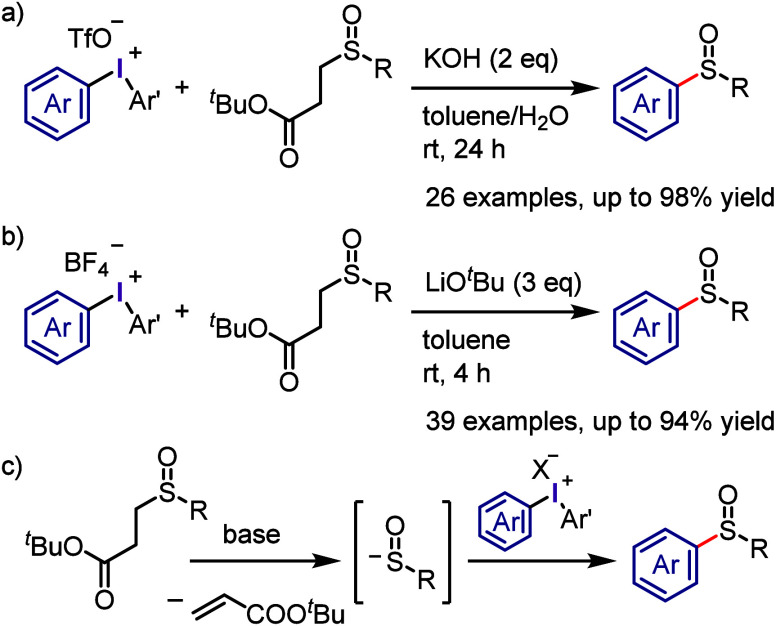
Synthesis of Sulfoxides through Generation of Sulfinate Anion and Coupling with Iodonium Salts

The Bolm group has introduced a new method for synthesizing sulfoxides, which involves the in situ generation of sulfinate anions from β-sulfinyl esters under basic conditions and the subsequent reaction with diaryliodonium triflates (Scheme 35a).^318^ A broad range of *S*-(hetero)aryl-β-sulfinyl esters were successfully reacted to produce diaryl sulfoxides. In a related study, Zhang and colleagues reported a method for synthesizing *S*,*S*-disubstituted sulfoxides using diaryliodonium tetrafluoroborate, which produced the desired product in high yield (Scheme 35b).^319,320^ The proposed mechanism for the reaction commenced with a retro-Michael reaction of the β-sulfinyl ester to generate a sulfinate anion, which then underwent ligand exchange followed by ligand coupling to form the sulfoxide product (Scheme 35c).

**Scheme 36:**
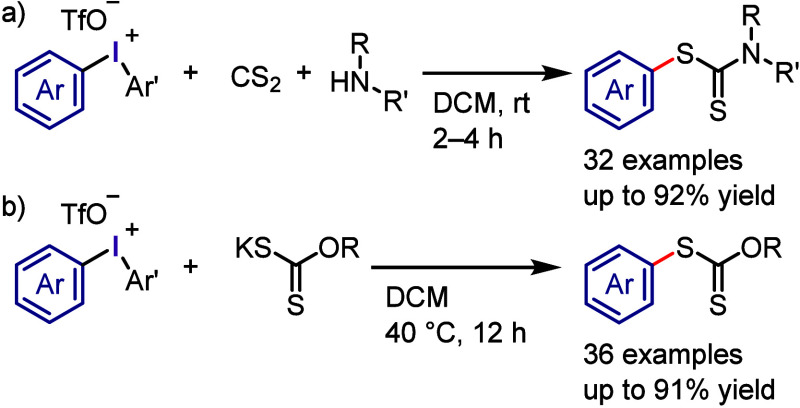
(a) One-Pot 3-Component Synthesis of *S*-Aryl Dithiocarbamates. (b) Coupling of Iodonium Salt with Xanthates

The reaction of diaryliodonium salts with dithiocarbamic acid sodium salt provided the corresponding *S*-aryl dithiocarbamates.^300^ A multicomponent protocol, developed by Murarka et al., was a convenient process for the synthesis of *S*-aryl dithiocarbamate (Scheme 36a).^321^ This new method involved a cascade reaction of iodonium salts with cyclic/acyclic aliphatic amines and carbon disulfide under additive-free conditions, leading to the production of a diverse range of *S*-aryl dithiocarbamates. The synthesis of aryl thiols using diaryliodonium salts is a challenging process, which Karchava and colleagues addressed by developing a new approach for synthesizing aryl thiol surrogates through the reaction of iodonium salts with potassium *O*-alkylxanthates (Scheme 36b).^322^ This approach utilized mild conditions that prevented further functionalization and over-reaction of the resulting *S*-aryl *O*-alkylxanthate products. Based on these results, the same group developed a new thiol-free process for the preparation of alkyl aryl thioethers from the corresponding *S*-aryl *O*-alkylxanthates.^323^

**Scheme 37:**
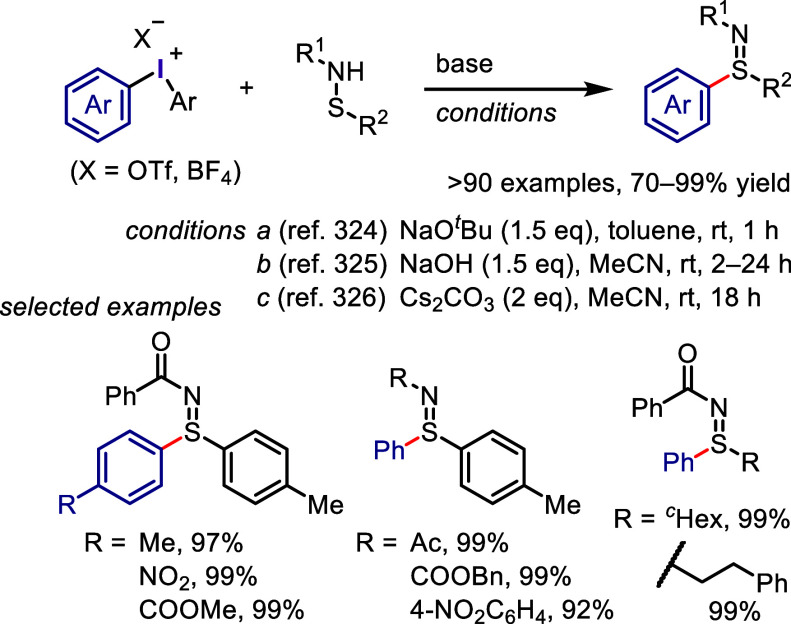
S-Arylations of Sulfenamides with Diaryliodonium Salts

Lu, Yang, and Wu reported independently base-promoted S-arylations of sulfenamides with diaryliodonium salts and the synthesis of the related sulfilimines with excellent chemoselectivity (Scheme 37).^324−326^ The reaction conditions tolerated sulfenamides with *N*-aryl/alkyl acyl groups and *S*-alkyl/aryl substituents.

#### 2.3.7 Miscellaneous Bond Formations

**Scheme 38:**
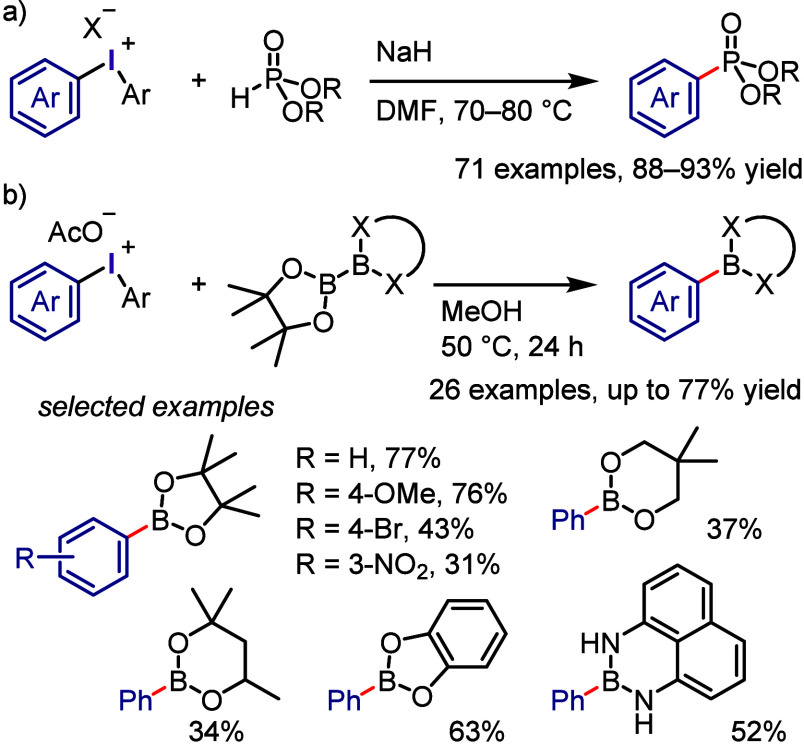
Ar–P and Ar–B Bonds Formations with Diaryliodonium Salts

The versatility of diaryliodonium salts extends beyond their ability to form C–C, C–N, and C–S bonds as arylating reagents. They also facilitate the formation of other chemical bonds, such as C–P bonds. The reaction of diaryliodonium salts with phosphite anions resulted in the synthesis of arylphosphonates in high yields (Scheme 38a).^327^ Electron-rich and electron-deficient diaryliodonium salts were used for covalent functionalization of few-layer black phosphorus, offering excellent ambient stability and the potential to tune electronic properties.^328^

The formation of C–B bonds was the focus of research conducted by Muñiz and colleagues, who developed a useful methodology for the reaction of diaryliodonium acetates with diboron reagents in methanol as a solvent under base-free conditions (Scheme 38b).^329^ The acetate counteranion and methanol solvent played a crucial role, interacting as Lewis bases with the diboron reagent and umpolunging the electrophilic character. This resulted in the generation of a nucleophilic boron center capable of reacting with diaryliodonium salts and forming aryl boronic esters. Arylation of various diboron substrates was also demonstrated.

**Scheme 39:**
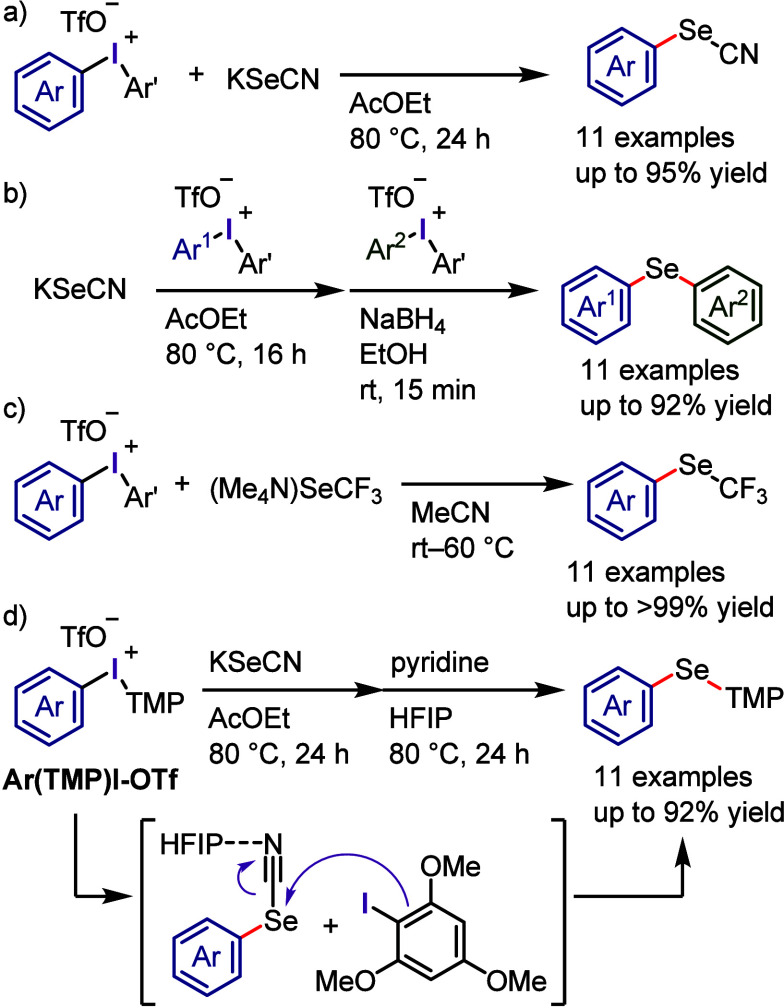
Arylation of KSeCN and (Me_4_N)SeCF_3_ by Diaryliodonium Salts

Chalcogens with properties similar to sulfur, such as selenium and tellurium, have been observed to perform the same type of reactions with similar reactivity.^301,330−336^ Townsend and colleagues demonstrated the reactivity of potassium selenocyanate (KSeCN) with diaryliodonium salts to produce corresponding aryl selenocyanates (Scheme 39a).^337^ Upon treatment of the resulting products with sodium borohydride, selenium anions were generated, which are reactive toward various iodonium salts and glycosyl halide electrophiles. The one-pot and sequential reaction of KSeCN with two different diaryliodonium salts resulted in the formation of unsymmetrical diaryselenides in high overall yields (Scheme 39b). The Zhang group utilized (Me_4_N)SeCF_3_, a readily available nucleophile, to generate aryl trifluoromethylselenoethers (Scheme 39c).^338^ Soldatova, Postnikov, and collaborators designed a one-pot strategy for double arylation of KSeCN with the two aryl ligands of the aryl(TMP)iodonium triflate (**Ar(TMP)I-OTf**) (Scheme 39d).^339^ The arylation of KSeCN nucleophile generates ArSeCN and TMP-I as an electrophile and electron-rich arene intermediate, which reacted together in the presence of HFIP as a crucial solvent^340^ to yield the desired diarylselenide.

The formation of C–halogen bonds can be achieved through the thermolysis of an iodonium salt with a halide counteranion, either preformed or prepared in situ. This process involves a *pseudo* S_N_2-type mechanism, where the halide anion is transferred to the equatorial aryl group of the iodonium salt, resulting in the production of the corresponding aryl halide.^341−345^ This same process can be used to synthesize radiolabeled haloarenes,^346−349^ including radiofluorination of iodonium salts. This process has been extensively studied and practical application in positron emission tomography (PET) was found after the pioneering work of Pike and colleagues.^350−361^ The use of diaryliodonium salts and aryliodonium ylides as arylating reagents provides a useful expansion for the radio-functionalization of a broad library of electron-rich and electron-deficient (hetero)aryl moieties, which are not accessible through traditional methods. Indeed, the nucleophilic radiofluorination process is a rapid and effective method for synthesizing various ^18^F-labeled compounds and radiotracer molecules for biomolecular imaging with PET. As this topic has been summarized in many reviews before,^356−361^ we will focus on recent examples of radio-functionalization of iodonium salts/ylides.

**Scheme 40:**
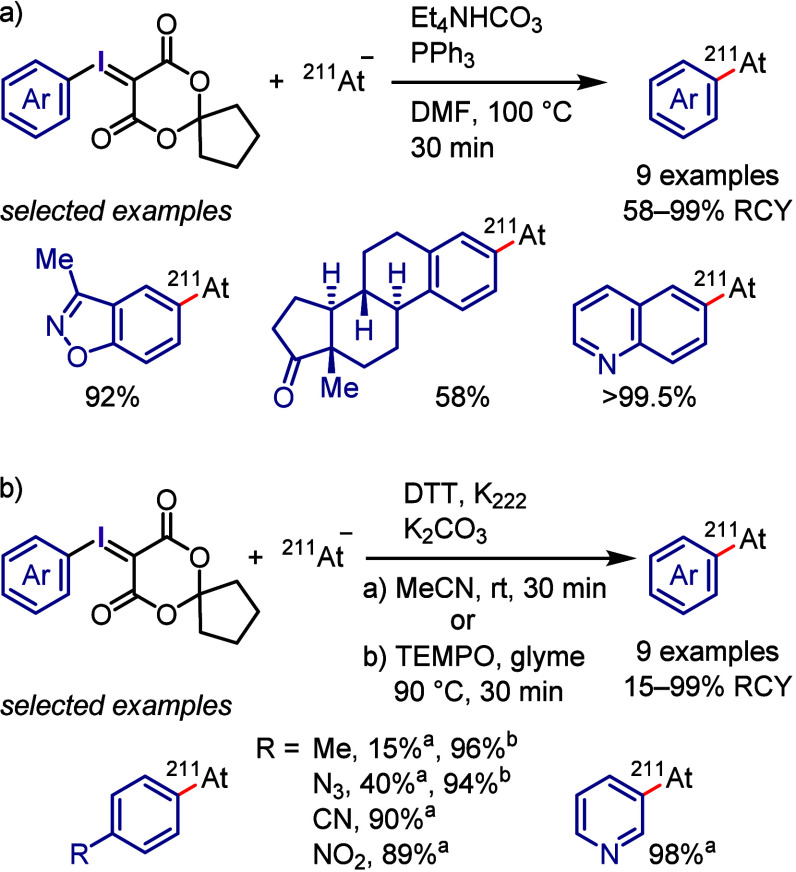
Synthesis of ^211^At-Labeled Compounds Using Aryliodonium Ylides DDT = dithiothreitol, K222 = kryptofix, and TEMPO = 2,2,6,6-tetramethylpiperidin-1-yl)oxyl.

The Matsunaga group achieved the successful synthesis of astatine-211 radiolabeled multifunctionalized molecules by employing a formal S_N_Ar reaction between an aryliodonium ylide and astatide nucleophile (^211^At^–^) under reducing conditions (Scheme 40a).^362^ This radiolabeling process was applicable to the functionalization of various substrates, including estrone natural product, fibrate drug, phenylalanine amino acid, and other (hetero)arenes with ^211^At. The nucleophilic radioastatination process was expanded by Guérard et al., who screened various aryliodonium ylides under different conditions (Scheme 40b).^363^ Aryliodonium ylides with electron-withdrawing substituents performed the reaction smoothly at room temperature (conditions a), while electron-rich aryliodonium ylides required modified conditions (conditions b). Under conditions b, the presence of the radical scavenger TEMPO was crucial to avoid degradation of the iodonium ylide and achieve a high radiochemical yield and molar reactivity. Aryliodonium ylides serve as excellent reagents for arylating ^125^I^–^ and ^211^At^–^, which resolved the previously identified aryl selectivity issue with aryl(PMP)iodonium salts.^364^

**Scheme 41:**
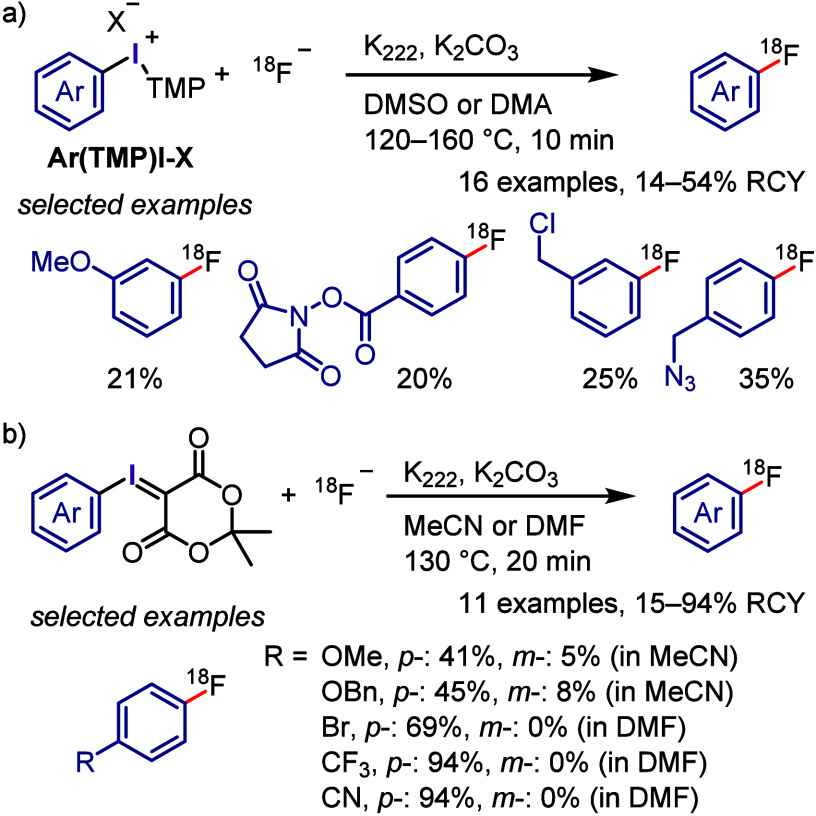
Radiofluorination of Aryliodonium Precursors

Aryl(TMP)iodonium salts (**Ar(TMP)I-X**) played a pivotal role in achieving superior aryl transfer selectivity and radiochemical yields (RCY) during the radiosynthesis of [^18^F]fluoroarenes (Scheme 41a).^365^ The utilization of these salts facilitated the synthesis of a broad range of electron-rich/deficient [^18^F]fluoro(hetero)arenes, as well as [^18^F]fluoroarenes with additional functional groups, which are valuable for constructing potential PET radiopharmaceuticals. Pike et al. evaluated ^18^F-fluorination of aryliodonium ylides under the normal radiofluorination conditions (Scheme 41b).^366^ 4-substituted aryl iodonium ylides with alkoxy and halogen groups afforded mixtures of regioisomeric ^18^F-labeled products in moderate ylides when MeCN was used as a solvent, whereas the reactions in DMF yielded lower ylides with significant decrease or absence of the undesired 3-^18^F products. These results indicated the generation of aryne intermediate as a competing pathway with the direct *ipso*-nucleophilic mechanism^367,368^ and declared the role of DMF solvent as an aryne quencher.

#### 2.3.8 Synthesis of Heterocycles via Intramolecular Cyclization

**Scheme 42:**
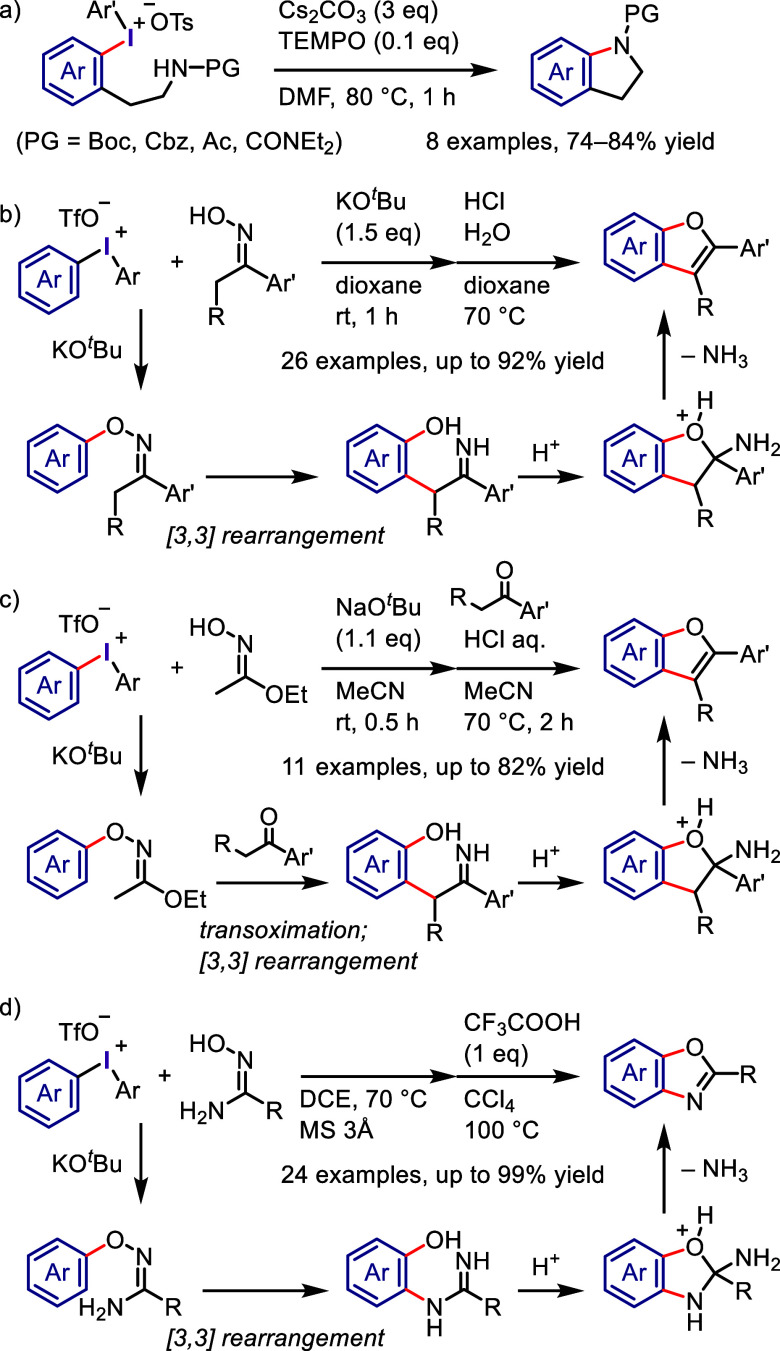
Diaryliodonium Salts for the Synthesis of Indolines, Benzofurans, and Benzoxazoles

Diaryliodonium salts are noteworthy for their ability to form diverse carbocyclic and heterocyclic structures through a range of coupling reactions with nucleophiles. These salts can facilitate arylation via intramolecular cyclization, by directly generating the desired cyclic product or providing an intermediate that can undergo further functionalization and cyclic skeleton formation in a single or multistep process. For instance, Chi and colleagues designed diaryliodonium tosylates that linked the *ortho*-position of one aryl group to a nucleophile, while the other aryl ligand was substituted with electron-withdrawing and donating groups (Scheme 42a).^369^

Intramolecular ligand coupling resulted in the production of *N*-protected indolines in high yield. A critical factor in this process was the inclusion of TEMPO as a radical scavenger for achieving optimal results. Kürti and Olofsson independently synthesized benzo[b]furans by two methods: *O*-arylation of ketoxime using diaryliodonium salts followed by [3,3]-rearrangement and cyclization under acidic conditions, and chemospecific *O*-arylation of ethyl acetohydroxamate using diaryliodonium salts, followed by the addition of ketones and subsequent treatment with aqueous hydrogen chloride. Kürti’s approach involved *O*-arylation of ketoximes with diaryliodonium salts to give *O*-arylketoximes, which were then treated with aqueous hydrogen chloride to produce a diverse range of benzofurans (Scheme 42b).^211^ Togo et al. also reported a similar sequential reaction using one-pot procedure.^212^ Olofsson’s method involved a sequence of hydrolysis/transoximation, [3,3]-rearrangement, and cyclization, ultimately yielding the substituted benzofurans (Scheme 42c).^213^ Mo et al. extended the synthesis of substituted benzoxazoles by using a two-step protocol involving selective *O*-arylation of amidoxime with diaryliodonium salts and treatment with trifluoroacetic acid to achieve [3,3]-rearrangement and cyclization, resulting in the final benzoxazole products (Scheme 42d).^370^

**Scheme 43:**
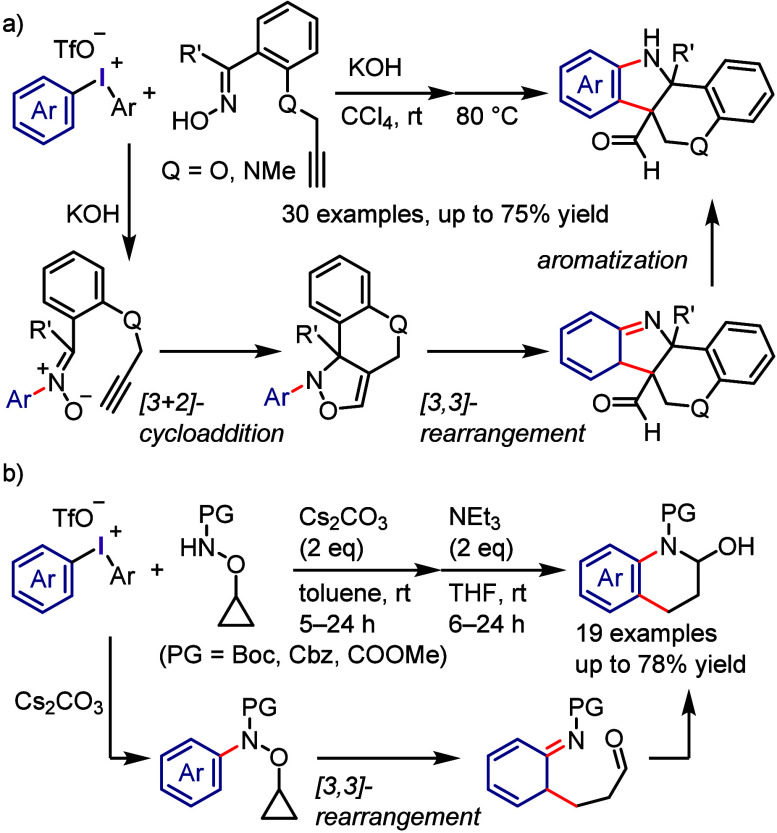
a) Synthesis of Fused Indolines via *N*-Arylation, [3+2] Cycloaddition, and [3,3]-Rearrangement. b) Synthesis of 2-Hydroxytetrahydroquinolines from *O*-Cyclopropyl-hydroxylamine via *N*-Arylation, Rearrangement, Cyclization, and Aromatization

Mo and colleagues have developed a one-pot cascade reaction of alkyne-tethered-oximes, which proved effective in synthesizing 2,3-quaternary fused indolines with high diastereoselectivity. This reaction sequence involved *N*-arylation of oxime, 1,3-dipolar cycloaddition, and [3,3]-rearrangement (Scheme 43a).^371^

The use of an ether-linker (X = O) in the reaction led to the production of only fused indoline products through [3,3]-rearrangement. The Kürti team proposed that *N*-arylation of *O*-cyclopropyl hydroxylamine could be a suitable precursor for [3,3]-sigmatropic rearrangement and the formation of diverse heterocycles (Scheme 43b).^372^ To test this hypothesis, a library of *O*-cyclopropyl hydroxylamines was reacted with diaryliodonium triflates, resulting in the formation of *N*-aryl-*O*-cyclopropyl-hydroxamates. Treating the *N*-arylation products with triethylamine successfully resulted in the formation of 2-hydroxytetrahydroquinolines via a cascade reaction involving [3,3]-rearrangement, cyclization, and rearomatization.

**Scheme 44:**
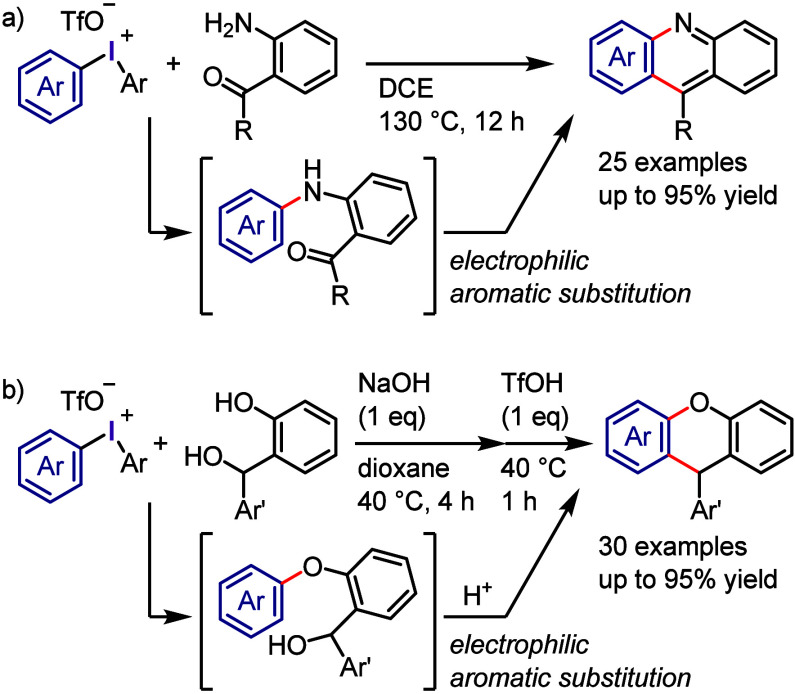
Synthesis of Acridine/Xanthene via Arylation and Friedel-Crafts Cyclization

Acridine derivatives were synthesized through a one-pot reaction of substituted ortho-acylanilines with diaryliodonium triflate, which involved *N*-arylation of the amino group followed by intramolecular Friedel–Crafts cyclization (Scheme 44a).^235^ The same hypothesis was applied to the synthesis of 9-arylxanthenes using a cascade reaction of 2-(aryl(hydroxy)methyl)phenol with diaryliodonium triflates to generate an *O*-arylation product, which subsequently cyclized under acidic conditions to form 9-arylxanthenes (Scheme 44b).^373^

**Scheme 45:**
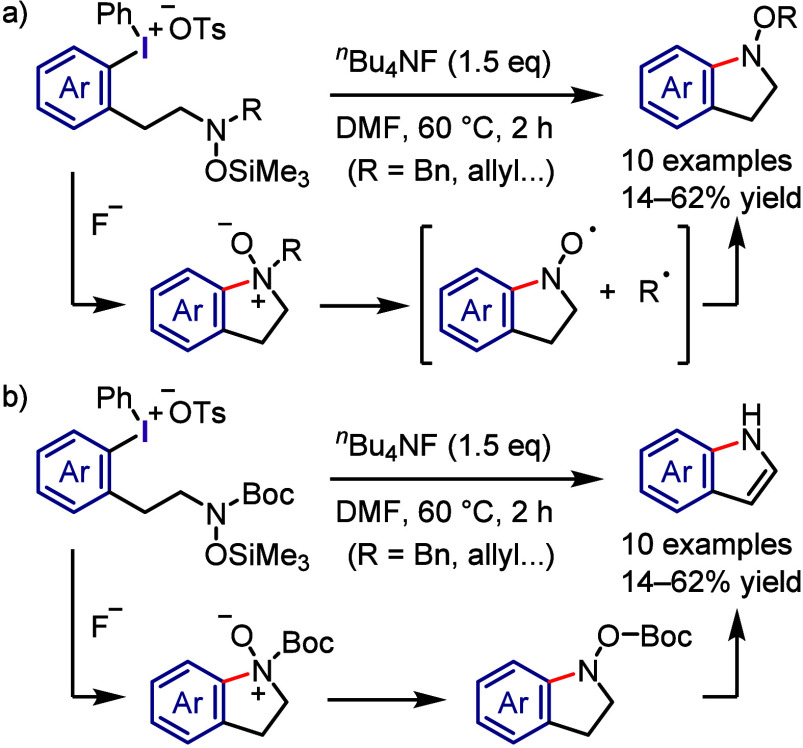
Intramolecular Cyclization of Iodonium Salts into *N*-Alkoxyindolines and Indoles

Ogura and co-workers synthesized a range of substituted indolines and indoles by using *ortho*-*N*-alkyl-*O*-TBS-hydroxylamine tethered diaryliodonium tosylates and treating with TBAF (Scheme 45a).^374^ When an electron-withdrawing N-Boc group was introduced to the substrate, the related functionalized indole was afforded instead (Scheme 45b). The reaction involved the initial C–N bond formation followed by Boc-migration to generate Boc-protected *N*-hydroxy indoline, which underwent extrusion of *tert*-butyl hydrogen carbonate and isomerization to afford the indole product.

### 2.4 Arylation via Generation of Aryl Radical

Aryldiazonium salts are utilized as precursors for aryl radicals; however, they are limited in accessibility due to their instability, explosiveness, and the difficulty associated with their handling. Additionally, aryl halides require a high reduction potential for activation.^375^ In contrast, diaryliodonium salts offer a more suitable alternative since they are easy to prepare, less toxic, and have a lower reduction potential compared to aryl halides, displaying reactivity comparable to aryldiazonium salts. They are highly reactive and stable, making them ideal candidates for the construction of C–C and C–heteroatom bonds through formal aryl cations and arynes. Additionally, diaryliodonium salts can exhibit radical reactivity by homolysis of the weak hypervalent bond under thermal or light-mediated conditions.^375,376^ Transition metal-free aromatic C–H arylations with hypervalent iodines, including diaryliodonium salts, have gained significant attention due to their environmentally friendly and sustainable nature, making them attractive alternatives to metal-catalyzed processes.^39,377−383^

**Scheme 46:**
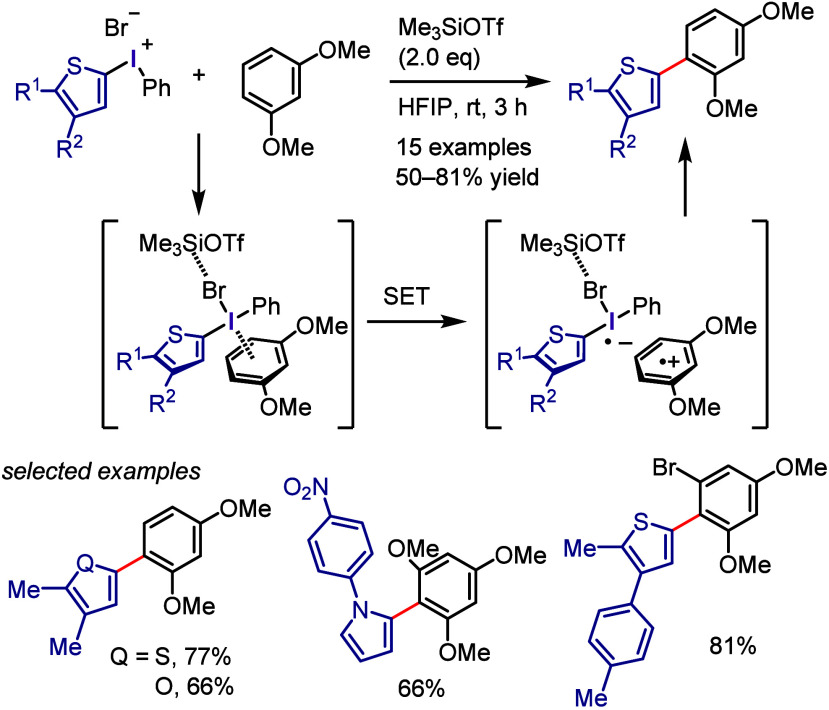
Oxidative Couplings and Biaryl Formation via a Unique SET Mechanism

Kita and his group also discovered a mechanistically different approach for the construction of biaryl that depended on the reaction conditions, and particularly the Lewis acid activator used (Scheme 46).^101^ Cross-coupling of aryl(heteroaryl)iodonium bromide with electron-rich arene in the presence of TMSOTf led to the synthesis of heteroaromatic biaryl with a unique *ipso*-substitution of the heteroaryl moiety on the iodonium salt. The reaction scope was expanded to include diverse diaryliodonium salts with electron-rich aryl moieties in addition to aryl(heteroaryl)iodonium bromide and a broad range of electron-rich (polycyclic)aromatic substrates.^101,102^ UV–vis and ESP spectroscopic studies indicated the generation of radical cation species during the reaction and consequently supported the SET reaction mechanism via EDA complex. Distinctive *ipso*-substitution of the iodonium salt through the highly chemoselective transfer of the more electron-rich thienyl moiety to the radical cation resulted in the formation of the final biaryl product after a rearomatization step.

**Scheme 47:**
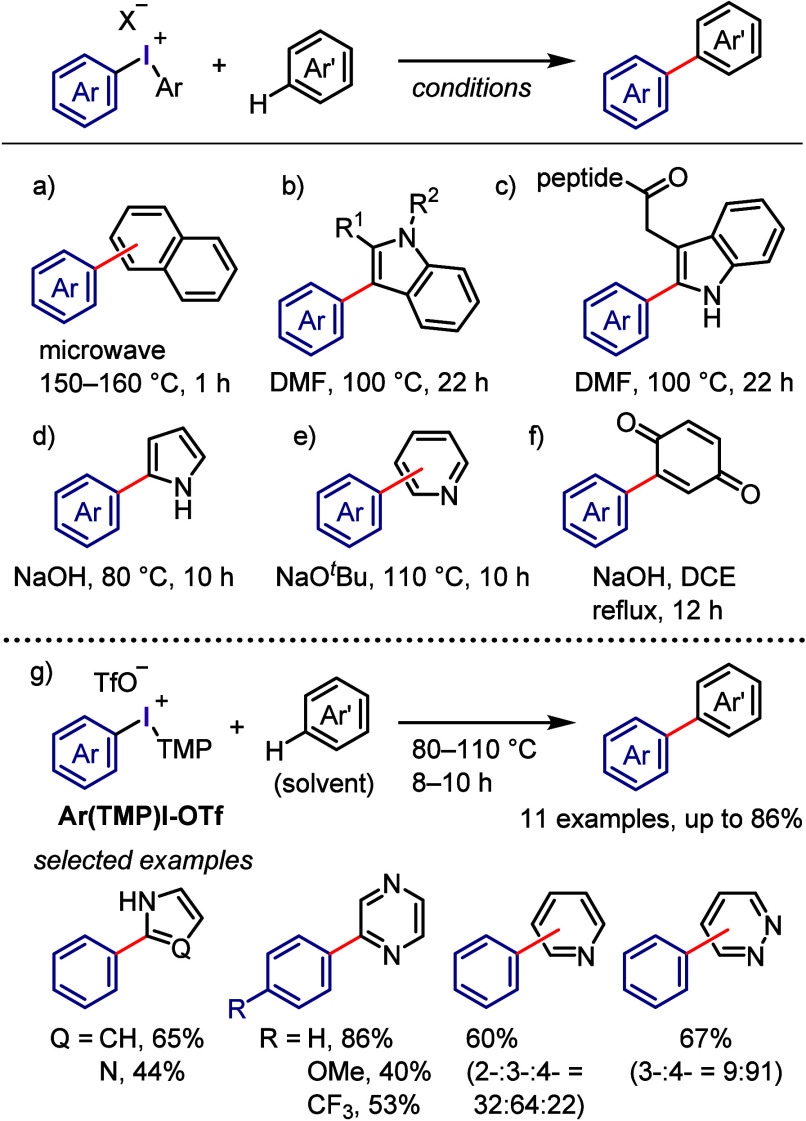
General Conditions for Csp^2^-Arylation with Diaryliodonium Salts

Arylating aromatic hydrocarbons using diaryliodonium salts under base and solvent-free microwave conditions was developed by Rodríguze et al. (Scheme 47a).^384^ Ackermann reported on the base-free site-selective C3-arylation of various substituted indoles (Scheme 47b).^385^ Oligopeptides containing indole-3-acetamide moieties were effectively arylated (Scheme 47c).^386^ Zhang, Yu, and their colleagues selectively arylated *N*-heteroarenes, such as pyrroles and pyridines, with electron-rich/poor diaryliodonium salts under basic conditions (Scheme 47d and e).^387^ Quinones such as 1,4-benzoquenone and 1,4-naphthoquenone were effectively arylated at the C2-carbon with a broad range of electron-rich diaryliodonium salts (Scheme 47f).^388^ The challenge of aryl transfer selectivity in the radical arylation of heteroarenes was tackled through the design of diaryliodonium salts (Scheme 47g).^389^ Aryl(TMP)iodonium triflates (**Ar(TMPI-OTf**) were identified to be the optimal design for base-induced radical couplings with *N*-heteroaromatic substrates, leading to the formation of aryl-heteroaryl products with selective transfer of the aryl group rather than the TMP group. The scope of heteroarenes included pyrrole, imidazole, and *N*-heteroaromatics of pyridine, pyrimidine, and pyridazine, resulting in a mixture of regioisomeric products. Notably, a one-pot sequential arylation reaction via in situ preparation of aryl(TMP)iodonium salt from iodosobenzene was also found to be applicable.

**Scheme 48:**
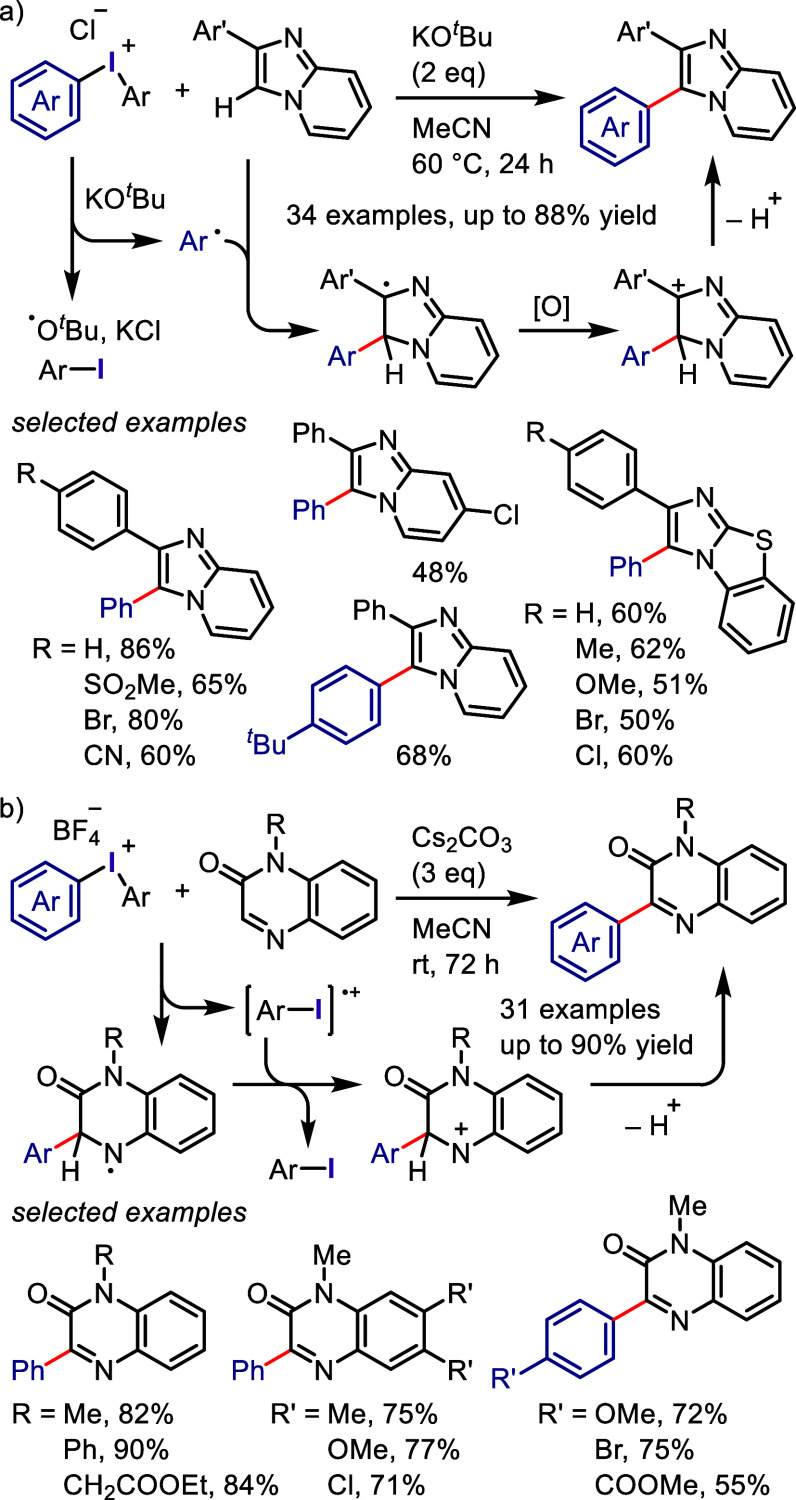
C–H Arylation of Imidazo-pyridine/Thiazole with Diaryliodonium Salts

The C3 arylation of imidazopyridines and imidazothiazoles using diaryliodonium chloride was achieved in the presence of KO^*t*^Bu (Scheme 48a).^390^ When the C3-position in the starting imidazopyridine was substituted, no reaction was observed at any other C–H positions. In contrast, the unsubstituted imidazopyridine yielded a relatively low (38%) amount of the desired C3-arylation product. Experimental studies and related reports suggested two possible free radical pathways and ionic mechanisms involving the generation of an aryne intermediate to explain the observed behavior. Successful arylation at the C3-position of quinoxaline-2-ones was achieved using diaryliodonium salts under mild conditions (Scheme 48b).^391^ Productivity of the process can be influenced by the electronic and steric effects of substituents, with electron-rich quinoxalinones giving better results than electron-deficient ones. High yields were only attained when the *N*4-imine was present, as it coordinated with the I(III) center and facilitated the generation of aryl radical species. In addition, a free radical mechanism was proposed, based on radical scavenger experiments and the ability of iodonium salts to generate aryl radical intermediates.

**Scheme 49:**
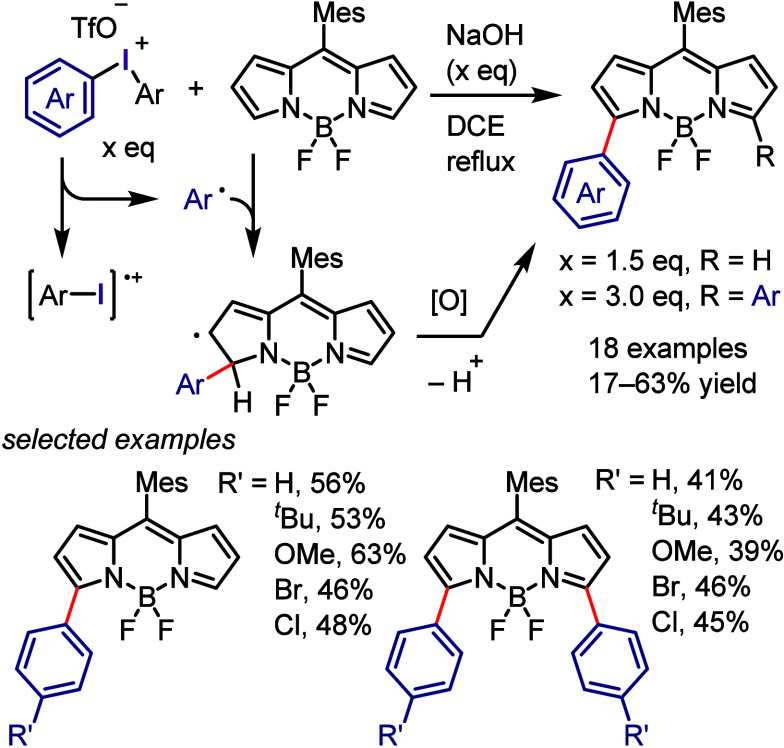
Regioselective α-Arylation of BODIPY Dyes with Diaryliodonium Salts

According to Jiao’s research, diaryliodonium salts were utilized for the direct and regioselective α-arylation of boron dipyrromethenes (BODIPYs) under mild reaction conditions (Scheme 49).^392^ As the equivalents of diaryliodonium salts increased, 3,5-diarylated BODIPYs were obtained with high regioselectivity. The high selectivity of α-arylation, as opposed to the expected β- or meso-arylation of BODIPYs, was supported by DFT calculations. A free radical mechanism, initiated by the decomposition of diaryliodonium salt to generate aryl radical, was proposed.

**Scheme 50:**
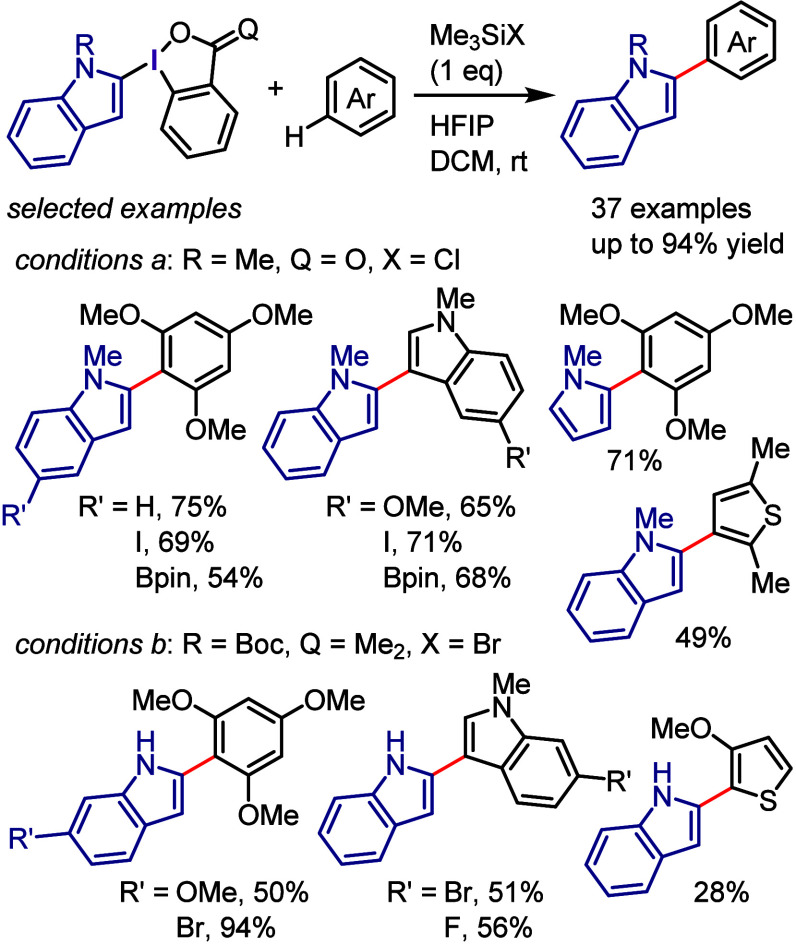
(Hetero)arylation through Oxidative Cross-coupling of (Hetero)arenes with Heteroaryl-benziodoxol(on)es

Indole-based iodonium salts could potentially be employed in cross-couplings; however, their limited availability and low stability prompted Waser and colleagues to explore more stable cyclic indole- and pyrrole-benziodoxolones for couplings with electron-rich (hetero)arenes under the conditions established by the Kita group, including TMS-Cl/Br and HFIP (Scheme 50).^393^ Notably, the coupling of 2- or 3-indolyl-benziodoxol(on)e with electron-rich (hetero)arene nucleophilic molecules resulted in the formation of related 2-(hetero)arylated indoles with high regioselectivity. Additionally, 2- or 3-pyrrolyl-benziodoxolones reacted with 1,3,5-trimethoxybenzene to generate the related 2-aryl pyrroles. The authors proposed three possible mechanisms initiated by the Lewis acid: SET from a charge-transfer complex as defined by Kita, an S_N_Ar process through either the general ligand exchange/reductive elimination, or participation of the *N*-atom of the indolyl moiety in the generation of the iodonium ylide.

**Scheme 51:**
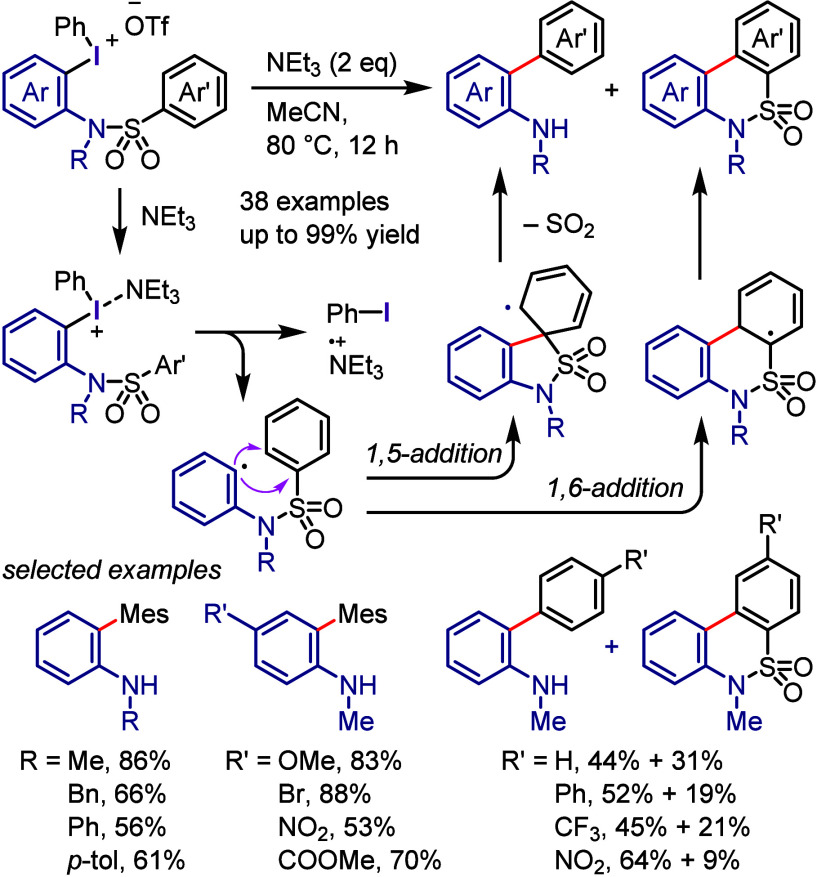
*ortho*-Substituted Diaryliodonium Salts and Construction of Hindered Biarylamines

Wang, Han, and co-workers have investigated *ortho*-functionalized diaryliodonium salts for intramolecular aryl migration and the construction of valuable skeletons (vide infra).^394−398^ They discovered that treating *N*-alkyl/aryl sulfonamide-substituted diaryliodonium salt with triethylamine resulted in desulfonylative aryl migration and the formation of sterically congested biarylamines (Scheme 51).^398^ Cyclic sulfonamides could also be produced using this method, depending on the Ar3 substituents of the aryl sulfonyl group. The reaction was initiated by electron-donor–acceptor complexation of triethylamine with iodonium salt, followed by internal SET to generate the aryl radical. This radical subsequently underwent [1,5]-substitution followed by desulfonylation and H-abstraction to ultimately yield the biaryl product. The cyclic amides were generated via [1,6]-addition and subsequent aromatization.

**Scheme 52:**
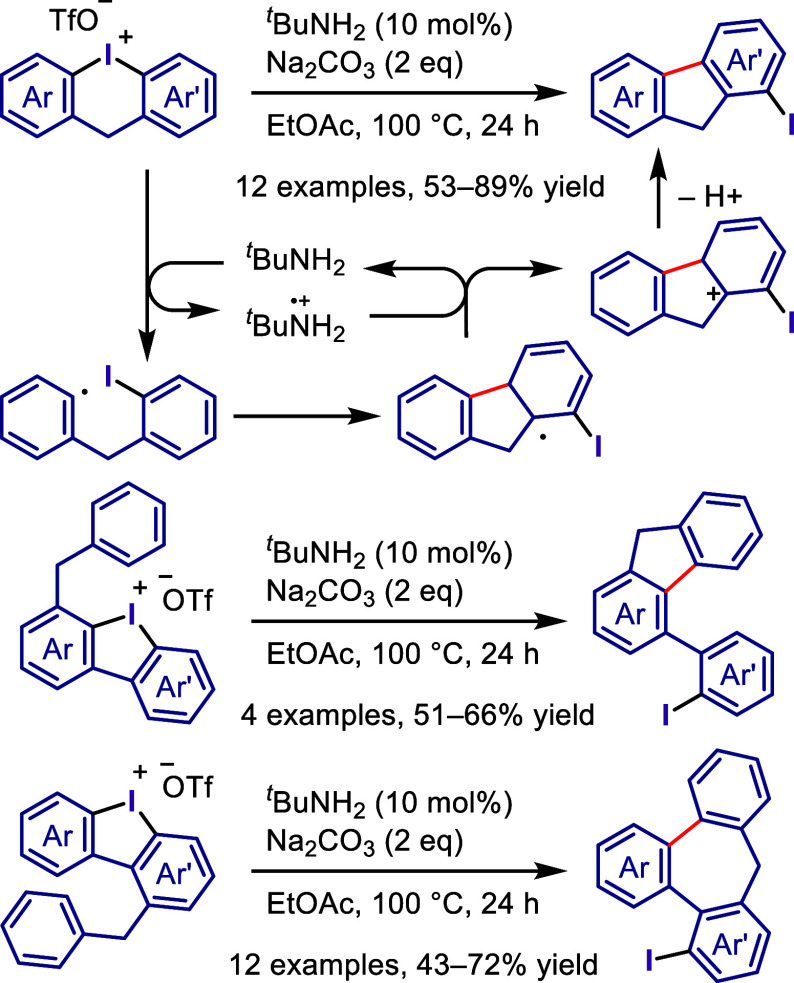
Base-Promoted Intramolecular Annulation of Cyclic Diaryliodonium Salts

Cyclic diaryliodonium triflates were designed to undergo intramolecular annulations catalyzed by alkylamine and deliver polycyclic aromatic hydrocarbon frameworks (Scheme 52).^399^ Cyclic iodonium salts underwent intramolecular ring contraction in the presence of ^*t*^BuNH_2_ to give the corresponding polycyclic fused systems. The catalytic cycle was initiated by SET from ^*t*^BuNH_2_ to the iodonium salt and the generation of a ring-closed radical, which reacted with the amine cation radical to generate a polycyclic cation intermediate. In the final step, aromatization proceeded in the presence of a base to give the fluorene derivative. Various cyclic iodonium salts successfully afforded the corresponding polycyclic arenes.

**Scheme 53:**
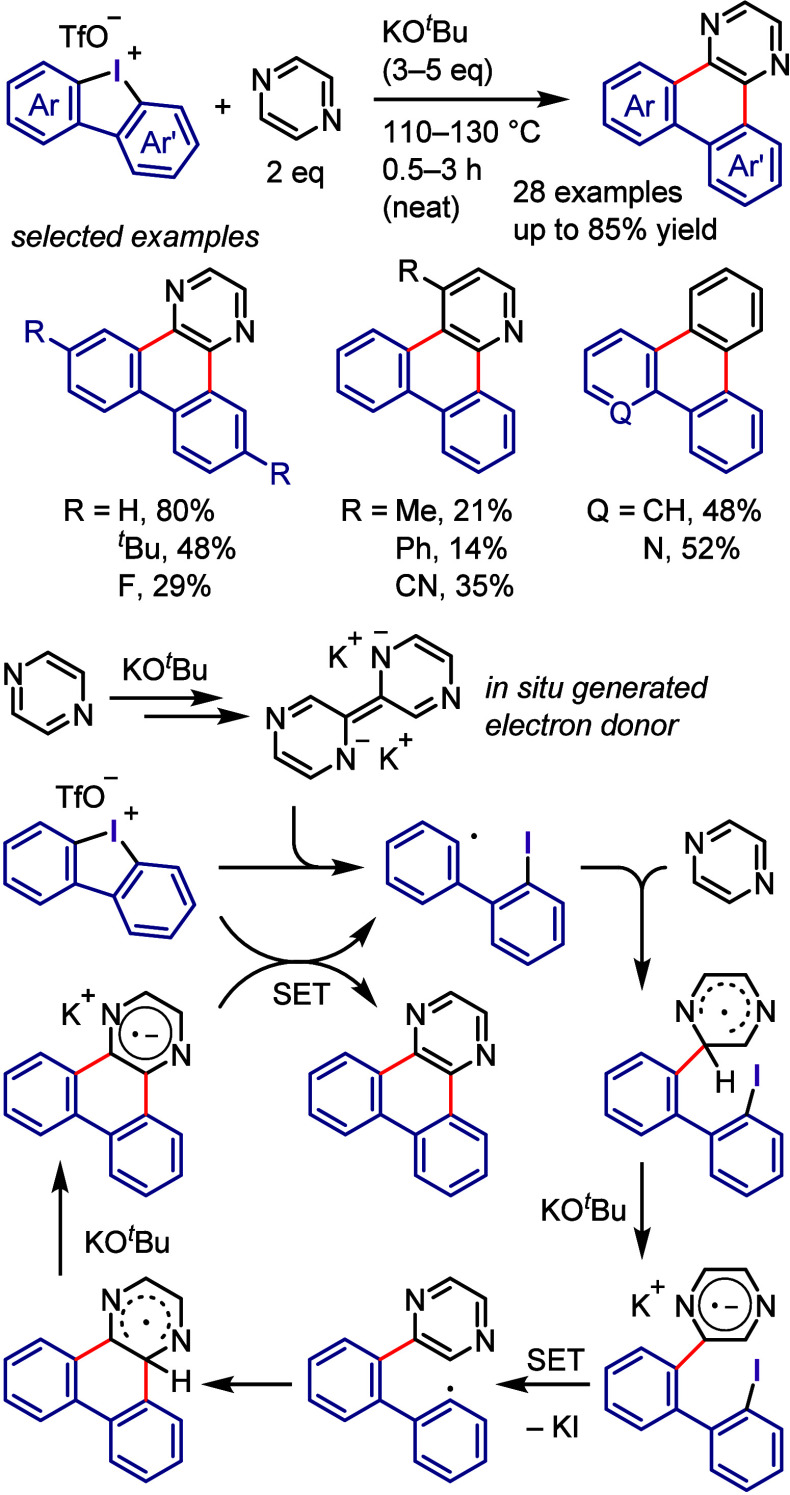
Base-Promoted Annulation of Azines with Iodonium Salt and Formation of *N*-Doped Polycyclic Aromatic Compounds

Rohde, Hong, and co-workers proposed BHAS (base-promoted homolytic aromatic substitution) as an alternative strategy to the failed annulative π-extension (APEX) approach for the construction of polycyclic *N*-heteroarenes from the reaction of azine with cyclic diaryliodonium salt (Scheme 53).^400^ In addition to the reaction as substrate, azines participated as a promoter with KO^*t*^Bu to in situ generate the organic electron donors (OEDs) that are required for the dissociation of the iodonium salt. Pyrazines, pyridines, and quinoxalines were used as azine substrates, and unactivated benzene was also used in the presence of pyrazine (2 equiv) as a promoter to give the corresponding polycyclic aromatic products. The reaction was initiated by a reaction of pyrazine 2 with KO^*t*^Bu to in situ generate dianion and radical anions species as OEDs. Reduction of the iodonium salt via SET from OEDs produced aryl radical via C–I bond homolysis. Minisci-type addition of an aryl radical to azine generated an aryl-heteroaryl radical. Subsequent deprotonation followed by intramolecular SET yielded a poly cyclic radical, which underwent deprotonation followed by SET to afford iodonium salt and then the desired product.

**Scheme 54:**
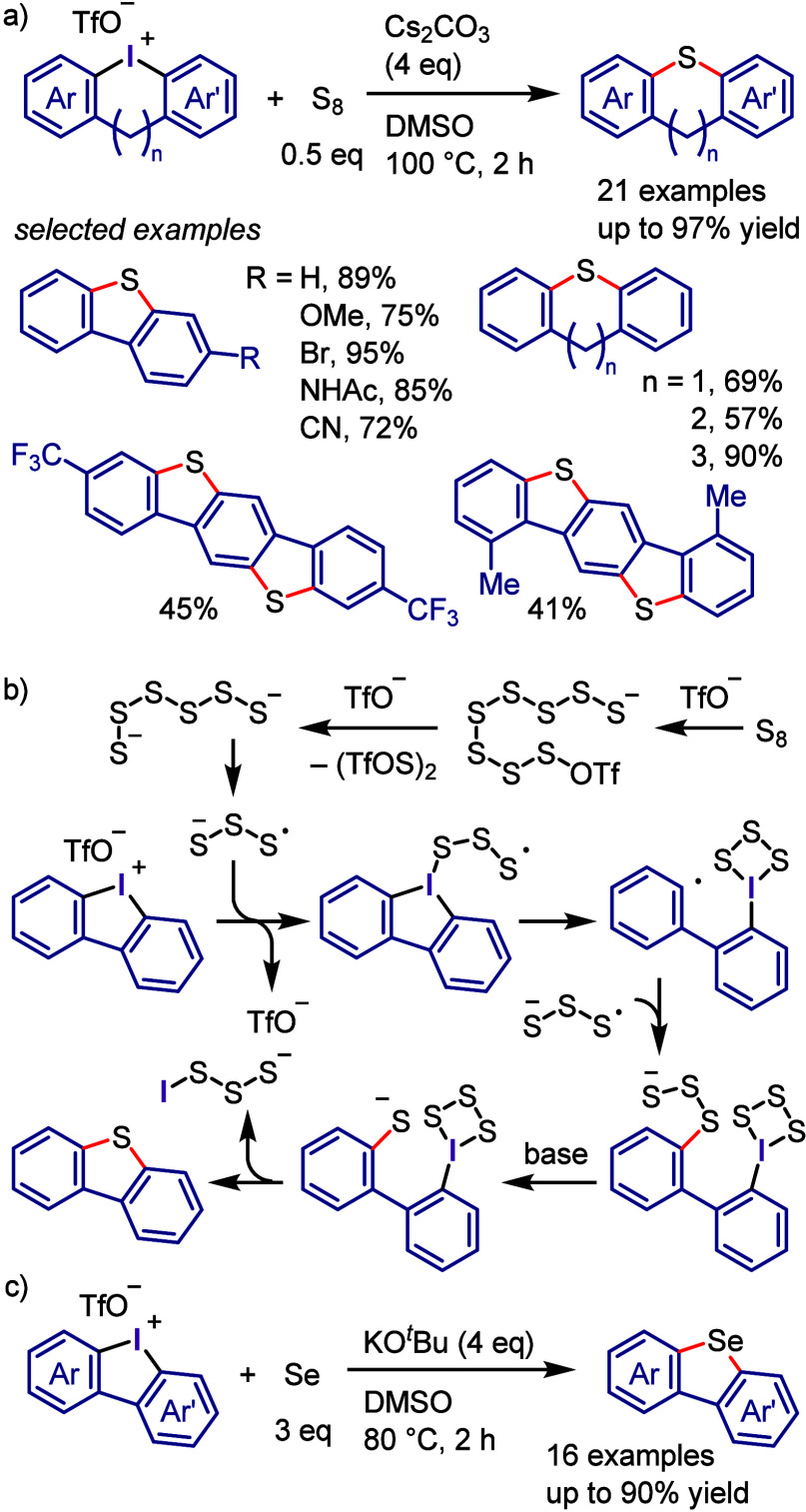
Synthesis of Annulated Sulfide and Selenide π-Conjugates via S/I and Se/I Exchange

Various aryl- and heteroaryl-flanked cyclic iodonium salts have been synthesized and transformed via S, Se, Te, and SO_2_ exchange with I to give the corresponding diary-annulated frameworks under transition metal-free conditions.^332,401,402^ The pioneering work of Jiang and co-workers included the synthesis of diaryl-annulated sulfides and selenides by reaction of cyclic diaryliodonium salt with elemental sulfur and selenium under basic conditions, respectively (Scheme 54a).^331^ This process allowed the synthesis of π-conjugated sulfide and selenide cyclic compounds as promising motifs in organic field-effect transistors (OFET). The reaction mechanism was supported by mechanistic and electron paramagnetic resonance (EPR) studies (Scheme 54b). The deep blue solution of the reactive trisulfur radical anion species was obtained by reaction of elemental sulfur with a base. During the anion exchange process, an iodonium-trisulfur intermediate was formed, followed by radical transfer from the trisulfur moiety to the aryl group and dissociation of Ar–I bond. The thus-generated aryl radical intermediate coupled with another trisulfur radical anion, and the corresponding thiophenol anion underwent intramolecular cyclization via nucleophilic substitution to afford the final product. Elemental selenium can be also employed to synthesize the corresponding diarylannulated selenides in the presence of KO^*t*^Bu (Scheme 54c).

**Scheme 55:**
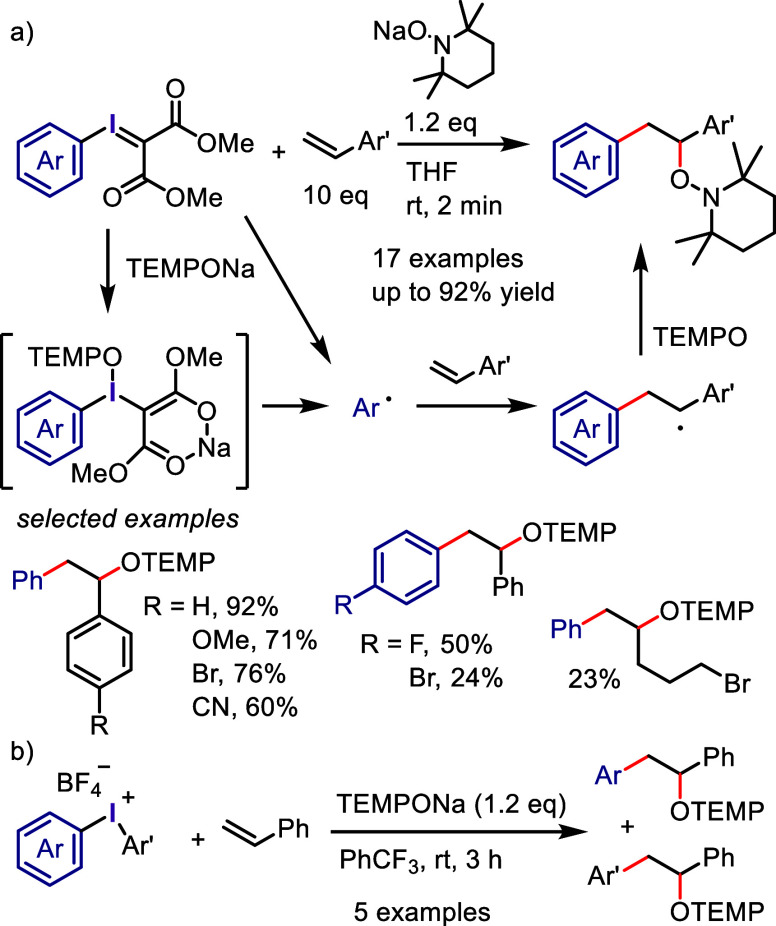
Single Electron Reduction of Iodine(III) Compounds with TEMPONa and Cascade Radical Alkene Oxyarylations

The addition of TEMPONa promotes the generation of aryl radical species from aryl-λ^3^-iodanylidene malonates, and was utilized for a three-component cascade reaction forming 1,2-oxyarylated products, reported by the Studer group (Scheme 55a).^376,403−405^ The reaction of iodanylidene malonates gave the desired oxyarylation products with excellent aryl selectivity through fragmentation of the aryl radical. The reaction was well-tolerated by various styrene substrates, but poor yields were obtained with internal alkenes and aliphatic alkenes. The reaction mechanism involved the generation of an aryl radical through either the association of TEMPONa with iodine(III), followed by inner-sphere SET or direct reduction through outer-sphere SET to generate the aryl radical. The addition of the aryl radical to the alkene resulted in the formation of an alkyl radical, which coupled with the persistent TEMPO radical to produce the observed product. When unsymmetrical diaryliodonium salts (Ar ≠ Ar’) were employed as the aryl source, mixtures of oxyarylation products were observed (Scheme 55b).

**Scheme 56:**
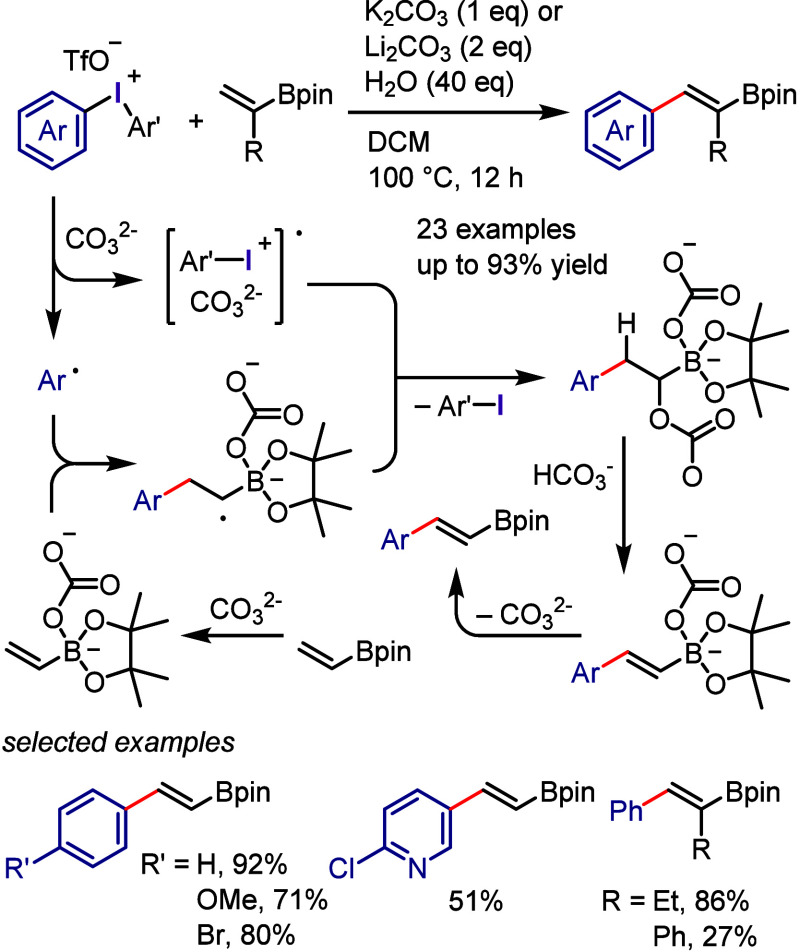
Wet Carbonate Promoted the Arylation of Vinylboronates with Iodonium Salt

The arylation reaction between vinyl pinacolboronates and diaryliodonium triflates resulted in the formation of *trans*-arylvinylboronates through the unique involvement of wet carbonates (Scheme 56).^406^ The reaction mechanism commenced with the ionization of K_2_CO_3_ by water to generate the carbonate anion (CO_3_^2–^), which led to the generation of an aryl radical and [Ph-I^+^][CO_3_^2–^]^•^. Meanwhile, the aryl radical reacted with CO_3_^2–^-activated vinyl boronate to give α-boronate radical, which underwent SET with [Ph-I^+^][CO_3_^2–^]^•^ followed by deprotonation and the subsequent intramolecular release of bicarbonate to afford the final product.

**Scheme 57:**
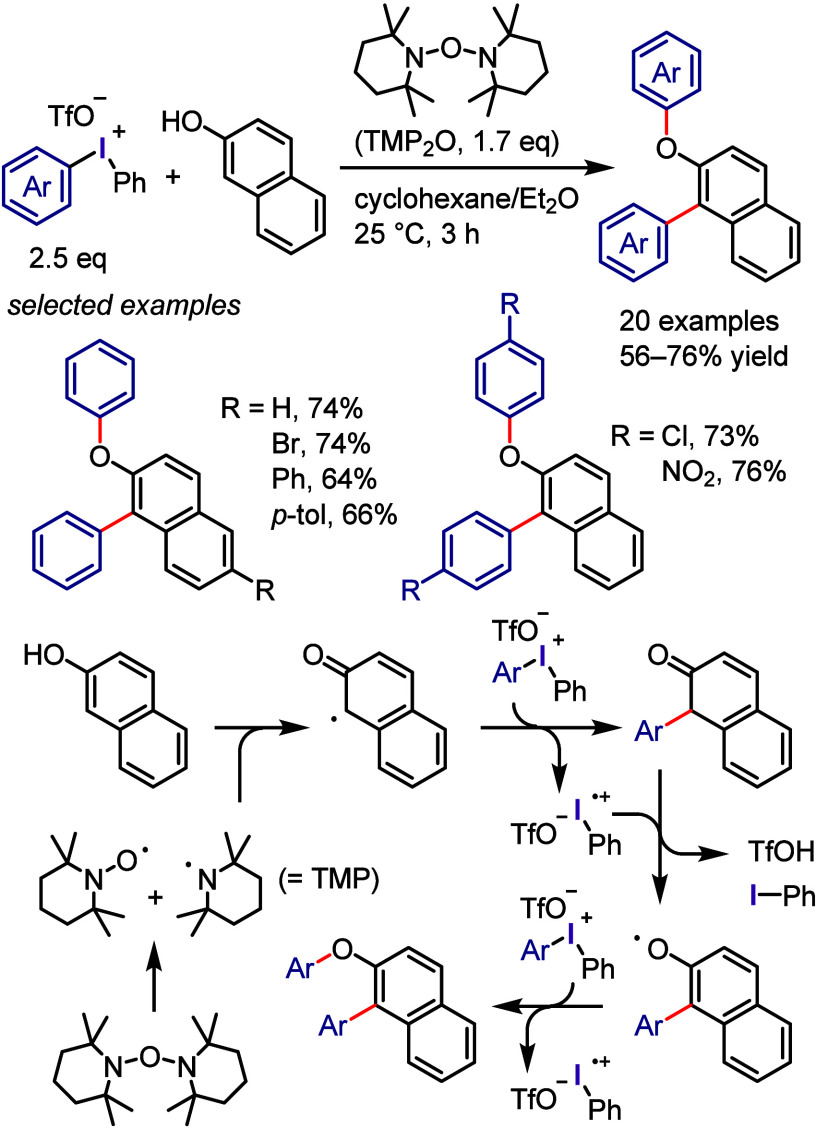
TMP_2_O Mediated Double Arylation of 2-Naphthol with Diaryliodonium Triflates

In addition to the former protocols for functionalization of 2-naphthol with iodonium salts,^220,288,289^ Solorio-Alvarado and co-workers discovered a new activation mode for diaryliodonium salts during *C*- and *O*-double arylation of 2-naphthols in the presence of the radical precursor TMP_2_O [1,1′-oxybis(2,2,6,6-tetramethylpiperidine)] under base-free conditions (Scheme 57).^407^ Experimental and DFT studies indicated a radical mechanism, initiated by TMP_2_O via spontaneous N–O bond homolysis to give TMP^•^ and TEMPO. HAT from 2-naphthol to TMP^•^ provided a *C*-radical, which reacted with diaryliodonium triflate to generate a *C*-arylated intermediate. The second equivalent of diaryliodonium salt delivered the diarylated product.

**Scheme 58:**
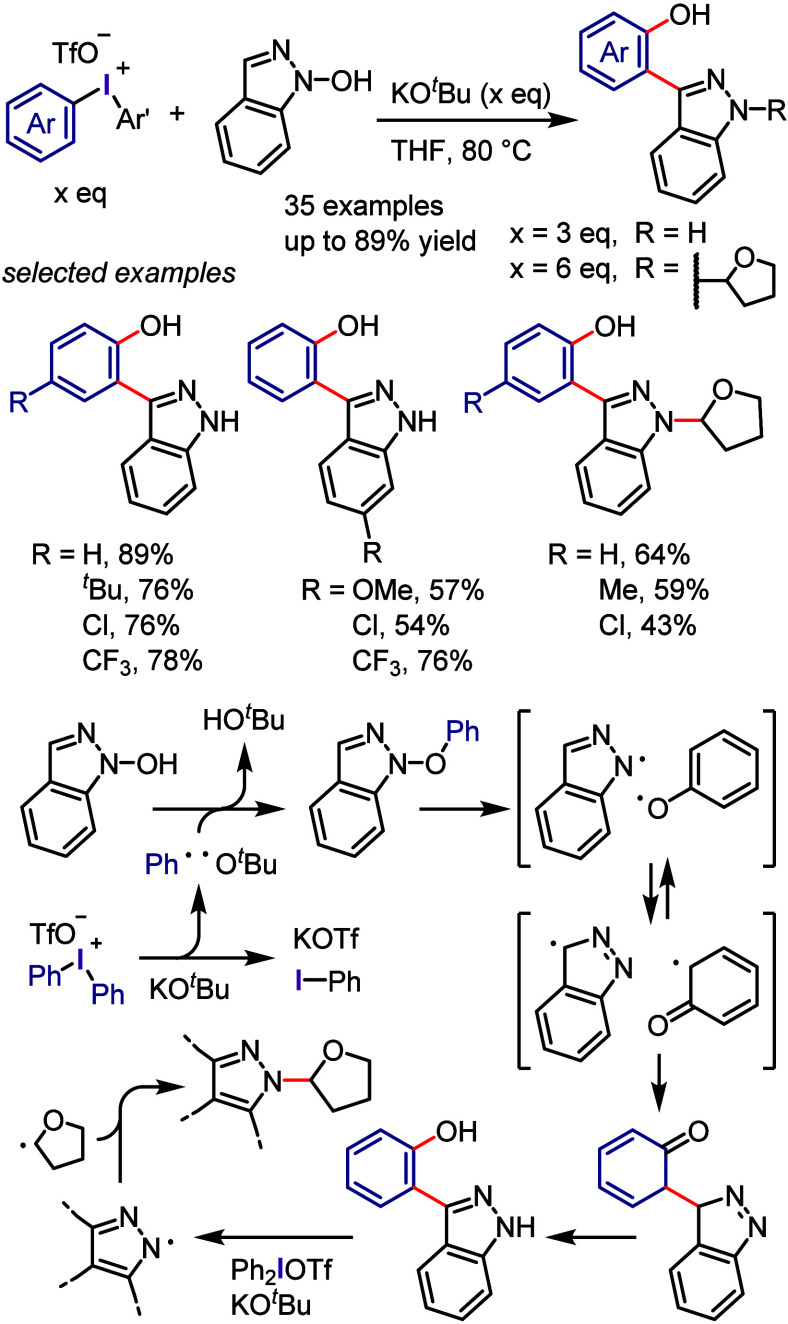
Radical *O*-Arylation and [3,3] Rearrangement to Afford C3-Hydroxyarylindazoles

Radical *O*-arylation of *N*-hydroxyindazoles with diaryliodonium salts and subsequent [3,3] rearrangement was observed to be condition-dependent (Scheme 58).^408^ 3 equiv of diaryliodonium salt and KO^*t*^Bu produced the corresponding and highly preferred 3-(2-hydroxyaryl)indazoles, whereas 6 equiv of diaryl iodonium salts and KO^*t*^Bu gave the corresponding dehydrogenative cross-coupling products *N*-(tetrahydrofyran-2-yl)-3-(2-hydroxyaryl)indazoles. Based on mechanistic studies, the postulated mechanism was initiated by the generation of aryl and ^*t*^BuO radicals, which then reacted with *N*-hydroxyindazole to generate phenoxy and indazole radicals. Subsequently, radical–radical coupling followed by rearomatization afforded the desired product.

**Scheme 59:**
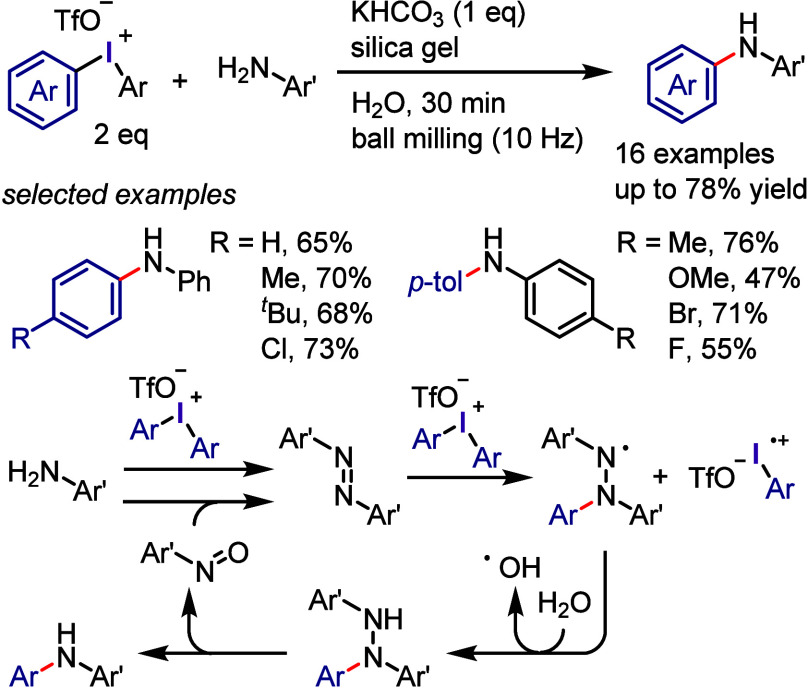
*N*-Arylation of Anilines with Iodonium Salt Using the Ball-Milling Technique

Li and co-workers replaced the traditional reaction conditions with liquid-assisted ball-milling conditions for *N*-arylation of aniline derivatives with diaryliodonium triflates (Scheme 59).^409^ In contrast to the popular nucleophilic substitution mechanism of this transformation, this reaction proceeded via a radical mechanism. Oxidation of aniline by diaryliodonium triflate afforded azobenzene, which attacked via the phenyl radical generated from the homolytic cleavage of Ar–I^(III)^ to give an amidyl radical. In the presence of H_2_O, the amidyl radical was protonated to give a hydrazine derivative, which collapsed to afford the desired product along with nitosobenzene.

### 2.5 Arylation via Generation of Arynes

In addition to acting as aryl cation and aryl radical precursors, diaryliodonium salts are superior candidates for aryne precursors due to the high electrophilicity of the central I^III^ atom and the hypernucleofuge capability of aryl-λ^3^-iodane (hyperleaving group) which is a million times more than the triflate group.^410−412^ The generated aryne intermediates provide double functionalization of arenes in a single step, which is a highly desirable synthetic approach.^116^

**Scheme 60:**
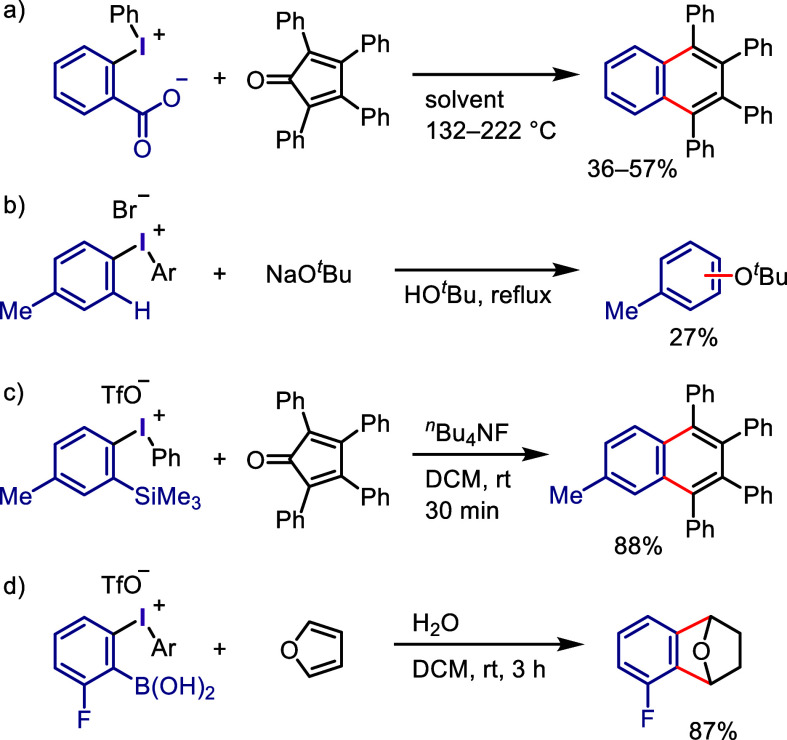
Generation of the Aryne from *ortho*-(Un)functionalized Diaryliodonium Salts

Originally, Beringer et al. noticed the decomposition of diphenyliodonium-2-carboxylate (also known as phenylbenziodoxole) at high temperatures (>180 °C) and generation of benzyne that was trapped by broad range of arynophiles (Scheme 60a).^413^ The ability of diaryliodonium salt to generate an aryne intermediate through deprotonation of *ortho*-hydrogens with strong base was discovered by Akiyama.^414^ The reaction of di(4-tolyl)iodonium bromide in the presence of NaO^*t*^Bu under reflux conditions afforded a 27% combined yield of 3-tolyl- and 4-tolyl-ethers in a 1:1 ratio through the in situ generation of the aryne (Scheme 60b). Kitamura and co-workers designed (phenyl)[*o*-(trimethylsilyl)aryl]iodonium triflate as an aryne precursor to solve the previously associated challenges of the generation of more than one aryne intermediate, the use of a strong base and the requirement of harsh conditions (Scheme 60c).^415−417^ Controlling the reaction temperature led to a chemoselective generation of the aryne functionality. Thus, double cycloadditions with two different arynophiles in a sequential one-pot reaction produced complex polycyclic aromatic compounds. This study represented the unique reactivity and hypernucleofugality of the aryliodonium group in the presence of a triflate-leaving group.^21−23^ Yoshimura, Zhdankin, and co-workers developed a substrate analogous to Kitamura’s substrates for the generation of aryne under benign conditions (Scheme 60d).^418,419^ The presence of *ortho*-B(OH)_2_ to the hypernucleofugic aryliodonium group made the boronic acid group more oxophilic and allowed it to be triggered by water as the only activator.

**Scheme 61:**
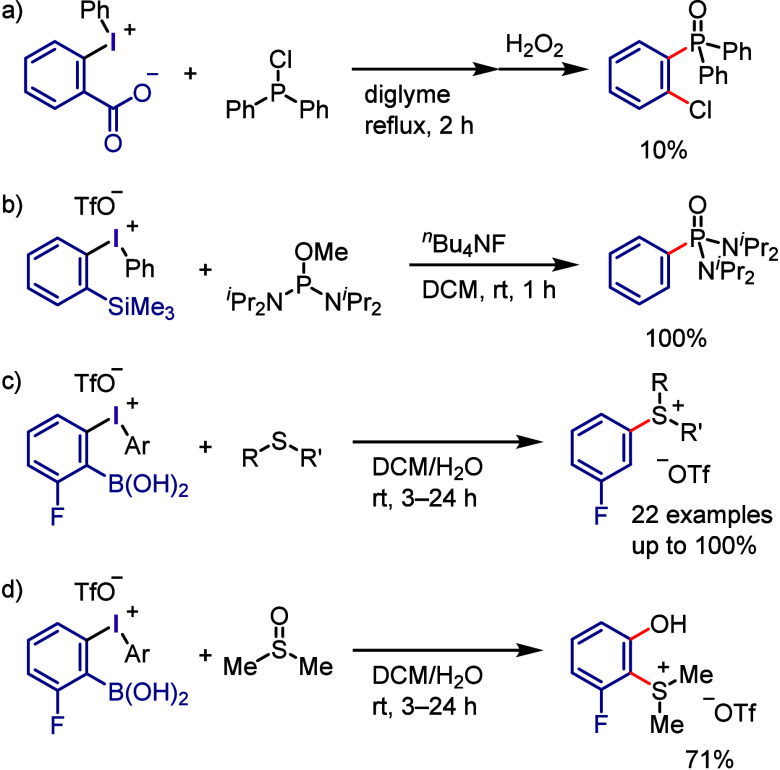
Ar–P and Ar–S Bonds Formation with Diaryliodonium Salts

Aryne generation from *ortho*-functionalized diaryliodonium salts was applied to C–P and C–S bond formations (Scheme 61). Chlorodiphenylphosphine was used to trap the benzyne intermediate generated from the collapse of diphenyliodonium-2-carboxylate, then oxidation with hydrogen peroxide yielded the *ortho*-chlorophenyldiphenylphosphine oxide (Scheme 61a).^420^ Kitamura and Stang’s benzyne precursor reacted with methyl phosphorodiamidite in the presence of TBAF to produce phenylphosphonic diamide in a quantitative yield (Scheme 61b).^421^ Yoshimura, Zhdankin, and co-workers used pseudocyclic arylbenziodoxaborole triflate for a reaction with organic sulfides and the synthesis of the related sulfonium salts with high regioselectivity (Scheme 61c).^422^ A broad library of cyclic/acyclic dialkyl, aryl alkyl, and diaryl-sulfides were tolerated under the reaction conditions to give the corresponding *meta*-fluorophenyl-substituted sulfonium salts. Additionally, dimethyl and methyl phenyl sulfoxides were incorporated to give *ortho*-hydroxy-substituted sulfonium salts via stepwise formation of four-membered cyclic intermediate (Scheme 61d).

**Scheme 62:**
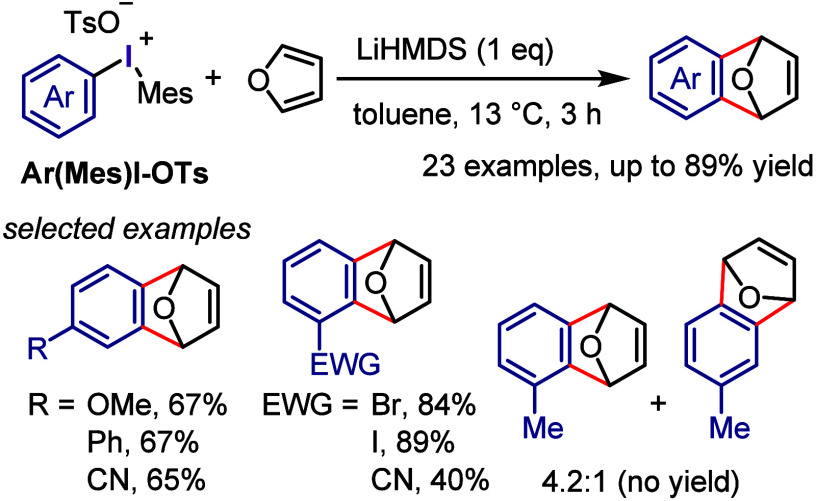
Selective Generation of Arynes from Diaryliodonium Salts

Using unsymmetrical diaryliodonium salts with *ortho*-hydrogens on the two aryl ligands for the generation of arynes through a C–H deprotonation approach is an uncontrollable strategy because of the possibility for generation of up to four chemo- and regioselective arynes. Therefore, Stuart and co-workers employed, under basic LiHMDS conditions, the synthetically accessible aryl(Mes)iodonium tosylate (**Ar(Mes)I-OTs**) for chemospecific generation of arynes and extrusion of mesityl iodide, which can be recycled (Scheme 62).^237,423^ The generated arynes were trapped through [4 + 2] cycloaddition with furan to evaluate the regioselectivity of the deprotonation process. When the aryl groups have electron-rich/poor substituents at C-4, the desirable oxabicyclic products were produced in high yields. Notably, iodonium salts substituted with inductively withdrawing groups at the C-3 position led to deprotonation of the hydrogen at the C-2 position with high regioselectivity (>20:1). However, substitution of the aryl ligand of the iodonium salt with an electron-donating and sterically hindered methyl group at the C-3 position afforded mixture of regioisomers.

**Scheme 63:**
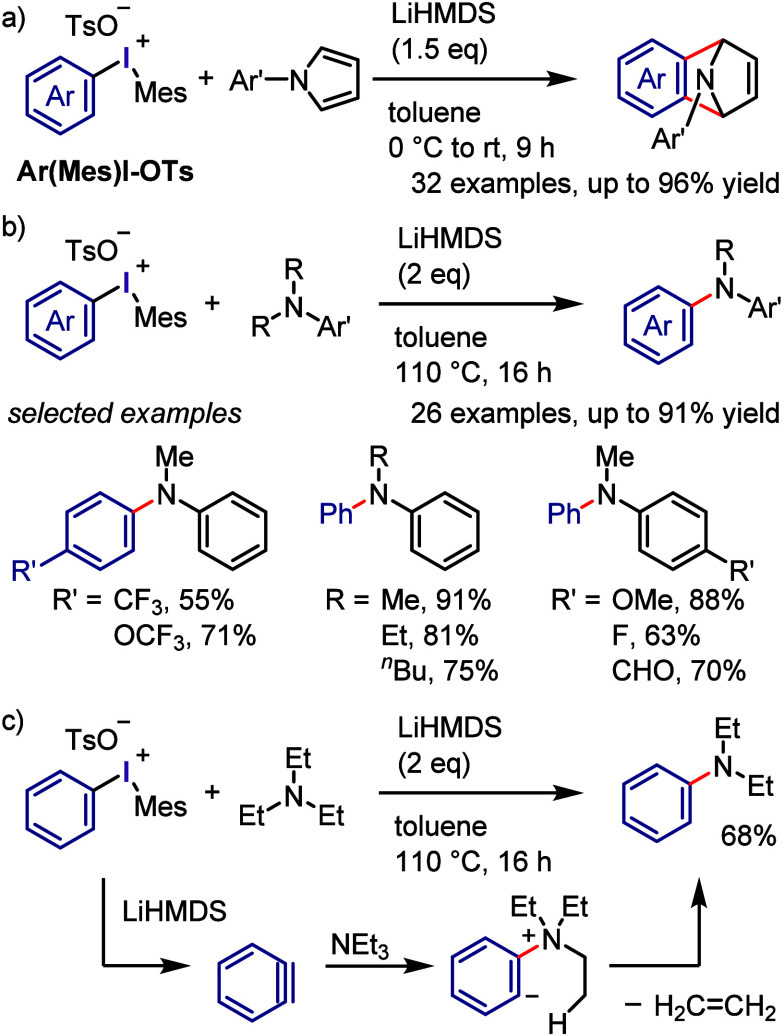
Reaction of Aryne with Pyrrole or Tertiary Amines

In the same vein, Wang and collaborators used **Ar(TMP)I-OTs** for the generation of arynes in the presence of LHMDS (1.5 equiv), and cycloaddition with *N*-arylpyrroles to yield the corresponding bridged amine cycloadducts (Scheme 63a).^424^ The observed trend of chemo- and regio-selectivity was in accordance with Stuart’s trend. The same group developed a direct *N*-arylation of tertiary amines by using aryl(mesityl)iodonium tosylates under LiHMDS/toluene/110 °C conditions (Scheme 63b);^425^ the reaction worked well with aliphatic tertiary amines and electron-rich aromatic tertiary amines. A possible mechanism was suggested, beginning with the base-promoted generation of aryne from the iodonium salt, followed by nucleophilic attack by the tertiary amine to generate a zwitterion intermediate, and finally a proton transfer to afford the final product (Scheme 63c).

**Scheme 64:**
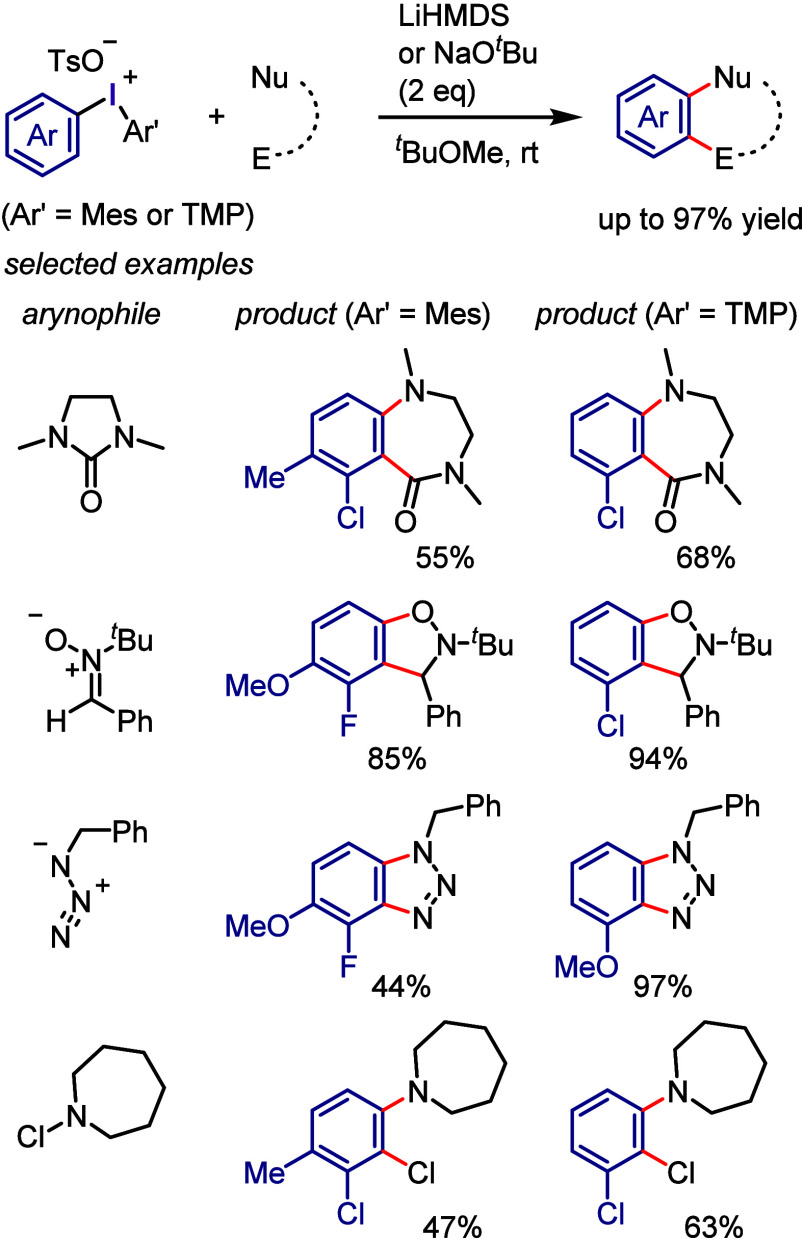
Reaction of Arynes with Various Arynophiles

Stuart et al. treated 1,2-disubstituted-aryl(Mes)iodonium tosylates with NaO^*t*^Bu to generate arynes that subsequently reacted in situ with arynophiles to finally afford 1,2,3,4-tetrasubstituted arenes (Scheme 64).^426^ The strong electron-withdrawing effect and superior leaving ability of the iodonium group, facilitated by the substituents at the 1- and 2-positions, allowed a concerted regioselective deprotonation at the 3-position and the chemo- and regioselective generation of the aryne. The limitations of aryl(Mes)iodonium tosylates motivated the same group to develop aryl(TMP)iodonium tosylates as a relatively more reactive species and achieve control of the reaction pathway for the selective generation of arynes.^427^ Reactions of a broad range of aryl(TMP)iodonium salts and arynophiles were performed in the presence of an appropriate base. The reaction of 3-chlorophenyl(TMP)iodonium tosylate and furan was used as an ideal system for one-pot competition reactions between various arynophiles and furan to measure the relative reactivity of these arynophiles and parametrize them under a single arynophilicity (A) value scale.^428^

Furthermore, Stuart and co-workers reported aryne generation under mild basic conditions using K_3_PO_4_.^429^ Similarly, Han and collaborators substituted aryl(Mes)iodonium salts with *meta*-OTf groups to generate an aryne intermediate under mild K_2_CO_3_ basic conditions.^430^ Various *meta*-OTf iodonium salts reacted smoothly with (substituted)furans, azide, amine, and ethylene arynophiles. *Meta*-substituted iodonium salts showed a higher reactivity than their *para*-substituted counterparts with excellent regioselectivity. These conditions tolerated a broad array of iodonium salts and arynophiles with sensitive functional groups such as benzyl halide/alcohol, boronate esters, and ketones. Quantitative analysis of functional group compatibility demonstrated that this approach is more functional group compatible than other known methods.

**Scheme 65:**
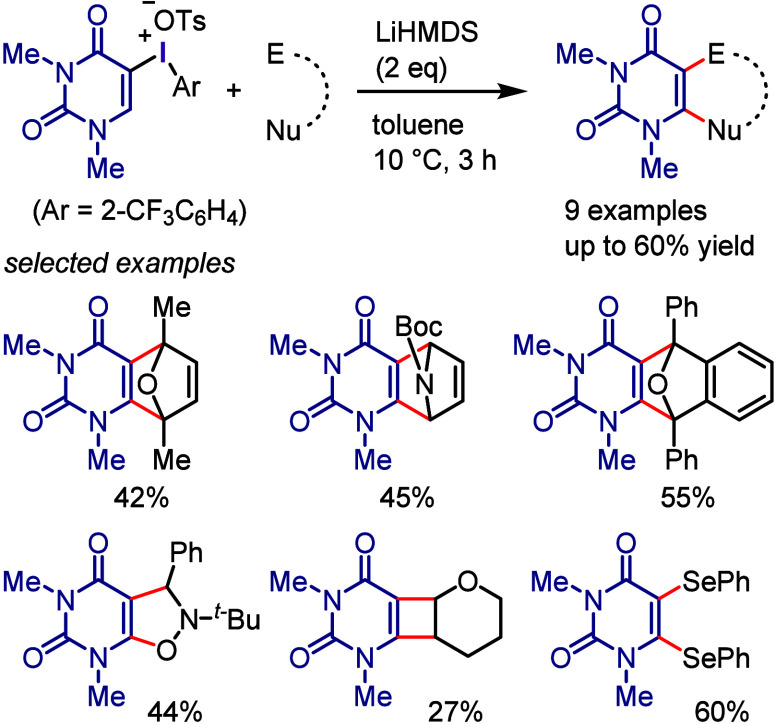
Generation of Uracil-heteroaryne from Uracil-iodonium Salt and Participation in Cycloaddition and Insertion Reactions

Takenaga and Dohi studied the potency of uracil(aryl)iodonium salts for the highly chemoselective generation of aryne/heteroaryne. Treating the uracil(aryl)iodonium salt with LiHMDS in the presence of arynophile exclusively gave the uracil-adduct via in situ generation of the heteroaryne analogue of uracil (uracilyne) (Scheme 65).^121,431,432^ Optimization of the iodonium salt revealed that 2-trifluoromethylphenyl group and tosylate counteranion were important for improving the chemical properties of the iodonium salt and increasing the yields. The reactivity of the generated uracilyne tolerated a broad range of cycloaddition and σ-bond insertion reactions. Unsymmetrical arynophiles were converted to the corresponding cycloadducts with excellent chemo- and regio-selectivity.

**Scheme 66:**
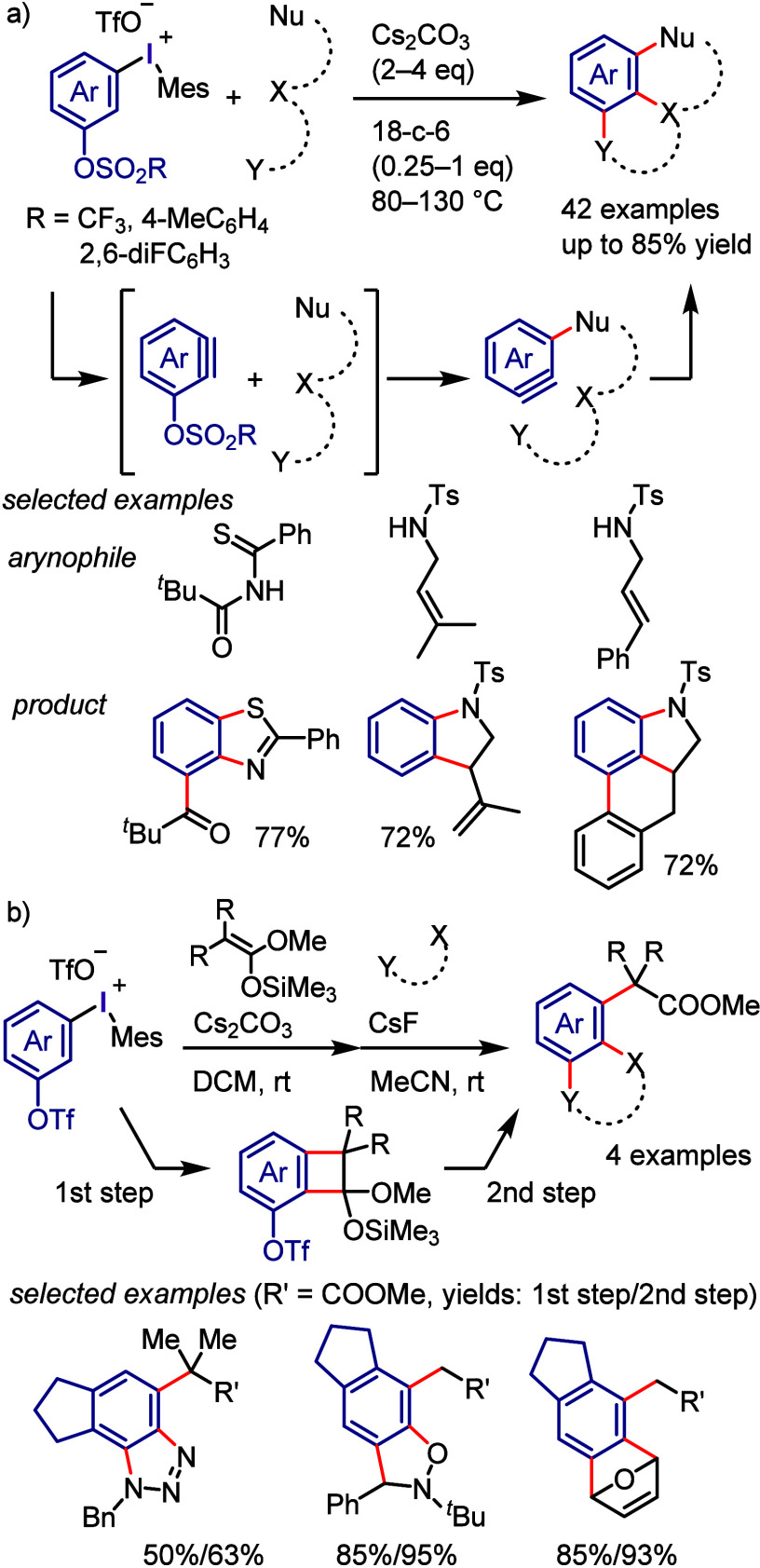
Sequential Transformation of Aryne Generated from Diaryliodonium Salts

The Li group designed densely substituted 3-sulfonyloxyaryl(mesityl)iodonium triflates as efficient and high-functional-group-economy 1,2-benzdiyene precursors (Scheme 66a).^433^ The presence of the mesityliodonium moiety at the *meta*-position of the sulfonyloxy group allowed the *ortho*-deprotonative elimination strategy to generate 1,2-aryne under weakly basic conditions, in addition to predicting the site-elective generation of aryne and the subsequent aryne transformation. Consequently, deprotonation of the *ortho*-proton led to regioselective generation of an aryne intermediate. The presence of the 3-sulfonyloxy group directed the nucleophile to attack the *meta*-position and released 2,3-aryne, which subsequently participated in the cascade process to finally afford the multisubstituted arene product. The mild conditions applied tolerated 1,2-benzdiyne precursors and were applicable to diverse tandem reactions besides the two-step [2 + 2] cycloaddition and Grob-fragmentation process, accessing chemically and biologically useful skeletons (Scheme 66b).

### 2.6 Transformations to Iodine-Containing Products

Diaryliodonium salts are superior reagents in terms of reactivity and stability for arylating diverse substrates and forming C–C and C–heteroatom bonds. The traditional reaction of diaryliodonium salt involves transferring one of its aryl ligands to the substrate to afford the product, while the other aryl ligand is reductively eliminated in a stoichiometric amount as aryl iodide waste along with the counteranion. Therefore, the atom economy of this process was approximately 10–20%, as estimated by Nachtsheim and co-workers.^434^ This disadvantage could be overcome by using various recyclable hypervalent iodine techniques, such as polymer-supported, nonpolymeric, ionic-liquid/ion-supported, and metal–organic framework (MOF)-hybridized reagents.^19,434^

Several protocols were developed to improve the overall atom economy of the process and afford highly functionalized products in a one-pot reaction.^435−438^ These elegant strategies relied on the incorporation of the two aryl ligands of the acyclic iodonium salt in the final product through a one-pot cascade reaction, in which the aryl iodide waste from the first step successively participated as a reagent in the second step. Additionally, connecting the two aryl ligands of the iodonium salt in one system (cyclic diaryliodonium salt) was a sophisticated strategy for designing high atom-economical processes. These earlier atom-efficient transformations of acyclic and cyclic diaryliodonium salts were catalyzed by transition metals.^183,402,434,439,440^ Herein, we will cover the recent transition metal-free strategies which were developed to improve the sustainability, practicability, and applicability of these atom-economical processes.

**Scheme 67:**
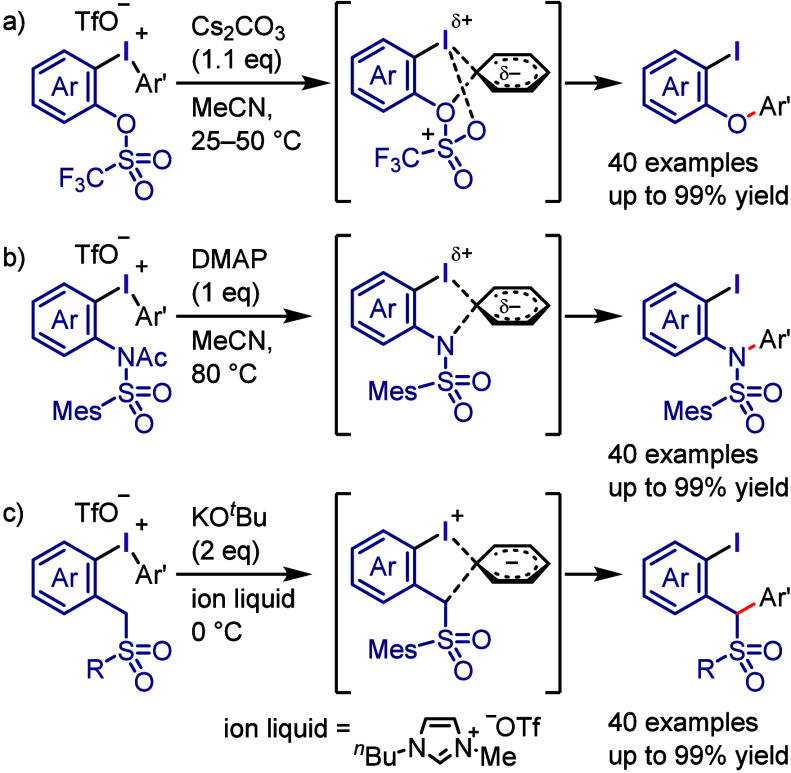
Intramolecular Aryl Migration of Iodonium Salt and the Formation of Iodo-products

*ortho*-Functionalized diaryliodonium salts were designed by Wang, Han, and co-workers to undergo intramolecular aryl migration and deliver useful products with available *ortho*-iodo-substituents for further derivatization.^394−398^ The strategy started with diaryliodonium salts bearing *ortho*-trifluoromethylsulfonyloxy groups designed to undergo intramolecular aryl migration and deliver the corresponding *othro*-iodo diaryl ethers (Scheme 67a).^394^ The reaction proceeded through an intramolecular S_N_Ar mechanism directed by the sulfonyl group at the *ortho*-position. The base was important for trapping the liberated anion and accelerating the dissociation of the sulfonyl group. The same hypothesis was applied to a two-step protocol involving C–H activation of complex aromatic hydrocarbons and synthesis of diverse *ortho*-OTf substituted diaryliodonium salts followed by site-selective O-arylation via an intramolecular aryl migration mechanism.^395^ Further investigations delivered a *N*-acetyl sulfonamide substituted diaryliodonium salt as an ultimate scaffold for intramolecular aryl migration in the presence of DMAP to afford *ortho*-iodo *N*-aryl sulfonamides (Scheme 67b).^397^ The reaction mechanism started with deacetylation of the *N*-Ac group using DMAP followed by S_N_Ar via a *spiro*-Meisenheimer complex to afford the final product.

The same group designed *ortho*-sulfonylmethylene-substituted diaryliodonium triflates for an efficient intramolecular S_N_Ar (Truce-Smiles rearrangement) and the synthesis of *ortho*-iodo-diarylmethylene sulfones (Scheme 67c).^441^ Ionic liquid (IL) was important for stabilization of the transition states and achieving the reaction under mild conditions with high yields.

**Scheme 68:**
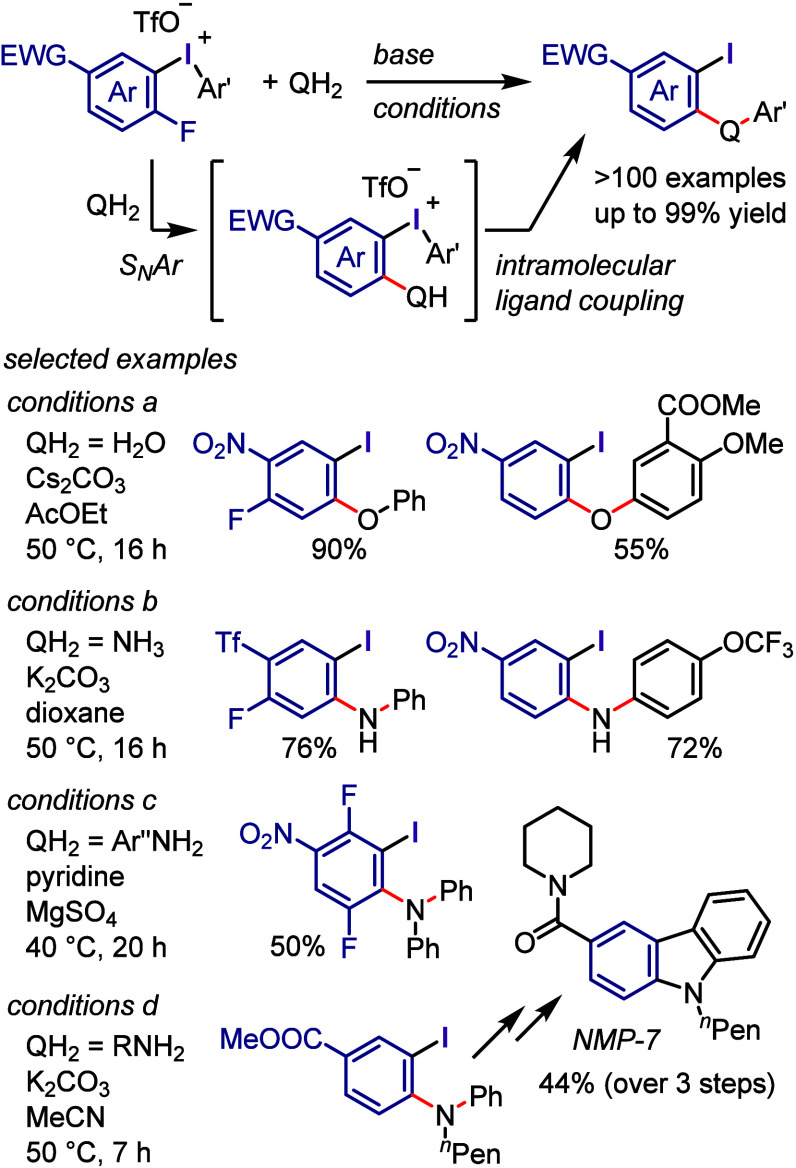
Atom-Economic Diarylation of Primary Amines, Ammonia, and Water

Olofsson et al. combined the benefits of the S_N_Ar methodology and arylation with diaryliodonium salt in one cascade process to difunctionalize primary amines, ammonia, and water (Scheme 68).^442,443^*Ortho*-fluorinated diaryliodonium salts with an additional electron-withdrawing group (EWG) substituted *para* to the fluoro-group were designed and synthesized to react with amine/ammonia/water nucleophiles under mild conditions. The presence of two EWGs in the *ortho*- and *para*-positions to the fluorine atom caused the S_N_Ar reaction with *N*- and *O*-nucleophiles to generate the new iodonium salt intermediate, which underwent intramolecular aryl transfer via I^(III)^–Ar bond dissociation to afford the diarylated products. Furthermore, the retained iodine atom in the products allowed further derivatization as demonstrated by the conversion to NMP-7.

**Scheme 69:**
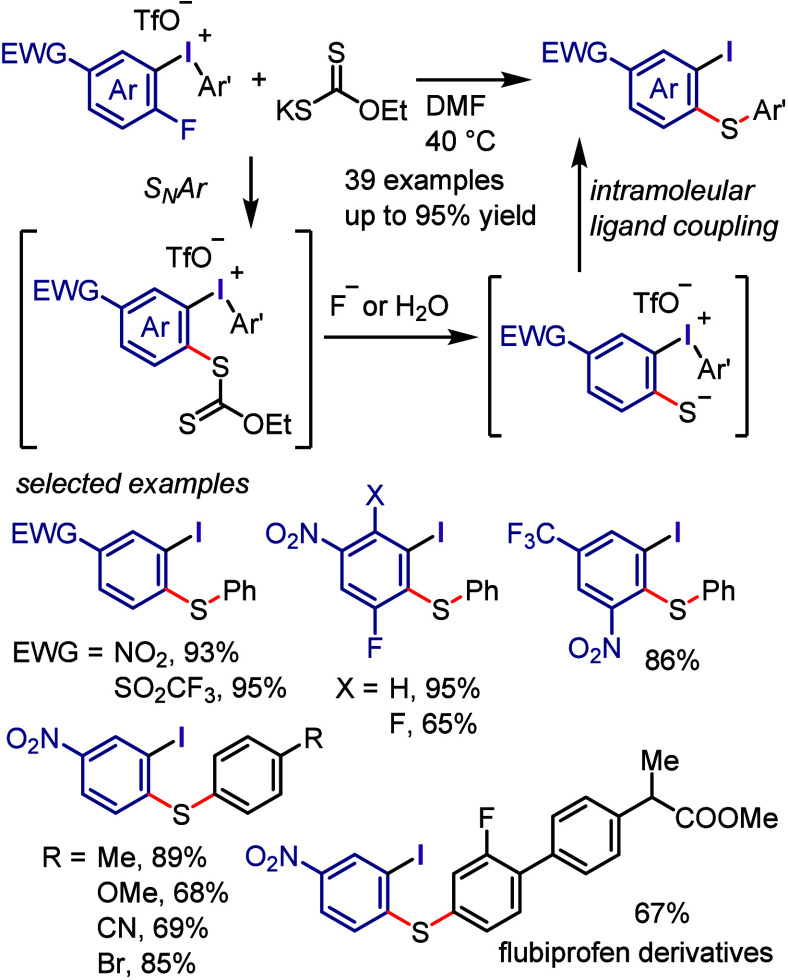
Atom-Economic Diarylation of a Sulfur Nucleophile with Diaryliodonium Salts

In addition, Olofsson and colleagues used the previous diaryliodonium salts for developing a novel strategy for diarylation of a sulfur nucleophile (potassium ethyl xanthogenate) and to access an iodo-substituted unsymmetrical diaryl sulfides under base- and thiol-free mild conditions (Scheme 69).^444^ The reaction conditions tolerated a broad range of functional groups and were employed for the synthesis of complex products using diaryliodonium salts derived from heterocycles, bioactive compounds, and drug molecules. The proposed mechanism was supported by experimental and theoretical DFT studies. The reaction of diaryliodonium salts with *S*-nucleophiles underwent S_N_Ar followed by nucleophilic cleavage of the xanthogenate moiety. Intramolecular aryl transfer produced the desired diaryl sulfides.

**Scheme 70:**
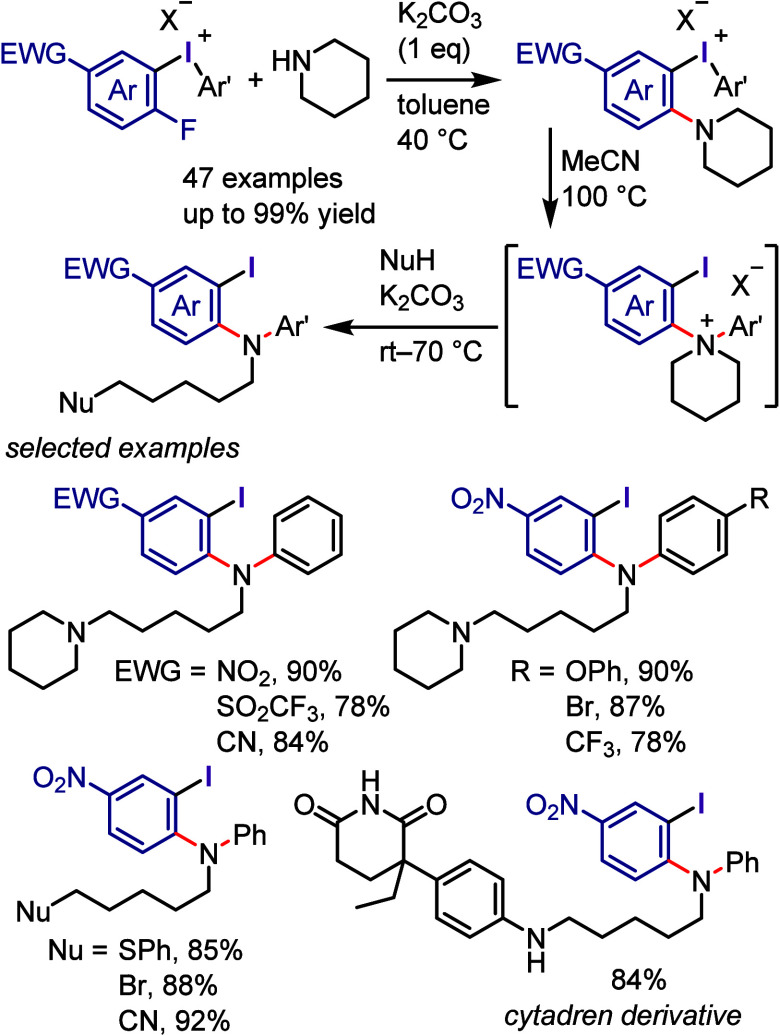
Atom-Economic *N*,*N*-Diarylation and *C*-Functionalization through Ring Opening of Cyclic Amines

After the atom-efficient diarylation of *N*-, *O*-, and *S*-nucleophiles with the two aryl groups of diaryliodonium salts, Olofsson et al. moved forward and designed a novel von Braun-type reaction for the diarylation/ring opening of cyclic amines to give highly functionalized diaryl amines (Scheme 70).^445^ An iodonium salt with a highly activated *ortho*-fluoroaryl group was required for the first step of the S_N_Ar reaction with an unstrained cyclic amine to give ortho-amino-substituted diaryliodonium salts in high yields. A one-pot sequential reaction of intramolecular arylation generated the diarylammonium intermediate, then nucleophilic substitution with a nucleophile afforded *C*- and *N*-functionalized diaryl amines. The reaction tolerated various iodonium salts and 5/6-membered cyclic amines in addition to a variety of amine, phenol, carboxylic acid, thiol, and halogen nucleophiles.

**Scheme 71:**
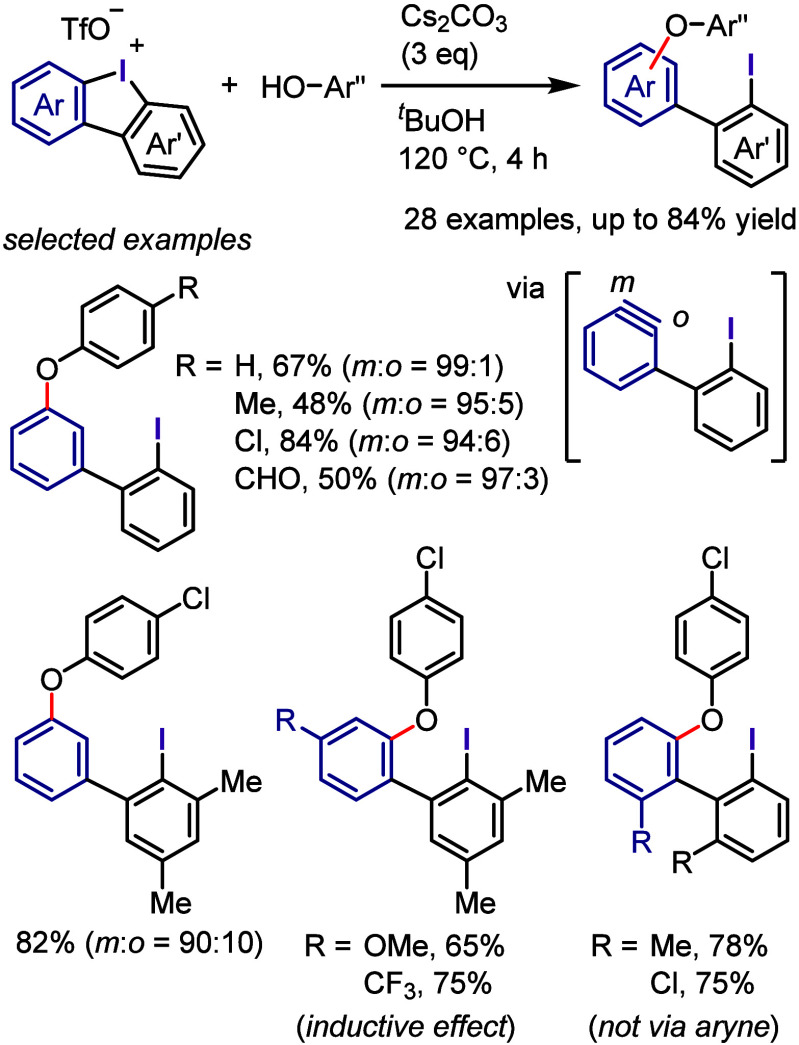
Selective *O*-Arylation of a Cyclic Iodonium Salt with Phenol

Cyclic diaryliodonium salts were used by Zhang, Wu, and co-workers as aryne precursors for *meta*-selective halogenation and *O*-arylation (Scheme 71).^446,447^ The scope of the phenol included diverse (hetero)aromatic phenols to give high selectivity of the related 2-iodo-3′-phenoxy-1,1′-biaryl products (*meta*:*ortho*, 90:1–99:1). The substituents on the aryl ring of the cyclic iodonium salt controlled the site of *O*-arylation process. 2-Substituted and 2,4-disubstituted iodonium salts afforded *meta*-functionalization of the less hindered aryl ring (*meta*:*ortho*, 75:25–99:1). In contrast, 3′-inductively electron withdrawing substituents (e.g., OMe, CF_3_) and 6,6′-disubstituted iodonium salts with high torsional strain gave *ortho*-derivatizations as the major products (*meta*:*ortho*, 1:99), involving the ligand coupling with the phenol not via aryne generation.

### 2.7 C–H Functionalization of (Hetero)arenes via In Situ Formation of Iodonium Salt

The generation of recyclable diaryliodonium salts by recovering the generated aryl iodide waste and reconverting it to the required diaryliodonium salts is not only a useful technique to overcome the disadvantages of the diaryliodonium salt but also could provide an attractive approach for C–H derivatization of (hetero)arenes without prefunctionalization. As there are different techniques used for recycling hypervalent iodines, in this section we discuss only the simplest approach for recycling diaryliodonium salt reagents.^448^

**Scheme 72:**
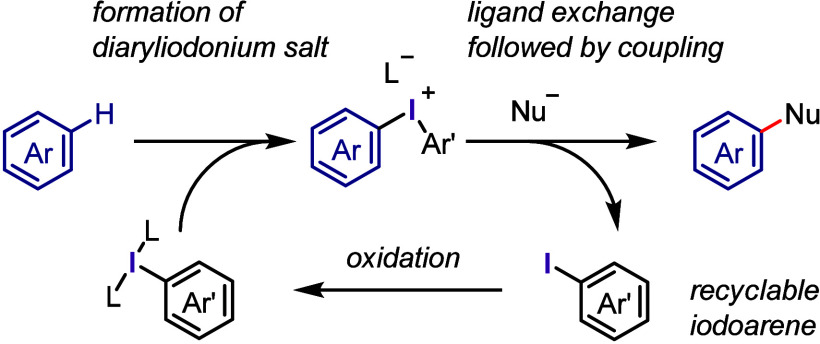
Sustainable C–H Arylation Strategy Mediated by Diaryliodonium Salt Formation

Transition metal-free C–H derivatization of inactivated arenes/heteroarenes is a highly challenging but desirable green and sustainable strategy.^449,450^ Diaryliodonium salt could provide a cascade approach to achieve this challenging transformation via a sequence of Ar^1^–I oxidative activation, coupling with Ar^2^–H to give Ar^1^(Ar^2^)IX, and finally cross-coupling with a nucleophile (Scheme 72). However, this iodonium salt-mediated strategy is also difficult due to the possibility of regio- and chemo-selective products during the second and third cross-coupling steps, respectively. Thus, finding suitable conditions to achieve this process in a regio- and chemo-selective manner is highly desirable as it will reproduce Ar^1^–I as a coproduct, which could be recycled, minimizing the cost and environmental impact of the process. Also, this hypothesis could help the hypervalent iodine community to develop the catalytic transformation and consequently overcome the main drawback of diaryliodonium salt transformations.^230^

**Scheme 73:**
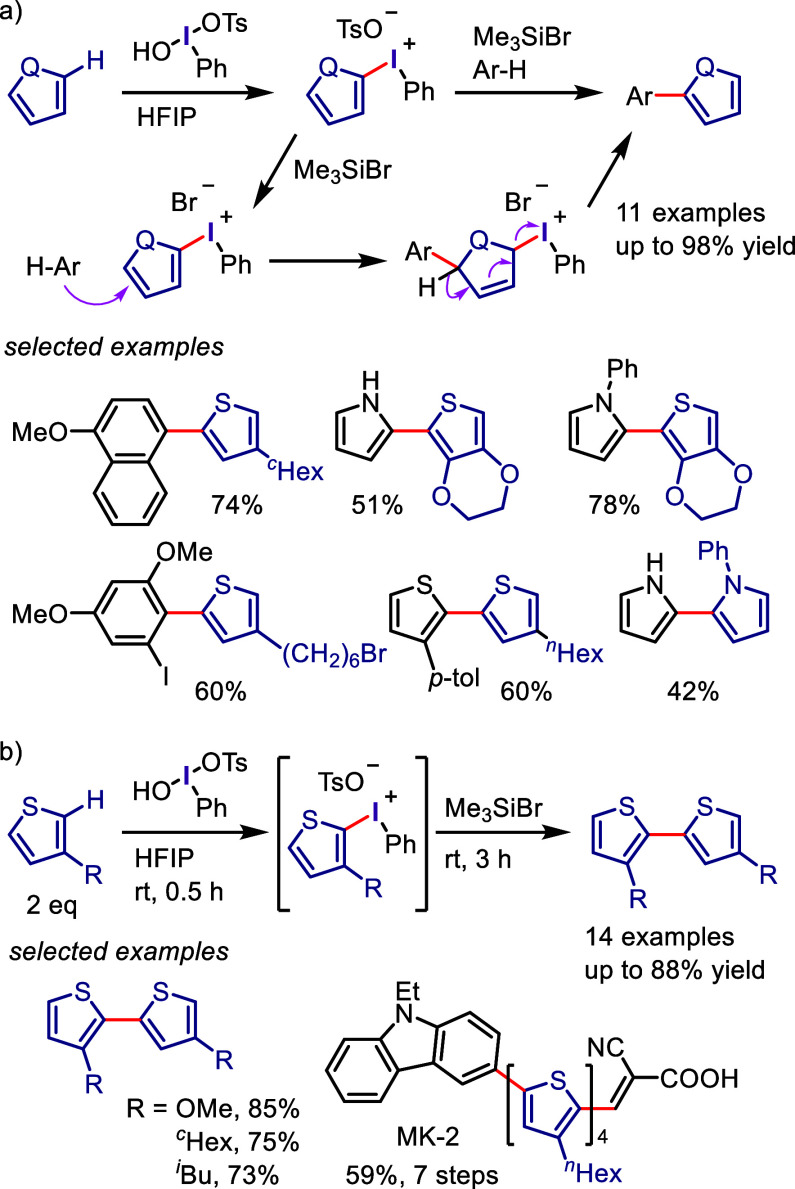
Oxidative Coupling of C–H Thiophene with C–H Arene Nucleophile Mediated by the Formation of Diaryliodonium Bromide

Kita’s group discovered the first oxidative cross-coupling strategy based on in situ generation of diaryliodonium tosylate from the condensation of [hydroxy(tosyloxy)iodo]benzene (Koser’s reagent) with a heteroaromatic compound followed by ligand exchange of the tosylate with the bromide by the addition of TMSBr; this would give aryl(heteroaryl)iodonium bromide as the key reaction intermediate as there is no reaction with the tosylate salt (Scheme 73a).^100^ Cross-coupling of the generated iodonium bromide with an aromatic nucleophile, via the formal hydroarylation, produced the related biaryl with an excellent degree of chemo- and regio-selectivity. The presence of hexafluoroisopropanol (HFIP)^340^ as a solvent and trimethylsilyl bromide (TMSBr) as an activator appeared crucial. This unique oxidative cross-coupling protocol can take place in a stepwise or one-pot sequential reaction and showed applicability for the coupling of thiophene and pyrrole with electron-rich aromatic nucleophiles, such as methoxy-substituted arenes, pyrroles, and thiophenes.^100,451,452^ Furthermore, this process successfully cross-coupled (hetero)arene C–H with azole N–H, and achieved the construction of C–N biaryls.^453^ Further extension of this unique oxidative coupling achieved the synthesis of unsymmetrical head-to-tail (H-T) bithiophenes as the sole regioisomeric product (Scheme 73b).^98^ Under the previously optimized conditions, 3-alkyl- and 3-alkoxy-thiophenes participated as both the iodonium salt component and aromatic nucleophile. The exclusive H-T connection of the bithiophenes was attributed to the high electron-richness of the 2-position of the thiophene substrates that facilitated, in part, the highly regioselective synthesis of the phenyl(thienyl)iodonium salt. The other unreacted part of the thiophene substrate attacked, through its reactive 2-position, the free 5-position on the thienyl moiety of the iodonium salt to give the unsymmetrical thiophene dimer. This methodology was further expanded^99^ and successfully applied to the concise synthesis of the efficient photovoltaic donor–acceptor oligothiophene MK-2, a high-performance organic dye for application in solar cells.^454^

**Scheme 74:**
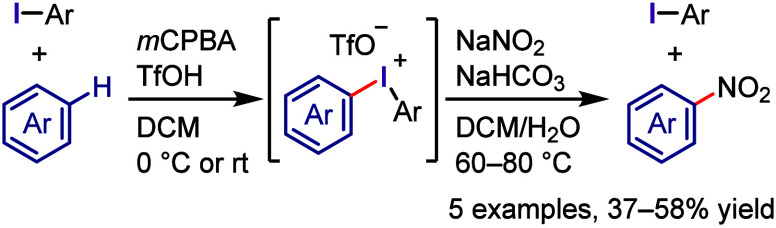
C–H Functionalization of Arene Mediated by In Situ-Generated Diaryliodonium Salt

Olofsson and co-workers designed a new strategy for C–H nitration of arenes via a one-pot sequential oxidation of Ar^2^–I, coupling with Ar^1^–H and formation of diaryliodonium salt, and finally nitration with NaNO_2_ to produce the functionalized nitroarene (Scheme 74).^230^ However, the scope of this C–H derivatization was limited to symmetrical diaryliodonium salts and arene substrates with halogen- and alkyl-substituents in addition to the unsubstituted benzene.

**Scheme 75:**
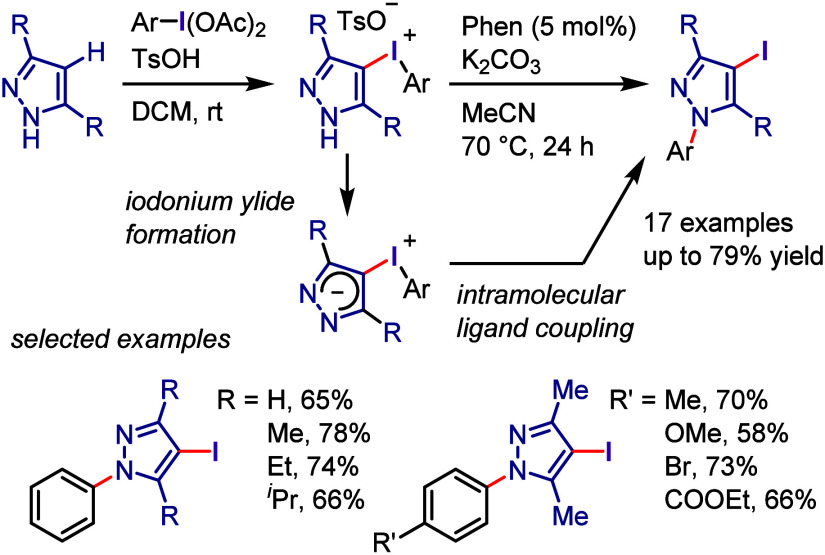
1,4-Difunctionalization of Pyrazole via In Situ Generation of Iodonium Salt

Iodoarylation of heterocycles through the in situ generation of iodonium salt/ylide was developed by Cheng et al. as an efficient one-pot two-step strategy for highly economical and useful transformations.^455,456^ For example, the reaction of *NH*-pyrazole with aryliodine diacetate gave *N*-aryl-4-iodopyrazole via a sequence of in situ generation of pyrazolyl(aryl)iodonium salt under acidic conditions followed by intermolecular regioselective *N*-arylation (Scheme 75).^457^ In this reaction, the iodine atoms were incorporated in the final product.

**Scheme 76:**
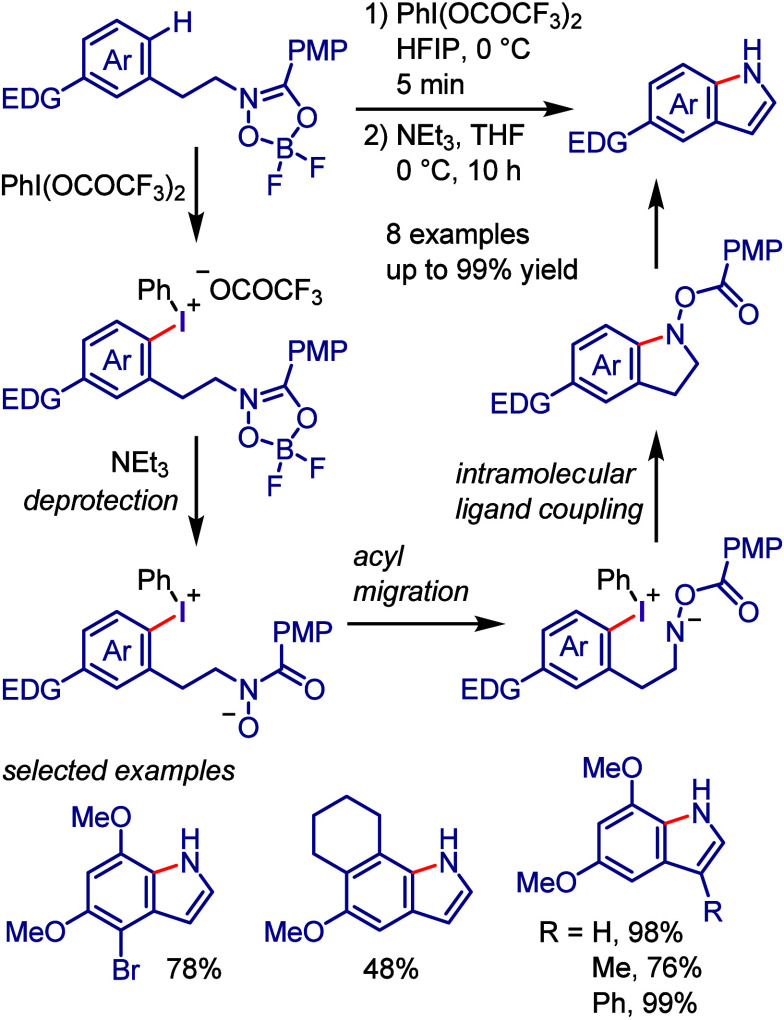
Synthesis of Indoles via In Situ Generation of Iodonium Salt and Intramolecular C–N Bond Formation

Ishikawa et al. synthesized an electron-rich indole using a one-pot reaction of electron-rich aromatic-tethered boron-masking *N*-hydroxylamides with PIFA, followed by treatment with triethylamine (Scheme 76).^458^ The reaction mechanism involved the in situ generation of diaryliodonium salt, which was then treated with triethylamine to produce the indole product after a sequence of deborylation, acyl migration, intramolecular cyclization, and desorption of carboxylic acid before tautomerization. The use of a boron-masked scaffold, together with the careful design of electron-rich aryl substrates, was essential for the unique regioselective diaryliodonium salt formation.

**Scheme 77:**
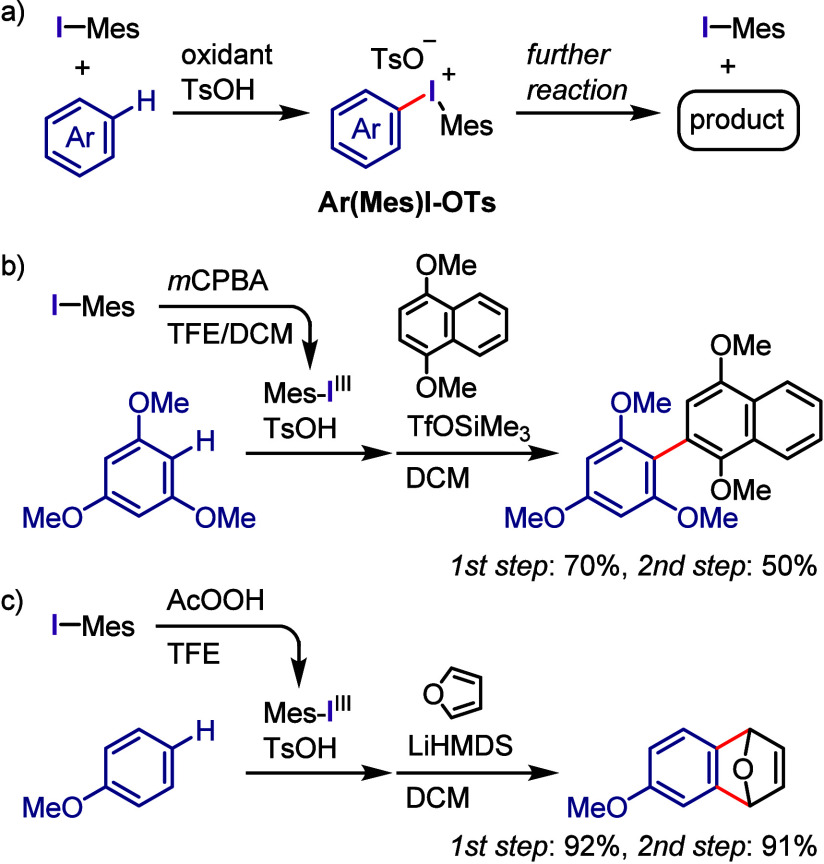
Recyclable Diaryliodonium Salt-Mediated C–H Functionalization of Arenes

Dohi and co-workers used iodomesitylene as a recyclable iodoarene for C–H functionalization of arenes through the synthesis of aryl(Mes)iodonium salts (**Ar(Mes)I-X**) via a stepwise or one-pot strategy (Scheme 77a).^141^ The reaction tolerated a broad range of functional groups and worked well with arene and heteroarene nucleophiles to produce the related iodonium salts with excellent regioselectivity. These aryl(mesityl)iodonium salts were screened in transition metal-free transformations such as SET-induced coupling of 2,4,6-trimethoxyphenyl(mesityl)iodonium tosylate with 1,4-dimethoxynaphthalene and production of the corresponding biaryl with exclusive chemoselective transfer of the trimethoxyphenyl moiety from the iodonium salt (Scheme 77b). Aryne generation from the 4-methoxyphenyl(mesityl)iodonium tosylate in the presence of LiHMDS and [3 + 2] cycloaddition with furan gave a bridged cycle product (Scheme 77c). Recently, the Stuart group designed a one-pot formal dehydroboration of aryl boron reagents via in situ generation of aryl(Mes)iodonium salt and aryne intermediates.^459^ This sequence was applied to the formal synthesis of the investigational Aurora Kinase inhibitor PF-03814735.

**Scheme 78:**
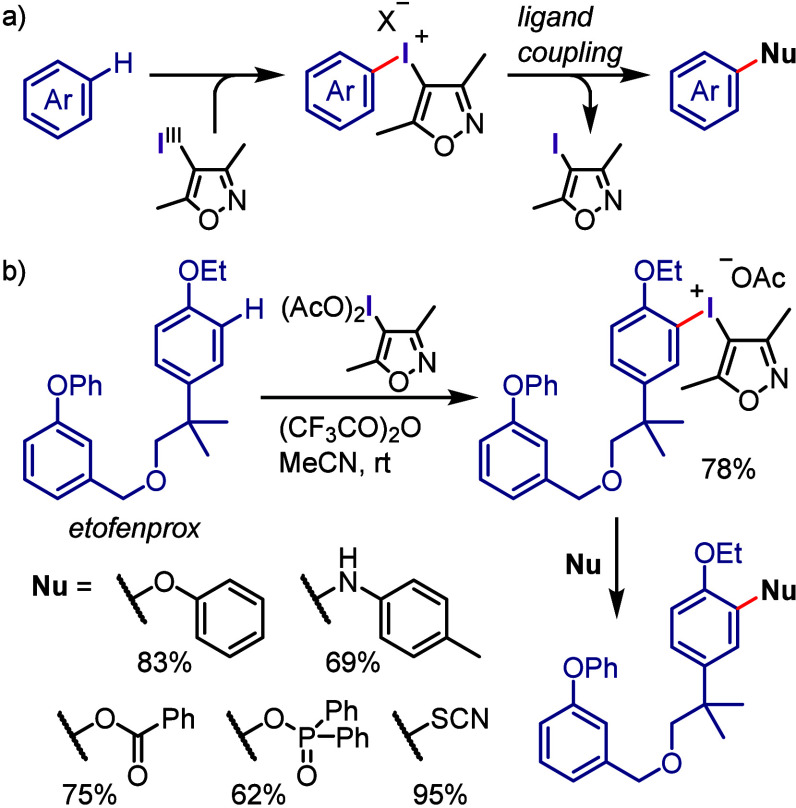
C–H Derivatization of (Hetero)arenes through the Formation of Diaryliodonium Salt Intermediates

Zhao and co-workers developed a novel two-step strategy for C–H functionalization of arenes mediated by in situ generated isoxazole diaryliodonium salt (Scheme 78a).^460^ The incorporation of 4-iodo-3,5-dimethylisoxazole (DMIX-I) as a recyclable iodoarene for the synthesis of aryl(DMIX)iodonium salts provided superior reactivity and selectivity over the well-established aryl(TMP)iodonium salts. This discovery provided an alternative and more practical approach for the preparation of diaryliodonium salts by introducing the target C–H arene with a high site selectivity at a late-stage after the synthesis of DMIX-I(OH)OTs and DMIX-I(OAc)_2_ intermediates. The scope of aryl(DMIX)iodonium salt was extended to various arenes including a bioactive compound with regioselective C–H derivatization. Ligand coupling of the aryl(DMIX)iodonium salt by a broad range of nucleophiles including C-, N-, O-, and S-nucleophiles afforded the related functionalized arenes with high selectivity (Scheme 78b). In addition to the two-step strategy, a one-pot procedure for the regioselective C–H λ^3^-iodanation of arenes followed by chemospecific arylation of a nucleophile was also possible.

**Scheme 79:**
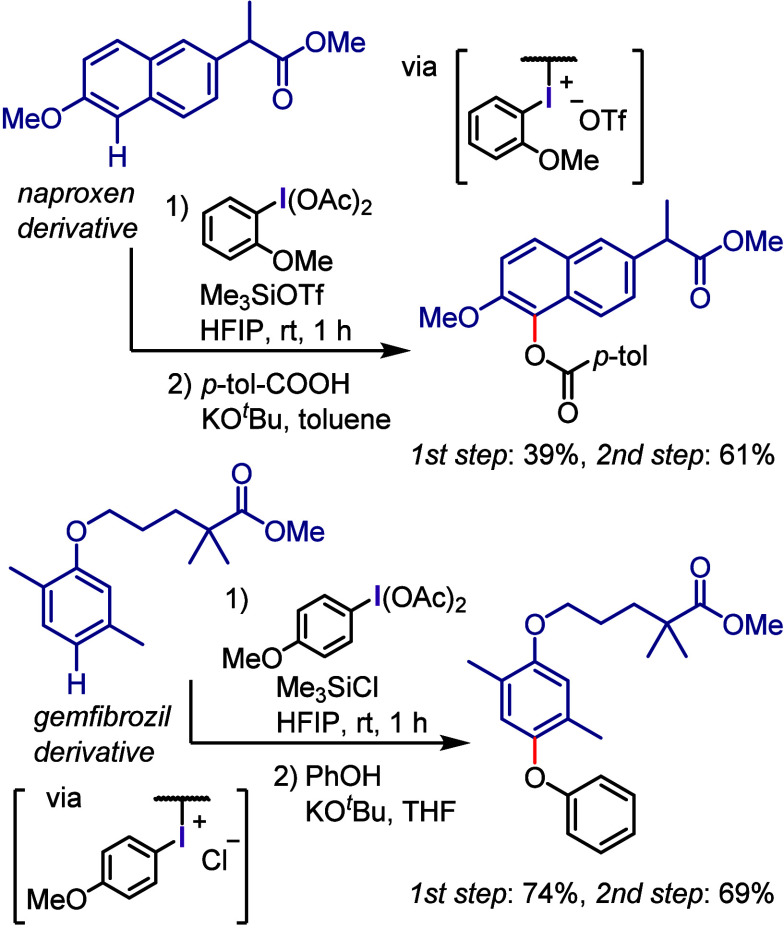
C–H Functionalization of Drugs Mediated by Iodonium Salt

The usefulness of the C–H functionalization of arenes mediated by diaryliodonium salt was assessed in drug design and discovery through C–H functionalization of naproxen and gemfibrozil drugs.^461,462^ Coupling of a C–H drug substrate, such as naproxen or a gemfibrozil derivative, with ArI(OH)OTs or ArI(OAc)_2_ in the presence of fluorinated solvent afforded the corresponding diaryliodonium salt (Scheme 79). These iodonium salts are suitable for diverse functionalization with excellent chemoselectivity under transition metal- free conditions to provide a library of modified drugs.

## 3 Base-Promoted Ar–I Bond Dissociation

**Scheme 80:**
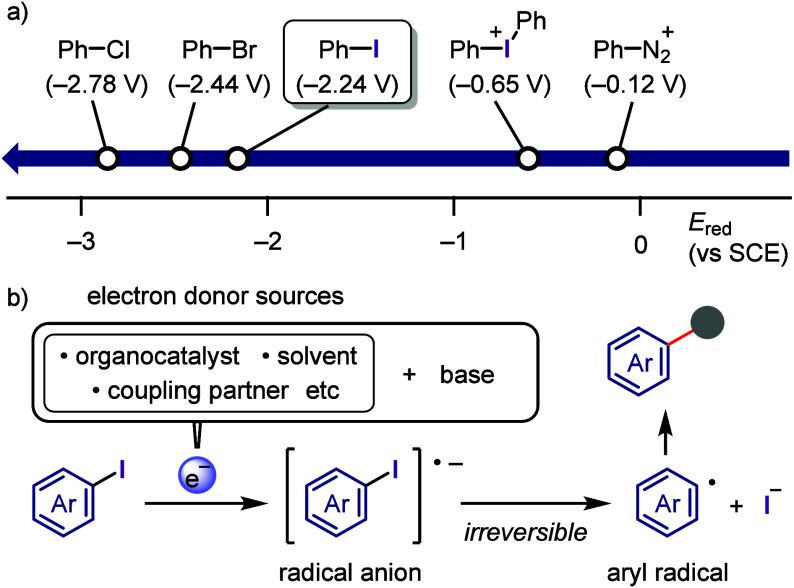
(a) Reduction Potentials of Arylating Reagents. (b) Aryl Radical Generation via Single-Electron-Transfer (SET) Process

Transition metal-free C- and heteroatom-arylations via direct cross-coupling with aryl halides provide atom- and step-economical processes for the syntheses of functionalized arene skeletons.^463−466^ In addition to diaryliodonium salts and their unique hypervalent bond (*E*_red_ ≈ −0.65 V vs SCE),^467,468^ which are highly efficient arylating reagents under transition metal-free benign conditions, aryl halides are attractive arylating reagents because they can be used directly without further modification and are commercially available, easily synthesized, inexpensive, and bench-stable starting materials. The BHAS or organocatalytic C–H arylation reaction has recently garnered significant interest owing to the possibility of the construction of diverse skeletons via inter- and intramolecular BHAS reactions under mild reaction conditions without requiring sophisticated transition metals and ligands.^377,469,470^ In these reactions, aryl halides are effective aryl radical precursors in the arylation of a broad range of arenes under base-catalyzed conditions. Cleaving the aryl–halogen bond without external thermal, photo- or electrochemical stimuli is challenging mainly because of a higher energy barrier, that results from a high reduction potential (Scheme 80a).^471,472^ Among all aryl halides, iodoarenes are characterized by their reduction potential, bond dissociation energy, polarizability, and different Ar–X bond cleavage mechanism,^473^ which enable the transition metal-free arylation process to be performed under benign conditions with a broad substrate scope and functional group tolerance. For base-promoted Ar–I bond dissociation, the transformation requires either a strong base or combination of base and ligand (also named promoter, activator, or catalyst). The general mechanism of this strategy is based on the generation (in situ) of an OEDs with sufficient redox potential to initiate a single-electron transfer (SET) process to the aryl halide and affords a reactive aryl radical species for arylation reactions (Scheme 80b). Notably, single electron donors with potentials as low as −1.3 V versus Fc/Fc^+^ (−0.8 V vs SCE) still have potency for activating aryl iodide substrates. The high energy barrier of the SET process (uphill by 0.6–0.8 V) can be overcome by the irreversible conversion of [Ar–I]^•–^ to Ar^•^ and I^–^. The SET process is a technique that can be used to access previously inaccessible aryl halides or accomplish chemical transformations under relatively mild conditions; furthermore, inexpensive and commercially available starting substrates can be used.^474−491^ This transformation may be recognized as an “electron-catalyzed reaction”,^466,492,493^ which played a crucial role in modern radical chemistry and delivered a promising route to obtain radical intermediates.

**Scheme 81:**
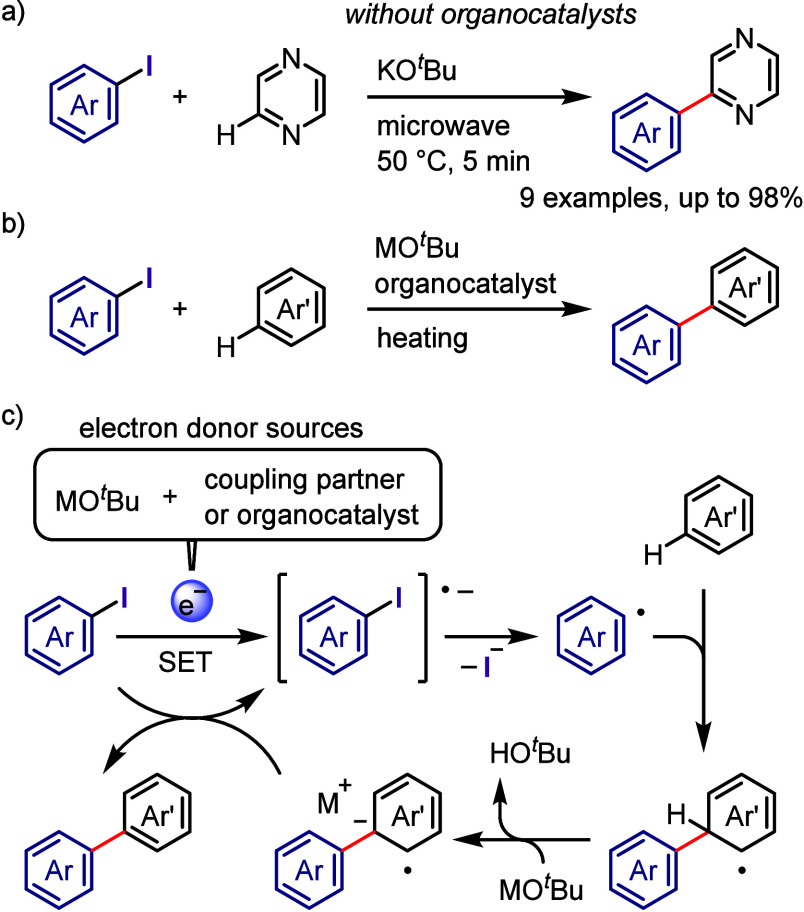
Transition Metal-Free Cross-Coupling of Aryl Halide with Arenes

The pioneering work of Itami et al. emphasized the importance of using KO^*t*^Bu for the arylation of azines (i.e., pyrazine) with aryl iodides (Scheme 81a).^494,495^ The efficiency of this type of transformation was maximized by the addition of a catalytic amount of organic compound as an additive,^469^ which coordinated MO^*t*^Bu (M = Na, K) and facilitated the initiation step of the chain process (Scheme 81b). A plausible mechanism for the radical reaction was proposed by Studer and Curran and involves initiation and propagation processes (Scheme 81c).^492^ The initiation process was clarified by Murphy et al.^469^ to include the reaction of MO^*t*^Bu with an organic additive or in situ generation of an OED as a promoter of the initiation step via the single electron transfer (SET) reduction of the aryl halide into the aryl halide radical anion, which subsequently dissociates to the aryl radical and halide anion. The generated aryl radical reacts with aromatic compounds to produce cyclohexadienyl radical species, which are subsequently deprotonated by a base to generate the keystone biaryl radical anion, which serves as a powerful reducing agent (reductant upconversion^470^). Propagation of the process via a SET to the aryl halide produces the biaryl product and regenerates another radical anion to continue the radical chain process.

Murphy et al. performed extensive experimental and theoretical studies to propose a unified mechanism in which various organocatalysts served as precursors of the electron donor species. Thus, the organocatalysts reacted first with *tert*-butoxide salt to generate the electron donors in situ; the electron donors transfer an electron to halobenzene and form the aryl radicals.^469,496−503^

### 3.1 Organocatalytic Activation of Ar–I Bond

**Scheme 82:**
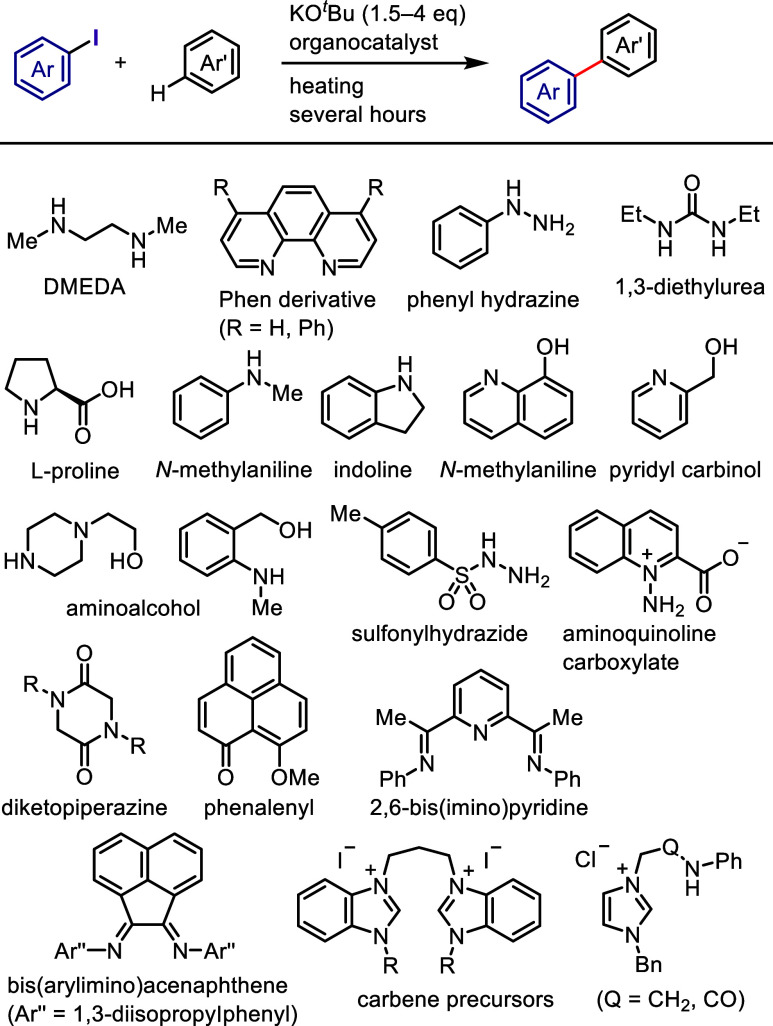
Transition Metal-Free Organocatalytic Arylation of Unactivated C–H Arenes with Aryl Iodide

Activation of the reaction between (hetero)aryl halides and unactivated (hetero)arenes using a combination of *tert*-butoxide salt and organocatalyst is the most significant and practical alternative to using transition metals from an environmental, economic, and safety perspective.^382,493,504^ After the first transition metal-free biaryl coupling was discovered by Itami et al.,^494,495^ various additive were found to be crucial for coupling of aryl halides to nonactivated arenes (Scheme 82). In 2010, three research groups described organocatalysts as being efficient activators for the C–H arylation of unactivated arenes with haloarenes in the presence of *tert*-butoxide salts as no reactions occur in the absence of organocatalysts. Kwong and Lei^505−507^ reported dimethyl ethylenediamine (DMEDA) and the groups led by Shirakawa and Hayashi^508^ and Shi^509^ extolled the importance of 1,10-phenanthroline (Phen) derivatives as promoters of the C–H arylation of arenes. 1,10-Phenanthrolines became popular organocatalysts for the activation of aryl halides to generate aryl radicals which cross couple with (hetero)arene via inter- and intramolecular approaches.^510−513^ Interestingly, commercially available and inexpensive compounds, such as phenyl hydrazine,^514,515^ amino acid (l-proline),^516^ alcohol (^*n*^BuOH),^517,518^ and urea (1,3-diethylurea)^519^ were found to be efficient catalysts for activating the cross-coupling between C–H (hetero)arene and (hetero)aryl iodide. Aniline derivatives (*N*-methylaniline^520^ and indoline^521^) and pyridine derivatives (8-hydroxyquinoline^522^ and 2-pyridyl carbinol^496,523^) were also used as catalysts. Similar to the previously known behavior of the organocatalyst/KO^*t*^Bu combination, aminoalcohols,^524,525^ sulfonylhydrazide,^526^ quinoline-1-amino-2-carboxylic acid,^527^ diketopiperazine,^528−531^ 9-methylamino-phenalen-1-one (phenalenyl ligand),^532^ 2,6-*bis*(imino)pyridine,^533^ and *bis*(arylimino)acenaphthene^534^ were used catalytically in the presence of KO^*t*^Bu to promote the C(sp^2^)-H arylation of unactivated arene with aryl halide to afford the corresponding biaryls. Carbene precursors are also efficient organocatalysts for the C–H arylation of arenes in the presence of a strong base.^497,535,536^ Porphyrin,^537^ macrocyclic aromatic pyridone pentamer,^538^ and foldamer-based pyridone dimer,^539^ in addition to other promoters,^540−546^ are potent organocatalysts for activating the cross-coupling between unfunctionalized C–H arenes and aryl iodide. In addition to the previously mentioned homogeneous organocatalysts, heterogeneous promoters such as graphene oxide,^547^*N*-doped porous carbon nanotubes,^548^ and glucose-derived carbonaceous^549^ and porous phenanthroline-based polymers^550^ displayed a high catalytic activity and recyclability for the direct C–H arylation of unactivated arenes with aryl iodide.

**Scheme 83:**
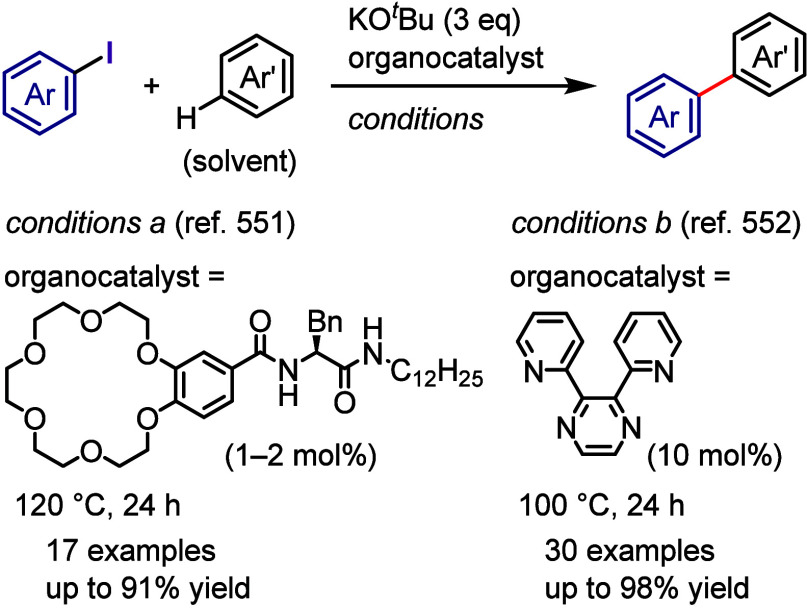
Crown Ether-Derived Supramolecular Organocatalyst to Promote the C–H Arylation of Arenes with Aryl Halides: 2,3-Di(pyridine-2-yl)pyrazine Promoted Haloarene for the Arylation of Benzene

Zhou and Zeng et al. designed crown ether-containing monopeptides as organocatalysts for activating the direct C–H arylation of (hetero)arene by aryl iodides in the presence of KO^*t*^Bu (Scheme 83, conditions a).^551^ Association of the organocatalyst through noncovalent interactions enables multiple crown ether moieties to efficiently bind K^+^ ions and subsequently allows the ^*t*^BuO^–^ anion to facilitate the activation of aryl iodides via the SET protocol. The size of the crown ether and hydrophobicity of the side chain are crucial for the high performance and lower catalytic loading of the organocatalyst. The catalyst activates only aryl iodides as haloarenes. From among the *N*,*N*-/*O*,*O*-/*N*,*O*-bidentate organocatalysts, Chaudhary et al. introduced 2,3-di(pyridine-2-yl)pyrazine as a new organocatalyst for the direct cross-coupling of haloarene with unactivated benzene (Scheme 83, conditions b).^552^ Diverse aryl halides and heteroaryl halides were used to afford the corresponding biaryls.

**Scheme 84:**
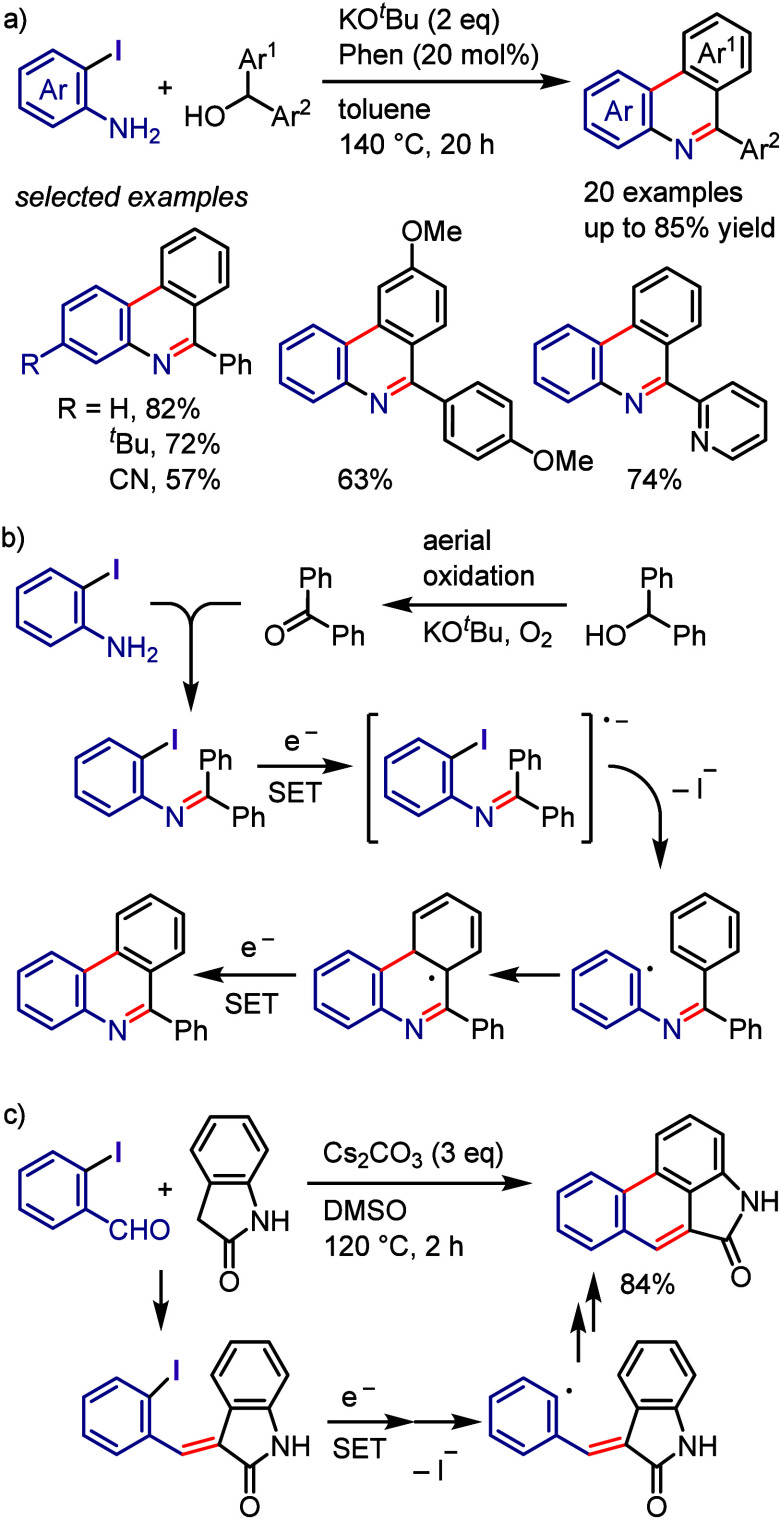
Cascade Synthesis of Phenanthridine Derivatives: Indoin-2-one as Substrate and Promoter for the Synthesis of Tetra-Fused Skeletons

The cascade reaction of benzhydrol and 2-iodoaniline in the presence of KO^*t*^Bu/1,10-phenathroline, via a controlled sequence of the oxidation of an alcohol to a ketone followed by the direct condensation with the aniline-NH_2_ group and radical annulation, provided a direct process for the synthesis of phenanthridine derivatives (Scheme 84a).^553^ Under the reaction conditions, KO^*t*^Bu played an important role in the aerobic oxidation of the alcohol and the homolytic aromatic substitution (HAS). The scope of 2-iodoaniline extended to include electron-rich/deficient and biaryl substrates. Substituted benzhydrols afforded regioisomeric mixtures based on the mode of action together with the annulation with both aromatic rings. The proposed mechanism involves the reduction of the in situ formed imine by the generated super electron donor species (Scheme 84b). Thus-generated aryl radical cyclized to afford thus-generated aryl radical which cyclized to afford the tricyclic radical, which converted to the final product via the SET process. Wu et al. developed a cascade reaction of 2-halobenzaldehydes and indolin-2-ones in the presence of Cs_2_CO_3_ for the synthesis of naphtho[3,2,1-*cd*]indol-5(4*H*)-ones (Scheme 84c).^554^ Indolin-2-ones served as the substrates and electron donor precursors. The proposed mechanism initiated with indolin-2-ones by Cs_2_CO_3_ to generate the electron donor enolate, which reacted with the aldehyde to form the condensed intermediate. The SET from the electron donor enolate to the condensed intermediate provided the aryl radical, which underwent 6-*endo* cyclization to produce the final product.

**Scheme 85:**
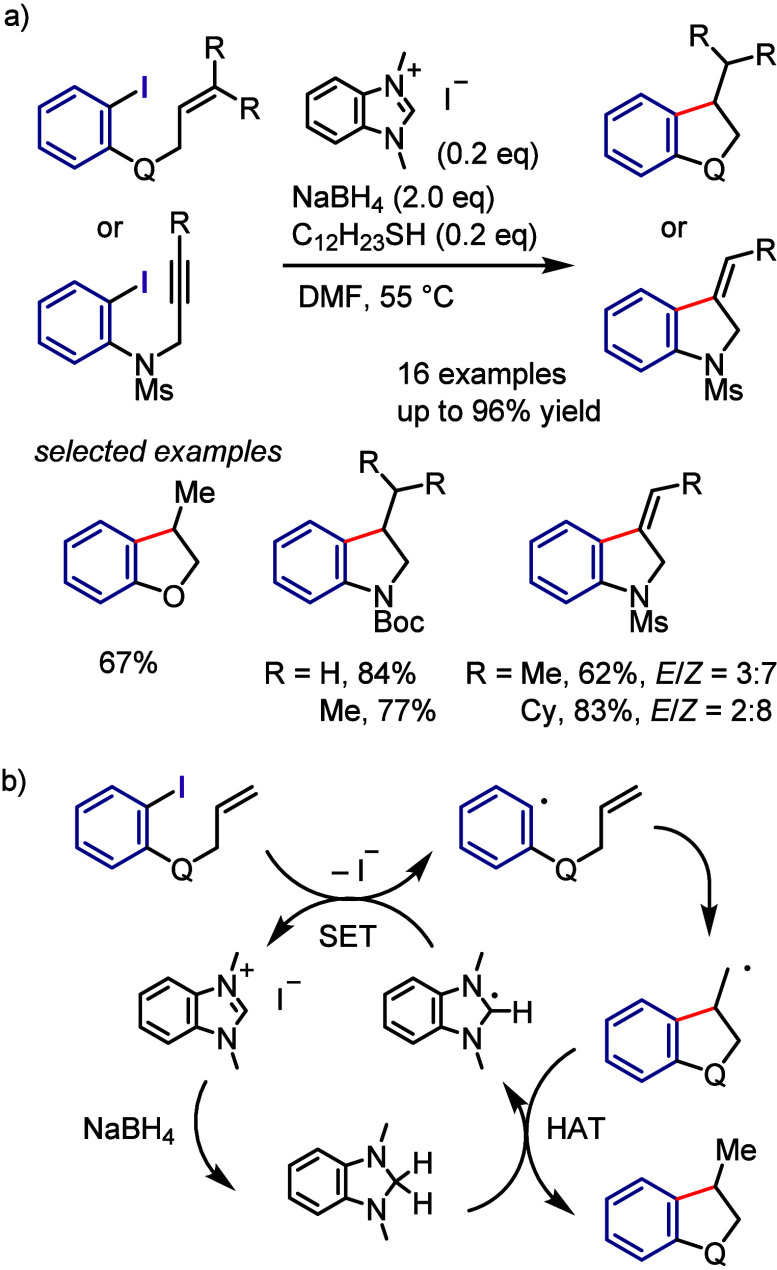
Catalytic Reductive Radical Cyclization Reactions with Super Electron Donors

Murphy et al. have long been involved in the development of neutral organic super electron donors for the activation of various substrates including aryl halides.^555−564^ However, these super electron donors are used in stoichiometric amounts or in excess to promote the reactions and this factor impedes the use of these reagents in radical chain reactions. Recently, the same group solved this problem by altering the nature of the super electron donors.^565^ Thus, using benzimidazolium salt led to the catalytic radical cyclization of *N*-allyl- and propargyl-2-iodoaniline through 5-*exo-trig* and 5-*exo-dig* cyclization and the cyclized indoline and indolenine products (Scheme 85a) were obtained. Computational and experimental studies revealed that the generated benzimidazole radical is among the most potent electron donor reducing agents (*E*_ox_ = −1.86 V vs SCE). The proposed mechanism for the reaction involves the reduction of the aryl iodide substrate by the benzimidazole radical via the SET process to generate an aryl radical, which undergoes 5-*exo-trig* cyclization to afford a radical intermediate, followed by the hydrogen atom transfer (HAT) process to furnish the final product (Scheme 85b). Similarly, Kondo et al. developed an intermolecular cross-coupling of aryl halide with styrene (1,1-diarylethelene) in the presence of NaH/1,10-phenanthroline, which generated reduced anion species as super electron donors and H-sources.^566^ The hydroarylation of styrene proceeds in an anti-Markovnikov fashion to afford 1,1,3-triarylethane.

**Scheme 86:**
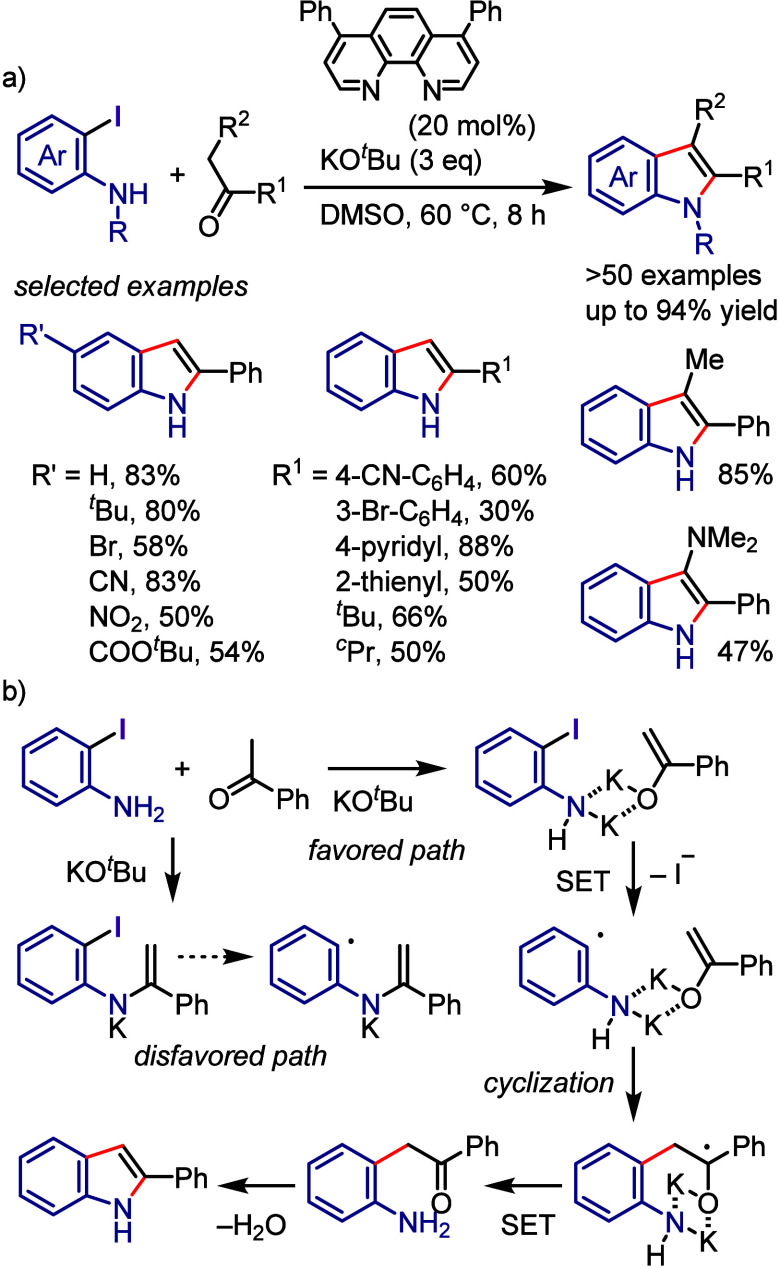
Synthesis of Indoles *via* Radical-Enolate Coupling

Cheong and Lee reported the combination of 2-iodoaniline derivatives and ketones in the presence of KO^*t*^Bu and 4,7-diphenylphenanthroline for the construction of diverse indoles under mild conditions (Scheme 86a).^567^ DFT calculations supported the mechanism shown in Scheme 86b. Deprotonation of both substrates followed by the SET process from the electron donor generated the aryl radical complex. Radical-enolate coupling via 7-*endo*-*trig*, followed by the SET process, protonation, and condensation afforded the final indole product.

**Scheme 87:**
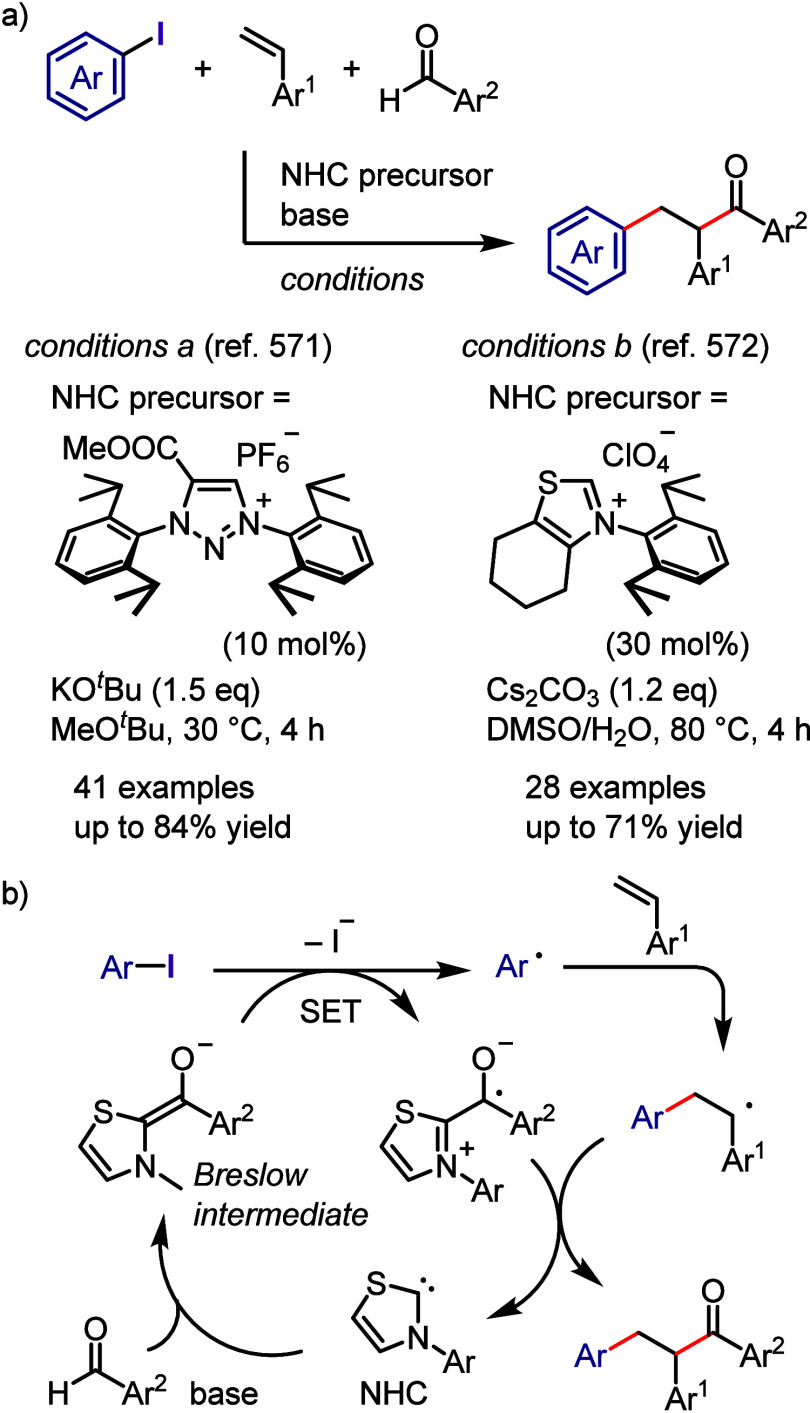
Mesoionic Carbene Catalyzed Acylarylation of Alkenes

The Breslow intermediate derived from *N*-heterocyclic carbene organocatalysis enabled various incredible transformations and successfully radical NHCs were applied to the HAT strategy for the direct activation of aliphatic C–H bonds.^568−570^ Bertrand et al. demonstrated that the reaction of aryl iodide, aryl aldehyde, and styrene under mesoionic carbene-catalyzed conditions afforded the corresponding acylarylation products (Scheme 87a, conditions a).^571^ Ohmiya et al. used thiazolium salt as an NHC precursor for the three-component arylacylation of styrenes (Scheme 87a, conditions b).^572^ The catalytic cycle began with the *in situ* formation of the Breslow intermediate (−1.93 V vs SCE), which donates a single electron to aryl iodide to generate an aryl radical and the Breslow intermediate-derived ketyl radical (Scheme 87b). After the addition of the aryl radical to styrene to generate the reactive alkyl radical, intermolecular acyl transfer from the ketyl radical intermediate proceeds to afford the final product and regenerates the NHC catalyst.

**Scheme 88:**
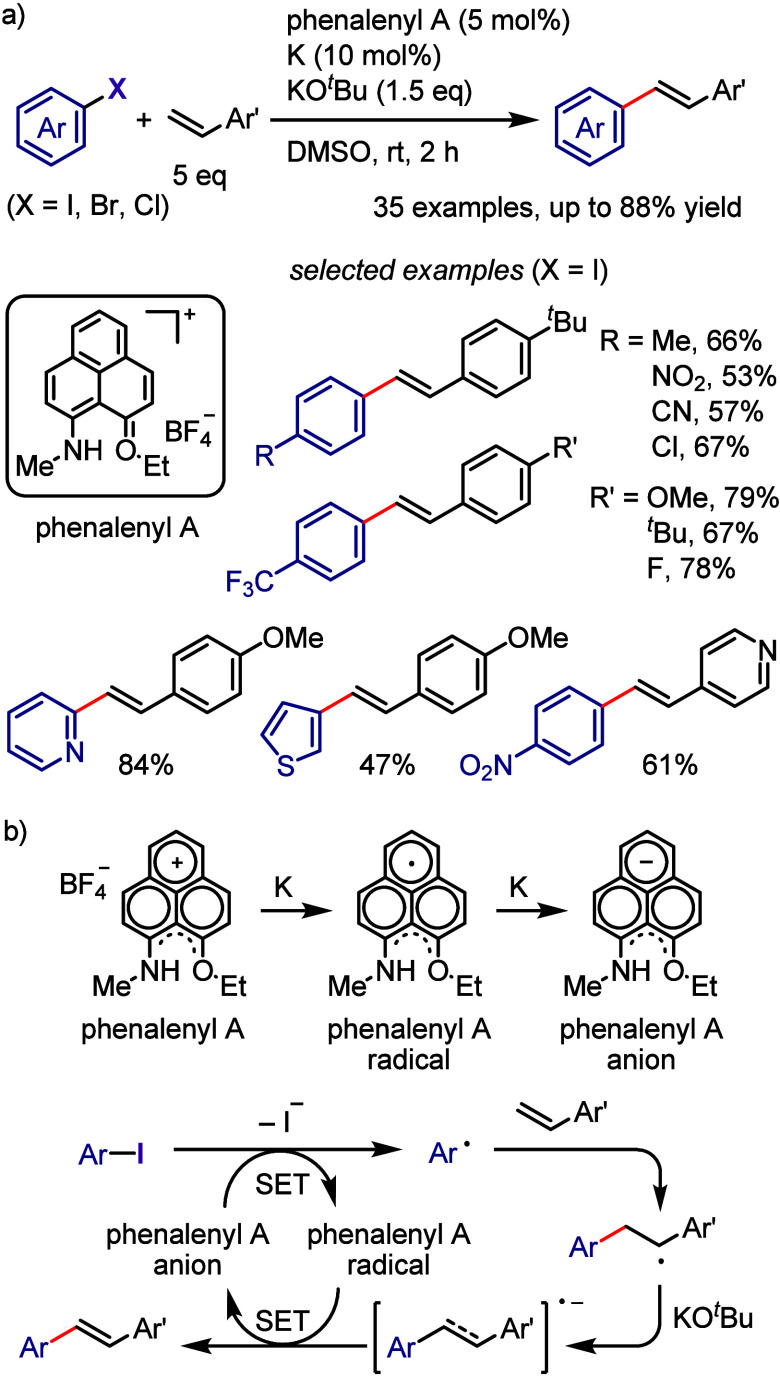
Phenalenyl-Anion Catalyzed Heck-Type Cross-Coupling of Aryl Iodide with Styrene

The unique characteristics of phenalenyl-based molecules and in particular its more reactive doubly reduced anionic redox state prompted Mandal et al. to design efficient catalytic processes for the activation of aryl iodide during Mizoroki-Heck-type cross-coupling with styrenes, C–H arylation of arenes, and borylation under solvent-free ball-milling conditions.^502,503,573,574^ Metallic potassium was used to tune the redox state of the catalyst and generate the reactive anion species (*E*_red_ = −1.82 in acetonitrile), and was key to the activation of the Mizoroki-Heck-type cross-coupling between aryl iodide and styrene (Scheme 88a).^574^ The proposed catalytic cycle involves the consecutive injection of two electrons to the cationic phenalenyl to generate the anion, which reduced aryl iodide via the SET process to afford the aryl radical (Scheme 88b). The aryl radical was trapped by styrene and after deprotonation the radical anion intermediate was generated; the radical anion intermediate was reduced via the SET process to form the stilbene product regenerate the catalyst.

**Scheme 89:**
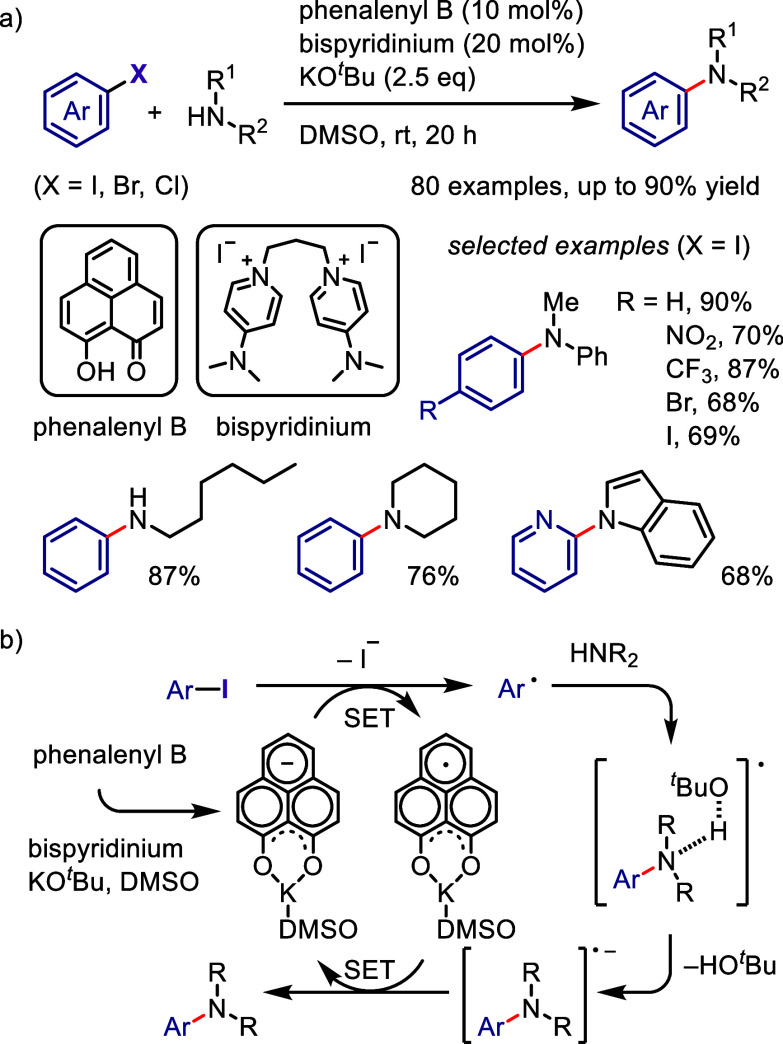
Reduced Phenalenyl-Catalyzed Buchwald-Hartwig-Type Cross-Coupling of Aryl Halide with Amine

The same group developed a unique Buchwald–Hartwig-type cross-coupling of aryl halides with amine using a phenalenyl-based organocatalyst (Scheme 89a).^575^ The bispyridinium salt and phenalenyl ligand generate the phenalenyl anion, which is responsible for performing this radical-mediated C–N cross-coupling at room temperature. Interestingly, (hetero)aryl dihalides and primary aliphatic/aromatic amines reacted with high selectivity to afford the related monofunctionalized products. The generated aryl radical binds with the amine via a concerted deprotonation and C–N bond formation to generate the radical anion, which is oxidized by the phenalenyl radical to afford the final aryl amine and the phenalenyl anion.

**Scheme 90:**
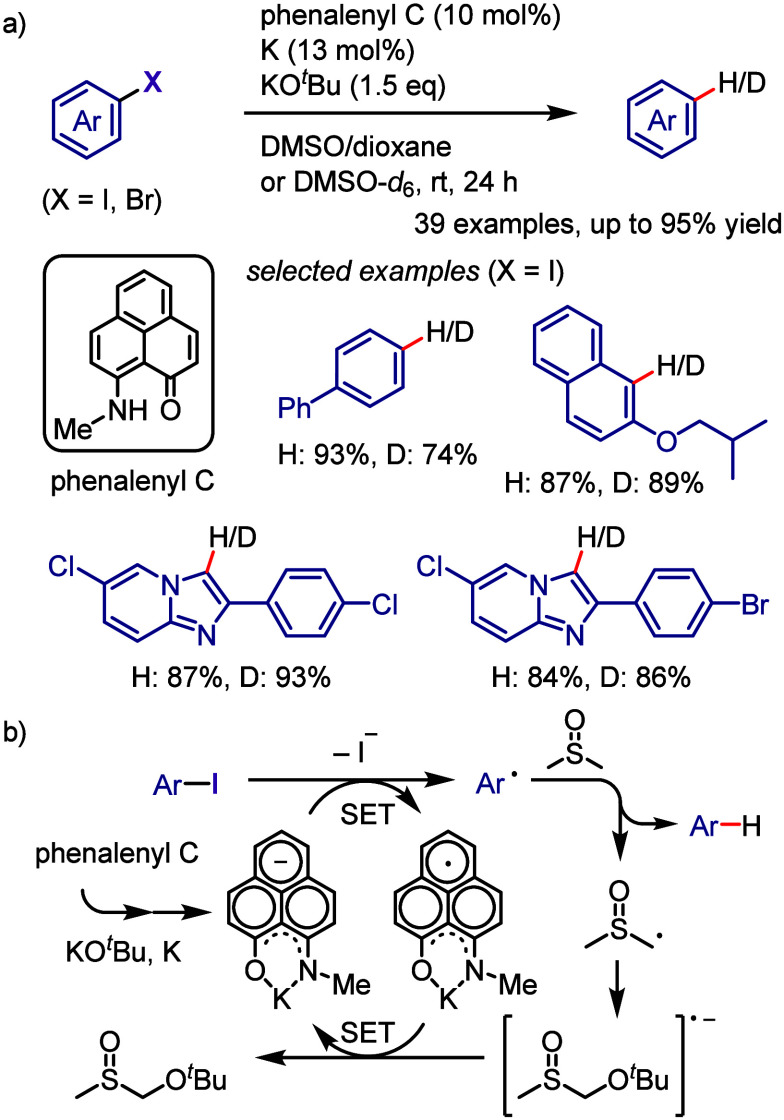
Reduced Phenylenyl Catalyzed Transformation of Ar-X to Ar-H/D

Furthermore, phenalenyl C was used for the catalytic hydrodehalogenation and dehalogenative deuteration of a broad array of aryl/heteroaryl halides under ambient conditions (Scheme 90a).^502,503,572,576^ The combination of the precatalyst, catalytic metallic potassium, and KO^*t*^Bu is essential to enable the desired transformation in high yields. The doubly reduced phenalenyl anion (*E*^2^_1/2_ = −1.88 V) undergoes the thermodynamically favorable SET with aryl iodide to generate the aryl radical, which accepts a hydrogen atom from DMSO to afford the dehalogenated products and the DMSO-radical (Scheme 90a). The thus-generated radical reacts with KO^*t*^Bu to generate the radical anion, which serves as the reductant to regenerate phenalenyl anion.

**Scheme 91:**
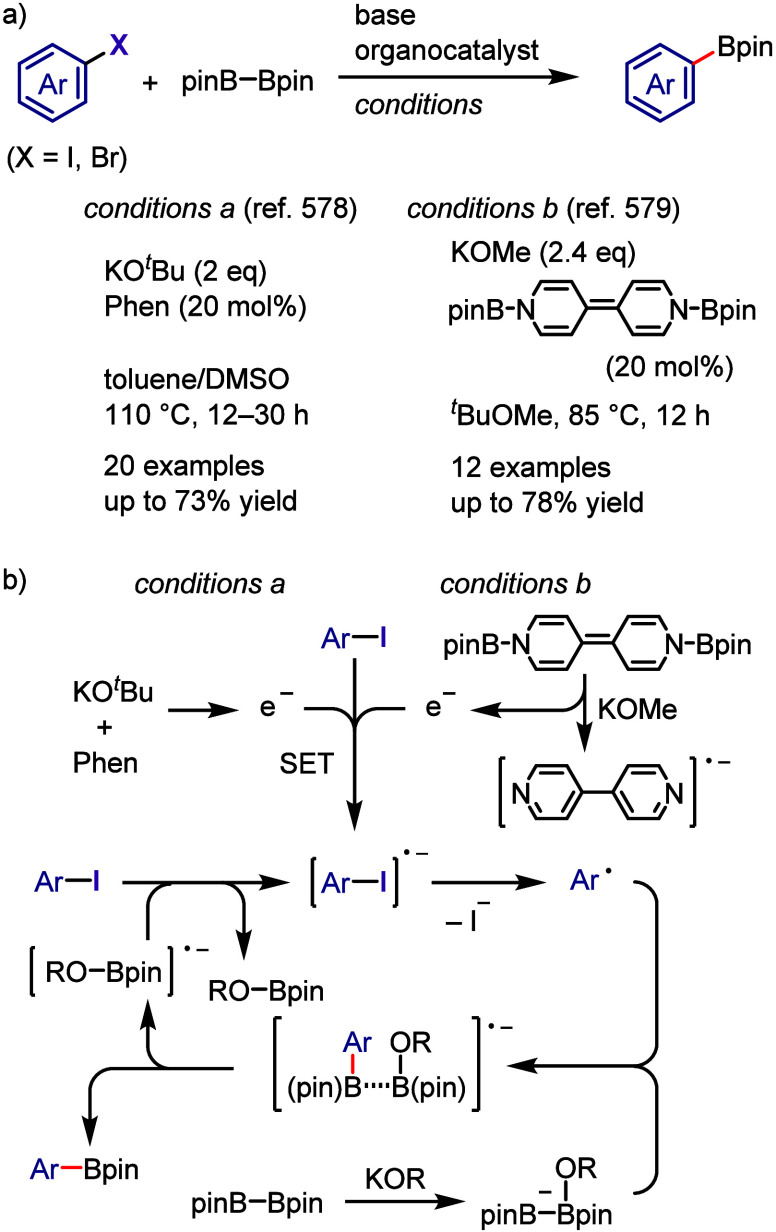
Dehalogenative Borylation Aryl Halides Using 1,10-Phenanthroline or Diboryl-bipyridinylidene Promoter

Borylation of aryl iodides by using B_2_(OR)_2_ has been achieved in the presence of Cs_2_CO_3_ under heating conditions.^577^ The combination of KO^*t*^Bu and 1,10-phenanthroline KO^*t*^Bu also enables this reaction (Scheme 91a, conditions a).^578^ The organic promoter *N*,*N*′-diboryl-4,4′-bipyridinylidene was also utilized with KOMe to activate the aryl halide (Scheme 91a, conditions b).^579^ The reaction mechanism is initiated by the SET process using 1,10-phenanthroline and KO^*t*^Bu or diboryl-bipyridinylidene to generate the aryl radical, which reacts with the in situ generated boronate anion to afford the aryl boronic acid pinacol ester (Scheme 91b).

**Scheme 92:**
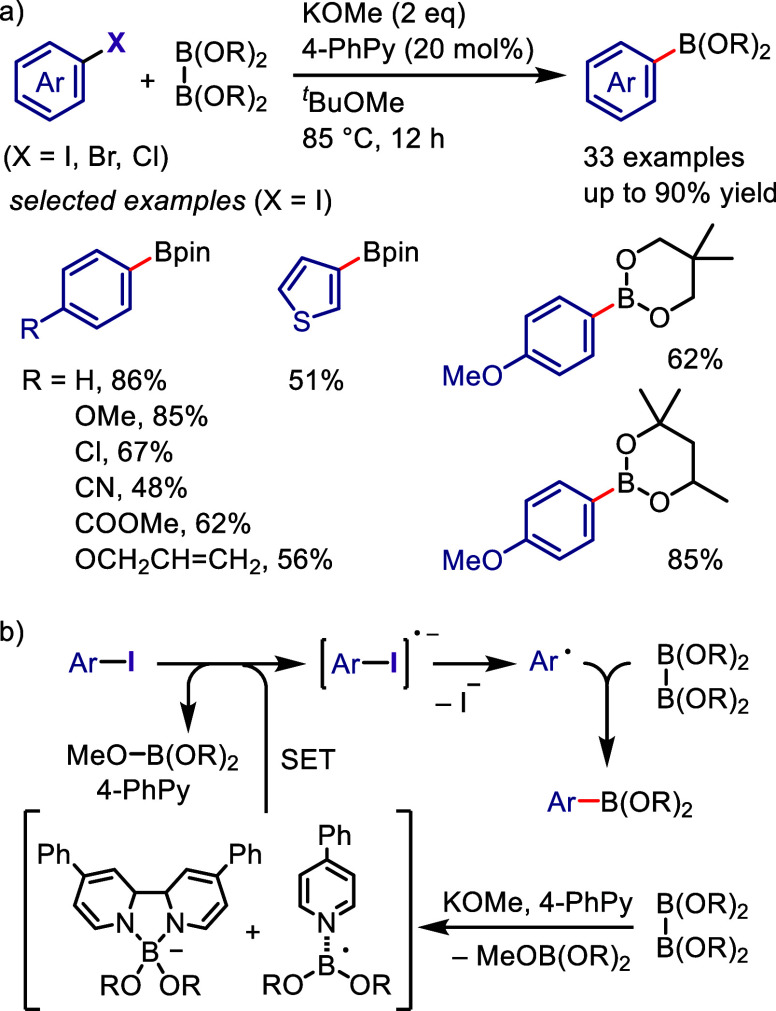
Dehalogenative Borylation Aryl Halides Utilizing Boryl-pyridine Complex

Jiao et al. developed efficient catalytic conditions using 4-phenylpyridine to activate the cross-coupling of haloarenes with diboron reagents and synthesis of arylboronates (Scheme 92a).^580−584^ The combination of diboron with the pyridine derivative in the presence of an appropriate base generates the boryl-pyridine complexes, which are well-known potent super electron donors (SEDs), that can be used effectively to reduce aryl halides under thermal and visible light conditions (Scheme 92b).

**Scheme 93:**
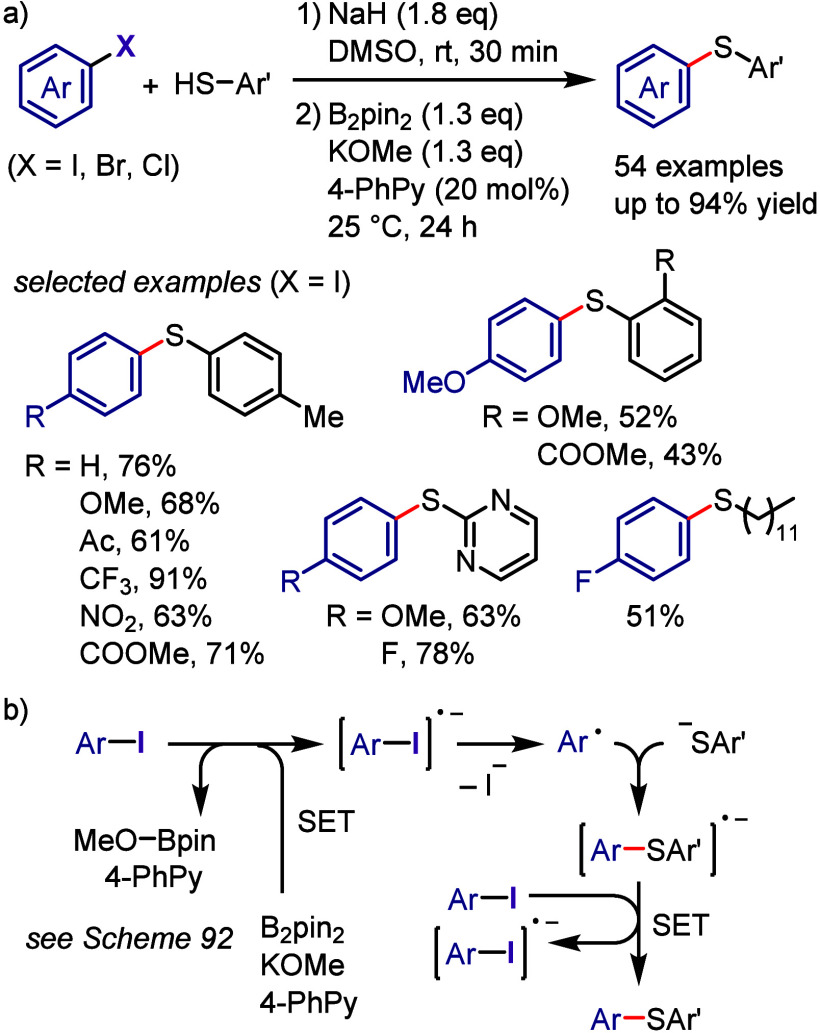
Pyridine-Boryl SEDs Promoting Cross-coupling of Aryl Halides with Thiols

In addition to its use in borylation, the versatility of boryl-pyridine complexes was expanded to induce cross-coupling of aryl halides with thiols (Scheme 93a).^585^ A broad array of (hetero)aryl iodides and aliphatic/aromatic thiols reacted well, and a wide range of functional groups was tolerated. The aryl radical generated by the boryl-pyridine complex reacts with the thio alkoxide to afford the thio ether radical anion, which undergoes the SET process to afford the corresponding thioether (Scheme 93b).

### 3.2 Organocatalyst-Free Dissociation of Ar–I Bond

**Scheme 94:**
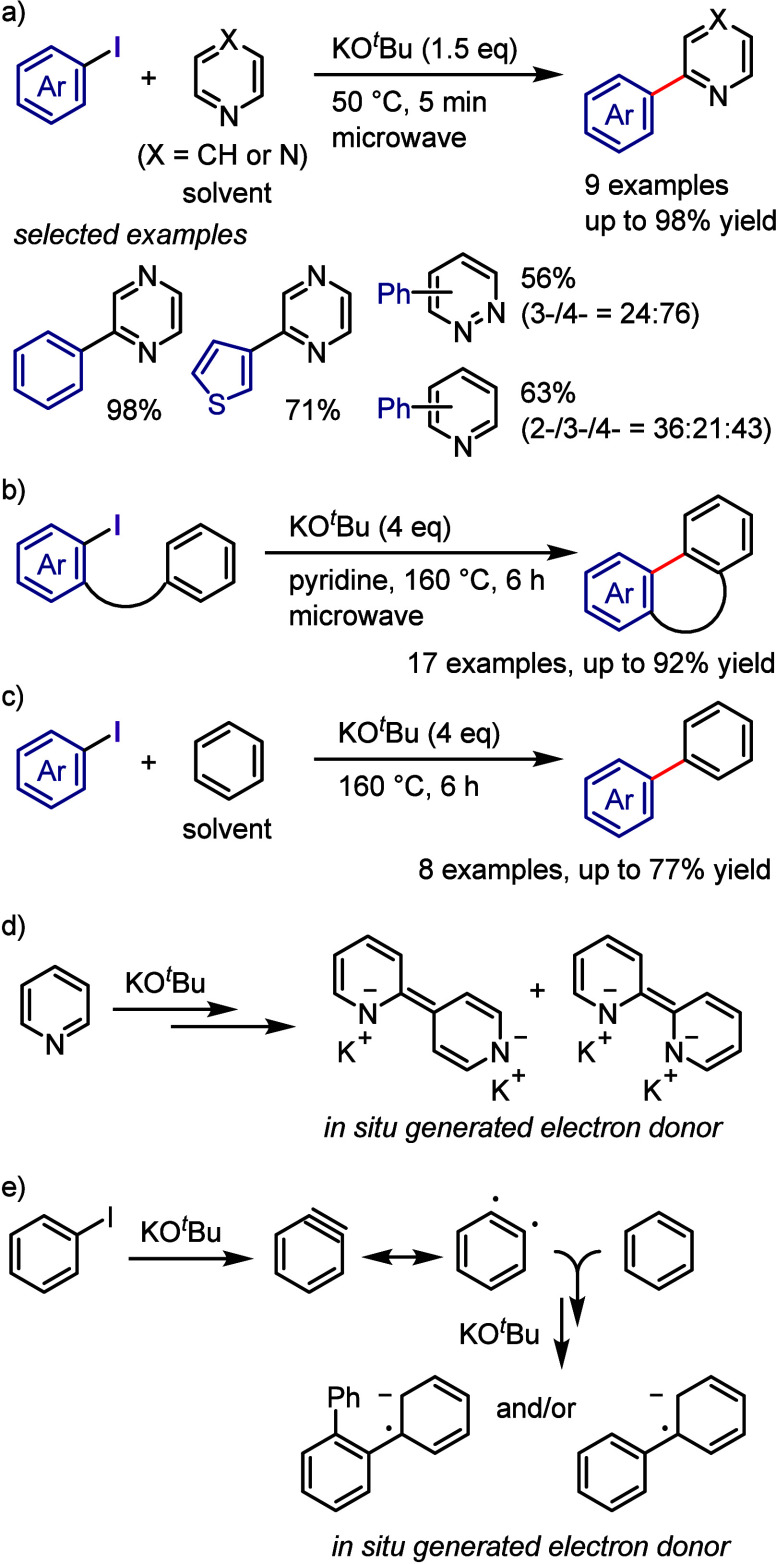
Organocatalyst-Free C–H Arylation of Arenes with Aryl Iodides

As mentioned earlier, Itami et al. reported the first transition metal-free arylation of electron-deficient *N*-heterocyclic compounds (used as solvent) with aryl halides and the synthesis of heterobiaryl products using KO^*t*^Bu as the sole promoter under microwave conditions (Scheme 94a).^494,495^ Charette et al. reported an efficient intramolecular arylation in the presence of KO^*t*^Bu in pyridine under microwave conditions that did not require additional organocatalysts (Scheme 94b).^586^ Furthermore, Wilden et al. observed that organic promoters are not essential for cross-coupling of unactivated benzene with aryl iodides as the reaction can be activated with KO^*t*^Bu alone (Scheme 94c).^587,588^ The reaction proceeded via the SET to initiate the generation of the aryl radical; however, the exact mechanism of this step was not clear.

Bisai et al. noted the possibility of performing the intramolecular BHAS reaction under organocatalyst and organocatalyst-free conditions in the presence of KO^*t*^Bu using mesitylene as a solvent.^589,590^ Murphy et al. studied in-depth the above-mentioned reactions, in which pyridine was used as a substrate and/or solvent with KO^*t*^Bu as the only promoter.^497,591,592^ As butoxide is not capable of directly initiating the radical reaction, the formed radicals must be generated in another way. Thus, after extensive computational and experimental studies, Murphy et al. observed the generation of super electron donor species (Scheme 94d) which are assumed to be responsible for the reduction of the aryl halides in Schemes 94a and 94b.

Additionally, for the coupling reaction between iodobenzenes and benzene using KO^*t*^Bu as the sole additive, Murphy et al. proposed a different mechanism from that suggested by Wilden. The researchers assumed a more sluggish activation mode can exist in the absence of the organic additive (Scheme 94e).^497,591,592^ Intensive experimental and theoretical studies unambiguously indicated the formation of benzyne which can be used for activating the process. In the presence of KO^*t*^Bu, aryl iodide can *in situ* generate benzyne in very low concentrations. Benzyne can work as a diradical, reacting with benzene to form distal diradicals, which serve as super electron donor species.

**Scheme 95:**
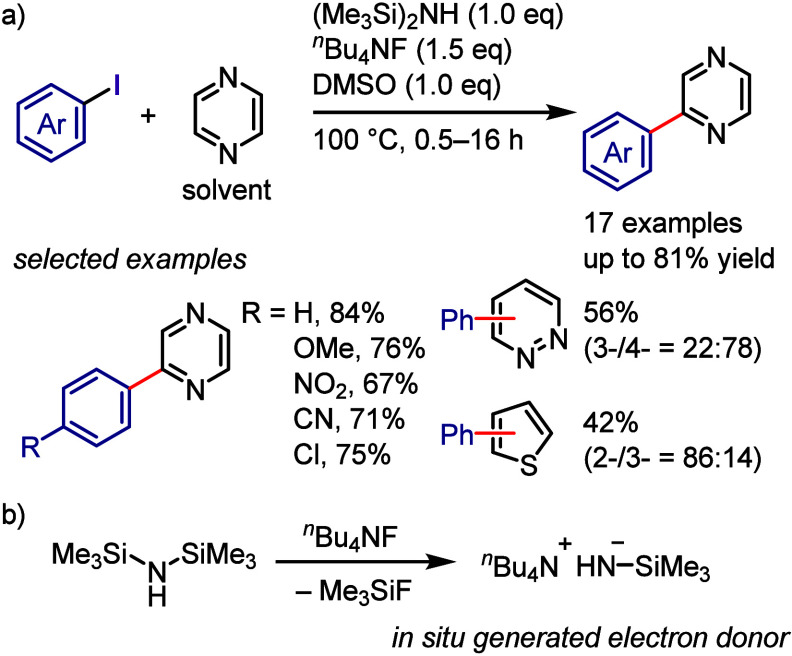
Amide Base Generated In Situ to Promote Csp^2^-H Arylation with Aryl Iodide

A combination of hexamethyldisilazane (HMDS) and tetramethylammonium fluoride (TMAF) was used by Kondo et al. to in situ generate an amide base to promote the cross-coupling of aryl iodide with C–H heteroarenes (Scheme 95a).^593^ Electronically and sterically diverse (hetero)aryl iodides reacted effectively with pyrazine to afford the corresponding heterobiaryls. Other C–H heteroarenes such as quinoxaline, pyridazine and thiophene afforded mixtures of regioisomers in moderate yields. The reaction mechanism involves the HMDS reacting with TMAF to generate the [(TMS)HN^–^ Me_4_N^+^] base, which transfers a single electron to aryl iodide, thereby promoting the following reaction (Scheme 95b).

**Scheme 96:**
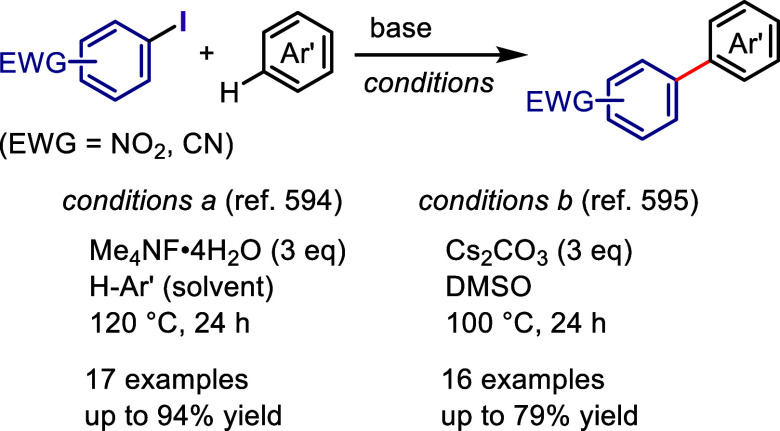
Base-Promoted Synthesis of Biaryls from Aryl Iodides

Electron-deficient aryl iodides were reacted with arenes in DMSO in the presence of tetramethylammonium fluoride or Cs_2_CO_3_ to afford the corresponding cross-coupling products (Scheme 96).^594,595^ Under these conditions, these additives participated as promoters to give a single electron to the deactivated aryl iodides and generate aryl radicals for cross-coupling with arenes.

**Scheme 97:**
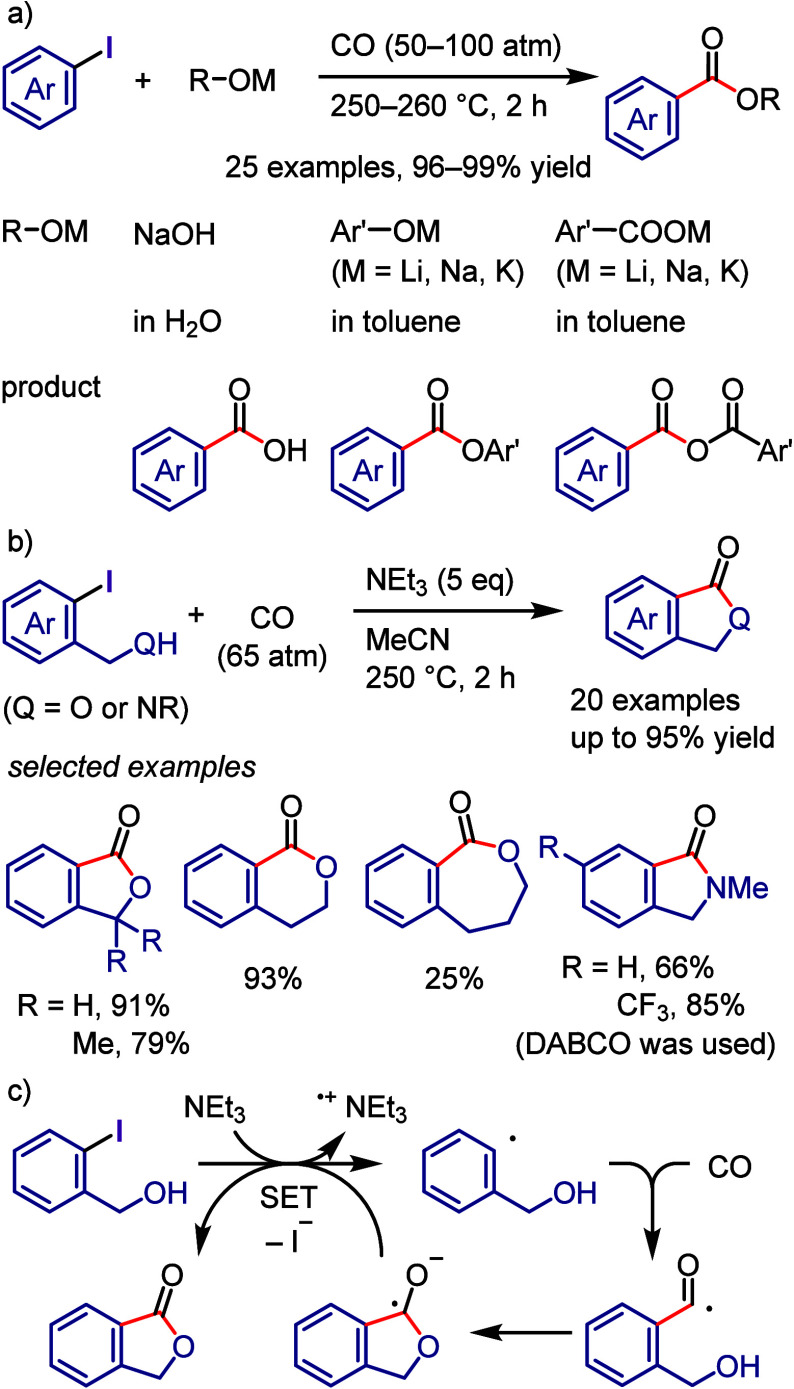
Organocatalyst-Free Carbonylation of Aryl Iodides

Fukuoka et al. developed a radical initiator-free protocol for the carbonylation of aryl iodides with CO in the presence of aryloxide, base/H_2_O or aryl carboxylic acid salt for the synthesis of aryl acid derivatives via a probable SET initiated radical mechanism (Scheme 97a).^596^ Similarly, Ryu et al. performed the intermolecular Heck aminocarbonylation of aryl iodides^597^ and intramolecular Heck carbonylation of 2-iodobenzyl alcohols and 2-iodobenzyl amines with CO for the synthesis of benzolactone and benzolactam derivatives, respectively (Scheme 97b).^598,599^ The scope of the alcohol substrate encompasses primary, secondary, and tertiary 2-iodobenzylalcohols in addition to 2-(2-iodophenyl)ethanol and 3-(2-iodophenyl)-1-propanol for the synthesis of 5–7 membered benzolactones. In reactions with 2-iodobenzylamine, DABCO was replaced Et_3_N to avoid the formation of *N*-ethyl side products. The postulated reaction mechanism starts with the SET from Et_3_N to aryl iodide to generate the aryl radical, which reacts with CO to afford the acyl radical. The following intramolecular nucleophilic addition by the OH group generates the cyclized intermediate and the SET process affords the final product.

**Scheme 98:**
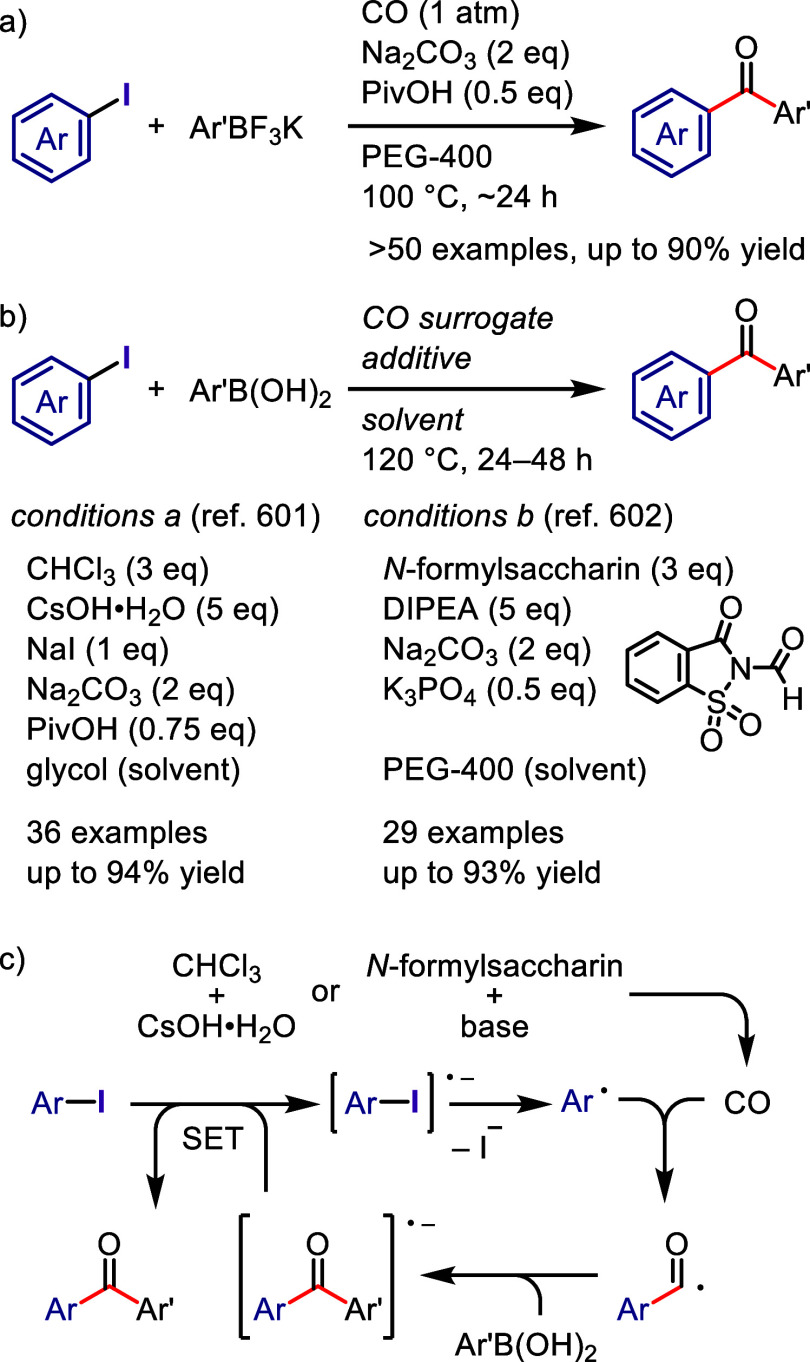
Carbonylative Coupling of Aryl Iodide and Aryl Boronic Acid with CO

Han et al. demonstrated a transition metal-free carbonylative coupling of aryl iodide with aryl trifluoroborate salt under CO using simple basic conditions (Scheme 98a).^600^ In addition, the same group used CHCl_3_ or *N*-formylsaccharin as the CO surrogates (Scheme 98b).^601,602^ The high reaction temperature and base are responsible for the dissociation of the aryl-to-iodide bond and generation of the aryl radical which is trapped by the generated CO from the reaction of CHCl_3_ or *N*-formylsaccharin with base (Scheme 98c). The obtained acyl radical reacts with aryl boronic acid in the presence of base to generate the biaryl ketone radical anion, which undergoes the SET process to aryl iodide to afford the biaryl ketone.

**Scheme 99:**
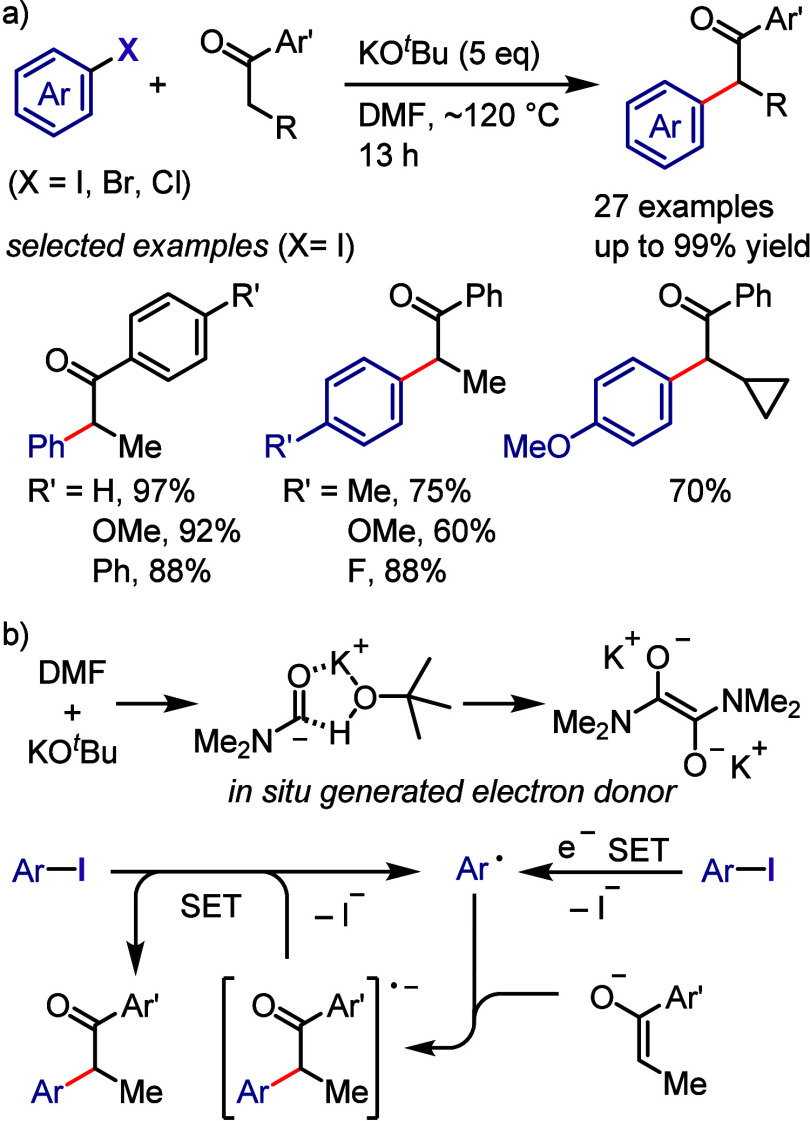
KO^*t*^Bu/DMF Initiated α-Arylation of the Enolizable Aryl Ketone by Aryl Iodide

Taillefer et al. discovered the capability of additive-free KO^*t*^Bu/DMF to initiate the S_RN_1 coupling reaction between aryl halides and enolizable aryl ketones and the synthesis of C(sp^3^)-arylation products (Scheme 99a).^603^ The combination of KO^*t*^Bu/DMF appears to be crucial as no reaction occur if one of these reagents is replaced. A radical chain mechanism was postulated based on the performed theoretical and experimental studies (Scheme 99b). Deprotonation of DMF by KO^*t*^Bu generates the corresponding carbamoyl anion, which reacts with DMF to afford the dianion. These anion species can serve as single electron donors to aryl iodide.^591^ The thus-generated aryl radical reacts with enolate to afford the aryl ketone radical anion, which exchanges an electron with aryl iodides to afford the final product.

**Scheme 100:**
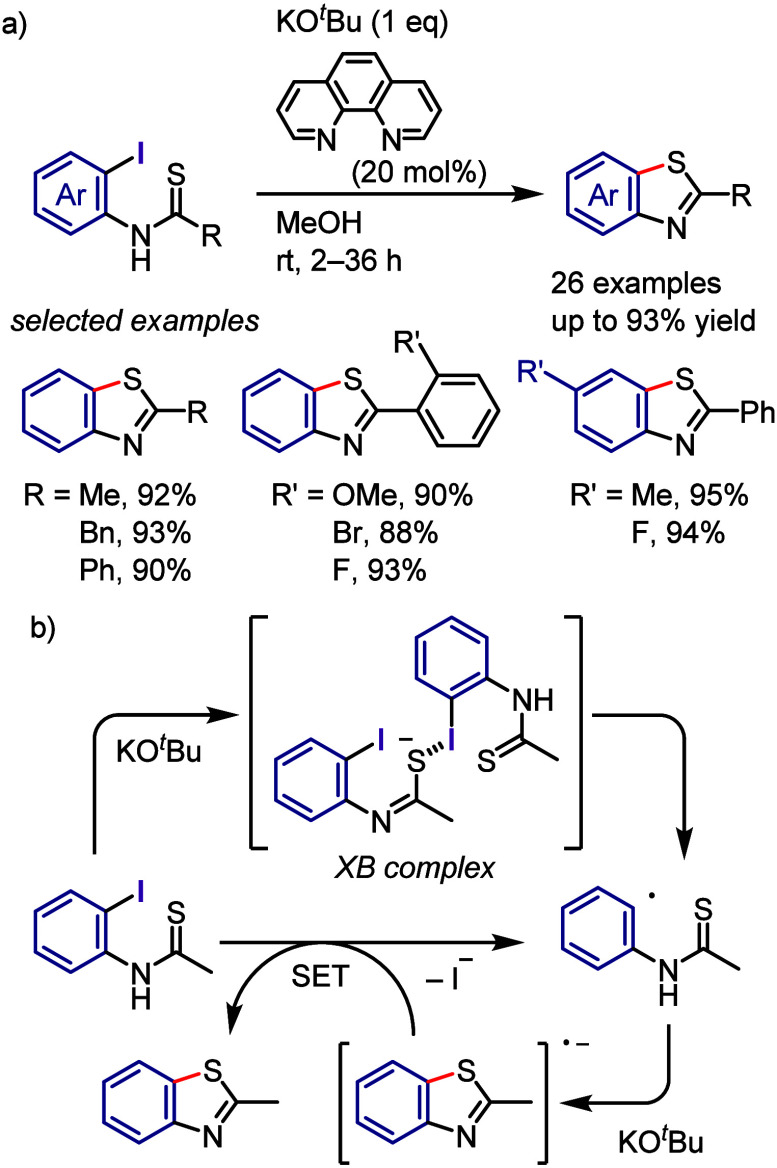
Halogen-Bond Assisted Ar–I Bond Dissociation and Intramolecular Cyclization

The halogen bond assisted dissociation of the aryl-iodide bond was found to be an efficient strategy to construct heterocycles under mild conditions. Thus, *ortho*-iodothioanilide in the presence of KO^*t*^Bu and 1,10-phenanthroline (additive) at room temperature underwent intramolecular C–S cross-coupling to afford a broad variety of 2-substituted benzothiazoles (Scheme 100a).^604^ The control experiment revealed that the free-NH of the thioanilide is key to this type of cyclization. XRD, DFT, NMR and UV studies indicated the presence of intermolecular (I···S) halogen bonding, that was responsible for activating the aryl-iodide bond via the SET process (Scheme 100b). A rational mechanism was initiated by deprotonating the NH-group to afford the corresponding anion, which interacted with the iodine atom of another molecule via halogen bonding to generate the XB complex. Aryl-iodide bond activation through the SET from the thiolate anion generated the aryl radical. Deprotonation by added base followed by intramolecular cyclization produced the benzothiazole radical anion, which underwent the SET process with another substrate to afford the final product.

**Scheme 101:**
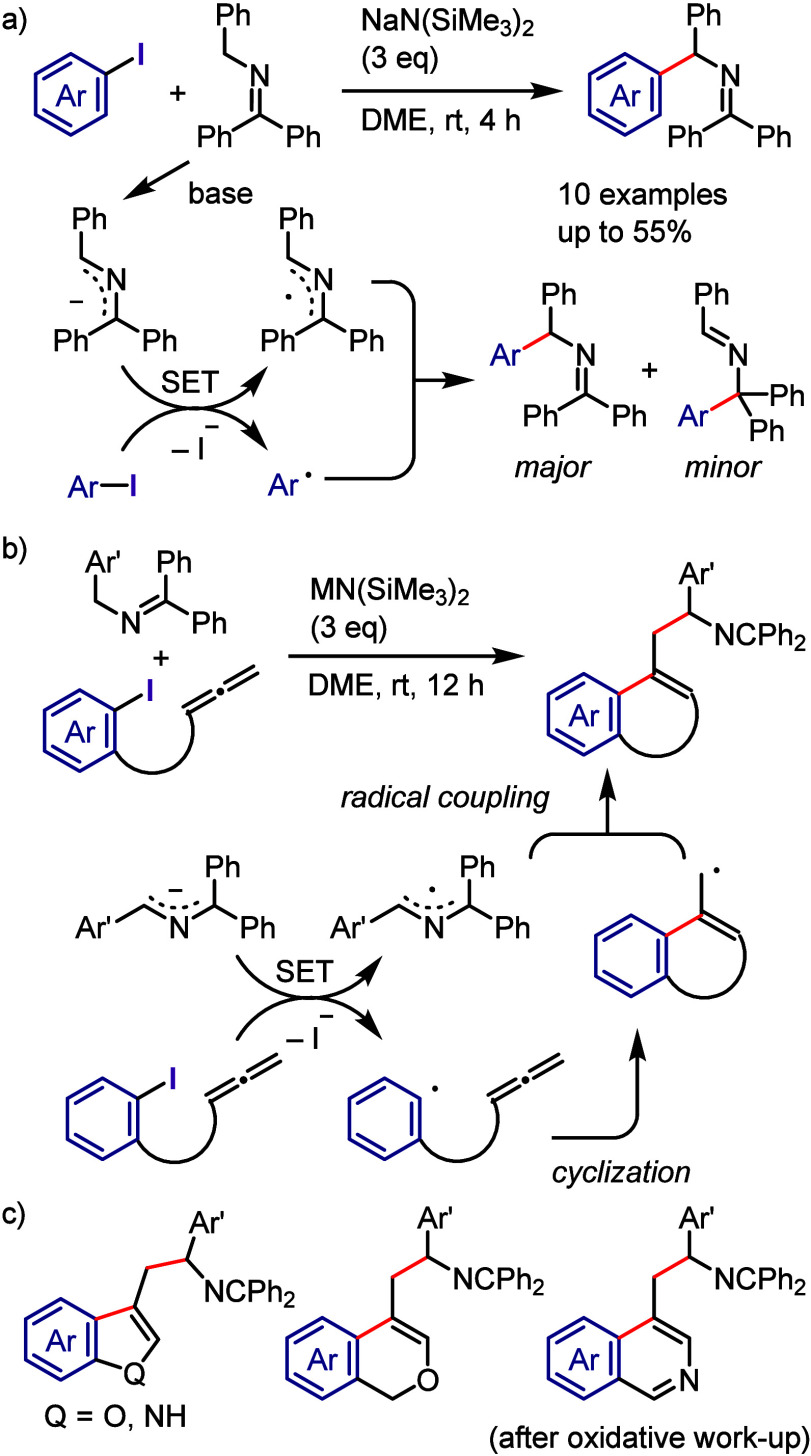
2-Azallyl Anion Initiated Radical Cyclization and Tandem Formation of Heterocyclic Skeletons

Walsh et al. reported that the 2-azaallyl anion generated from ketimine and strong base serves as a super electron donor for aryl iodides. The SET process proceeds to generate aryl and 2-azaallyl radicals, which undergo radical coupling to form C–C bonds (Scheme 101a).^605−607^ The same group combined this reaction with an intramolecular cyclization process to develop the constructions of various heteroaromatics bearing ethylamine moieties (Scheme 101b).^608−611^ Iodoarenes bearing an allenyl moiety undergo the SET process induced by the 2-azaallyl anion to generate the corresponding aryl radical. Intramolecular cyclization followed by radical coupling with the 2-azaallyl radical affords the final products. This strategy was applied to the construction of benzofuran, isochromen, isoquinoline, and indole derivatives bearing an ethyl amine moiety (Scheme 101c).

**Scheme 102:**
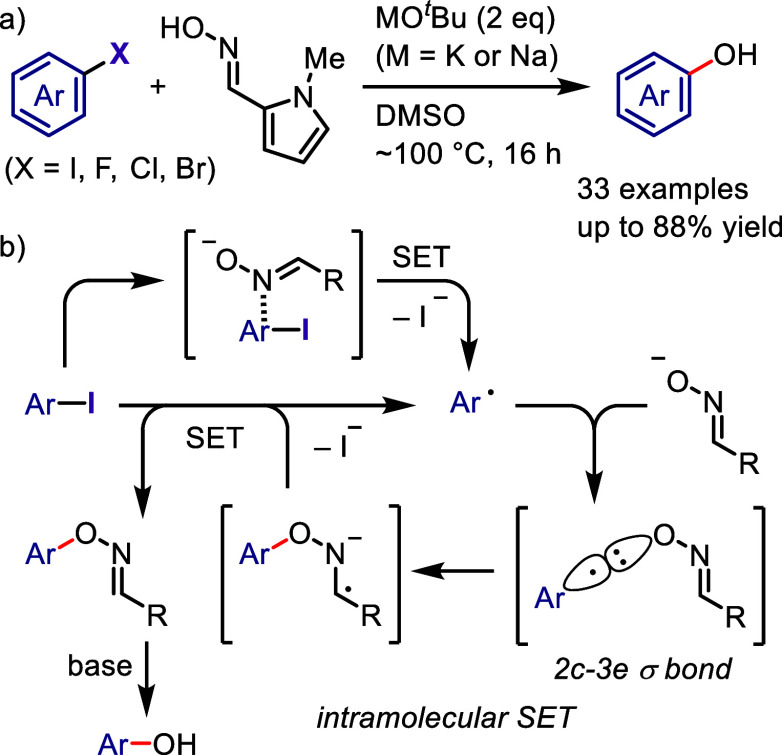
Oxime as Super Electron Donor and Hydroxide Equivalent in Dehalogenative Hydroxylation of Aryl Halides

James et al. discovered that oxime anions serve as dual S_RN_1 initiators and hydroxide surrogates in the dehalogenative hydroxylation of aryl halides to afford phenols under mild conditions (Scheme 102a).^612^ The S_RN_1 chain mechanism was supported by further experimental and computational studies. Either the oxime anion or charge-transfer (CT) complex could transfer a single electron to aryl halide under heat or light stimulation (Scheme 102b). The generated aryl radical interacts with the oxime anion via the formation of a weak 2-center-3-electron σ-bonded intermediate, which undergoes intramolecular SET into the adjacent π* orbitals to afford the delocalized radical anion (*E*_1/2_ = −2.14 vs SCE). Electron exchange with another aryl halide generates the arylate oxime, which transforms to the corresponding phenol under basic conditions.

**Scheme 103:**
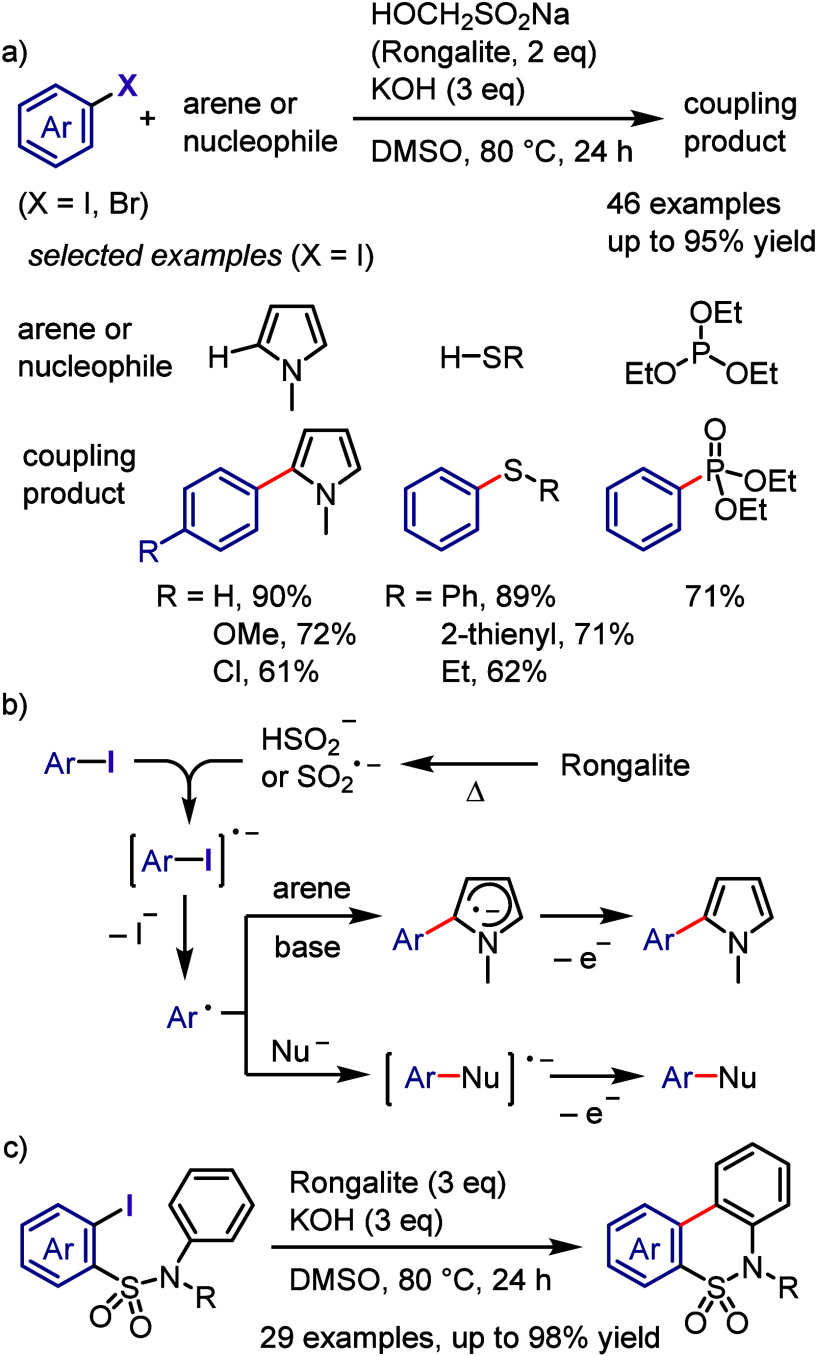
Sulfoxylate Anion Radical Effectively Induces the Generation of the Aryl Radical from Aryl Halides

Wang et al. discovered rongalite (sodium hydroxymethylsulfinate) as a novel precursor of the super electron donor sulfoxylate anion radical (SO_2_^•–^) species which initiates homolysis of aryl-halide bonds and generates the aryl radical for the arylation of (hetero)arenes, thiolates, sulfides, sulfinates, phosphines, phosphites, etc., through the BHAS or S_RN_1 mechanism (Scheme 103a).^613^ The radical chain cycle begins with the generation of SO_2_^•–^ species from rongalite or Na_2_S_2_O_4_ under thermal conditions (Scheme 103b). SO_2_^•–^ transfers a single electron to the aryl halide to generate the aryl radical, which reacts with other arenes or nucleophiles to afford the corresponding radical anion intermediates. The SET process proceeds to afford the coupling products. Laha et al. demonstrated the intramolecular arylation of 2-halobenzenesulfonamide and generated biarylsultams induced by the sulfoxylate anion radical (Scheme 103c).^614^

### 3.3 Dissociation of Ar–I to Aryl Anion

**Scheme 104:**
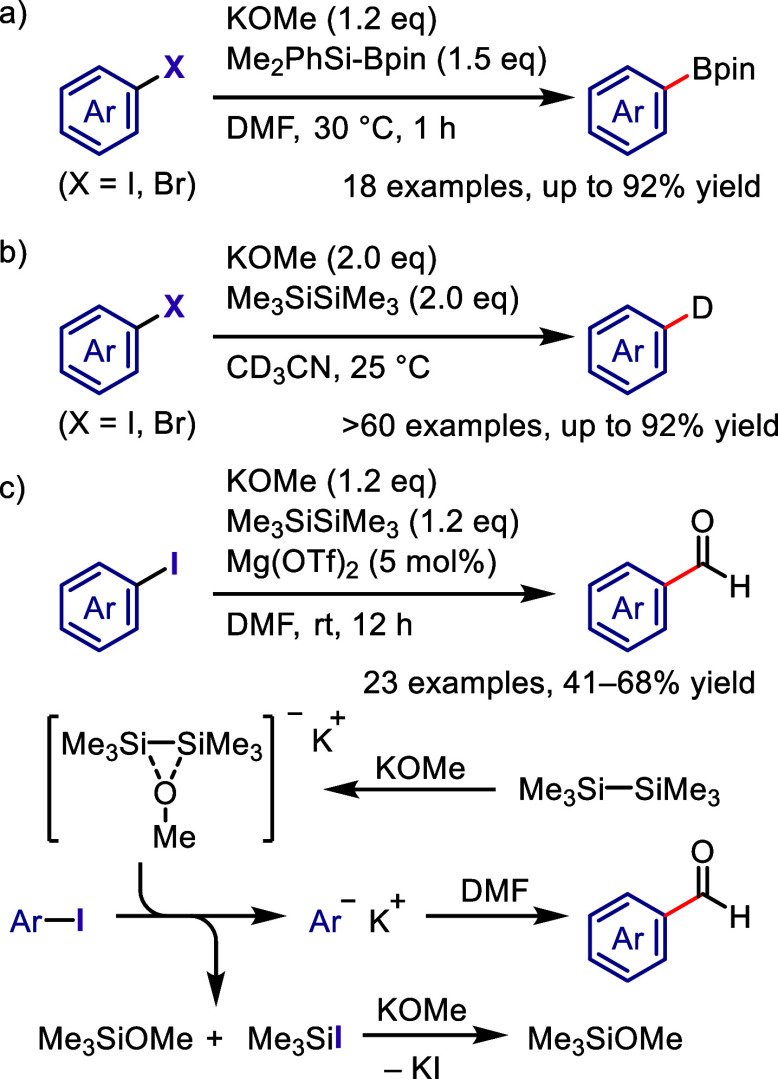
KOMe-Induced Dehalogenative Functionalization of Aryl Iodides

In addition to the dissociation of the Ar–I bond to generate the aryl radical; the bond can be cleaved to generate the aryl carbanion. KOMe was applied to the transition metal-free borylation of aryl halides in the presence of silylborane via the in situ generation of aryl carbanions, under ambient reaction conditions (Scheme 104a).^615^ Combining with hexamethyldisilane (TMS-TMS) enables dehalogenative deuteration in CDCN solvent (Scheme 104b).^616^ Additionally, KOMe/TMS-TMS reagents were involved in the dehalogenative formylation of aryl iodides with DMF to afford the corresponding aryl aldehydes (Scheme 104c).^617^ A plausible mechanism was suggested based on control experiments and extensive NMR studies. The methoxy anion coordinates the two silyl groups of TMS-TMS to generate hypercoordinate silane species as a strong base, which reacts with Ar–I to generate the aryl carbanion. The addition of Mg(OTf)_2_ improved the yield slightly via coordination/activation of the carbonyl group of DMF for the reaction with the aryl carbanion nucleophile.

**Scheme 105:**
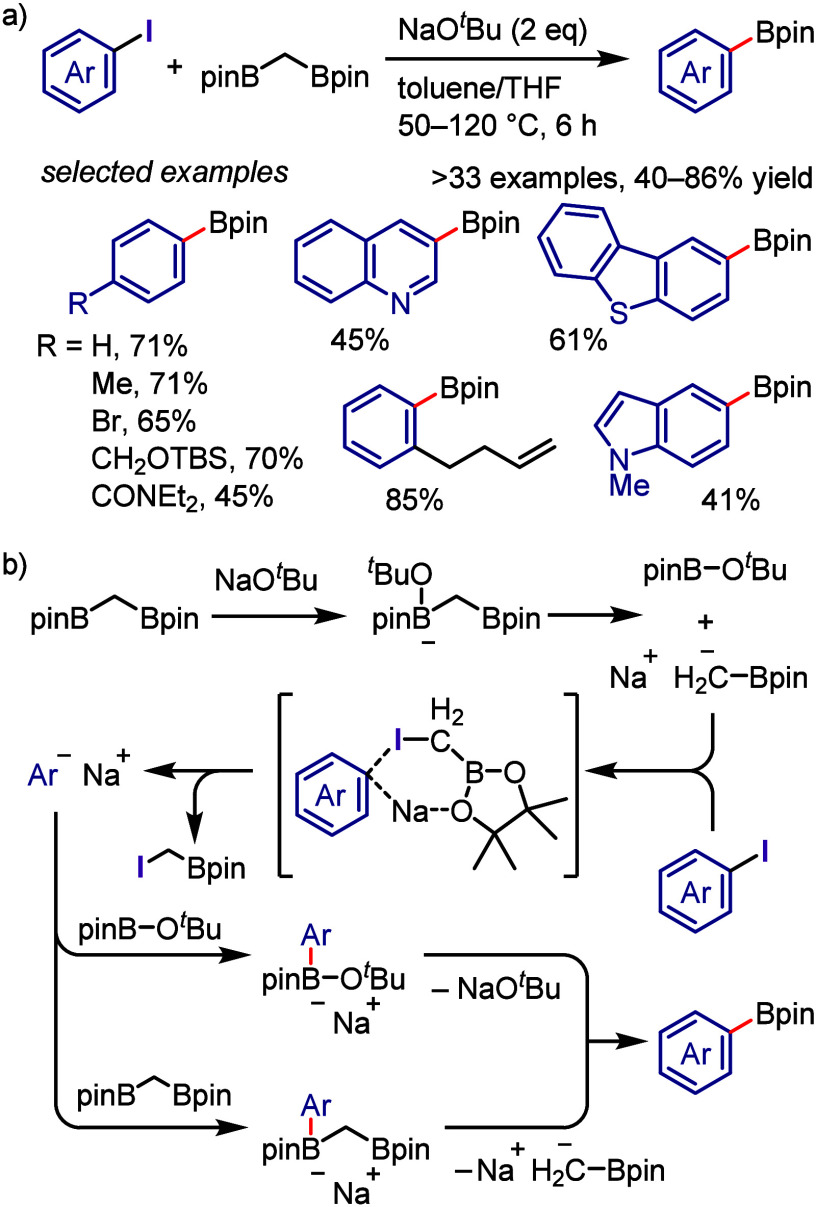
Borylation of Aryl Iodide with 1,1-Bis[(pinacolato)boryl]methane

Baik and Cho et al. reported the borylation of aryl iodides using commercially available 1,1-*bis*[(pinacolato)boryl]methane as a potent precursor to activate the Ar–I bond and as a boron source in the presence of NaO^*t*^Bu (Scheme 105a).^618^ Extensive theoretical and experimental studies supported the postulated mechanism in Scheme 105b. The reaction of pinB-CH_2_–Bpin with *tert*-butoxide anion forms the *tert*-butoxyboronate ester and borylmethyl anion. The latter and aryl iodide form a Lewis acid/base complex, that produces the sodium aryl anion. The generated aryl anion attacks the Lewis acidic boron center to form the borate species, which collapsed to afford the final product.

**Scheme 106:**
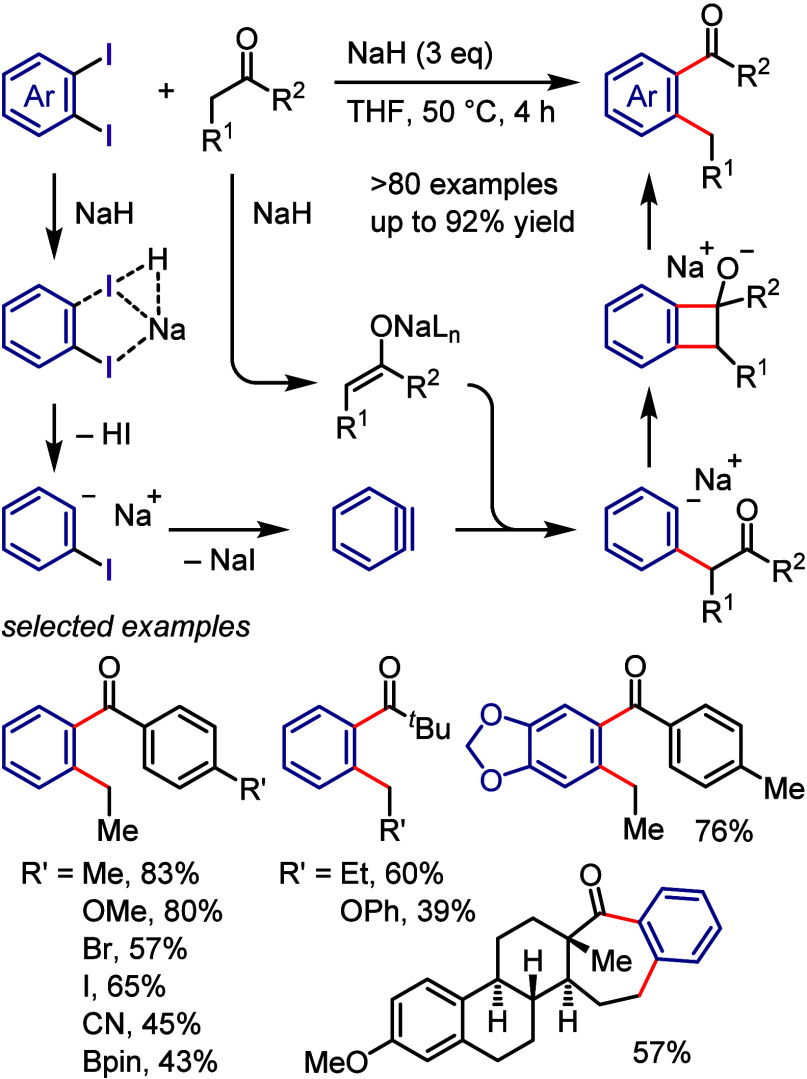
NaH-Activated Generation of Aryne for Double C–C Bond Formation

Sodium hydride exhibited potency alone or as an iodide composite for the hydrodehalogenation of aryl halides.^619,620^ Recently, Zhang et al. established the NaH-mediated activation of *ortho*-diiodoarenes and generated arynes in a controlled manner, that enabled their insertion into the C–C σ-bonds of unactivated ketones and the synthesis of vicinal difunctionalized arenes (Scheme 106).^621^ DFT calculations revealed the crucial role of the two adjacent iodines in *ortho*-diiodoarene for the interaction with NaH, which facilitates the generation of the aryl anion followed by that of the aryne. The enolate generated from the ketone reacts with the aryne with high regioselectivity for substrates comprising bulk substituents. The formed anion intermediate undergoes intramolecular cyclization and a Fries-type rearrangement to afford the final product. This strategy is compatible with diverse aryl alkyl and alkyl–alkyl ketones and unsymmetrical *ortho*-diiodoarenes to afford regioisomeric products with selectivity depending on the type and position of the substituents.

**Scheme 107:**
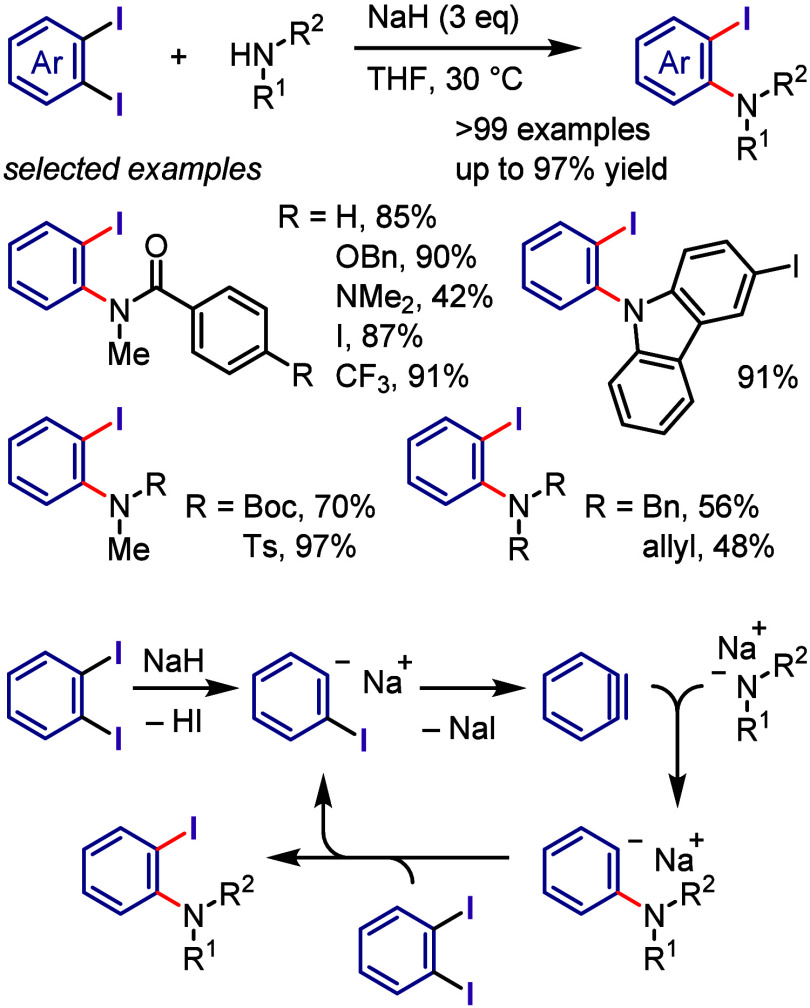
NaH-Activated Generation of Aryne for C–N and C–I Bond Formations

Analogously, a NaH/*o*-diiodoarene combination was used for the *N*-arylation of a broad array of secondary amides and amines in addition to *N*-heterocycles, hydrazine, urea, lactam, and sulfonamide derivatives (Scheme 107).^622^ In this strategy, *o*-diiodoarene acted as an aryne precursor and electrophilic iodine donor.

## 4 Photoinduced Ar–I Bond Dissociation

**Scheme 108:**
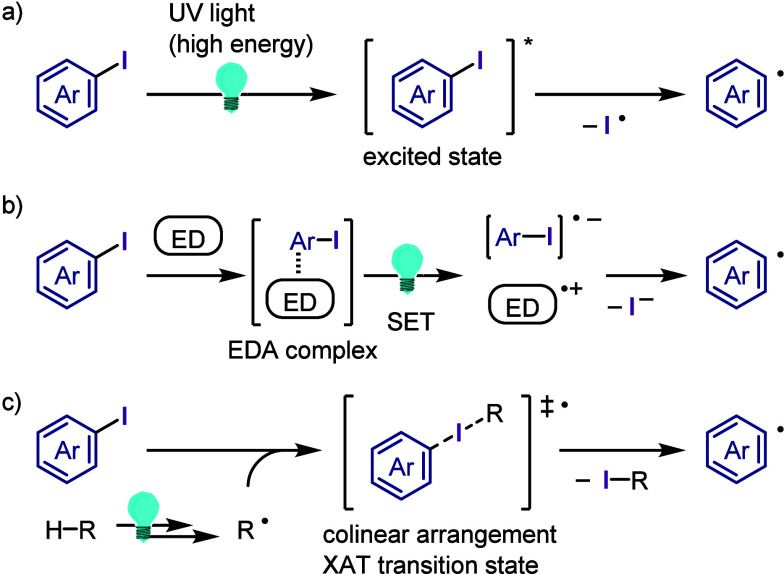
General Approaches for the Generation of Aryl Radical from Aryl Iodide

Photochemistry affords powerful opportunities to expand the potential of organic chemistry as harnessing the readily accessible light energy instead of the traditional thermal force is an abundant, renewable, and clean energy approach for performing novel molecular transformations and producing unique functional molecules. Therefore, significant progress of light-induced Ar–I bond dissociation has occurred and various transformations can be performed under benign conditions.^623^ Light-induced aryl-halogen bond dissociation, in the presence/absence of photocatalyst, allows for new green and sustainable avenues in aromatic substitution reactions.^375,624−643^ Upon absorbing light, the exciting components provide intriguing transformations that are relatively complicated or even not possible using other ground state approaches. The direct transformation of aryl halide into the related aryl radical represents a crucial approach to access a reactive synthetic intermediate to use in subsequent chemical transformations. The development of aryl radical-mediated transformations has played an essential role in shaping the development of synthetic radical chemistry.^623^ Generating aryl radicals from the related aryl halides under photoirradiation conditions can be accomplished via Ar–X bond homolysis, SET, and halogen-atom transfer (XAT) pathways (Scheme 108). The classic photoactivation via homolytic cleavage of the Ar–X bond usually requires irradiation with high-energy UV-light (Scheme 108a). This direct activation approach requires specialized setups and displays low functional group compatibility, which consequently limit its application in organic synthesis. The addition of electron donor species (ED) assists the activation of aryl halides via the formation of an EDA complex (Scheme 108b). Photoirradiation leads to the injection of an electron to the unoccupied orbital of aryl halide, followed by an intramolecular electron transfer from the π-orbital to the antibonding σ* orbital and cleavage of Ar–X bond. The SET to aryl iodide and Ar–I bond cleavage is much more thermodynamically and kinetically, respectively, favorable than that to other aryl halides.^644^ The XAT-based activation method involves the direct homolysis of an Ar–X bond by abstraction of the halogen atom with a suitable radical species (R^•^), which is generated from alkylamine under photoirradiation conditions. The abstraction step proceeds via a colinear arrangement controlled by several factors (Scheme 108c).^645^ In this section, we highlight the recent progress in this flourishing area by discussing the recent light-induced arylation with aryl iodide with or without organophotocatalysts, and their mechanisms.

### 4.1 Photocatalyst-Free Dissociation of Ar–I Bond

**Scheme 109:**
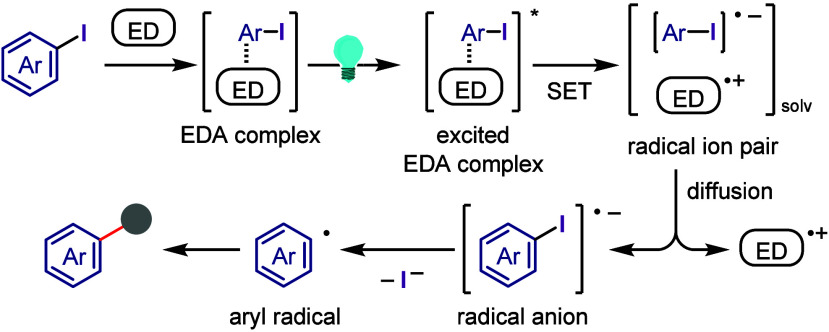
EDA Complexation and SET for the Generation of Aryl Radical from Aryl Iodide

The photocatalyst-free light-induced Ar–I bond cleavage and construction of the C–C and C–heteroatom bonds via an initiation step of either direct excitation of an aryl halide or the SET process, is a highly desired transformation as it provides an operationally simple, inexpensive, and environmentally benign transformation under mild conditions with high functional group tolerance. The SET process could be possible if one of the reaction components can absorb light or when the reactants form photoactive EDA complexes, comprising an electron acceptor aryl iodide and electron donor compound (Scheme 109).^646−657^ The UV–vis absorption spectra of the EDA complex have a new broad absorption peak called the CT band, that has shifted to longer wavelengths. Photoirradiation at the wavelength of the CT band generates the excited EDA complex, which undergoes intramolecular SET from the donor to the Ar–I to generate a pair of radical ions trapped in the solvent cage. Diffusion radical ions from the solvent cage affords the aryl iodide radical anion which collapses to generate the reactive aryl radical. The presence of the iodide leaving group causes the irreversible C–I bond cleavage to occur faster than the competing back electron transfer reverse process within the radical ion pair. Iodoarene-based EDA complex photochemistry has been noted as a robust tactic for broadening the potential of light-induced aryl radical synthetic chemistry. Thus, light could serve as an essential and unique energy source to cleave the Ar–I bonds, and chemical transformations that are impossible or difficult to conduct under alternative conditions become feasible.

#### 4.1.1 C–Chalcogen Bond Formation

**Scheme 110:**
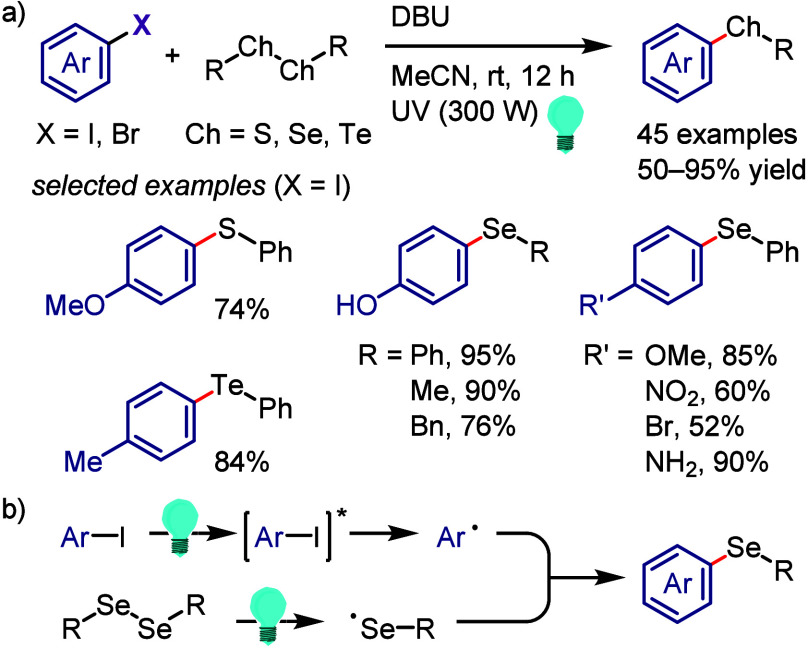
Direct Homolysis of Aryl Halide Bond under UV-Light and the Construction of C–Se, C–S, and C–Te Bonds

The feasibility of Ar–halide bond homolysis under UV irradiation persuaded many research groups to hypothesize photoinduced cross-coupling processes for the construction of C–chalcogen bonds.^658−660^ The reaction of an aryl halide with diselenide, disulfide, and ditelluride under the optimized UV-light conditions formed the corresponding selenoether, thioether, and diaryltelluride, respectively (Scheme 110a).^658^ Aliphatic and aromatic dichalcogenide substrates and various aryl halides bearing OH, NH_2_, and allyl groups were well-tolerated. Radical trapping experiments and electron spin resonance spectroscopy revealed the generation of a carbon centered free radical during the reaction, thus supporting the reaction mechanism in Scheme 110b. UV irradiation of the reaction mixture led to homolysis of the diselenide to the selenide radical and excitation of the aryl halide to generate an aryl radical, which reacted via radical–radical coupling to afford the aryl selenoether.

**Scheme 111:**
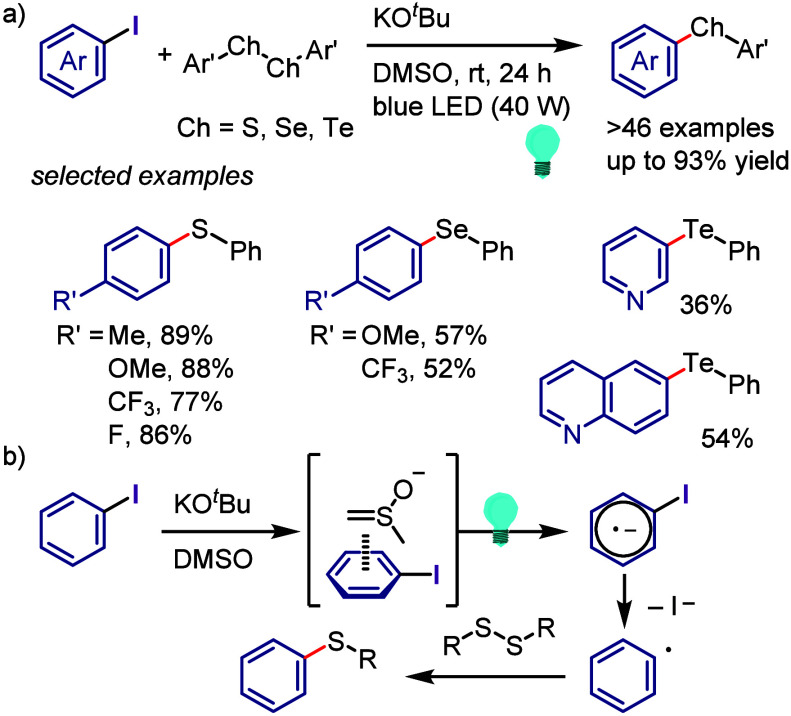
Photoinduced Charge Transfer from Dimsyl Anion for Ar–I Bond Dissociation

Laulhé et al. recognized the importance of electron-donor–acceptor (EDA) complex generation during visible-light-induced cross-coupling of an aryl iodide with diaryl dichalcogenides in the presence of KO^*t*^Bu/DMSO (Scheme 111a).^661^ The experimental and theoretical studies suggested the responsibility of an EDA complex between the in situ generated dimsyl anion and aryl iodide for the observed chemical reactivity. The EDA complex leads to the formation of the aryl radical via charge transfer; the aryl radical was trapped by the disulfide to afford the final product.

**Scheme 112:**
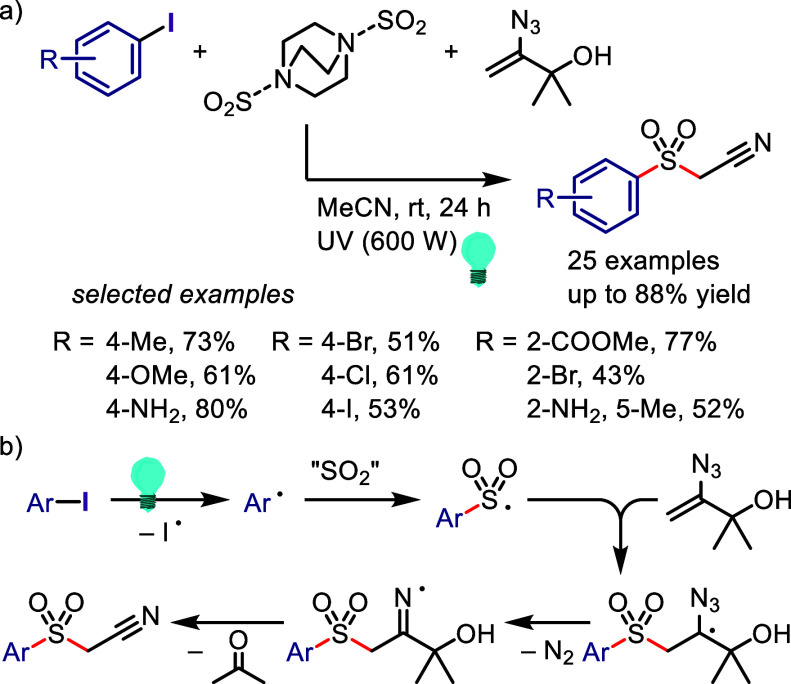
Photoinduced Synthesis of 2-Sulfonylacetonitriles via Insertion of SO_2_

Photoinduced aryl-halide bond dissociation along with the in situ liberation of SO_2_ using commercially available SO_2_-equivalents provide an attractive combination for the construction of diverse aryl sulfonyl-containing skeletons.^662−664^ Accordingly, Wu et al. envisioned a protocol for the synthesis of 2-(arylsulfonyl)acetonitrile via a one-pot cascade reaction of aryl iodide, SO_2_-equivalent (DABCO.(SO_2_)_2_), and 3-azido-2-methylbut-3-en-2-ol under UV-irradiation conditions (Scheme 112a).^664^ Mechanistic investigations indicated that UV irradiation of the aryl iodide provides an aryl radical, which undergoes SO_2_-insertion to afford an arylsulfonyl radical. Reaction of the generated radical with the alkene bearing azide group followed by release of N_2_ produces a nitrogen radical. Further C–C bond cleavage in the presence of an iodo-radical and DABCO delivers the final product and acetone.

#### 4.1.2 C–Chalcogen Bond Formation via EDA Complex with Chalcogen Anions

Phenolate and thiolate anions are among various organic anion species whose physicochemical properties can be markedly altered upon photoexcitation.^626,665,666^ These organic anions (which absorb light in the ground state) behave as strong reducing agents in the excited states and can generate reactive radicals from their stable precursors via the direct photoexcitation and SET processes. An alternative mechanism involved the affinity of electron-rich organic anions (which cannot absorb light) to associate with electron-deficient radical precursor substrates and form ground state EDA complexes (active toward light) which have the tendency to excite with light, undergo SET, and finally generate the reactive radical species.

The photochemistry of phenolate and thiolate anions as dual photocatalysts and reagents or only as photocatalysts is discussed in the section headed “Photoinduced Ar–I bond dissociation”.^667,668^ We discuss herein the versatility of thiolate anions as photocatalysts and reagents for cross-couplings and the construction of C–S bonds.

**Scheme 113:**
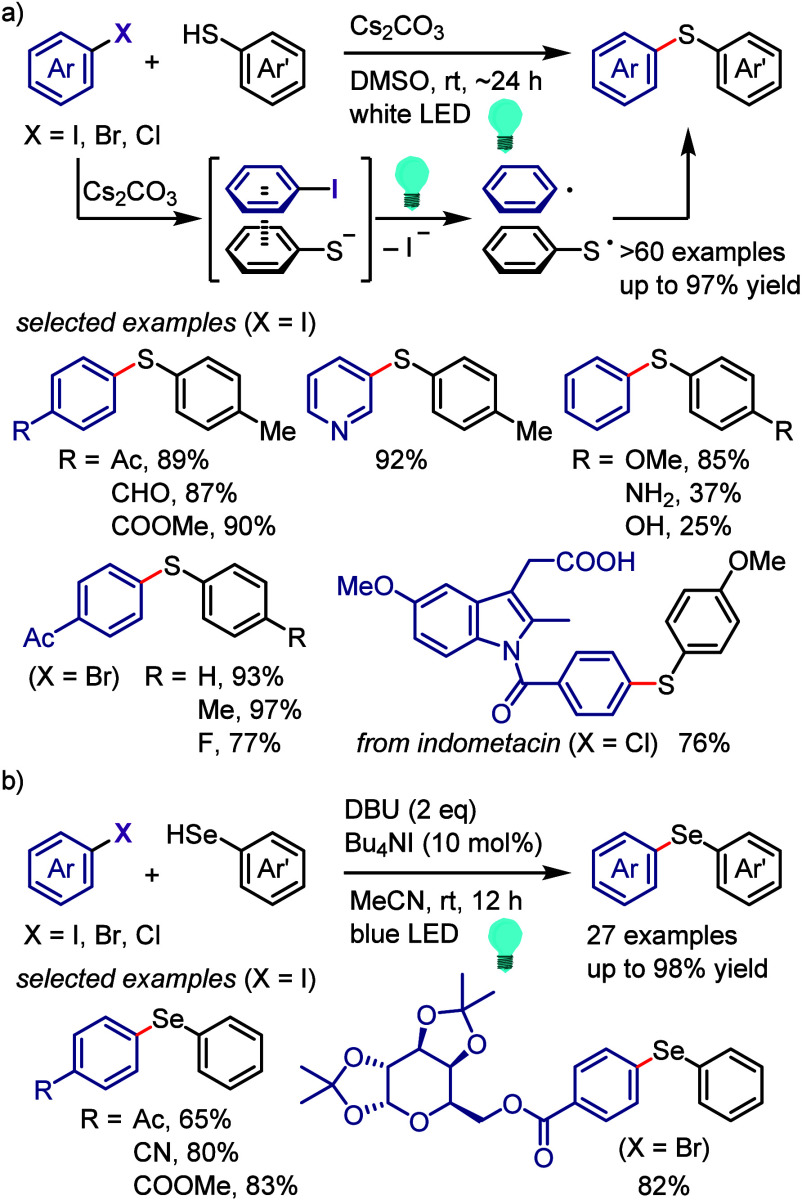
Photoinduced Coupling of Aryl Halides with Thio-/Seleno-phenols via an EDA Complex as the Key Intermediate

Sulfur anions exhibit versatile reactivity for promoting molecular transformations under transition metal-free, operationally simple, and green visible light irradiation conditions.^669−671^ Miyake et al. discovered C–S cross-coupling between aryl halides and aryl thiols under white LED irradiation even in the absence of reducing organic photoredox catalysts (Scheme 113a).^672^ Spectroscopic and theoretical studies indicated that the electron-poor aryl halide and electron-rich thiolate anion associated via π–π interactions and the formation of the EDA complex. Visible light irradiation of the EDA complex resulted in the generation of the thiyl and aryl radicals via the SET process, which coupled together to afford the final C–S cross-coupling product. This transition metal and photoredox-free pioneering work tolerated diverse functional groups and worked well with aliphatic/aromatic thiols and diverse aryl halides including heterocycles and pharmaceutical ingredients. Analogously, Yue et al. developed a convenient approach to construct the Ar–Se bond via in situ generation of the EDA complex between an aryl halide and aryl selenolate anion under blue light conditions (Scheme 113b).^673^ The reaction is compatible with a broad range of electron-deficient (hetero)aryl halides. Sekar et al. attempted C–S cross-couplings via halogen-bond assisted generation of EDA or CT complexes,^674^ followed by SET under visible light irradiation. This strategy was applied to the one-pot synthesis of heteroaryl thioethers via in situ formation of heteroaryl iodide, followed by cross-coupling with aryl/alkyl thiols in the presence of KO^*t*^Bu/DMSO under visible light conditions.^675^

**Scheme 114:**
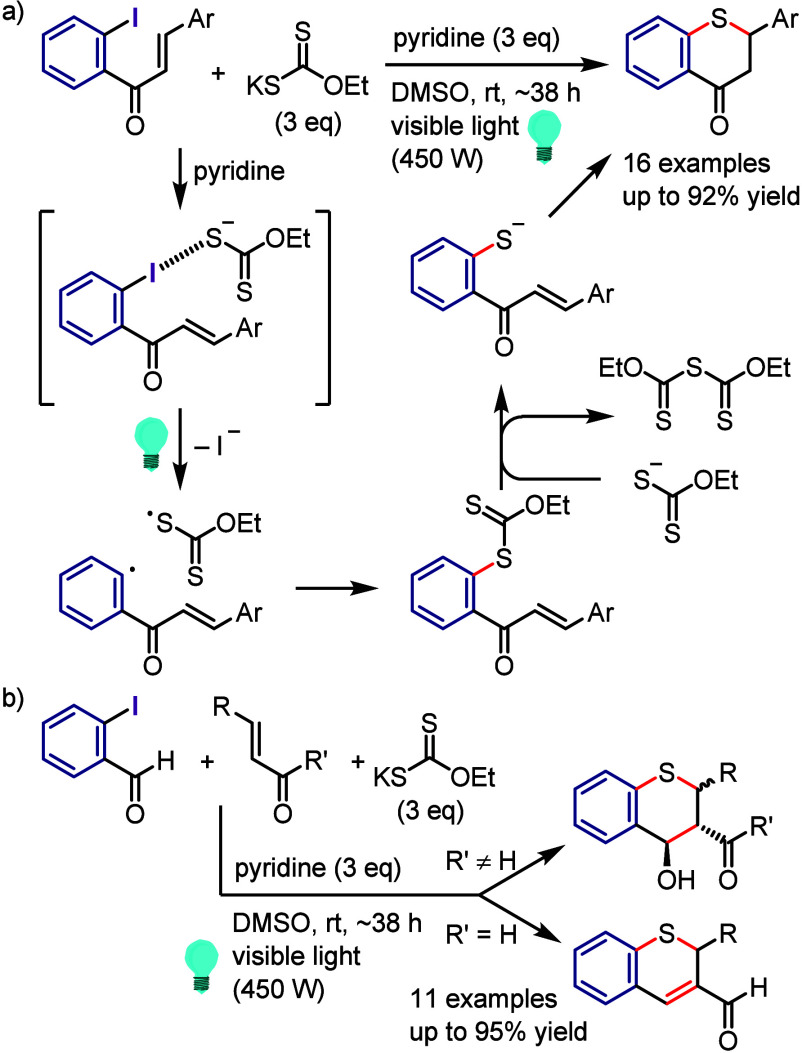
Visible Light Induced C–S Bond Formation via a Sequence of Halogen-Bond Interaction, EDA Complexation and Photoinduced SET Process

Xanthate was effectively incorporated as a dual sulfur surrogate and photocatalyst in the photoinduced cross-coupling reaction with 2-iodochalcones to produce thiochroman-4-one derivatives (Scheme 114a).^676^ Various control reactions, spectroscopic experiments, and DFT calculations were performed to sustain the interaction between the xanthate anion and aryl iodide via the formation of a halogen bond followed by the formation of the EDA complex. Visible light induced the SET from the xanthate anion to Ar–I to generate aryl and thiyl radicals, which underwent radical–radical cross-coupling to generate the xanthate ester. Thioester cleavage in the presence of xanthate anion furnishes thiolate, which undergoes intramolecular Michael addition to afford the final product. The reaction scope was expanded to a three-component reaction of xanthate, 2-iodobenzaldehyde, and chalcone or crotonaldehyde to synthesize the corresponding thiochroman-4-ol or thiochromene, respectively (Scheme 114b).

**Scheme 115:**
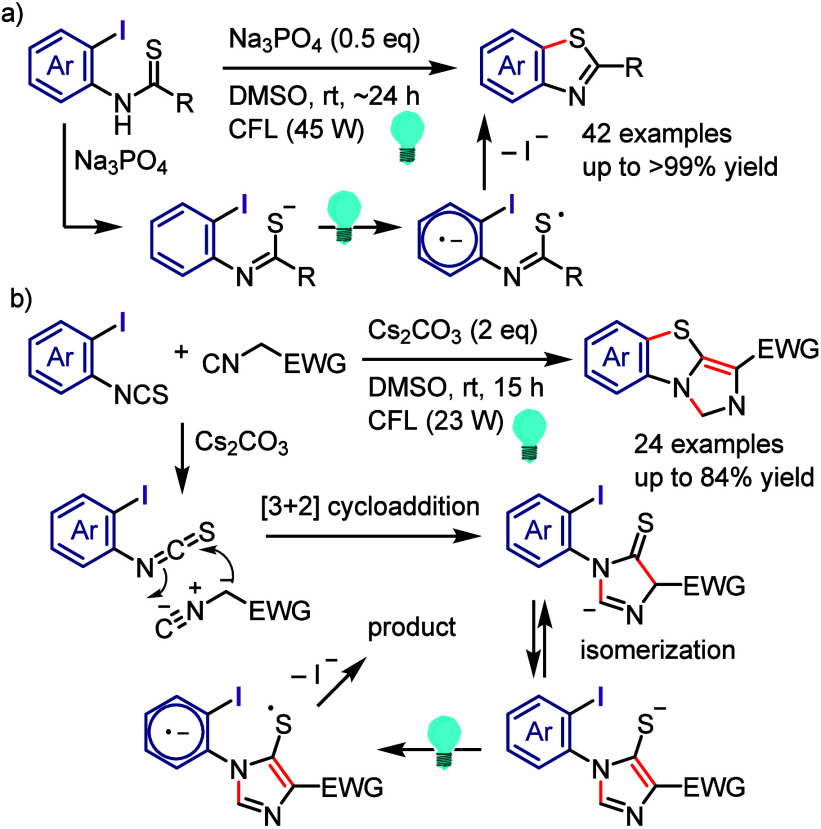
Photoinduced Ar–I Bond Dissociation and Intramolecular Cyclization

The importance of the EDA complexation of an aryl halide with a thiolate anion for the generation of aryl radical prompted Li et al. to design a modified strategy to synthesize benzothiazoles with high regioselectivity under oxidant-free conditions.^677^ Direct visible light photolysis of *ortho*-halothiobenzanilide under basic conditions afforded the related benzothiazole product via intramolecular dehalogenative C–S cyclization (Scheme 115a).^678^ In addition to CFL light, blue LED and natural sunlight were effective and afforded comparable yields even with gram scale experiments. Photoirradiation of the deprotonated starting material generates the excited state species, which undergoes intramolecular SET from the thiolate anion to the *N*-aryl ring followed by dehalogenative cyclization to finally afford the cyclization product. The cascade cyclization reaction of 1-iodo-2-isothiocyanatobenzene with 2-isocyanoacetate under visible light photolysis produces the corresponding benzo[*d*]imidazo[5,1-*b*]thiazole (Scheme 115b).^679^ The proposed mechanism begins with [3 + 2] cycloaddition in the presence of base to generate the imidazole intermediate, which isomerizes to the thiolate anion. An internal EDA complex forms between the thiolate anion and aryl moiety, followed by SET under light irradiation to generate the diradical intermediate. The cyclization that follows affords the final product.

**Scheme 116:**
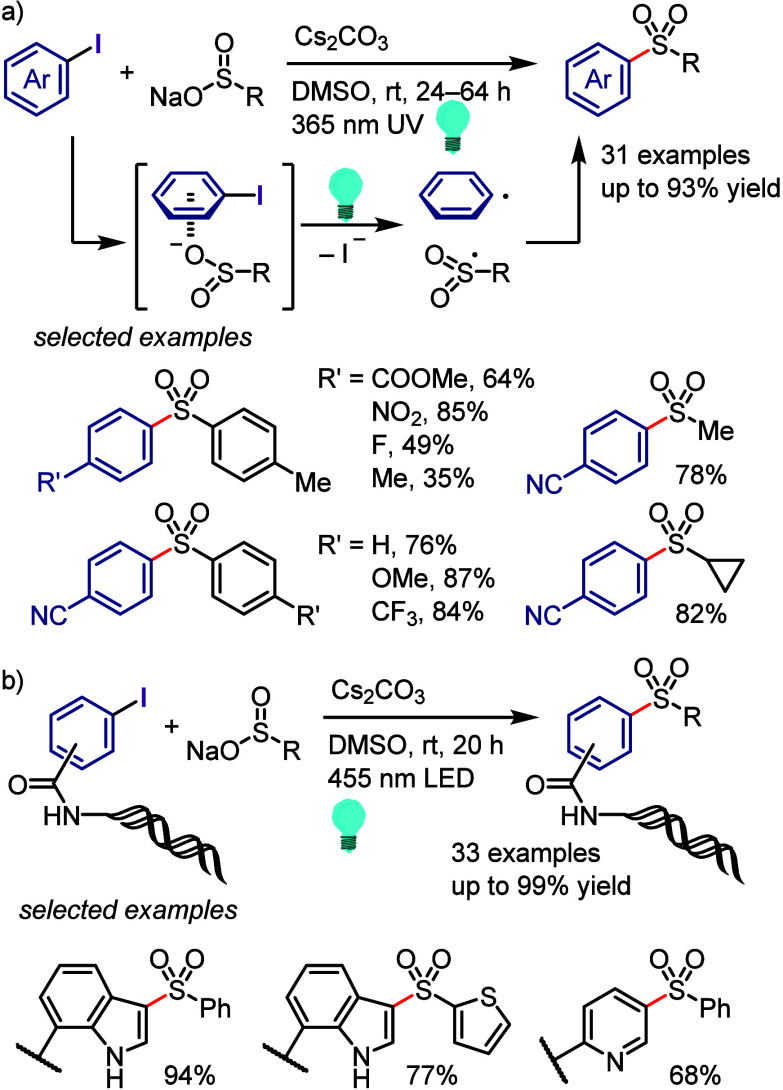
Light-Induced Cross-coupling of Sodium Sulfinate with Aryl Halides and Production of Sulfone

The UV-light promoted coupling of sodium sulfinate with an aryl halide in the presence of Cs_2_CO_3_ was reported by Yan and Zhang as a complementary strategy for the synthesis of sulfones (Scheme 116a).^680^ The UV–vis study indicated the formation of an EDA complex between the sulfinate anion and Ar–I. Thus, the proposed mechanism began with the formation of the EDA complex, followed by UV irradiation-promoted SET from the sulfinate anion to Ar–X; the complex then collapsed into an aryl and a sulfonyl radical. Subsequent radical–radical coupling afforded the cross-coupling sulfone product. This type of transformation is compatible with electron-rich/poor aryl sulfinate, alkyl sulfinate, and electron-deficient aryl halides; however, electron-rich aryl halides afforded lower yields even after a prolonged reaction time. The success of this protocol encouraged the same group to develop on-DNA radical cross-couplings using visible-light promoted SET within EDA complexes and reverse adsorption to support solid support (RASS) strategies.^681^ This type of transformation was applied, for example, to the cross-coupling of on-DNA-heteroaryl halides with alkyl/aryl sulfinates and synthesis of DNA-tagged heteroaryl sulfones (Scheme 116b).

**Scheme 117:**
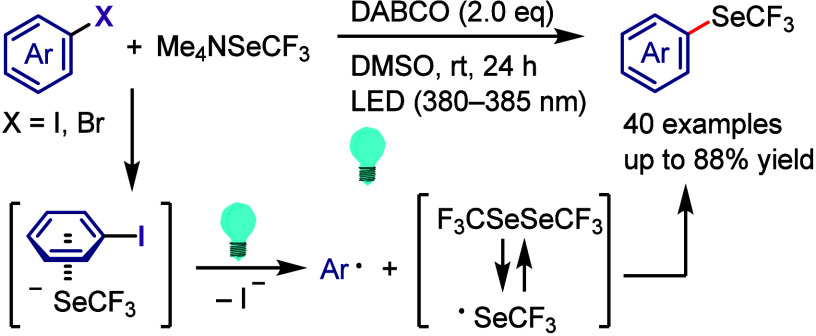
Photoinduced Cross-coupling of Aryl Halide with [Me_4_N][SeCF_3_]

Zhang et al. established an efficient method for the visible-light induced trifluoromethylselenolation of aryl halides with [Me_4_N][SeCF_3_] under benign conditions (Scheme 117).^682^ Mechanistic studies suggested the formation of an EDA complex between the electron acceptor aryl halide and electron donor selenium anion. Photoexcitation of the EDA complex led to intracomplex SET to finally afford an aryl and a trifluoromethyl selenium radical, which are rapidly cross-coupled to afford an aryl selenoether product. Alternatively, DABCO can also form the EDA complex and convert to an *N*-centered radical cation via the SET process, which oxidizes the trifluoromethyl selenium anion to generate the trifluoromethyl selenium radical. The coupling that follows, affords the final product.

#### 4.1.3 C–C Bond Formation

**Scheme 118:**
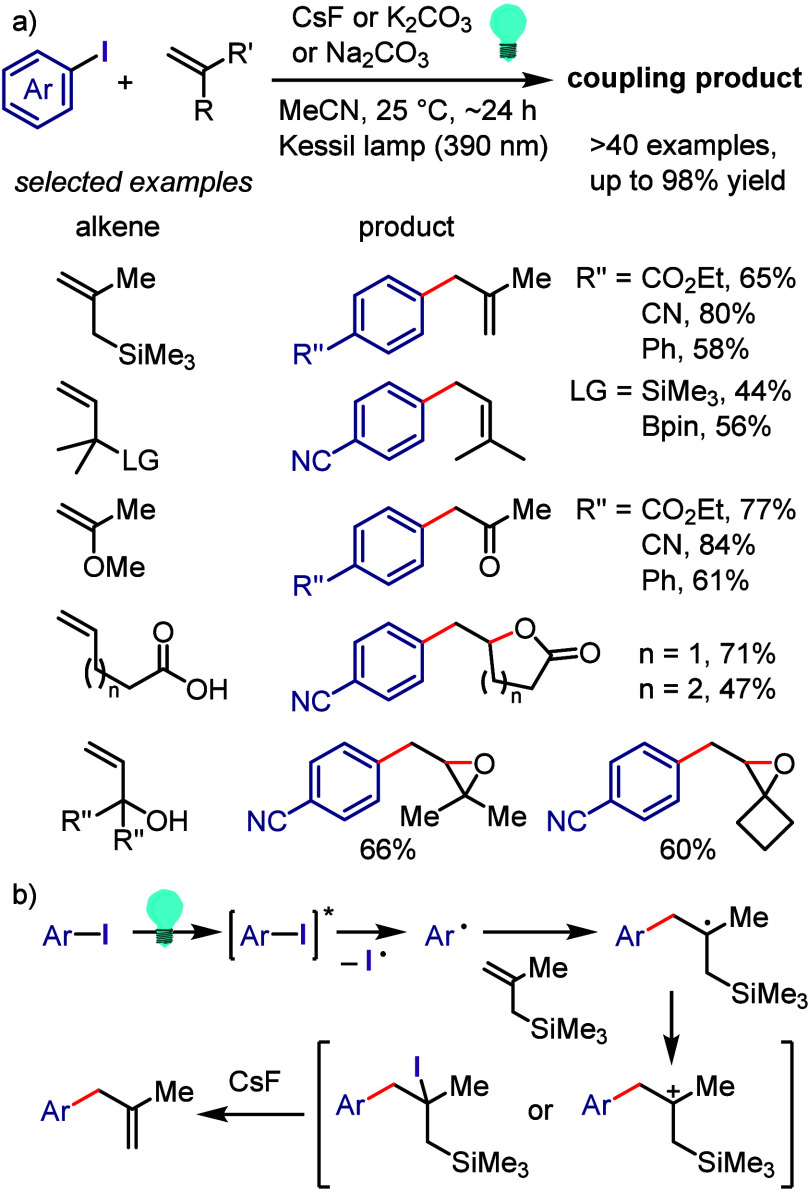
Photoinduced Cross-coupling of Aryl Iodide with Various Alkenes

Chiba et al. developed a process for the cross-coupling of aryl iodides with various alkenes under irradiation with violet light (390 nm) (Scheme 118a).^683^ The process is relevant for the synthesis of allyl, prenyl, acetomethyl, lactone, and spirocyclic epoxide derivatives of the corresponding arenes. The radical cascade reaction is initiated by the irradiation of Ar–I with violet light to transfer it to the photoexcited state, where it undergoes Ar–I bond homolysis to produce an aryl radical and atomic iodine (I^•^). For the reaction with allyl silane, the irreversible addition of an aryl radical to an alkene affords a β-silyl radical (Scheme 118b). Oxidation generates a β-silyl carbocation, which furnishes the allylation product after removal of the silyl group. A possible alternate pathway is that which occurs via the formation of an alkyl iodide followed by the elimination of the silyl group.

**Scheme 119:**
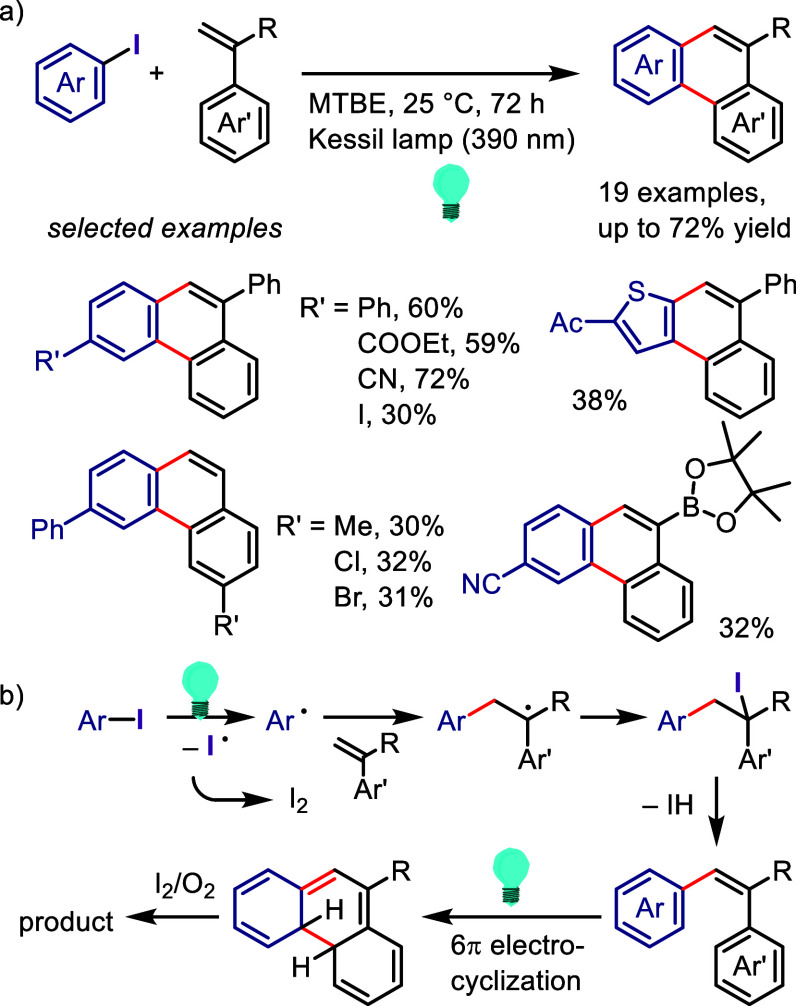
Photoinduced Arylation/Cyclization Cascade Generating Phenanthrene Derivatives

Parasram et al. used the results obtained by Chiba to design a cascade approach for the synthesis of phenanthrene derivatives via the photoexcitation of aryl iodides in the presence of styrenes (Scheme 119a).^684^ Unsymmetrical phenanthridines bearing useful functional groups were prepared under the reaction conditions which are applicable to gram scale synthesis. The reaction mechanism was postulated based on UV–vis studies and control experiments. Light-induced homolysis of the aryl iodide generated the aryl radical, which was captured by the styrene double bond to form the corresponding benzylic radical (Scheme 119b). Thereafter, iodination of the radical intermediate occurred followed by elimination of HI to generate a stilbene derivative. Subsequently, the photoinduced Mallory-type cyclization furnished dihydrophenanthrene, which subsequently oxidized in the presence of I_2_/air to afford the final product.

**Scheme 120:**
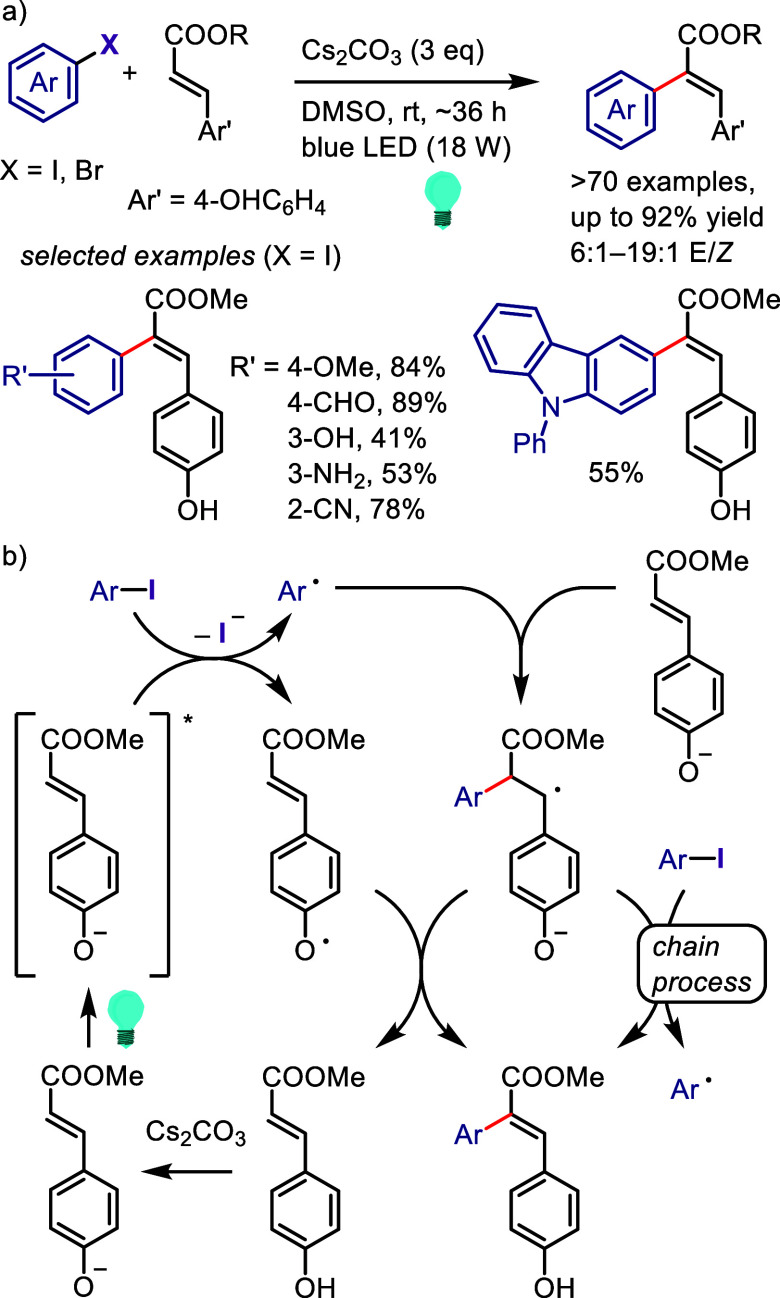
Vinylphenolate as Photoreductant and Heck-Type Reagent in Reaction with Aryl Halides to Afford Multisubstituted Alkenes

Xia et al. used the phenolate anion as a strong photoreductant catalyst in the oxyarylation of olefins with aryl halides and TEMPOH under visible light irradiation.^685^ In addition, the designed phenolate anion photocatalyst was used for the intramolecular dearomative spirocyclization of 4-hydroxyaryl-tethered-2-haloarenes and synthesis of spirocyclohexadienones.^686^ A photocatalyst-free process was designed using vinylphenolate anions as both the reaction component and redox mediator for the reductive activation of aryl halides under mild conditions (Scheme 120a).^687^ The Heck-type arylation of vinylphenols with aryl halides under visible light irradiation conditions afforded the corresponding multisubstituted alkenes. A plausible mechanism begins with the excitation of the phenolate anion under light irradiation (Scheme 120b). The redox potential of the excited species (−2.48 V vs SCE) implies it can undergo SET with Ar–X to afford phenoxy and aryl radicals. The generated aryl radical reacts with the vinylphenol to generate a radical anion intermediate, which transfers a hydrogen atom to the phenolate to afford the desired product. Alternatively, the radical anion intermediate undergoes a SET to the other Ar–X to afford the product along with the aryl radical, which propagates the chain process.

**Scheme 121:**
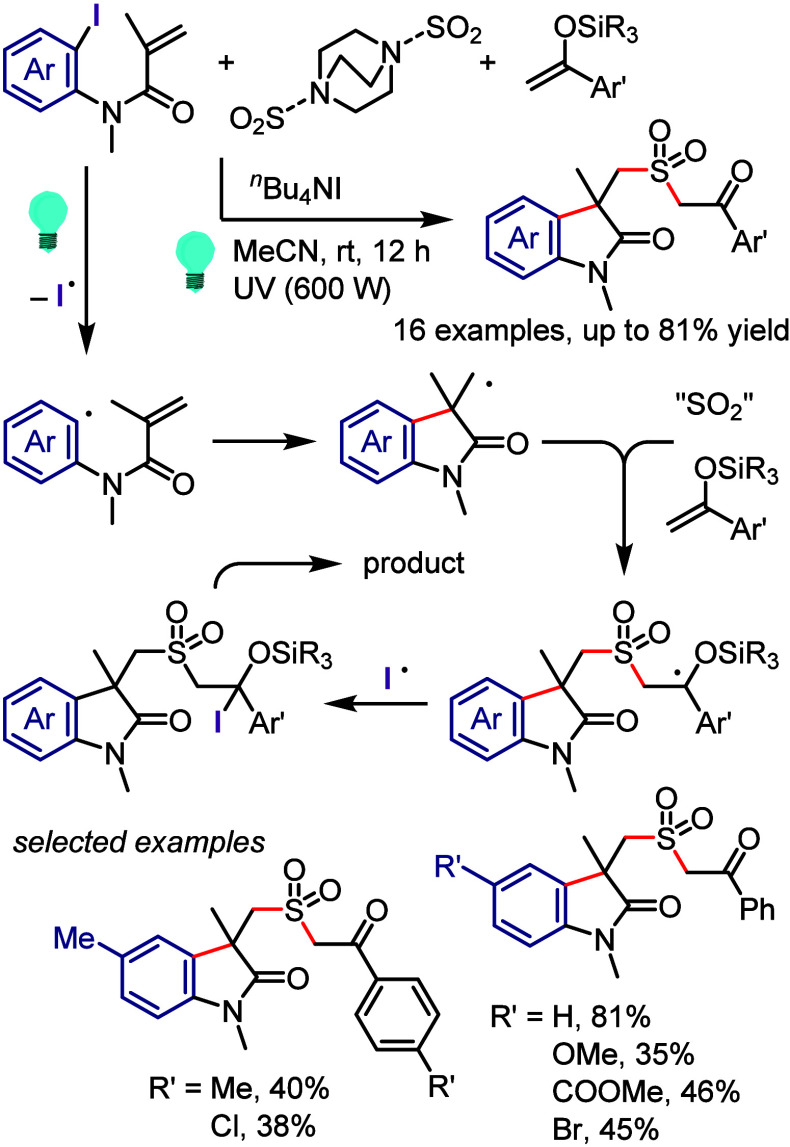
Photoinduced Synthesis of Sulfonated Cyclic Compounds *via* SO_2_-Insertion

Wu et al. synthesized sulfonated cyclic compounds by reacting aryl iodides tethering *N*-methylmethacrylamide with silyl enolate and an SO_2_ source (DABCO(SO_2_)_2_) under ultraviolet irradiation (Scheme 121).^688^ The reaction mechanism is initiated by UV-induced Ar–I bond cleavage to generate an aryl radical, which undergoes intramolecular 5-*exo*-cyclization to afford the cyclized alkyl radical. Further sulfonation and subsequent trapping by the silyl enolate, followed by radical–radical coupling with the iodine radical generate an alkyl iodide intermediate, which undergoes desilylation to afford the final product.

**Scheme 122:**
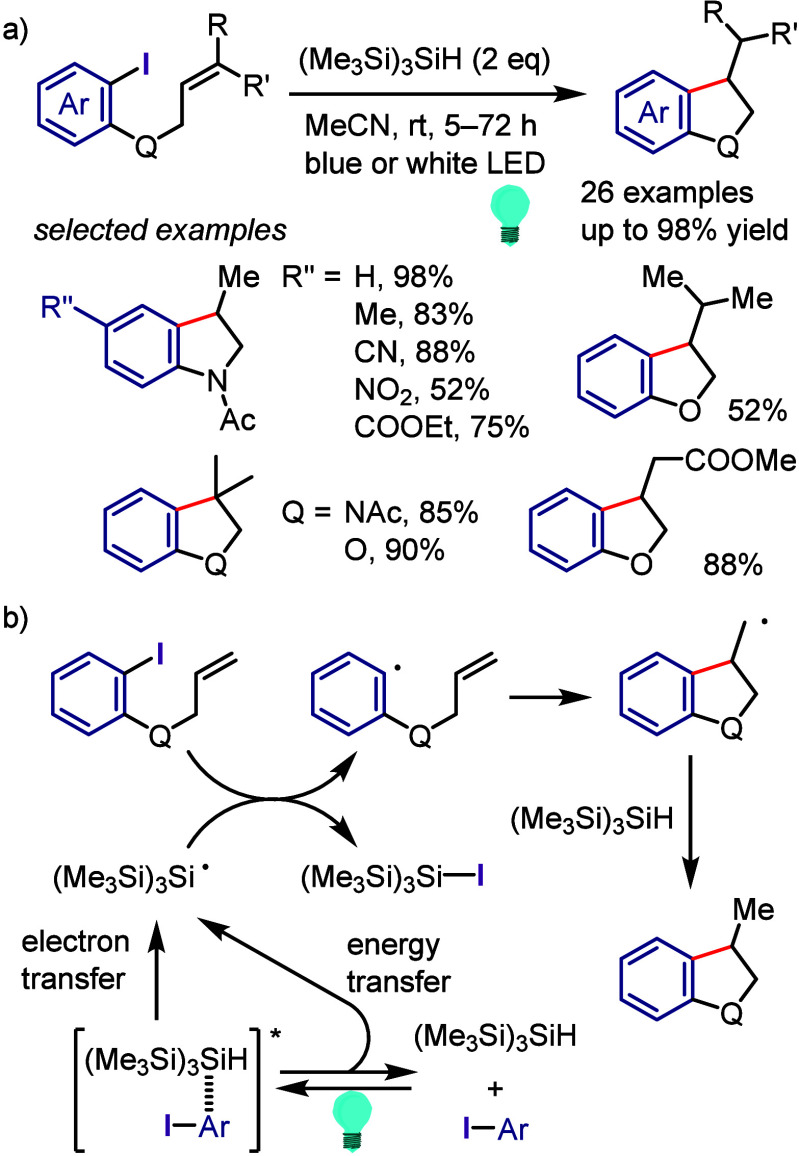
(TMS)_3_SiH/Visible Light Promoted Intramolecular Reductive Cyclization

Paixão et al. developed an intramolecular reductive cyclization protocol to convert *N*-propargyl-2-halobenzenesulfonamides and *N*-protected 2-halophenylacrylamides into the related *N*-heterocycle indoles and oxindoles, respectively, in the presence of TMS_3_Si-H and visible light as an efficient promoter system.^689^ Further extension was performed for the synthesis of highly substituted indolines and 2,3-dihydrobenzofurans from the related *N*-allyl-*N*-(2-iodophenyl)acetamide and 1-allyloxy-2-iodobenzene, respectively (Scheme 122a).^690^ The reaction mechanism begins with irradiation of the reaction mixture with visible light and generation of a reactive excited complex as an EDA complex or exciplex (Scheme 122b). Either energy or electron transfer from the generated complex leads to homolytic cleavage of the Si–H bond and generation of a silicon radical, which abstracts the iodine from the starting aryl iodide to generate an aryl radical. Intramolecular radical cyclization followed by H-abstraction from TMS_3_Si-H affords the final product.

**Scheme 123:**
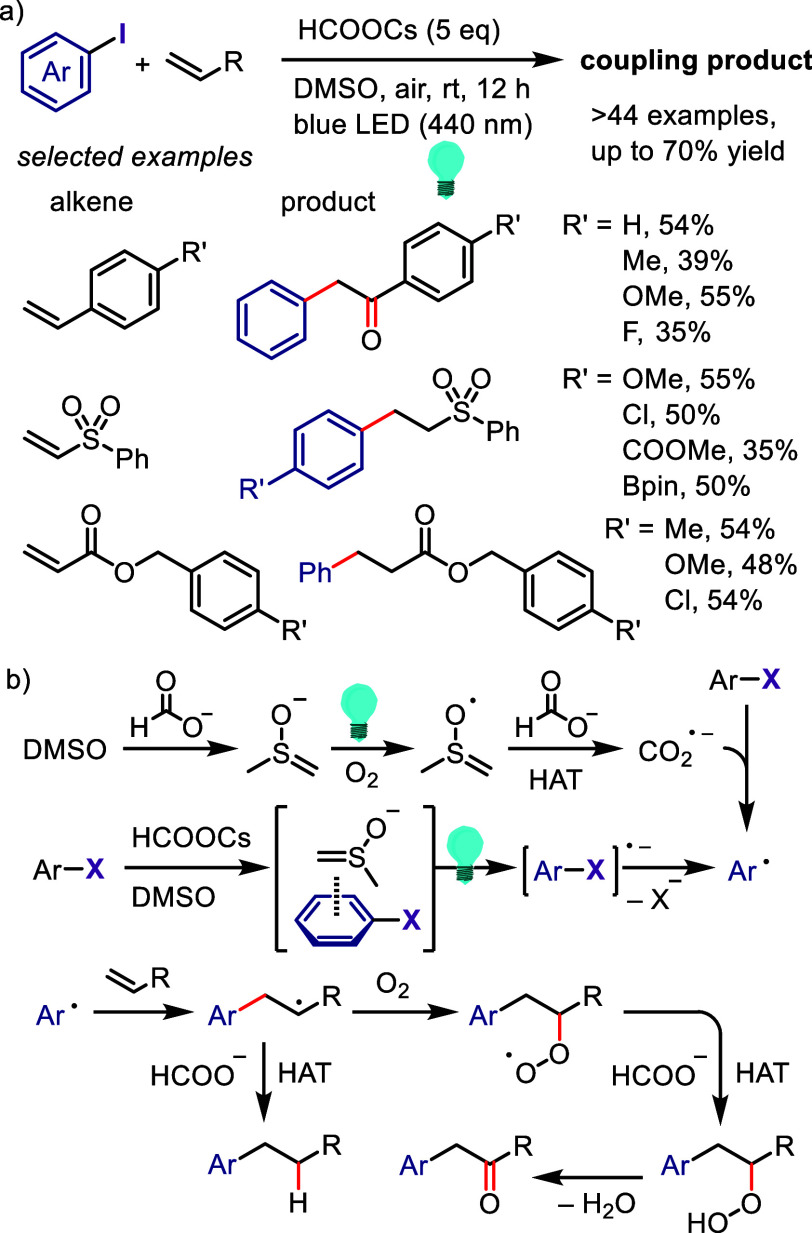
Photoreduction of Aryl Halides with O_2_/Format Salt/DMSO System

The carbon dioxide radical anion CO_2_^•–^ has emerged as a strong single electron donor to reduce recalcitrant aryl halides under photocatalytic conditions.^691−693^ Recently, Hou et al. used their oxygen-format salt-DMSO system for photopromoted oxyarylation and hydroarylation of alkenes with aryl halides (Scheme 123a).^694^ The hydroarylation pathway required electron-deficient alkenes for reaction with electronically and sterically diverse (hetero)aryl halides under the same conditions. Mechanistic investigations indicated the importance of CO_2_^•–^ and the EDA complex that forms between the dimsyl anion and aryl halide to generate an aryl radical under blue light irradiation (Scheme 123b). For the reaction with electron-rich styrene, the generated aryl radical is trapped by styrene, which reacts with O_2_ and subsequently abstracts a hydrogen atom from the formate salt to afford the final product after extrusion of H_2_O. By contrast, the reaction with an electron-deficient alkene does not involve the reaction with O_2_ before the HAT process.

**Scheme 124:**
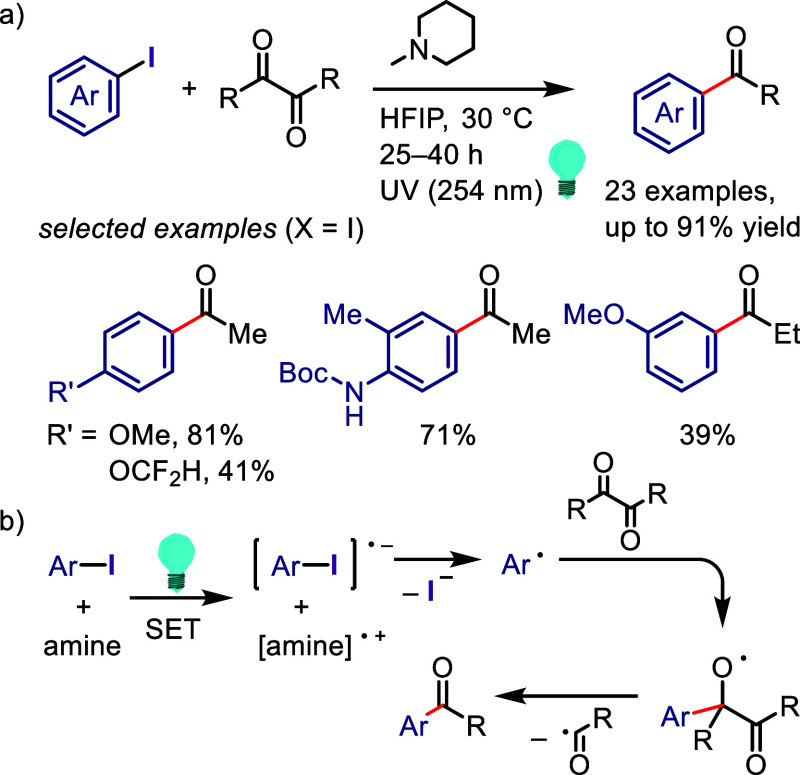
Photopromoted Cross-coupling of Aryl Halides with Diketones and the Formation of Aryl Ketones

Preparation of ketones under transition metal-free conditions by the direct addition of the generated radical species to carbonyl compounds is challenging owing to the formation of the thermodynamically unfavorable alkoxy radical and possibility of competing H-abstraction processes. Li et al. successfully designed a photochemical protocol for the synthesis of aryl ketones by the direct coupling of aryl halides with dicarbonyl compounds under UV-light/base conditions (Scheme 124a).^695^ Various aryl halides and symmetrical diketones were used to deliver the related aryl ketone; however, the incorporation of 2,3-pentanedione as an unsymmetrical diketone afforded a mixture of aryl ketones. Experimental studies indicated the importance of UV-light and using *N*-methylpiperidine as a base to generate the aryl radical via the SET process (Scheme 124b). Addition of the aryl radical to the diketone produced the corresponding alkoxy radical, which eliminated the radical to afford the final aryl ketone.

**Scheme 125:**
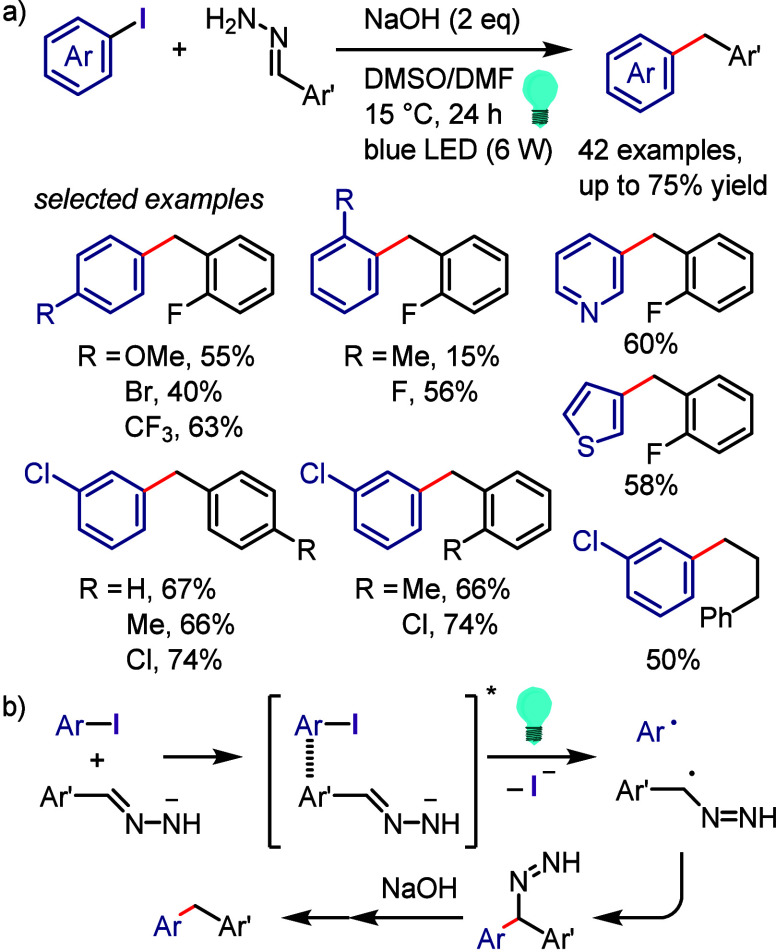
Light-Promoted Coupling of Aryl Iodide with Hydrazone and the Formation of Diarylmethane

The same group established the photoinduced cross-coupling of hydrazones with aryl iodides to form diarylmethanes under benign conditions (Scheme 125a).^696^ Mechanistic and DFT studies supported the formation of an EDA complex via π–π interactions between the deprotonated hydrazone and aryl iodide (Scheme 125b). The photoinduced SET process generated aryl and benzylic radicals, which undergo radical–radical cross-coupling to generate the benzhydryldiazene intermediate. Subsequent deprotonation liberates N_2_, followed by protonation by solvent/H_2_O to afford the final product.

**Scheme 126:**
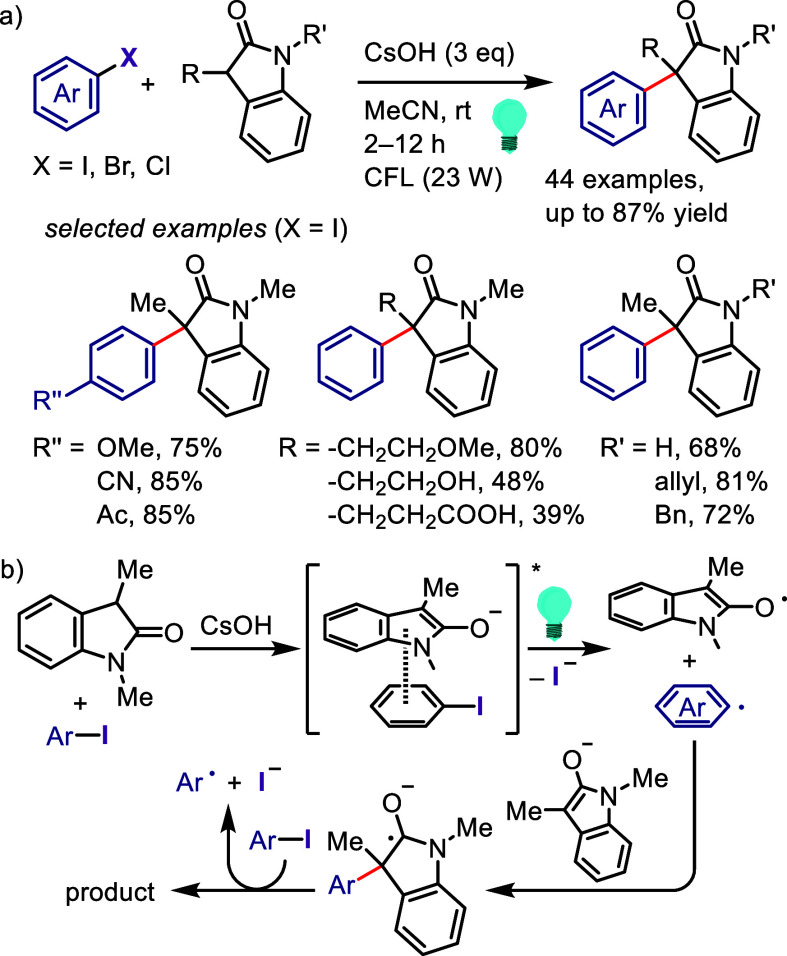
Oxindole Enolate Induced C-3 Arylation of Oxindole with Aryl Halide *via* EDA Complex Formation

Xia et al. designed a photochemical approach for the C-3 arylation of oxindole with aryl halides via the in situ generation of oxindole enolate as a strong electron donor (Scheme 126a).^697^ Mechanistic studies revealed the contribution of oxindole enolate as an electron donor in EDA complex formation; furthermore, the quantum yield of the process (Φ = 11.1) indicated the possibility of radical chain propagation (Scheme 126b). Thus, the reaction begins with the formation of an EDA complex, which, under visible light irradiation promotes the SET process to generate an oxindole and aryl radical. Trapping the generated aryl radical by the enolate affords a ketyl radical, which is a strong reductant (*E*_p_^red^ = −2.31 V vs Ag/Ag^+^ in MeCN) and transfers a single electron to the aryl halide to generate the final product and propagates the radical chain process.

**Scheme 127:**
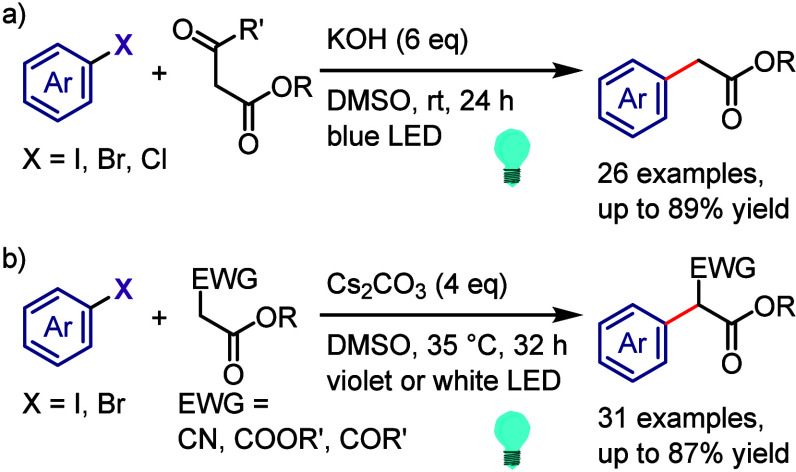
Photoinduced Arylation of Activated Methylene Compounds with Aryl Halide

Jiang and Karchava independently used the EDA complex photoactivation strategy for Csp^3^-arylation of activated methylene compounds with aryl halides under photoredox conditions.^698,699^ KOH/DMSO/blue LED conditions were designed by Jiang et al. to activate cross-coupling of various β-keto esters with electron-deficient aryl halides and the in situ deacetylation reaction for the synthesis of α-aryl ester derivatives (Scheme 127a). Milder reaction conditions were devised by Karchava to tolerate a wider substrate scope of activated methylene compounds, electron-deficient aryl halides, and heteroaryl halides (Scheme 127b). Mechanistic investigations supported the aggregation of the deprotonated active methylene and aryl halide via EDA complex formation and generation of aryl and alkyl radicals under visible light irradiation.

**Scheme 128:**
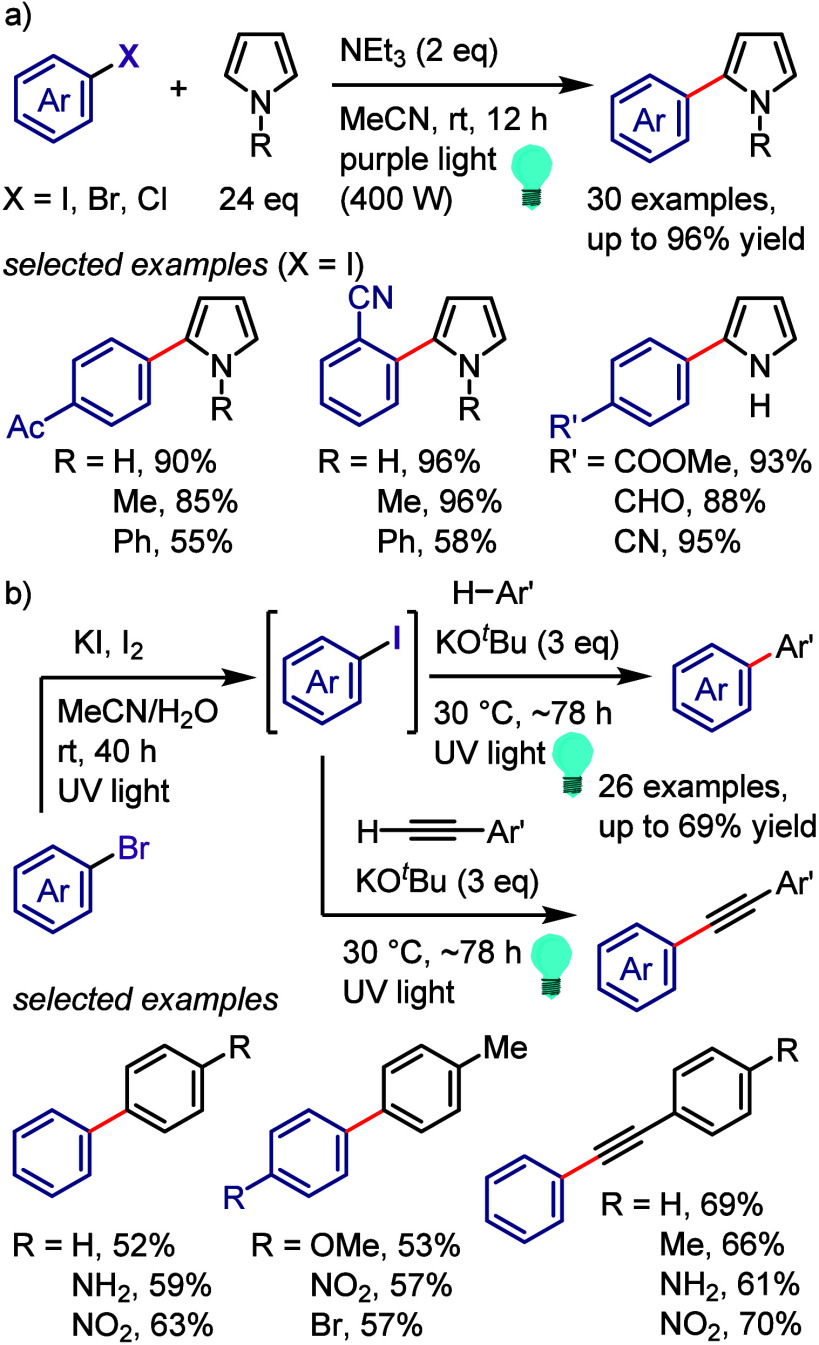
Photoinduced C–H Arylation of Heteroarenes and Terminal Alkynes

The cross-coupling reaction of electron-deficient haloarenes with electron-rich (hetero)arenes under solely violet light irradiation was studied by Yeow and Chiba to understand the crucial role played by photoinduced SET from electron-rich (hetero)arenes to photoexcited aryl halides for the formation of the desired biaryl products.^700^ Additionally, Fang et al. studied the interaction between aryl halides and a Lewis base (Et_3_N) during the activation of aryl halide bond dissociation under visible light irradiation (Scheme 128a).^701^ Base and light are essential for this type of activation and the generated aryl radical was confirmed by trapping with TEMPO radical trapping reagent. This protocol was applied to the C–H arylation of pyrroles with various deactivated aryl halides and radical-initiated polymerization. Bhalla et al. developed stepwise and one-pot protocols to synthesize biaryls and diarylalkynes via a sequence of UV-light mediated aromatic Finkelstein reactions to convert aryl bromides to aryl iodides followed by UV-light/KO^*t*^Bu promoted cross-coupling with unactivated arenes and terminal alkynes, respectively (Scheme 128b).^702^ The combination of UV-light and KO^*t*^Bu is essential to activate aryl iodide. These reactions tolerate electronically different aryl halides and C–H arenes in addition to phenyl/pyridyl-acetylenes to afford the target products with complete chemo- and regio-selectivity. The cross-coupling mechanism initiated by the photoirradiation of aryl iodides in the presence of KO^*t*^Bu, which induced the SET to the aryl iodide and generation of the reactive aryl radical for coupling with arenes or alkynes.

**Scheme 129:**
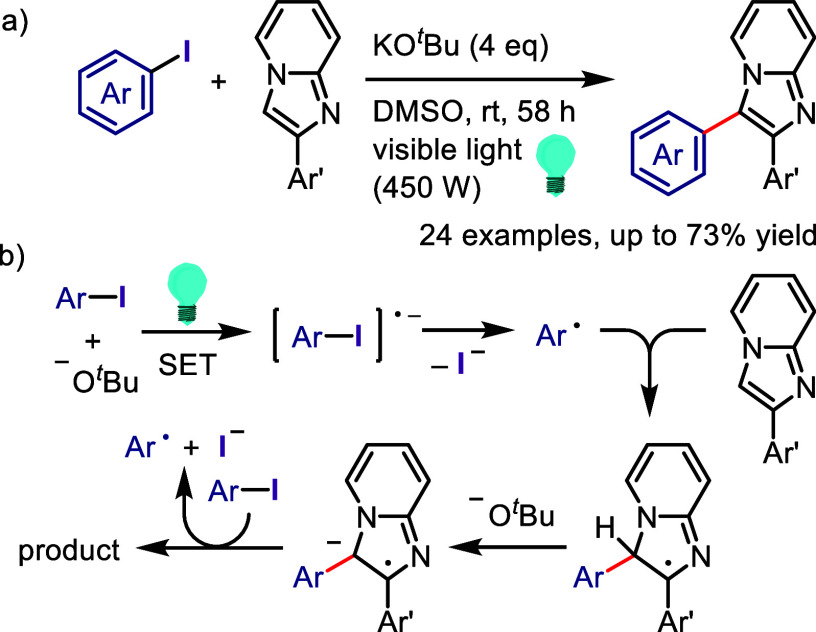
Halogen Bond Promoted Aryl Iodide for C-3 Arylation of Imidazopyridines

Sekar et al. extended their interest in halogen-bond activated aryl halides under visible light irradiation and designed a regioselective C–H arylation of 2-arylimidazo-[1,2-*a*]pyridines with aryl iodides (Scheme 129a).^703^ The proposed mechanism begins with the generation of aryl radicals induced by KO^*t*^Bu under light irradiation; the aryl radical couples with imidazopyridine to generate the radical intermediate (Scheme 129b). Deprotonation by KO^*t*^Bu and the subsequent SET process with aryl iodide affords the final product and the aryl radical.

**Scheme 130:**
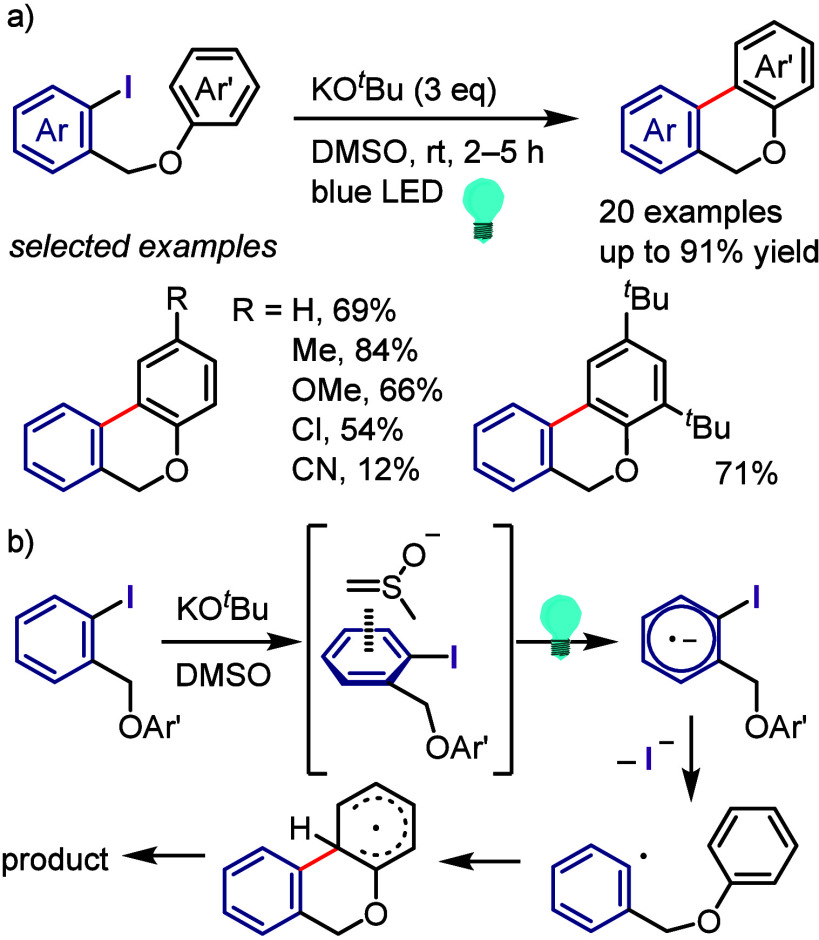
Intramolecular Homolytic Aromatic Substitution under Blue Light Irradiation

A combination of KO^*t*^Bu, DMSO, and light suffices to promote the intramolecular C–H arylation of (2-halobenzyl) phenyl ether and (2-halophenyl) benzyl ether and formation of the related benzo[*c*]chromenes and dibenzo[*c*,*f*]chromenes, respectively (Scheme 130a).^704^ UV–vis studies and DFT calculations proposed the in situ formation of the EDA complex (Scheme 130b). The reaction of DMSO with KO^*t*^Bu generates the dimsyl anion, which forms an EDA complex with the substrate. Photoirradiation leads to an excited state with a charge transfer character to generate the aryl radical, which undergoes 6-*endo*-cyclization followed by deprotonation and the subsequent SET to afford the final product.

**Scheme 131:**
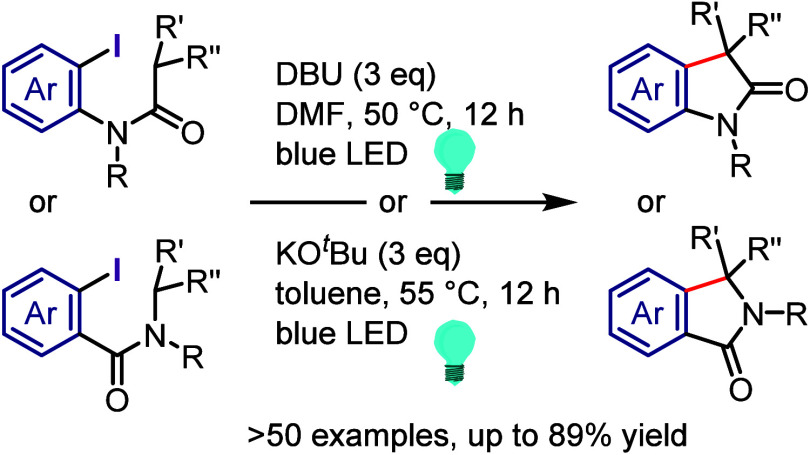
Base and Solvent Anion Activated Ar–X Bond Photodissociation and Formation of Heterocycles

Chen et al. photoactivated *ortho*-(5-Csp^3^-H)-tethered aryl halides via the in situ formation of EDA complexes for cascade reactions and the formation of various heterocyclic skeletons (Scheme 131).^705,706^ Various *ortho*-anilide aryl halides and *ortho*-halobenzamides were reacted under two different photochemical conditions for the formation of oxindole, isoindolinone, and phenanthridinone derivatives. Experimental and theoretical studies supported three weak interactions that occurred via the participation of DBU or KO^*t*^Bu, which serves as the electron donor for the complexation of aryl halides and formation of photoactive EDA complexes. Blue light irradiation of these complexes promoted the SET and generation of aryl radicals which underwent 1,5-HAT followed by radical cyclization to finally afford the desired product.

#### 4.1.4 Hydration and Hydroxylation of Aryl Halides

**Scheme 132:**
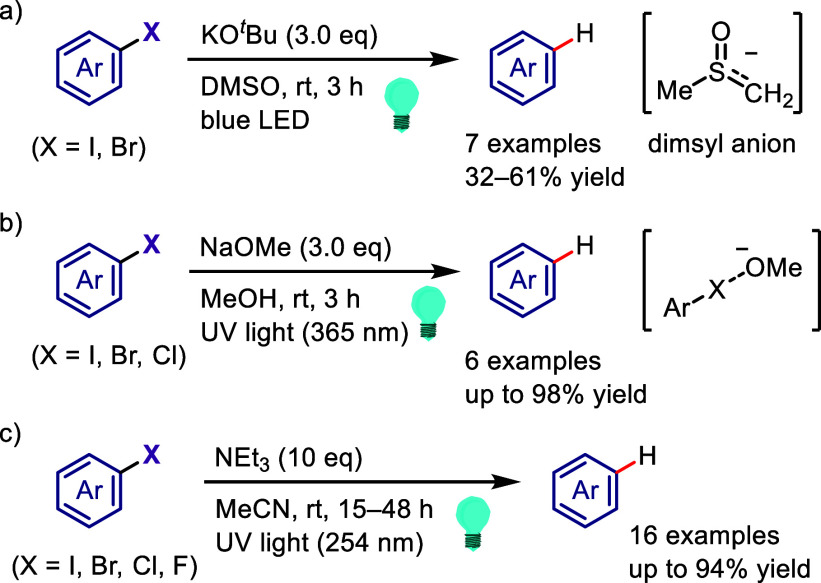
Light/Additive-Induced Hydrodehalogenation of Aryl Halides

Halogenated aromatic compounds are persistent organic pollutants as their scaffolds are hydrophobic and nonbiodegradable and consequently accumulate in the environment and living organisms.^644,707^ Thus, these highly carcinogenic compounds cause severe environmental and health problems. Replacing C–halogen with C–H or any other benign group is an important organic transformation in organic synthesis as it provides a pathway for detoxification/degradation of environmentally hazardous organic halides. Hydrodehalogenation and dehalogenative deuteration of aryl halides are desired processes to modify the properties of the candidate pharmaceutical and agrochemical compounds; the activity and metabolic stability can be improved, and the toxicity may be reduced.^708−712^ Interestingly, Rossi et al. used a combination of visible light, KO^*t*^Bu, and DMSO solvent for the hydrodehalogenation of aryl halides under benign conditions (Scheme 132a).^713^ Alternatively, Lanterna and Scaiano et al. reported the importance of halogen bond interaction between electron-deficient haloarenes and the methoxide base for the photodissociation of the Ar–X bond and generation of the aryl radical; subsequently, the C–H arene was produced under mild UVA irradiation conditions (Scheme 132b).^714^ Ryu and Wu et al. used Et_3_N as the electron- and hydrogen-donor to reduce various aryl halides to their related arenes under UV-light or thermal conditions (Scheme 132).^715^ By using a flow photomicroreactor with DABCO (1.5 equiv) as a base, the hydrodehalogenation reaction was complete after 20 min and afforded the related C–H product in quantitative yield.

**Scheme 133:**
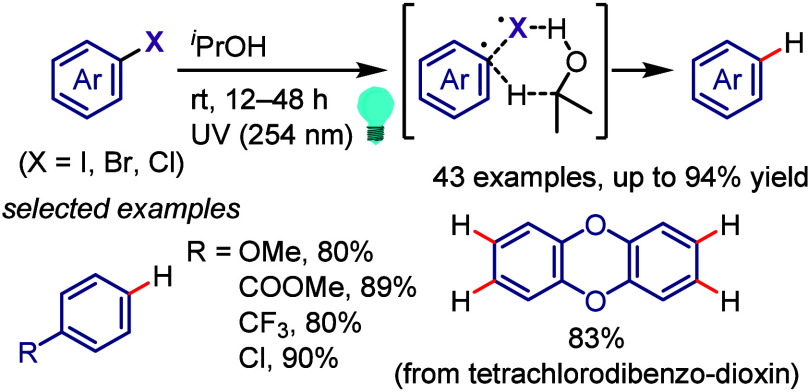
MPV-Type Reduction of Aryl Halides with Isopropanol under UV-Light

The Meerwein-Ponndorf-Verley (MPV) reduction of ketones to the related alcohols via hydrogen atom transfer from isopropanol motivated Zeng et al. to design a hydrodehalogenation process for aryl halides via the formation of a 6-membered ring transition state between the excited aryl halide and isopropanol (Scheme 133).^716^ The reaction of the aryl halide with isopropanol (as reducing reagent and solvent) under UV-light irradiation afforded the corresponding arene. Mechanistic studies and DFT calculations indicated (i) an inappropriate free radical mechanism, (ii) HAT from the C2 of isopropanol and (iii) an intramolecular pathway involving a simultaneous HAT and Ar–X bond cleavage via a 6-membered transition state.

**Scheme 134:**
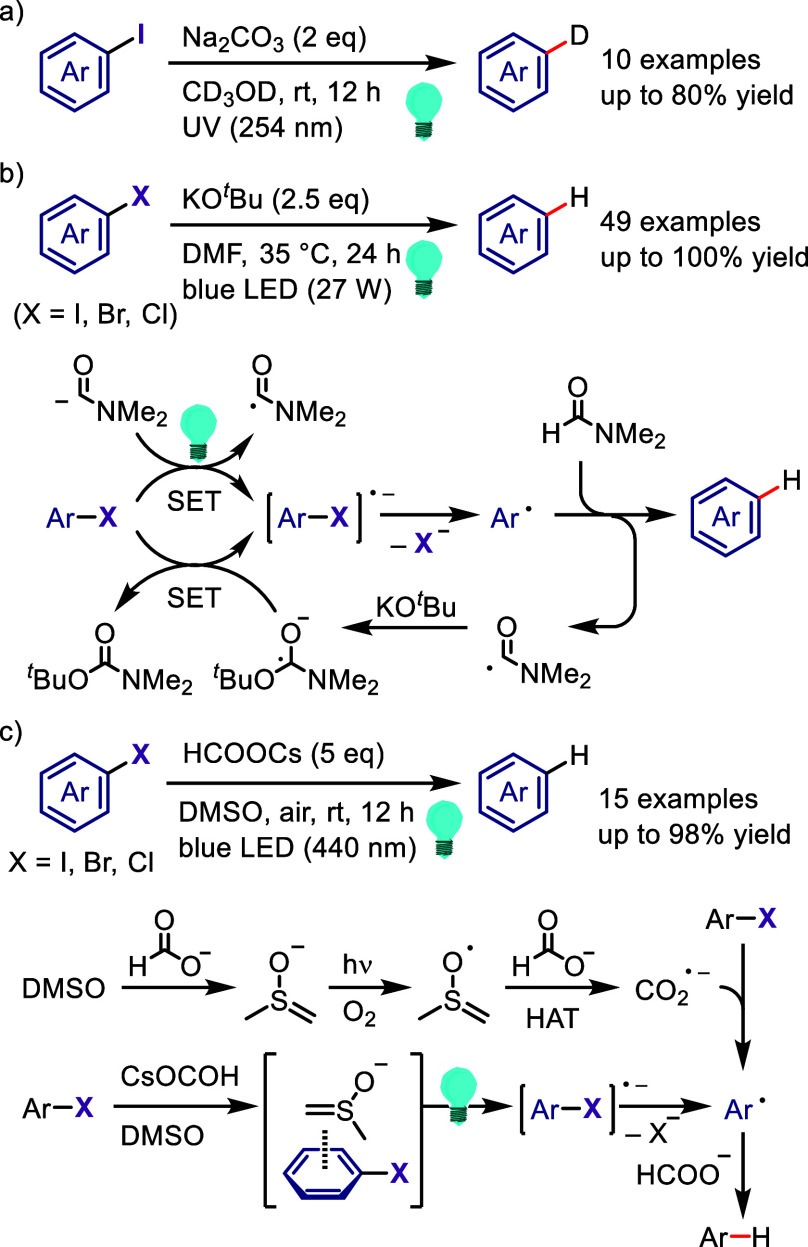
Hydrogenation/Deuteration of Aryl Halides via Photoinduced Ar–X Bond Cleavage

Yao et al. developed a direct reduction process for the hydrodefunctionalization and/or defunctionalization deuteration of functionalized arenes (Scheme 134a).^717^ Particularly, aryl iodides were subjected to deiodination deuteration under UV-light in the presence of CD_3_OD/Na_2_CO_3_ and formed deuterated arenes. The proposed mechanism involves the HAT process from the deuterated solvent to the aryl radical, which generated the product. Similarly, Qu and Kang used visible light/KO^*t*^Bu/DMF as a successful combination for hydrodehalogenation (Scheme 134b).^718^ DMF served as a hydrogen source as indicated by performing a control experiment using DMF-*d*_*7*_ as a solvent to afford the related deuterated product. The rational mechanism begins with deprotonated DMF transferring a single electron to Ar–X under visible light irradiation to generate the aryl radical, which abstracts a hydrogen atom from DMF to afford the product and the DMF radical. This radical species undergoes deprotonation by KO^t^Bu followed by the single electron reduction of the aryl halide to furnish the carbamate. Hou and Li et al. reported a hydrodehalogenation that uses the oxygen-format salt-DMSO system (Scheme 134c).^694^ The reaction involves the reduction of an aryl halide by CO_2_^•–^, which is generated via the HAT process between formate and the dimsyl radical. The thus-generated aryl radical abstracts a hydrogen atom from formate to afford the final product. Alternatively, the aryl radical can also be generated via the EDA complex between the dimsyl anion and aryl halide.

**Scheme 135:**
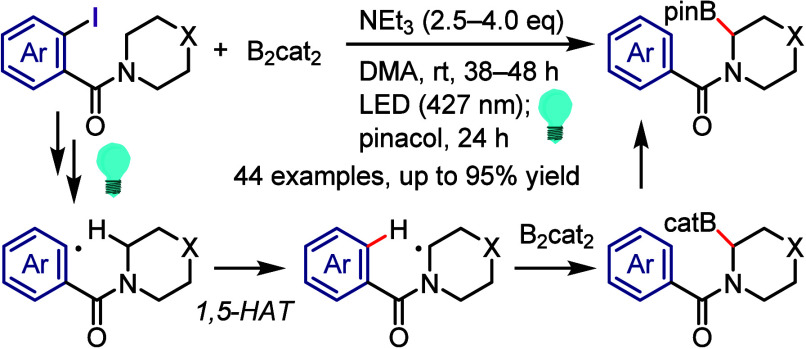
α-Csp^3^-H Borylation of *N*-(2-Iodobenzoyl)amine via In Situ Generation of Radical Translocating Group

Gevorgyan et al. used the photoinduced hydrodehalogenation reaction for an α-C–H borylation involving the 1,5-HAT process (Scheme 135).^719,720^ The reaction of an amine derivative bearing a 2-iodobenzoly group with *bis*(catecholato)diboron afforded the corresponding α-borylated amine derivative. The photoinduced generation of the aryl radical followed by 1,5-HAT generates an α-aminoalkyl radical, which after being trapped by the boronate ester afforded the catecholboronate. Transesterification with pinacol delivered the desired pinacol boronate product.

**Scheme 136:**
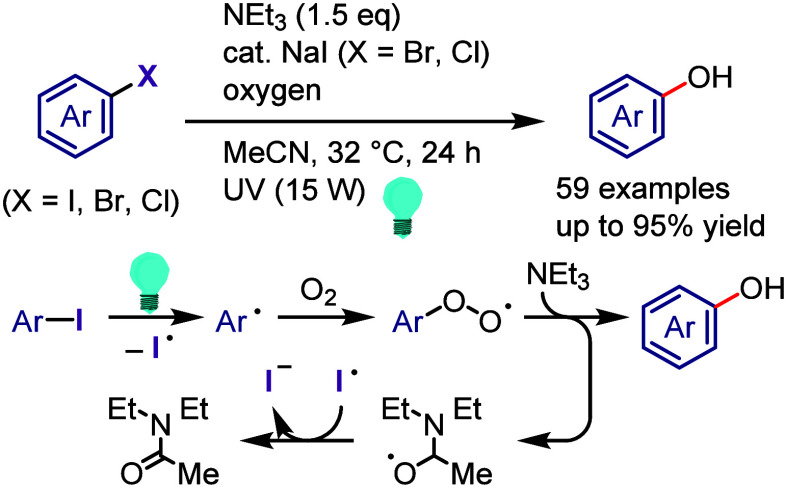
Hydroxylation of Aryl Halides with O_2_ under UV Irradiation

The viability of the homolytic cleavage of the Ar–X bond under ultraviolet irradiation enabled Liu et al. to design a new strategy for the hydroxylation of aryl halides via in situ trapping of the generated aryl radicals by molecular oxygen (Scheme 136).^721^ The addition of a catalytic amount of NaI enabled the recalcitrant aryl bromide/chloride substrates to react effectively under the UV conditions.^722,723^ Aryl halides with diverse functional groups and bioactive moieties were amenable to reacting under the irradiation conditions. The postulated mechanism begins with the homolysis of the Ar–X bond under UV irradiation to generate the aryl radical.^721^ For aryl bromide/chloride, the liberated Br^•^/Cl^•^ are reduced with the I^–^ of NaI. Trapping the generated aryl radical by O_2_ and subsequent reaction with Et_3_N affords the phenol derivative. The thus-generated oxygenated triethyl amine reduces the iodine radical to afford acetamide and the iodide anion.

#### 4.1.5 Borylation and Phosphinylation of Aryl Halides

**Scheme 137:**
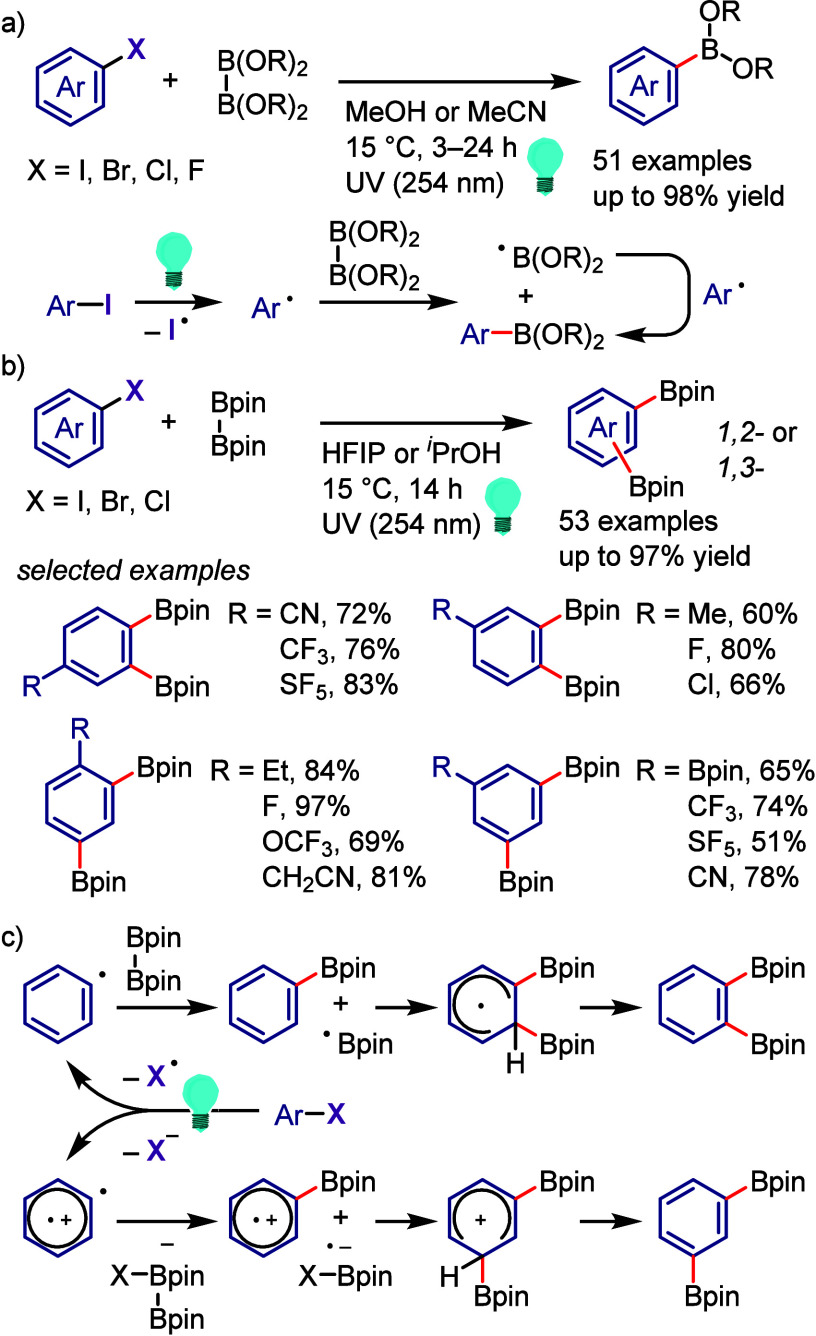
Additive-Free Photoinduced Borylation

Larionov et al. achieved the additive-free UV light–induced borylation of aryl halides under batch and continuous-flow conditions (Scheme 137a).^724^ Electronically diverse aryl halides were coupled with tetrahydroxydiboron and other diboron regents on a gram scale to afford the corresponding arylboronic acid, esters, and trifluoroborates (formed by treating arylboronic acids with KHF_2_). The haloarene reactivity increased with the decreasing dissociation energy of the Ar–X bond, which could be used to realize chemoselectivity. When the solvent was changed to isopropanol or HFIP, C–X/C–H diborylation was observed, and 1,2- and 1,3-diborylation could be regioselectively achieved by adjusting the solvent and haloarene substituents (Scheme 137b).^725^ 1,2-Diborylation was favored by isopropanol and *para* electron-withdrawing and *meta* alkyl groups or halogen atoms, whereas 1,3-diborylation was favored by HFIP and *ortho* alkyl groups/halogen atoms or *meta* electron-withdrawing groups. Scheme 137c illustrates the 1,2- and 1,3-diborylation mechanisms. The photoinduced homolytic cleavage of an Ar–X bond generates an aryl radical, which reacts with the diboron reagent to form an arylboronate and a boryl radical. The cage effect of isopropanol facilitates the subsequent addition reaction, which regioselectively yields a diborylated radical because of the stabilizing effect of the conjugated boryl group. Alternatively, the photoinduced heterolytic cleavage of an Ar–X bond in the presence of HFIP^340^ produces a triplet aryl cation, which reacts with an activated diboron anion to afford a borylated aryl radical cation. The recombination of these species regioselectively affords a 1,3-diborylated intermediate rather than other cationic intermediates, as the latter are destabilized by the π-accepting boryl group. Notably, *para* electron-deficient substituents favored 1,2-diborylation even in HFIP.

**Scheme 138:**
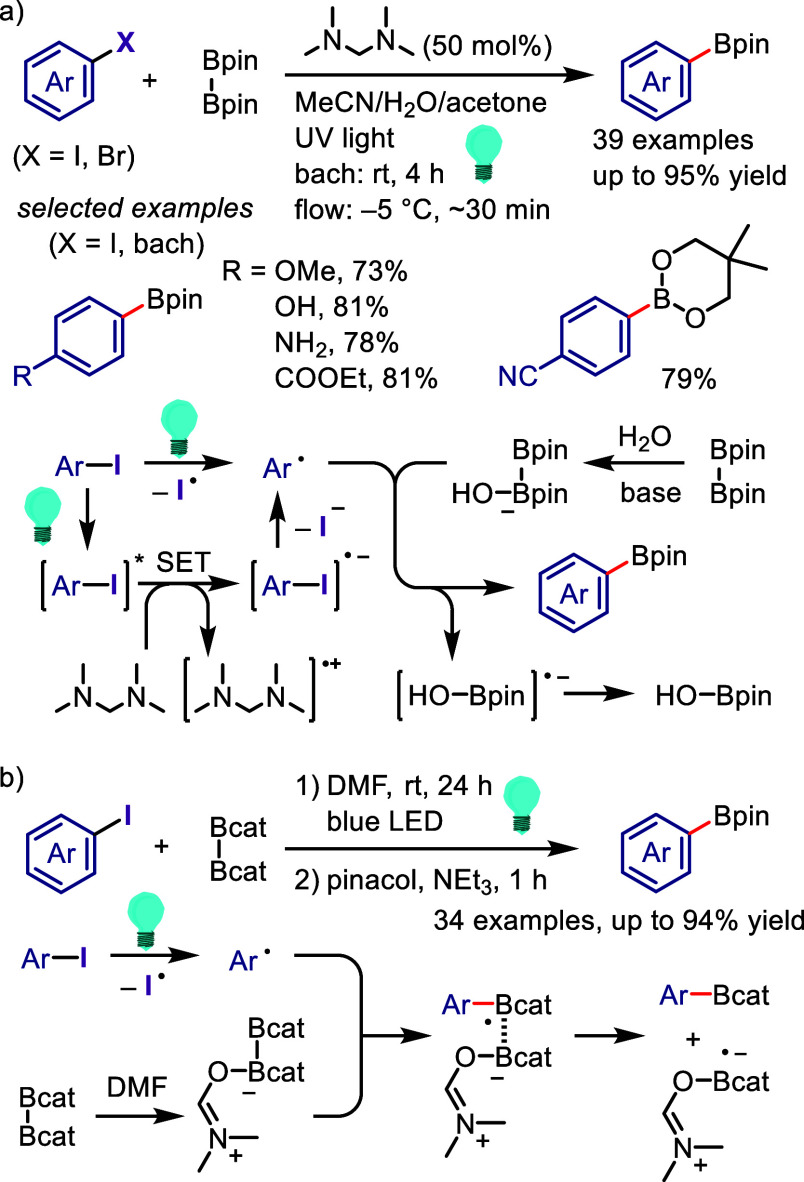
Additive- or Solvent-Assisted Photoinduced Borylation of Aryl Halides

Li et al. achieved the photoinduced borylation of aryl halides in the presence of *N*,*N*,*N′*,*N′*-tetramethyldiaminomethane (TMDAM) under batch and continuous-flow conditions (Scheme 138a).^726,727^ The combination of UV light and TMDAM was important for achieving high yields, with lower yields observed in the absence of either factor. According to the proposed mechanism, the UV irradiation–induced homolysis of the [Ar–I]* bond or reduction of the excited aryl iodide with TMDAM via SET generates an aryl radical. Concomitantly, TMDAM promotes the addition of water to B_2_pin_2_ to form a hydroxyborate intermediate, which reacts with the aryl radical to afford the desired product. The generated boryl radical anion is oxidized via SET to give a hydroxyborane. Studer et al. developed a mild protocol for photoinduced borylation with bis(catecholato)diboron (B_2_cat_2_) in the presence of DMF (Scheme 138b).^728,729^ In this scenario, the generated aryl radical reacts with B_2_cat_2_ to produce an aryl–boron bond, which is activated by DMF to afford an intermediate with a weak B–B one-electron σ-bond. The subsequent collapse affords the desired boronate, which is unstable and undergoes in situ transesterification with pinacol in the presence of Et_3_N to give the required pinacol boronate.

**Scheme 139:**
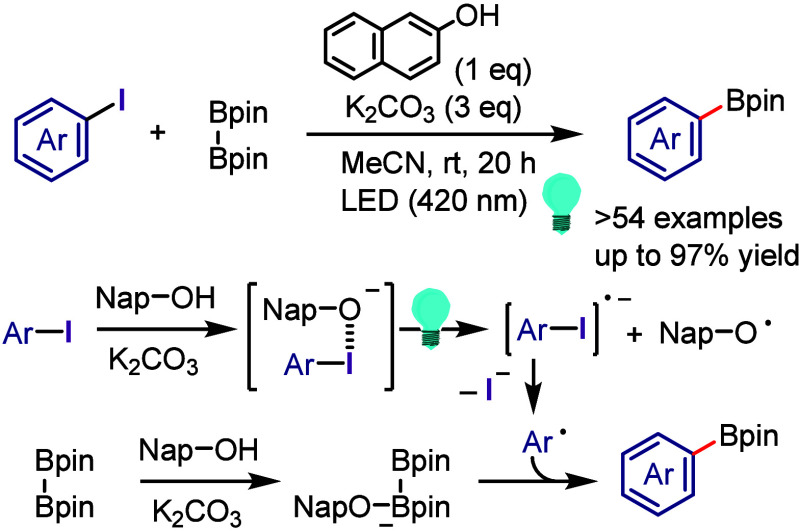
Photoinduced Borylation of Aryl Halides Involving EDA Complex Formation

Itoh et al. achieved photoinduced borylation by activating Ar–X bonds through halogen bonding.^730^ The reaction of haloarenes with B_2_pin_2_ in the presence of 2-naphthol (2-NpOH) as a halogen bond acceptor and K_2_CO_3_ under visible-light irradiation produced arylboronic esters (Scheme 139). According to the results of control experiments and spectroscopic studies, halogen bonding between Ar–X and naphthoxide affords an EDA complex, which is excited upon photoirradiation to generate an aryl radical via SET. The subsequent iodide elimination furnishes an aryl radical that reacts with the activated borate to form an arylboronic ester.

**Scheme 140:**
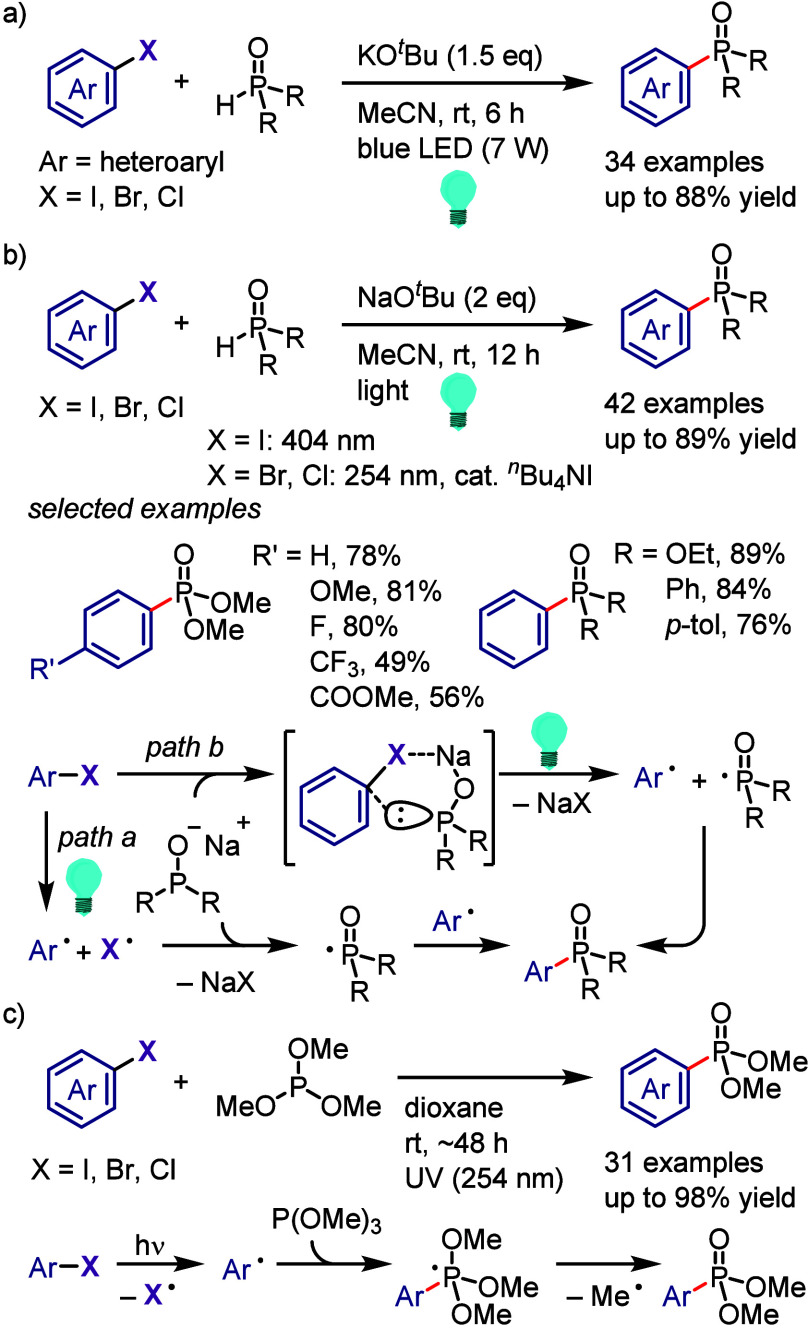
Photoinduced Phosphinylation/Phosphonylation Aryl Halides

Yu et al. phosphinylated heteroaryl halides under visible-light irradiation in the presence of diarylphosphine oxides (Scheme 140a).^731^ Zeng et al. developed a more practical protocol with a broad scope of substrates, including electronically and sterically diverse (hetero)aryl halides and H-phosphonates (dialkylphosphonates and diarylphosphine oxides) (Scheme 140b).^732^ According to the proposed mechanism, Ar–X bond photolysis generates aryl and halogen radicals (path a). The phosphonate anion is oxidized by the halogen radical via SET to generate a phosphonate radical, which couple with the aryl radical to afford the desired product. Alternatively, the aryl and phosphonate radicals can be produced by the photoexcitation of the Ar–X bond via a five-membered-ring transition state followed by intramolecular SET (path b). Hassan et al. synthesized (hetero)arylphosphonates via a UV light–induced photo-Arbuzov reaction between (hetero)aryl halides and trimethylphosphite under additive-free conditions (Scheme 140c).^733^ This reaction proceeds via the photoinduced generation of an aryl radical and its subsequent trapping by trimethylphosphite, with the subsequent methyl radical elimination affording the desired product.

**Scheme 141:**
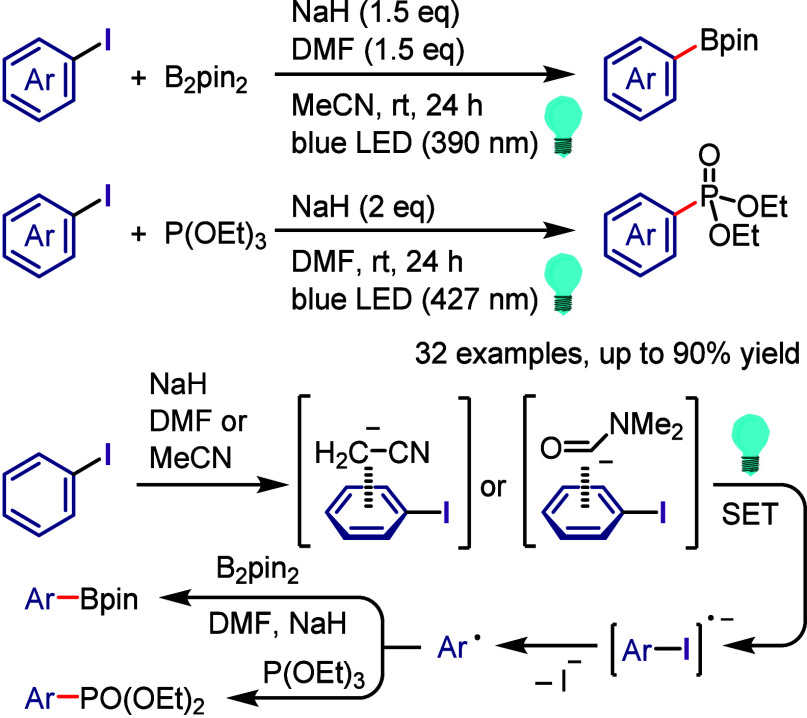
Photoinduced Borylation and Phosphonation of Aryl Halides via EDA Complex Formation

After the development of the dimsyl anion as an efficient electron donor for the formation of EDA complexes with electron-accepting aryl iodides and activation of Ar–I bond dissociation under visible light,^661,704,705^ Laulhé et al. revealed that the solvent anions of DMF and MeCN can photoactivate the borylation and phosphonation of aryl iodides through the formation of EDA complexes (Scheme 141).^734^ A broad range of aryl iodides could be reacted with either B_2_pin_2_ or P(OEt)_3_ to afford arylboronates or arylphosphonates, respectively. Mechanistic studies proposed the formation of solvent anion–aryl iodide EDA complexes, which generate aryl radicals through photoexcitation followed by SET. The subsequent radical trapping by DMF–B_2_pin_2_ or P(OEt)_3_ affords the desired products.

**Scheme 142:**
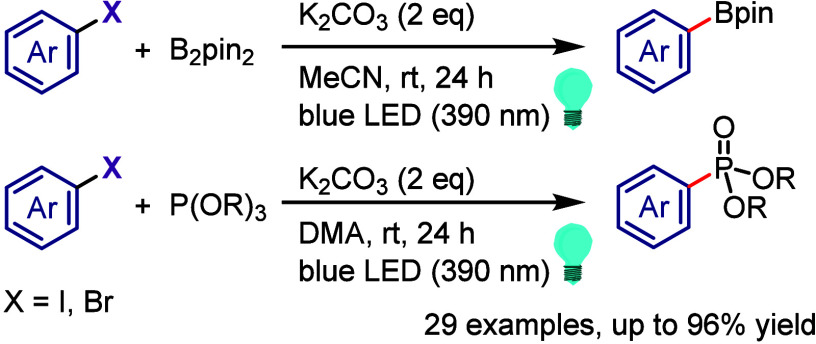
Carbonate Anion-Assisted Photodissociation of Aryl Halides and Formation of Ar–P and Ar–B Bonds

Roy et al. reported the carbonate anion–assisted photoactivation of aryl halides relying on anion−π interactions (Scheme 142).^735^ Several salts, particularly AcOK and K_3_PO_4_, effectively promoted the photoinduced dissociation of Ar–X bonds, with the highest activity observed for K_2_CO_3_. This simple protocol was successfully tested on a gram scale and applied to borylation and phosphonylation. The results of experimental and spectroscopic studies suggested that the key aryl radical intermediate is generated via an anion−π interaction between the carbonate anion and aryl halide and ruled out the involvement of interactions between the electron-rich phosphite and aryl halide in Ar–X bond dissociation.

### 4.2 Organophotocatalytic Activation of Ar–I Bonds

Photocatalytic reactions have been used to complement/access failed reactions and/or overcome the drawbacks associated with alternative transformations.^736−740^ Ru- and Ir-based organophotocatalysts are well suited for promoting arylation by aryl iodides but suffer from the scarcity of their metal components, high cost, low sustainability, and high toxicity. Therefore, transition metal-free organic photoredox catalysts hold promise as alternatives to their transition metal-based counterparts for aryl iodide activation, enabling robust, green, and sustainable arylation for modern organic synthesis.^626,630,636,741−749^ Organic photocatalysts are readily available, less toxic, and inexpensive and can utilize low-energy (e.g., visible) light-absorbing molecules, the structures of which can be tuned to improve their photophysical properties and photocatalytic performance. These organic compounds are privileged photocatalysts, avoiding the disadvantages of their precious metal-based counterparts and offering cheap and sustainable access to unique transformations and a broad array of substrates that are unreactive in most synthetic contexts. Additionally, the diversity of organophotocatalysts holds promise for the discovery and optimization of novel reactions.

**Scheme 143:**
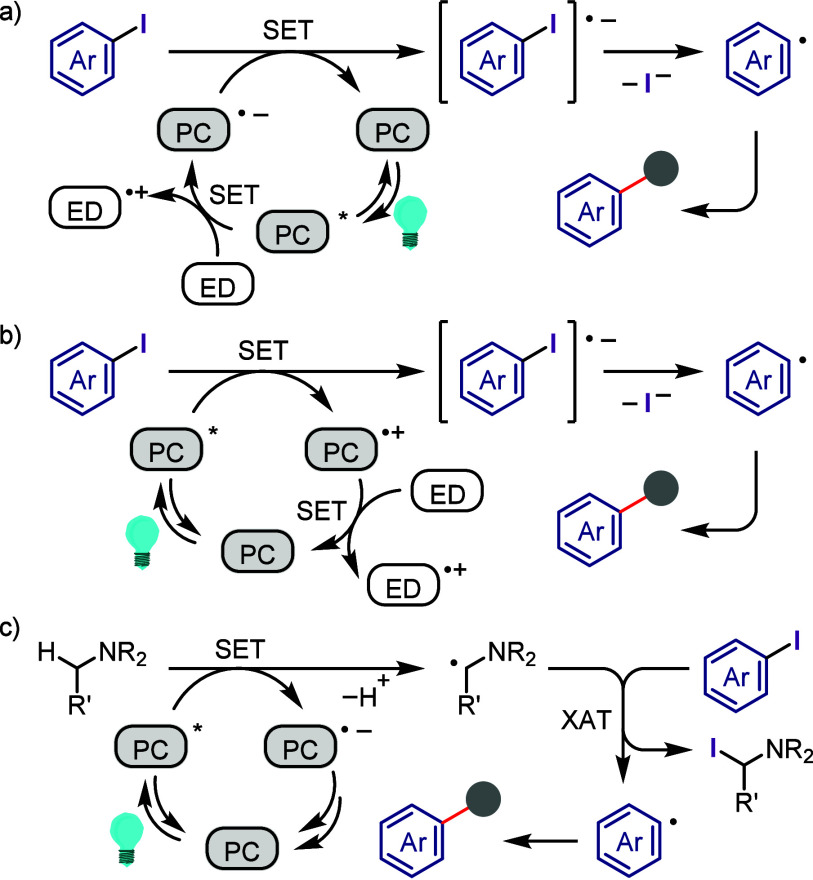
Generation of Aryl Radicals from Aryl Iodides Promoted by Organophotocatalysts

The interaction of an organic photocatalyst (PC) with light affords an excited state (PC*) suitable for reducing an aryl iodide via SET in an oxidative quenching cycle (Scheme 143a). Alternatively, PC* is reduced by an electron donor to generate PC^•–^ via SET, which is followed by SET-based aryl iodide reduction in a reductive quenching cycle (Scheme 143b). The organophotocatalyst promotes SET, providing access to previously inaccessible substrates (e.g., aryl halides) and transformations under mild conditions, thereby fostering the use of cheap and commercially available starting substrates.^474−491^ XAT is a promising alternative to SET for generating aryl radicals from aryl iodides via homolytic Ar–I bond cleavage, which involves iodine abstraction by an in situ generated alkyl radical under photoredox conditions (Scheme 143c).^645,750,751^ The advantages of these techniques have enabled the development of elegant visible light-induced activations of the challenging Csp^2^–X bonds in aryl halides without the participation of expensive and toxic transition metals under mild conditions.

**Scheme 144:**
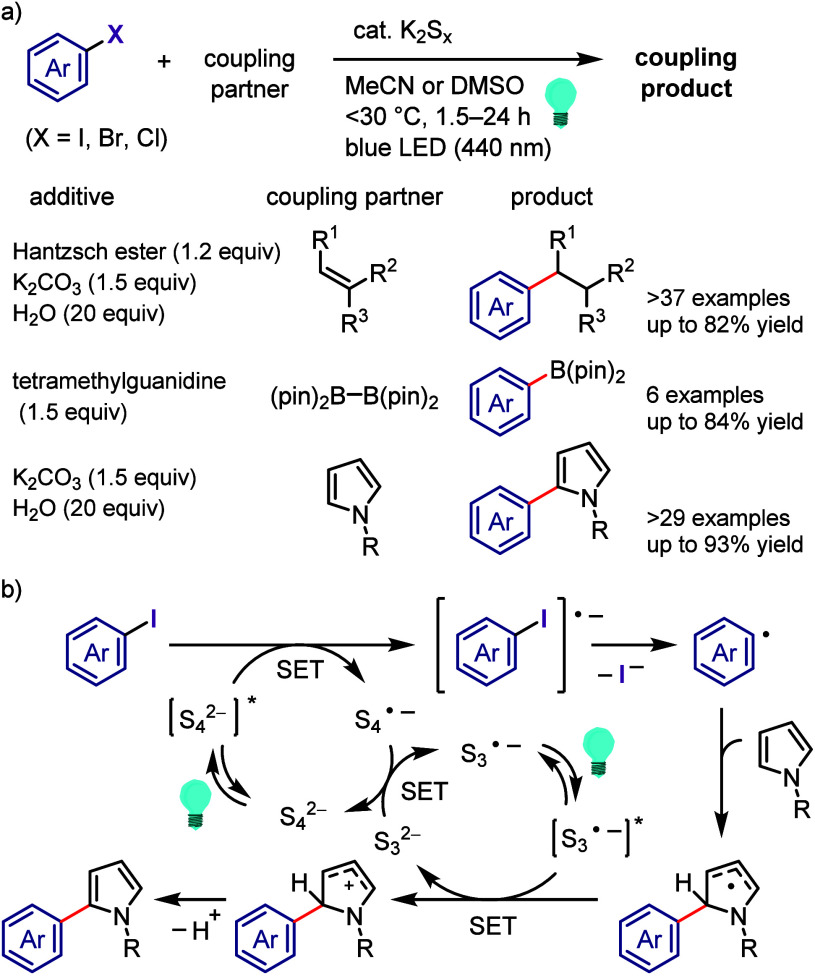
Polysulfide Anion-Photocatalyzed Functionalization of Aryl Halides

Chiba et al. demonstrated the catalytic reduction of aryl halides based on the formation of aryl radicals promoted by polysulfide anions as photocatalysts and their subsequent participation in the anti-Markovnikov hydroarylation of alkenes (in the presence of Hantzsch ester (HEH) as a reductant), borylation, hydrogenation, and biaryl formation (Scheme 144a).^752,753^ K_2_S_*x*_ was used to generate the photoactive S_3_^•–^, S_4_^2–^, and S_3_^2–^ species, with the ground-state redox potentials of S_4_^•–^/S_4_^2–^ and S_3_^•–^/S_3_^2–^ couples estimated at around −0.85 and −1.35 V vs SCE, respectively. For example, biaryl cross-coupling starts with the photoexcitation of S_4_^2–^, and the excited form reduces the aryl halide via SET to generate S_4_^•–^ and an aryl radical via the transient formation of an aryl halide radical anion (Scheme 144b). Charge transfer between S_4_^•–^ and S_3_^2–^ generates ground-state S_4_^2–^ and S_3_^•–^. The aryl radical adds to *N*-methylpyrrole to give a dearomatized biaryl radical, which is then oxidized by the photoexcited [S_3_^•–^]* to afford the desired product.

**Scheme 145:**
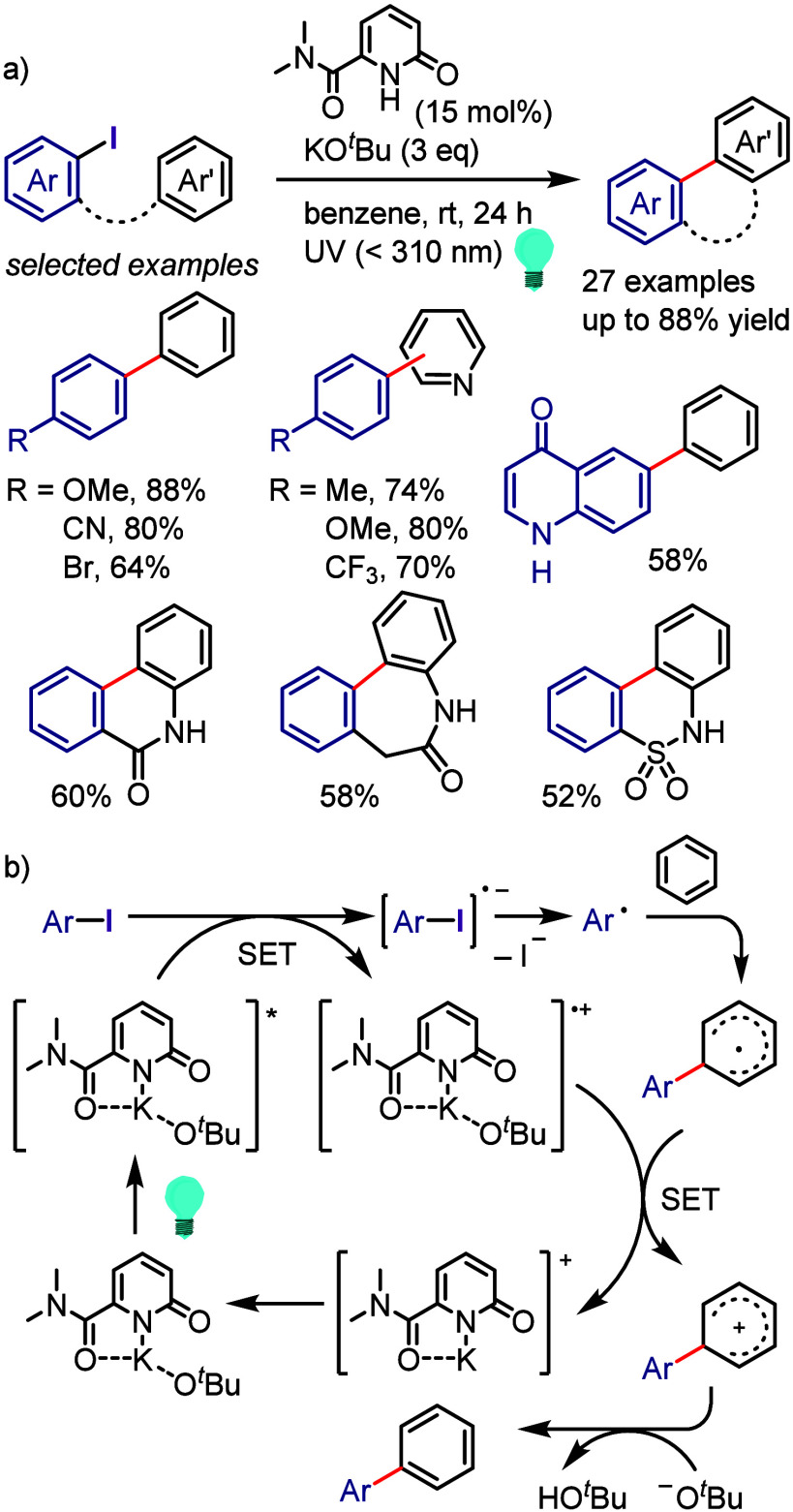
Photoinduced C(sp^2^)–H Arylation and Formation of Biaryls and Tricyclic Fused Lactams and Sultams

Among a small library of *N*,*N*- and *N*,*O*-coordinated organocatalysts, Das et al. explored pyridone amide as a potent organophotocatalyst for Ar–I bond activation inducing inter/intramolecular biaryl formation in the presence of ^*t*^BuOK under UV irradiation (Scheme 145a).^754^ The reaction tolerated a broad range of (hetero)aryl iodides and provided access to tricyclic lactam and sultam derivatives via intramolecular C–C bond formation. According to the proposed mechanism (Scheme 145b), the catalytic cycle is initiated by the deprotonation of the organocatalyst, with subsequent photoirradiation generating an excited complex. Subsequently, SET to Ar–I generates an aryl radical and the pyridone amide radical cation. The aryl radical is trapped by the arene substrate to give a biaryl radical, which is oxidized by the pyridone amide radical cation via SET to produce a biaryl cation and cationic amide complex. In the presence of a base, the biaryl cation is deprotonated to afford the coupling product.

**Scheme 146:**
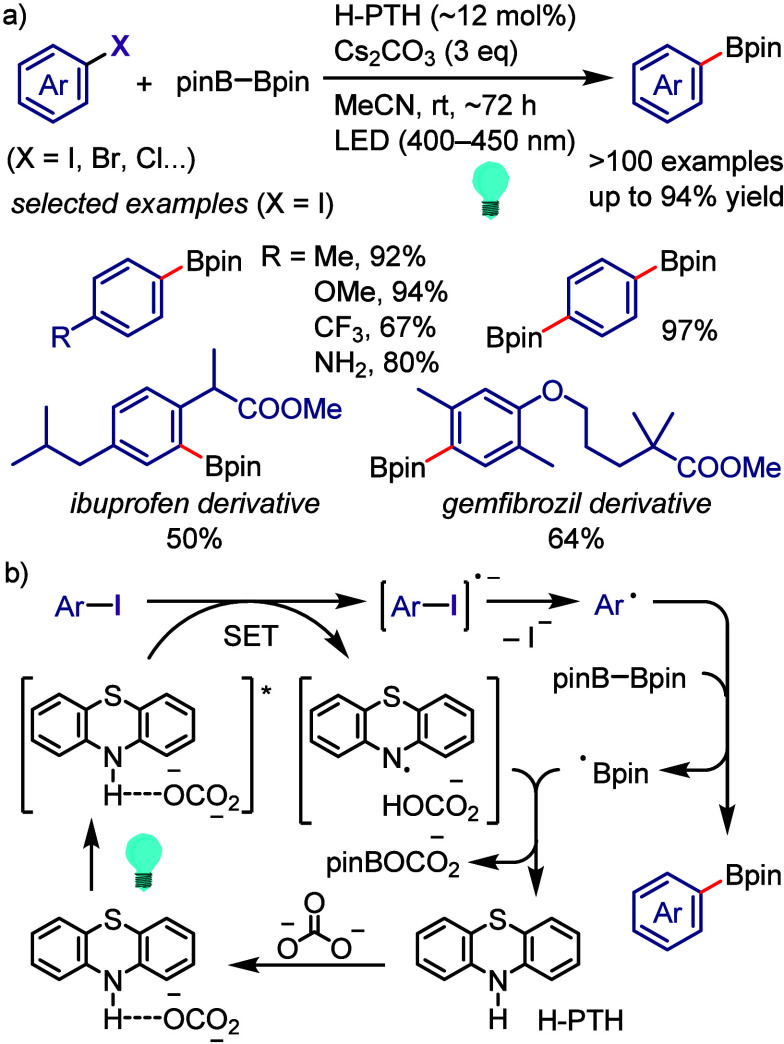
Visible Light/Phenoxide-Induced Borylation of Ar–X Bonds via Photoinduced Proton-Coupled Electron Transfer

10-Phenylphenothiazine (Ph-PTH) was used as an organophotocatalyst for the chemoselective hydrodehalogenation and C–C cross-coupling of polyhalogenated arenes.^755^ Larionov et al. developed phenothiazine (H-PTH) as an organic photocatalyst for the visible light–induced borylation of aryl iodides (Scheme 146a).^756^ In the presence of Cs_2_CO_3_, the reduction potential of phenothiazine reached approximately −3 V vs SCE, which enabled the photoborylation of Ar-O/N/Cl/Br/I substrates. The process was suitable for gram-scale synthesis and the borylation of structurally complex substrates, including bioactive ingredients and natural products, showing a high functional group tolerance. The photoactivation mechanism was based on photoinduced proton-coupled electron transfer, as verified by experimental and theoretical studies (Scheme 146b). According to the proposed mechanism, a complex consisting of H-PTH and Cs_2_CO_3_ held together by hydrogen bonding was converted to a singlet excited state under photoirradiation, with subsequent SET generating an aryl radical and a phenothiazinyl radical–hydrogen carbonate complex. The aryl radical reacts with B_2_pin_2_ to afford the desired borylation product along with a boryl radical, which reacts with the phenothiazinyl radical–hydrogen carbonate complex to regenerate H-PTH.

**Scheme 147:**
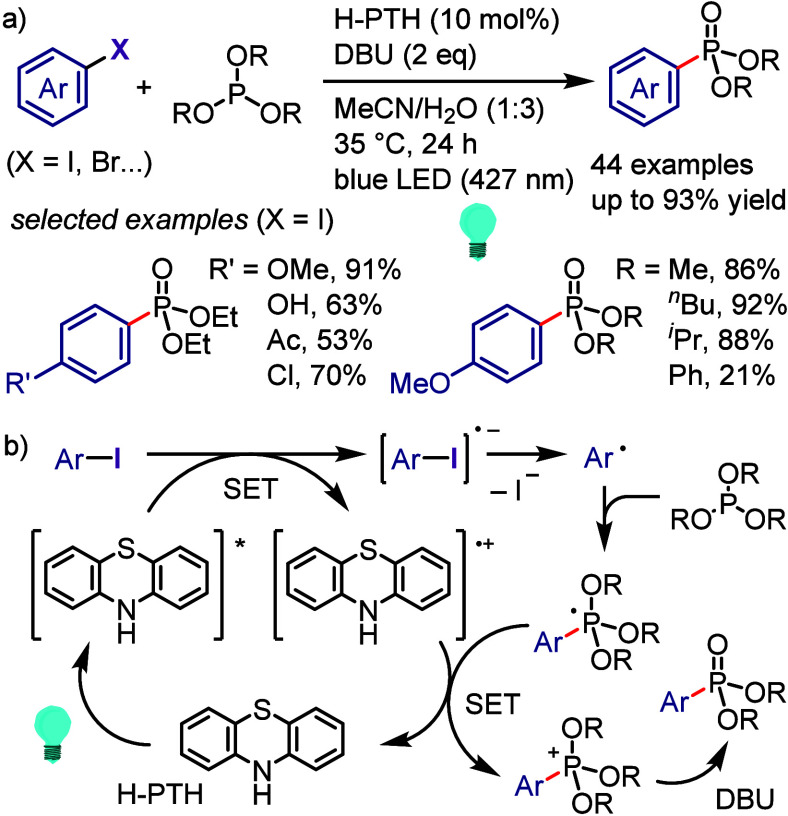
Phenothiazine-Photocatalyzed Phosphonation of Aryl Halides

Phenothiazine (H-PTH) photocatalysts were used for the phosphonation of aryl halides to form aromatic phosphonates under ambient conditions (Scheme 147a).^757^ According to the mechanism proposed based on the results of radical trapping and fluorescence quenching experiments (Scheme 147b), the photoirradiation of H-PTH generates an excited species, which reduces the aryl halide via SET to form an aryl radical and the H-PTH radical cation. The aryl radical reacts with phosphite to generate a phosphoranyl radical, which is oxidized by the H-PTH radical cation via SET to generate a phosphonium cation. The subsequent reaction with DBU affords the desired product.

**Scheme 148:**
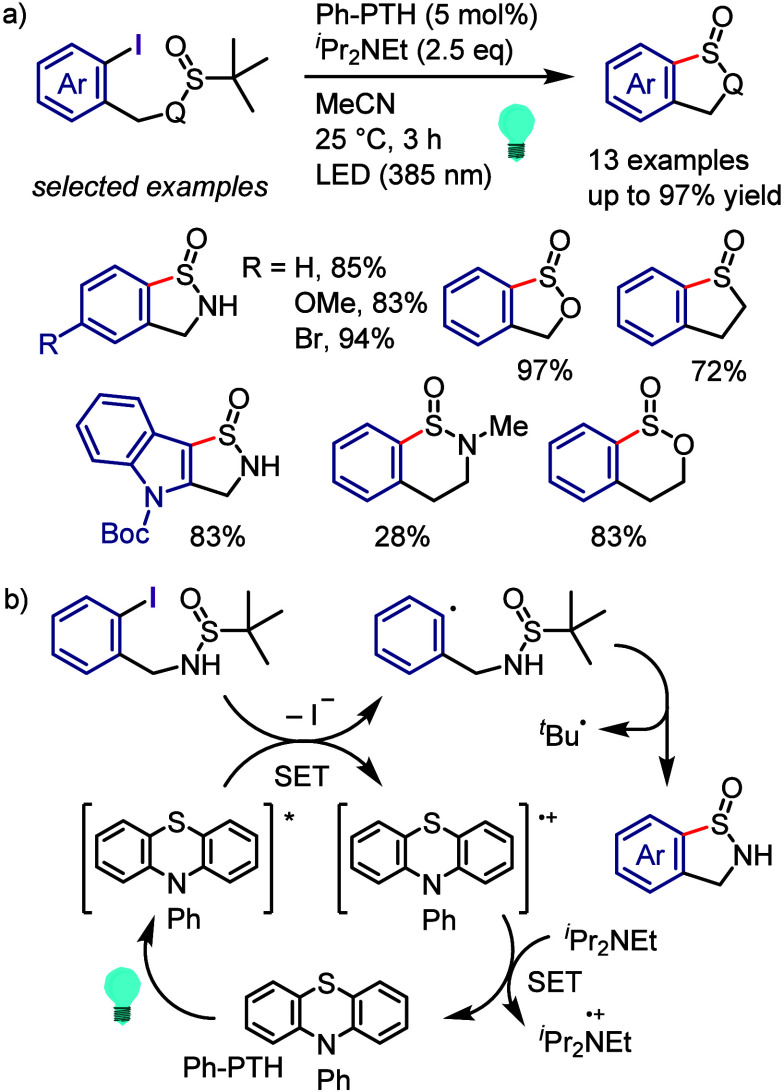
Photocatalytic Intramolecular Homolytic Substitution-Based Cyclization or Aryl Iodides

Maestro and Alemán constructed valuable heterocyclic skeletons via the photoinduced generation of aryl radicals in the presence of Ph-PTH followed by trapping by S, P, and Si atoms (Scheme 148a).^758^ The suggested mechanism starts with the reduction of the aryl halide by the photoexcited Ph-PTH (Ph-PTH*) through SET, and the thus generated aryl radical is attacked by the sulfur lone pair. The subsequent homolytic cleavage of the ^*t*^Bu bond affords a cyclic product (Scheme 148b). The catalytic cycle is completed by the reduction of Ph-PTH^•+^ via SET in the presence of DIPEA, which regenerates the active catalyst (Ph-PTH). This strategy was applied to the synthesis of cyclic sulfinamides, sultines, sulfoxides, phosphonates, and silyl ethers.

**Scheme 149:**
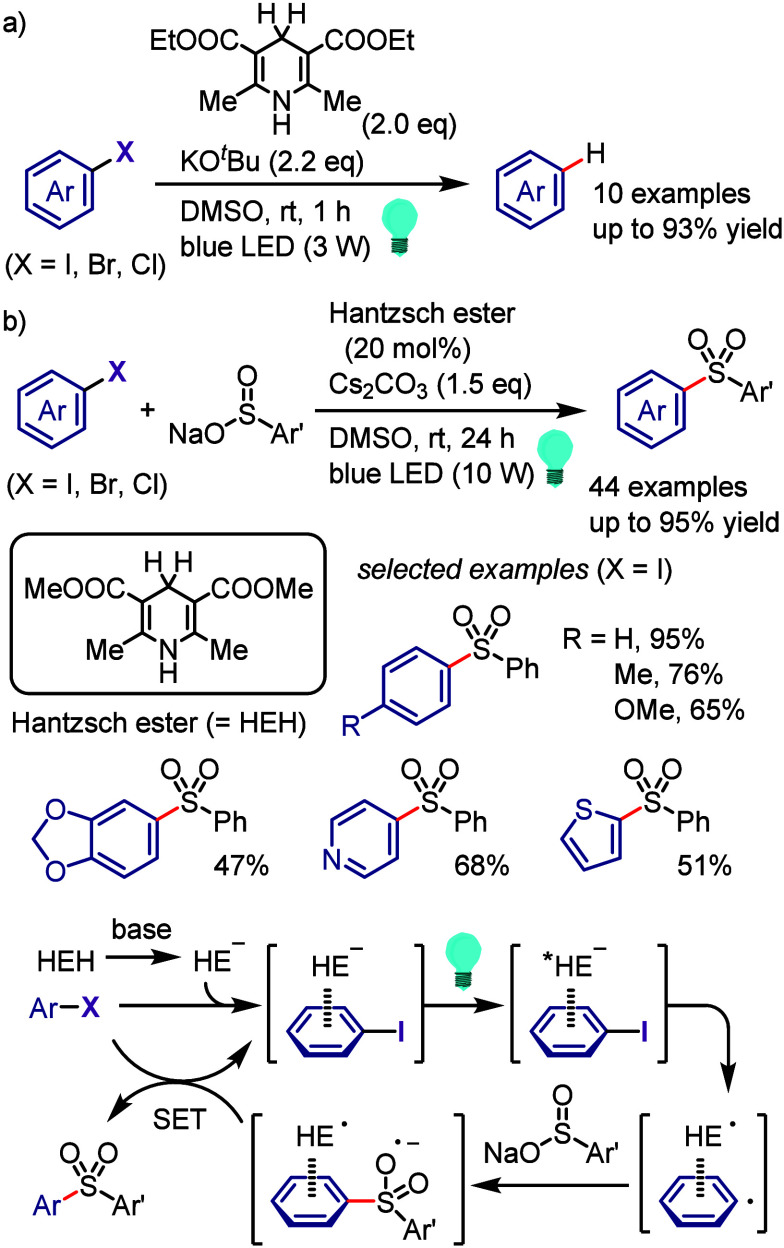
Hantzsch Ester-Photocatalyzed Hydrodehalogenation and Sulfonation of Haloarenes

Budén et al. hydrodehalogenated aryl halides using Hantzsch ester (HEH) as a visible light-absorbing electron-donating photoredox reagent (Scheme 149a).^759^ Li et al. used HEH to couple haloarenes and arylsulfinates under visible light (Scheme 149b).^760^ HEH was also used in the photocatalytic formation of C–S bonds in diarylsulfones, and this process was successfully performed on a preparative scale under sunlight. According to the proposed mechanism, deprotonated Hantzsch ester (HE^–^) interacts with Ar–X to form an EDA complex, the excited state of which has a reduction potential (*E*_red_ = −2.49 V vs SCE) sufficient for converting Ar–X into an aryl radical with the concomitant formation of the HE^•^ radical. The coupling of the aryl radical with an arylsulfinate gives a sulfone radical anion, which forms an EDA complex with the HE^•^ radical. Finally, SET to another aryl halide molecule affords the desired product.

**Scheme 150:**
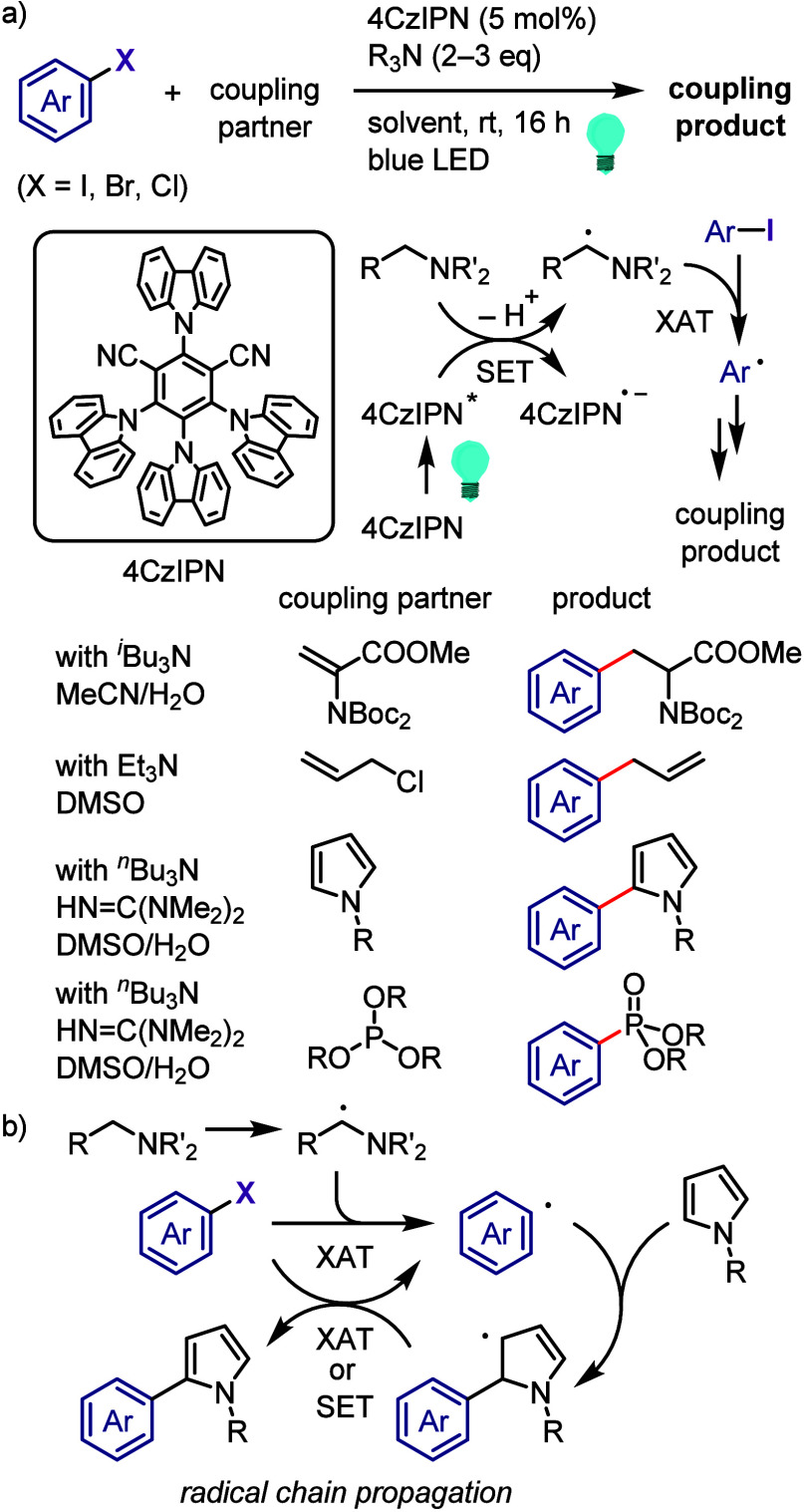
Aminoalkyl Radical-Mediated Cross-Coupling of Aryl Halides Based on Halogen-Atom Transfer (XAT)

Leonori et al. generated aryl radicals under photoredox conditions via the in situ generation of α-aminoalkyl radicals as Ar–X bond activators via XAT.^645,744−746^ Besides the Giese alkylation and allylation of aryl iodides using tertiary amines as α-aminoalkyl radical precursors and 4CzIPN as a photocatalyst under blue-light irradiation (Scheme 150a),^761^ this strategy was successfully applied to the arylation of pyrroles and phosphites with aryl halides under photoredox conditions.^762^ Experimental and computational studies pointed to the importance of 4CzIPN and the generated α-aminoalkyl radicals as initiators for the radical chain propagation mechanism. The excited form of the catalyst produced upon irradiation cannot directly activate Ar–X bonds via SET but rather oxidizes the tertiary amine to generate an α-aminoalkyl radical as the key intermediate for Ar–X bond activation via XAT. The thus generated aryl radical reacts with pyrrole to form a biaryl radical, which engages in XAT with the other aryl halides to afford the desired product and regenerate the aryl radical for the propagation cycle (Scheme 150b).

**Scheme 151:**
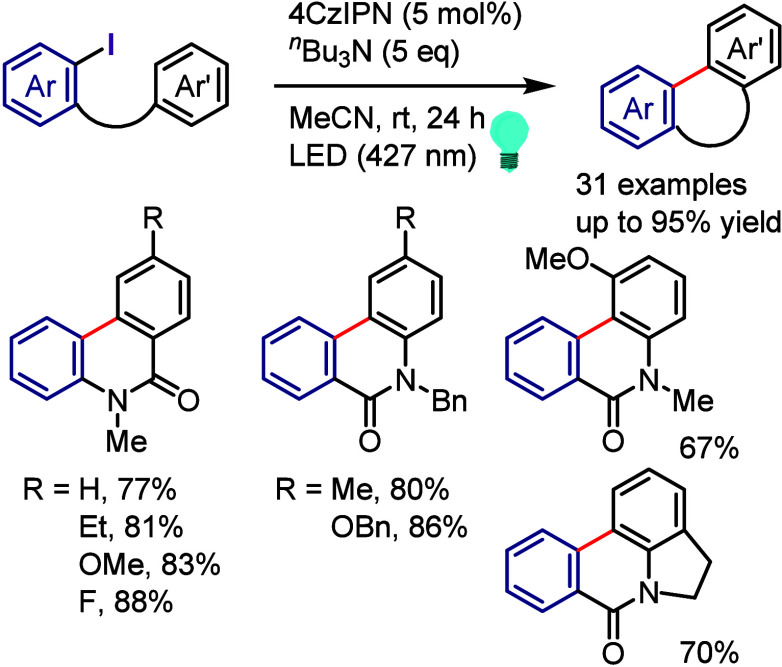
Aminoalkyl Radical-Mediated Intramolecular Cyclization of Aryl Halides Based on XAT

Baidya et al. used α-aminoalkyl radicals as powerful XAT agents to generate aryl radicals, with subsequent intramolecular cyclization affording biologically relevant alkaloids (Scheme 151).^763^ For example, the irradiation of *ortho*-iodo-substituted *N*-arylbenzamides in the presence of 4CzIPN/^*n*^Bu_3_N afforded phenanthridinone derivatives, including natural products from the Amaryllidaceae family.

**Scheme 152:**
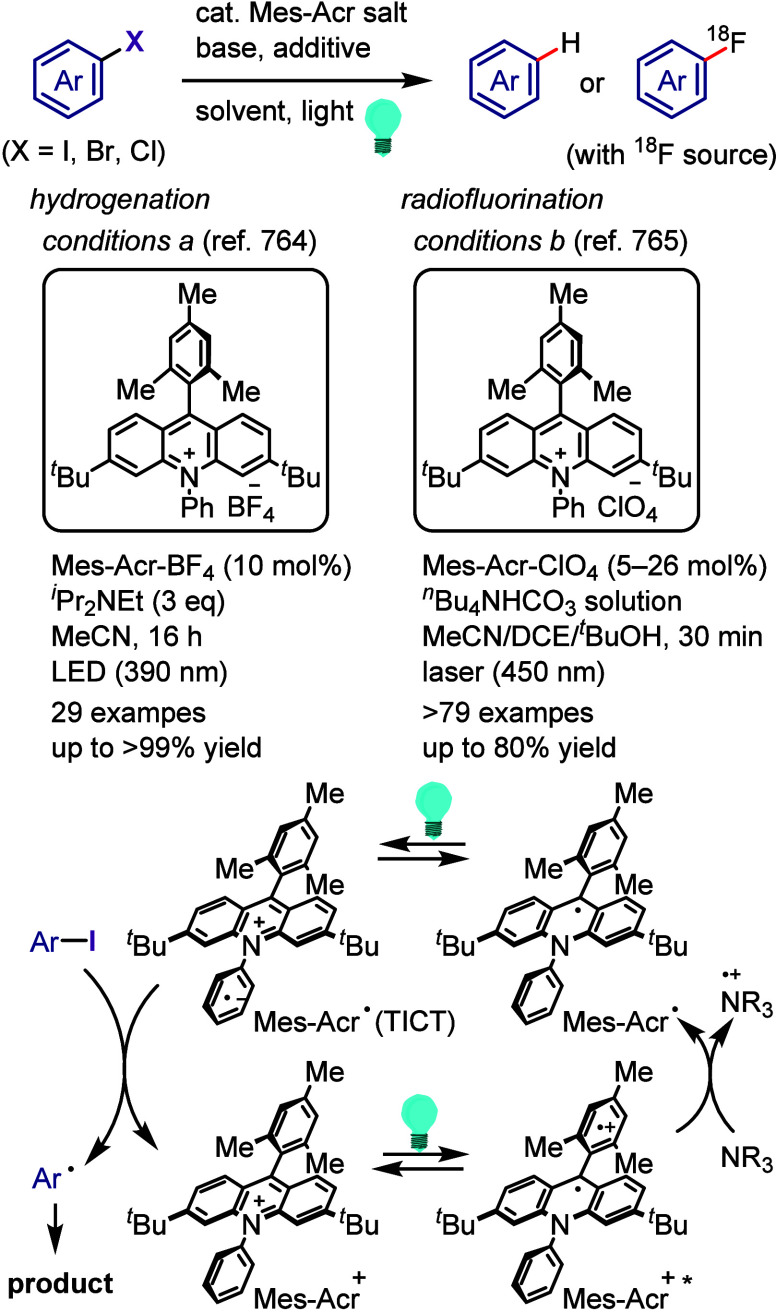
Hydrodehalogenation and Radiofluorination Promoted by Mes-Acr Photocatalysts

Nicewicz et al. developed various dehalogenative transformations, such as hydrodehalogenation and radiofluorination via halide/^18^F^–^ exchange, facilitated by acridinium photocatalysts (Mes-Acr^+^) as efficient photochemical reductants (Scheme 152).^764−767^ In these systems, the photoexcited catalyst (Mes-Acr^+^*) engages in SET with a tertiary amine to generate a Mes-Acr radical, which is photoexcited to afford a strongly reducing twisted intramolecular charge-transfer state that reacts with an aryl halide to give an aryl radical and regenerate Mes-Acr^+^.

**Scheme 153:**
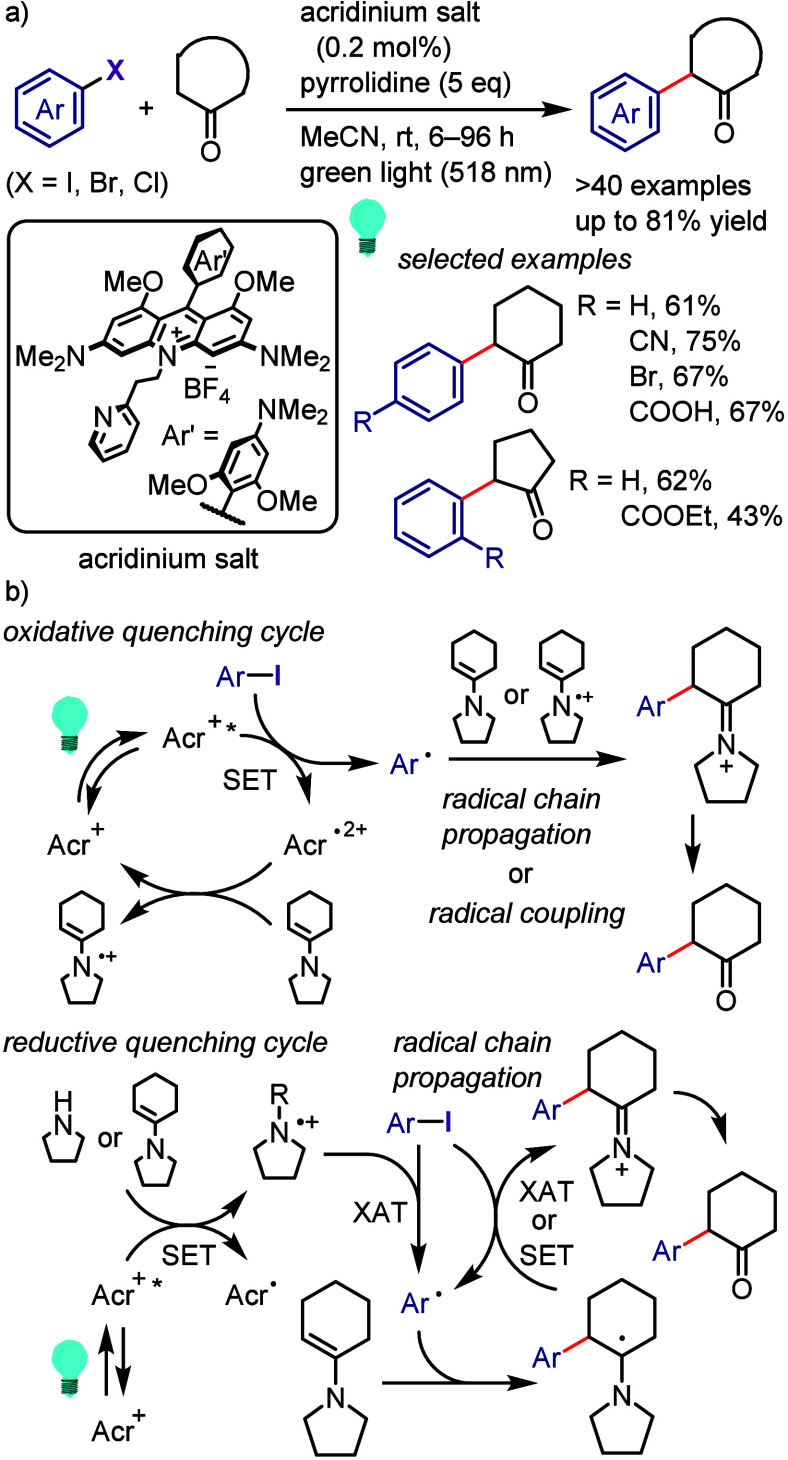
α-Arylation of Cyclic Ketones Promoted by Acridinium Photocatalysts

Gianetti et al. used a green light-absorbing acridinium photocatalyst for the efficient α-arylation of cyclic ketones with aryl halides in the presence of pyrrolidine to form α-aryl ketones (Scheme 153a).^768^ This method was applied to the multigram-scale syntheses of a selective β3-adrenergic agonist precursor and other biologically valuable intermediates. Mechanistic investigations revealed the possibility of the concurrent contributions of SET and XAT (Scheme 153b). Upon green-light irradiation, the catalyst is converted to an excited state (Acr^+^*) capable of engaging in oxidative/reductive quenching cycles (*E*_1/2_(C^2+•^/C^+*^) = −1.85 V and (*E*_1/2_(C^+*^/C^•^) = +1.15 V vs SCE). In the oxidative cycle, Acr^+^* acts as a strong reductant and engages in SET to the desired aryl halide to generate an aryl radical and the oxidized catalyst. The aryl radical reacts with an enamine intermediate via radical chain propagation or radical–radical coupling to afford the desired product after hydrolysis, while the oxidized catalyst engages in SET with the enamine to regenerate the acridinium salt. In the case of the reductive quenching cycle, the excited catalyst accepts an electron from the employed amine to provide an amine radical cation, which generates an aryl radical via XAT. The aryl radical initiates the radical propagation cycle to afford the desired product.

**Scheme 154:**
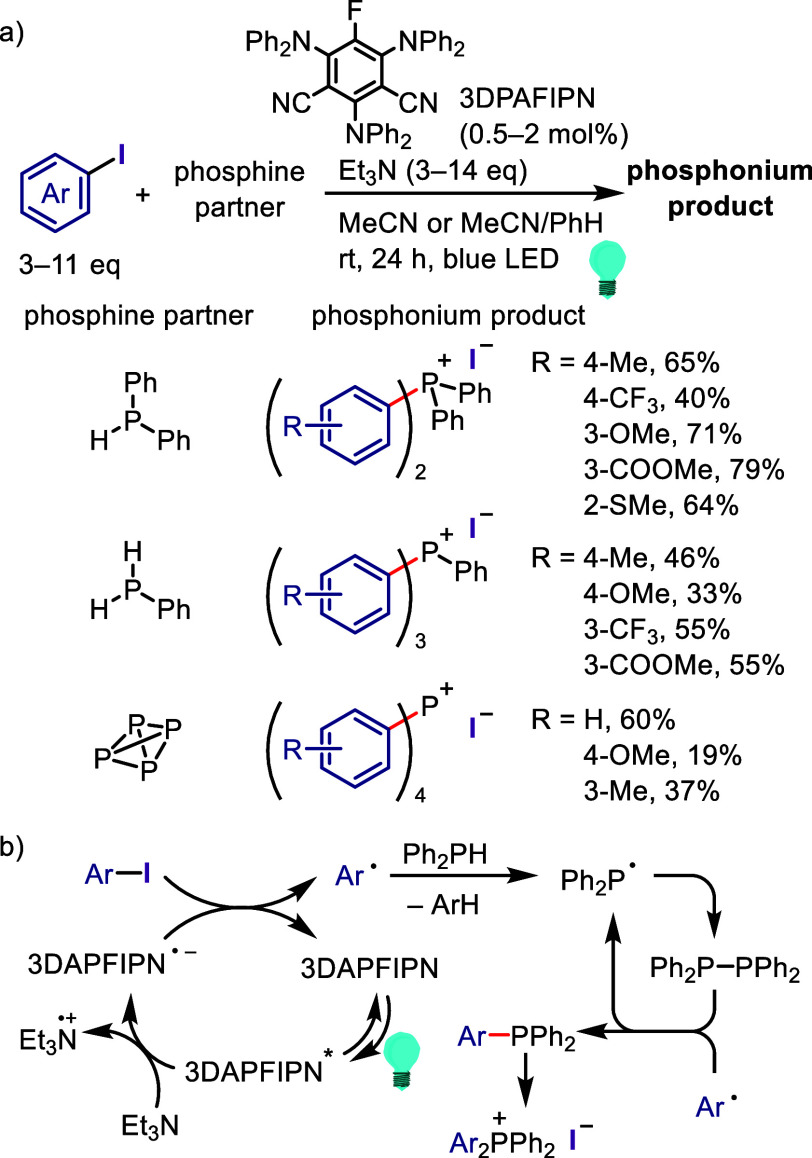
3DPAFIPN-Catalyzed Arylation of Phosphines and Phosphorus under Visible-Light Irradiation

Wolf et al. replaced precious Ir photocatalysts with a cheap organic alternative (3DPAFIPN) and used it to arylate HPPh_2_ with aryl iodides (Scheme 154a).^769,770^ The metal-free process showed superior productivity and selectivity toward quaternary phosphonium salts at base and catalyst loadings lower than those required by Ir catalysts. This more versatile metal-free synthetic method featured a broader scope, including the arylation of arylphosphines (e.g., H_2_PPh) and white phosphorus (P_4_) and affording the related asymmetrical/symmetrical products with excellent selectivity. Regarding the aryl iodide substituents, *ortho* substituents resulted in a preference for tertiary phosphines, whereas *meta* and *para* substituents resulted in the exclusive formation of quaternary phosphonium salts. According to the proposed mechanism (Scheme 154b), the photoexcited 3DAPAFIPN* engages in SET with Et_3_N to generate the 3DAPAFIPN radical anion [*E*_1/2_ (PC/PC^•–^) = −1.59 V vs SCE], which reduces the aryl iodide to give an aryl radical and regenerate the catalyst. The aryl radical abstracts a hydrogen atom from the phosphine to give a phosphine radical, which dimerizes to generate a diphosphine that is converted into a tertiary phosphine which is attacked by the aryl radical.

**Scheme 155:**
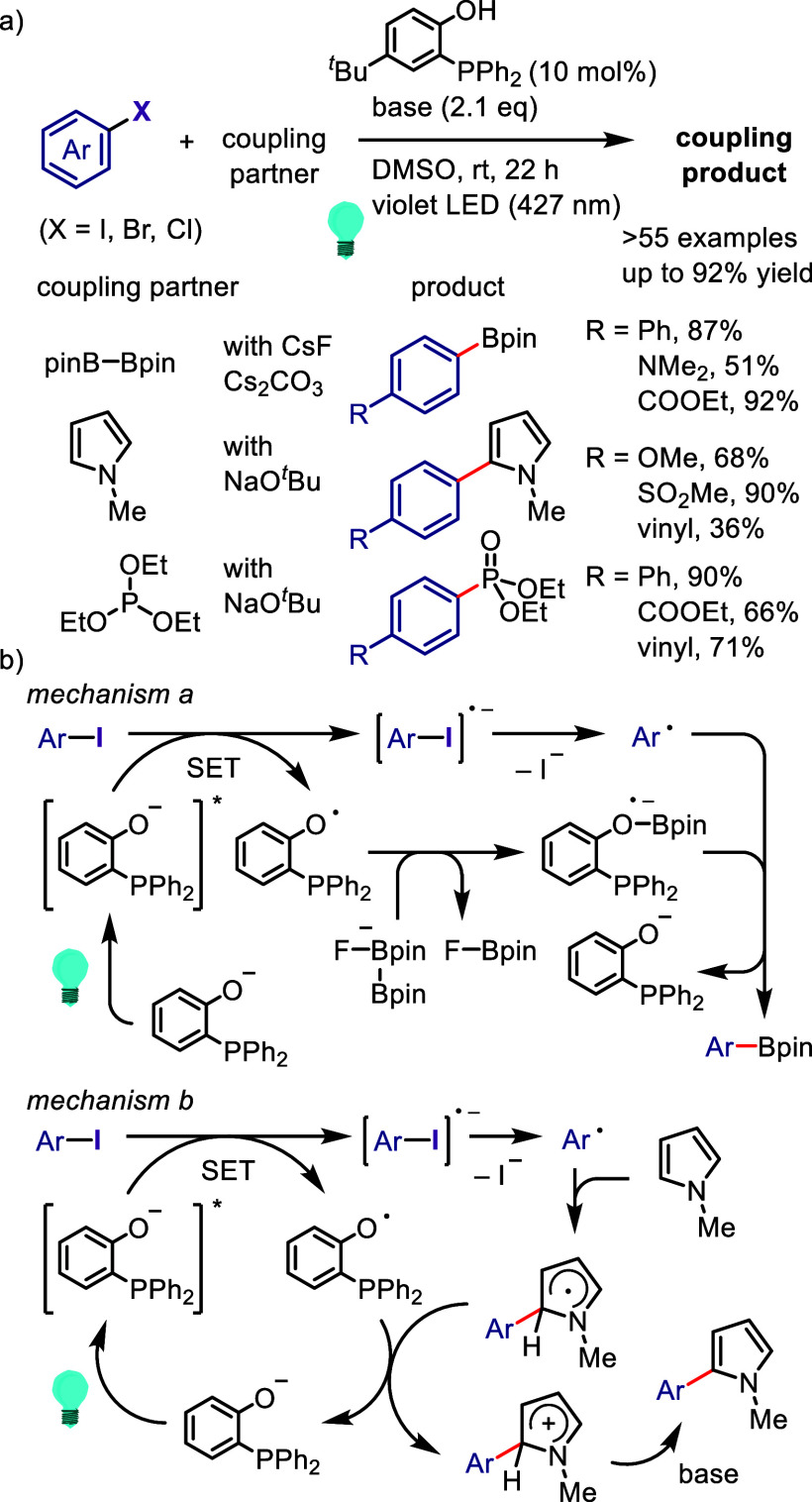
*ortho*-Phosphinophenolates as Potent Photoredox Catalysts for the Borylation, Arylation, and Phosphonation of Aryl Halides

Shang et al. designed diarylamides and (thio)phenolates with nitrogen, sulfur, oxygen, and *ortho*-diphenylphosphino groups as strongly reducing photocatalysts for activating challenging Ar–X and other bonds by visible light.^771−773^ The scope of this process included the coupling of aryl halides with diboron reagents, *N*-methylpyrrole, and triethylphosphite, which afforded the corresponding arylboronates, 2-arylpyrroles, and arylphosphonates, respectively (Scheme 155a).^773^ According to the suggested mechanism, the irradiation of the deprotonated phenolate catalyst generates an excited state (phenolate*) that activates the aryl halide via SET to afford aryl and phenoxy radicals (Scheme 155b). The reaction of the phenoxy radical with the activated diboronate affords the persistent borate radical anion, which reacts with the aryl radical to afford the desired product and regenerate the phenolate catalyst (mechanism a). In the case of arylation, the aryl radical is trapped by pyrrole to generate a biaryl radical, which is oxidized by the phenoxy radical to generate the desired biaryl after deprotonation by a base (mechanism b).

**Scheme 156:**
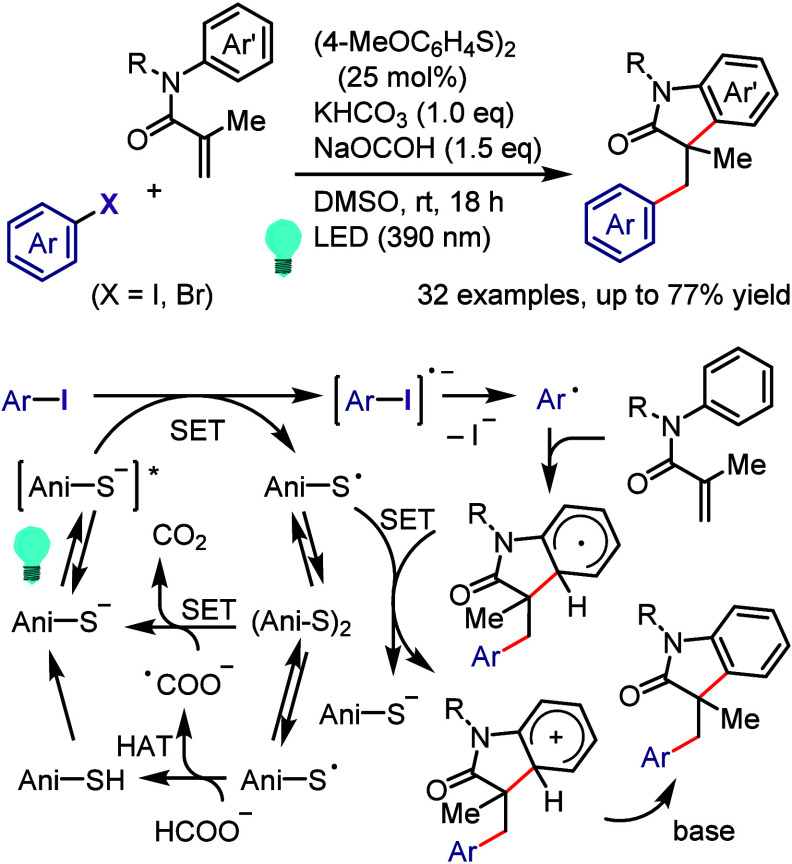
Thiophenolate-Catalyzed Photoactivation of Aryl Halides for Addition to Aryl Acrylamides

Molander et al. used thiophenolate as a photoredox catalyst to mediate the photoactivation of (hetero)aryl halides followed by Giese addition, thus obtaining 3,3′-disubstituted oxindoles (Scheme 156).^774^ The postulated mechanism starts with the photolysis of the disulfide catalyst to generate a thiyl radical, which abstracts hydrogen from HCO_2_Na to produce thiophenol. The deprotonation of thiophenol or the single-electron reduction of the disulfide catalyst delivers the thiolate, which is photoexcited to a strongly reducing state. SET between the excited thiolate and aryl halide generates an aryl radical, which undergoes Giese addition to acrylamide followed by SET with the thiyl radical to generate the desired oxindole after deprotonation.

**Scheme 157:**
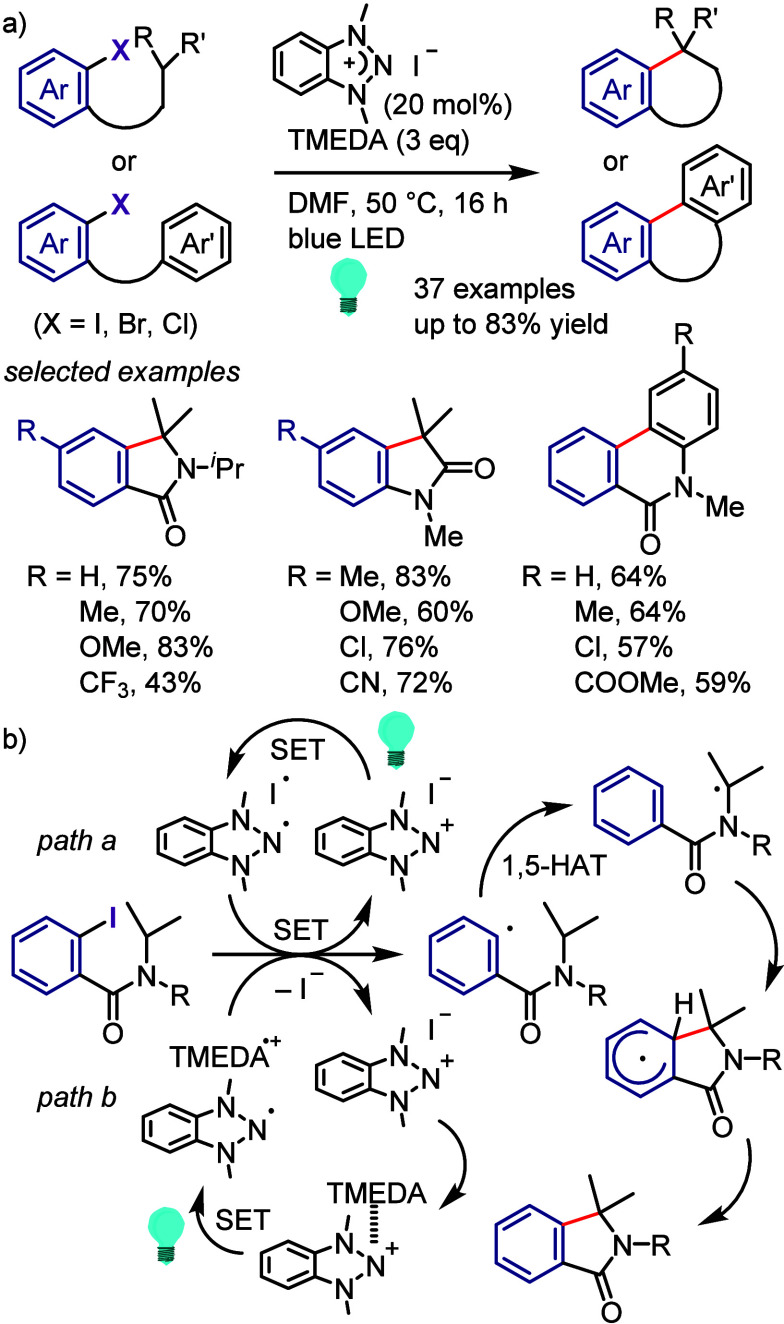
*N*-Heterocyclic Nitrenium Salts as Effective Photoredox Catalysts for Haloarene Activation

Chen et al. employed an *N*-heterocyclic nitrenium salt (NHN) as a potent photoredox catalyst for the reduction of aryl halides that generated aryl radicals and enabled a variety of radical transformations.^775^ Aryl halides bearing amide moieties were transformed into the corresponding cyclization products, such as isoindoline-1-ones, oxindoles, and phenanthridine-6(5*H*)-ones, via an aryl halide reduction/1,5-HAT/cyclization sequence in the presence of NHN under blue light (Scheme 157a). Other radical reactions, such as hydrodehalogenation, cascade cyclization/hydrodehalogenation, and biaryl cross-coupling, were also successfully carried out using NHN photocatalysis. Two pathways were proposed based on the results of experimental studies (Scheme 157b). In the first pathway, NHN photoexcitation results in intramolecular charge transfer, which generates an aminyl radical that reduces the aryl halide via a CT complex to generate an aryl radical. The following 1,5-HAT and intramolecular cyclization and aromatization via deprotonation afford the desired product. Alternatively, the aryl radical can be generated via SET from the photoexcited NHN/TMEDA CT complex.

**Scheme 158:**
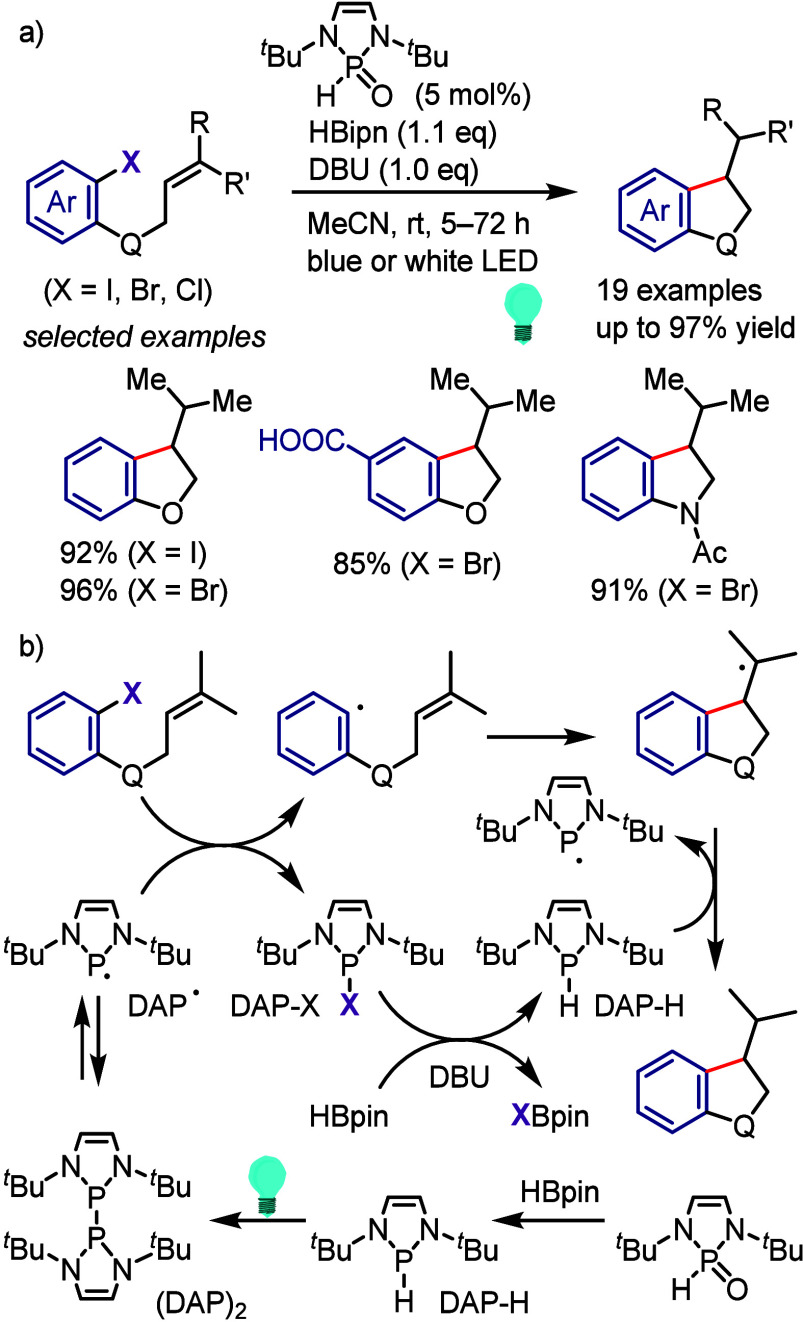
Diazaphospholene-Photocatalyzed Cyclization of Organohalides

Diazaphospholene and diazaphosphinane organocatalysts enable the radical functionalization of aryl halides.^776−779^ Cramer et al. used 1,3,2-diazaphospholene hydride (DAP-H) generated from the corresponding phosphine oxide as a pivotal catalyst in the presence of DBU and HBpin to achieve the photoinduced radical cyclization of olefinic group–bearing aryl halides and thus access diverse cyclic skeletons (Scheme 158a).^780^ Various aryl halides were used to construct dihydrobenzofurane, chromane, and indoline heterocycles. According to the proposed mechanism, the reduction of the phosphine oxide precatalyst by HBpin affords DAP-H, which dimerizes to (DAP)_2_ under irradiation (Scheme 158b). The dissociation equilibrium between (DAP)_2_ and 2DAP^•^ enables halogen abstraction from the aryl halide to initiate the radical chain process. The obtained DAP-X regenerates DAP-H in the presence of DBU/HBpin, whereas the aryl radical undergoes a 5-*exo*-trig cyclization to generate an alkyl radical. HAT between the generated radical and DAP-H affords the desired product and DAP^•^, which propagates the radical chain process.

**Scheme 159:**
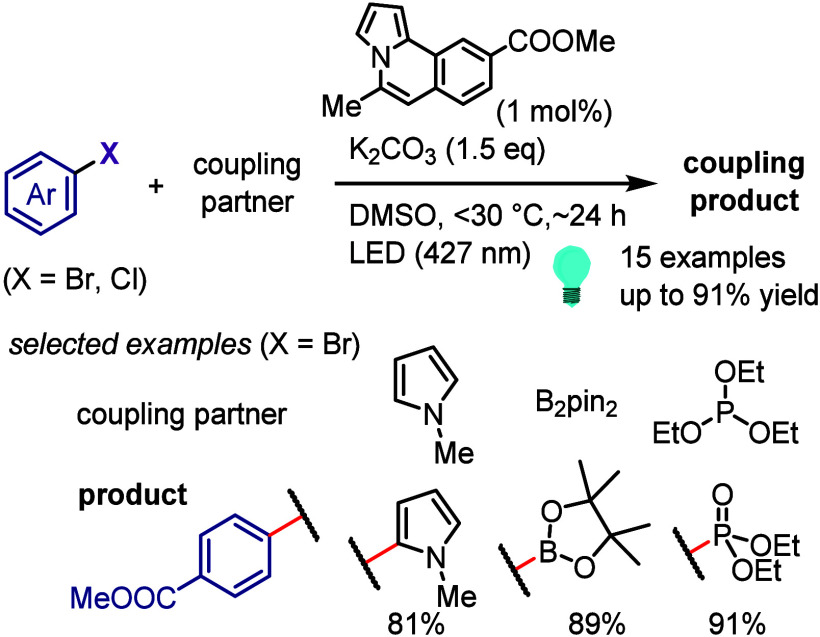
Pyrroloisoquinoline as an Efficient Photoredox Catalyst for Ar–X Bond Activation

Abe et al. designed a library of pyrrolo[2,1-*a*]isoquinolines as multifunctional photoredox organocatalysts (Scheme 159).^781^ A photocatalyst with advantageous S_1_ (E*_ox_^S^ = −2.06 V vs SCE) and T_1_ ((*E*_0,0_^T^) = 2.58 eV (59.5 kcal/mol) states was used to achieve photocatalyzed SET and energy-transfer reactions. For example, this organophotocatalyst promoted the couplings of aryl halides with (hetero)arenes, B_2_pin_2_, and triethylphosphite under visible light. The photoexcitation of this catalyst affords a singlet state with a highly negative oxidation potential, which results in the generation of an aryl radical, with the following coupling reaction affording the desired product.

**Scheme 160:**
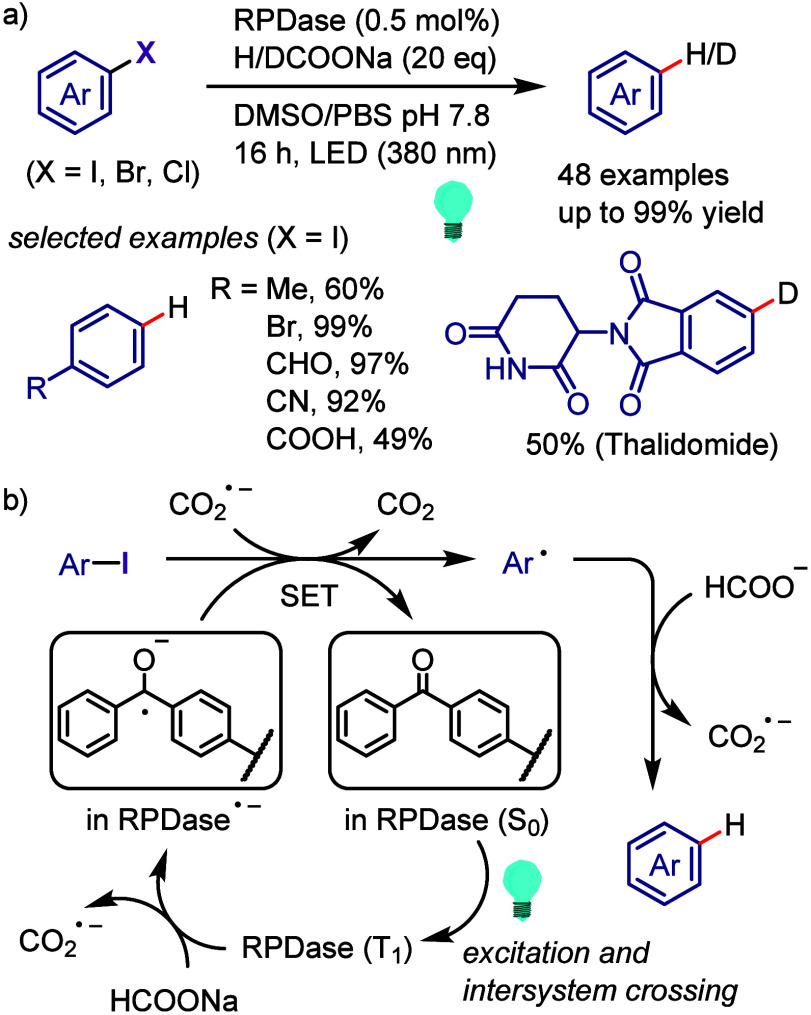
Artificial Photodehalogenase for the Hydrogenation and Deuteration of Aryl Halides

The merging of synthetic photoredox catalysis with natural proteins through genetic code expansion inspired Wang et al. to devise an artificial reductive photodehalogenase (RPDase) by encoding 4-benzoylphenylalanine at the 66th position of the superfolder yellow fluorescent protein followed by posttranslational intramolecular cyclization and oxidation to form a benzophenone-imidazolinone chromophore (Scheme 160a).^782^ This RPDase, in the presence of light and H(D)COONa as a sacrificial reductant, efficiently promoted the hydro(deutero)dehalogenation of sterically and electronically diverse (hetero)aryl halides and bioactive derivatives. Intriguingly, whole-cell biocatalysis using RPDase-expressing recombinant *Escherichia coli* enabled the efficient hydrodehalogenation of bioactive aryl halides. Control and spectroscopic experiments supported the mechanism outlined in Scheme 160b. According to this mechanism, the benzophenone moiety of the RPDase undergoes photoexcitation from S_0_ to S_1_ and subsequent intersystem crossing to T_1_ followed by reductive quenching with formate to afford the benzophenone radical anion (RPDase^•–^, *E*_0_ = −1.49 V in DMF) and CO_2_^•–^ (*E*_red_ = −2.2 V vs SCE). The reduction of the aryl halide by RPDase^•–^ or CO2^•–^ generates an aryl radical, which abstracts hydrogen from formate to yield the arene product and regenerate CO_2_^•–^ to propagate the chain mechanism.

**Scheme 161:**
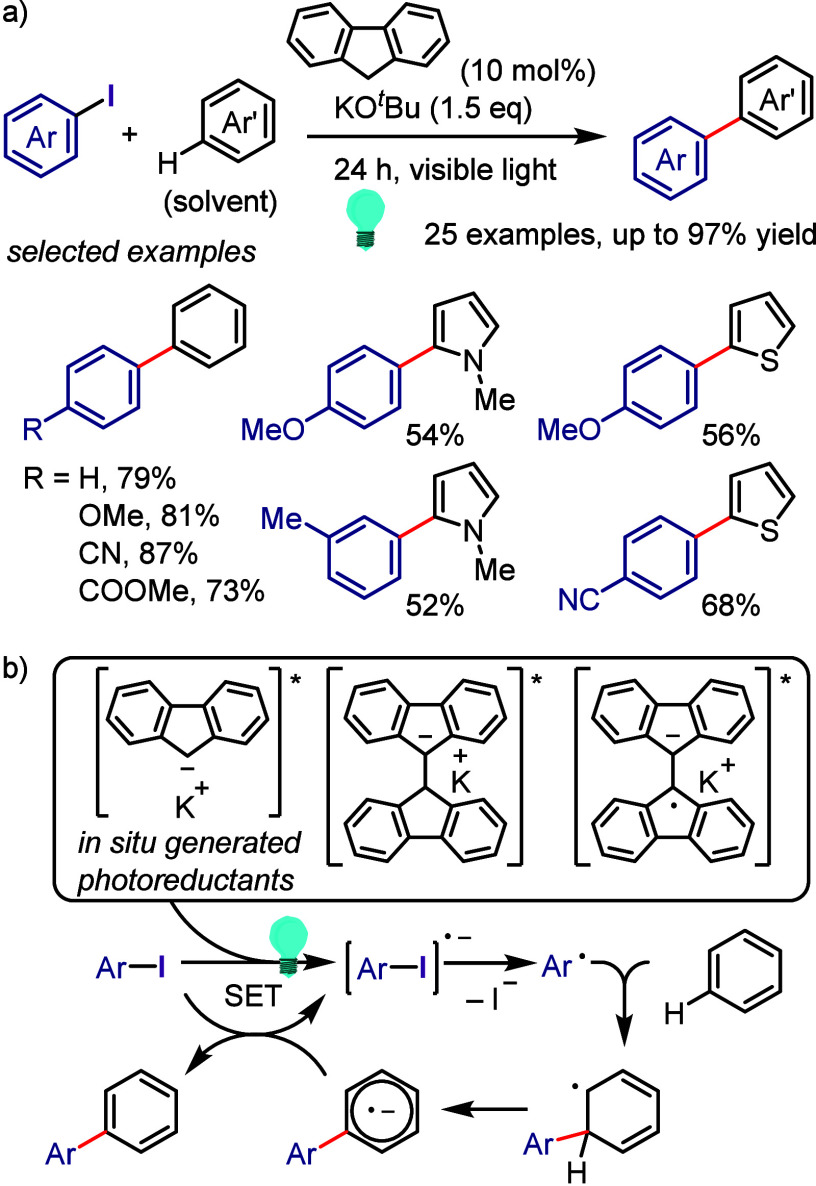
Fluorene-Mediated Reductive Cleavage of Ar–I Bonds in Biaryl Synthesis

Adhikari et al. showed that in the presence of KO^*t*^Bu and light, fluorene acts as an efficient radical initiator the for C–C coupling of aryl iodides with arenes producing (hetero)biaryls (Scheme 161a).^783^ The radical chain mechanism is initiated by the in situ conversion of fluorene to carbanion/radical anion species capable of SET in their excited states, which results in the reductive cleavage of the Ar–I bond (Scheme 161b). The remaining part of the mechanism is chain propagation, as in the case of BHAS.

**Scheme 162:**
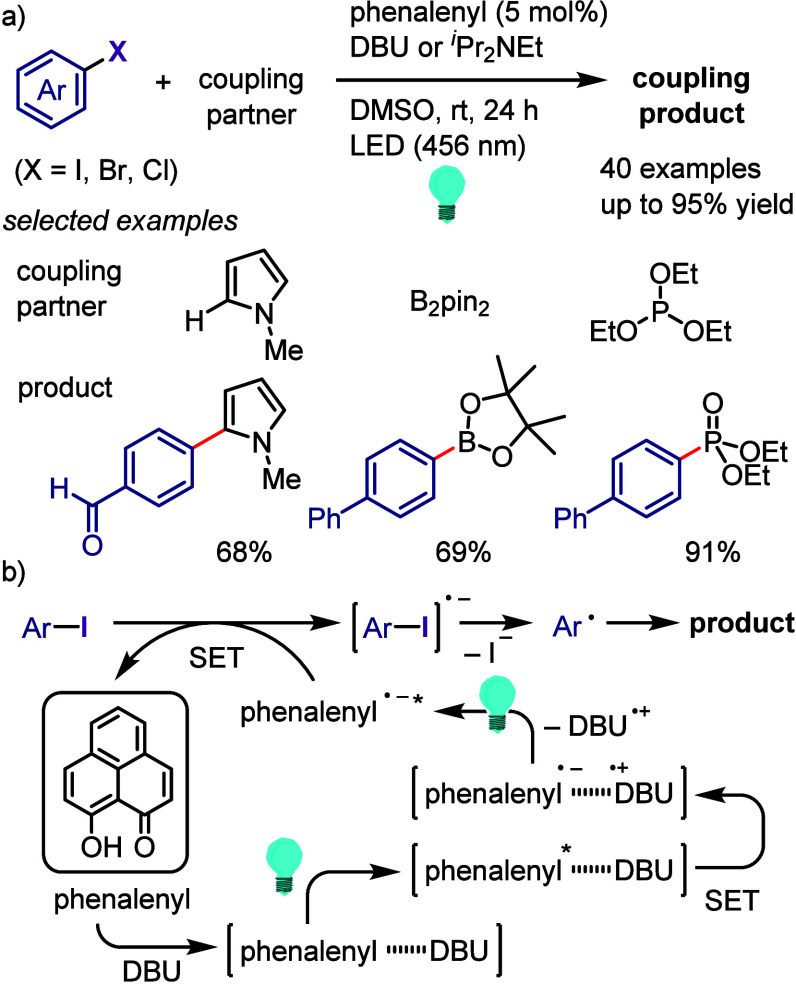
Phenalenyl-Photocatalyzed Reductive Functionalization of Aryl Halides

Besides the documented ground-state catalytic efficiency of phenalenyl-based scaffolds, Roy et al. explored the behavior of this system in the excited state, revealing that the excited phenalenyl radical anion is a potent reductant for the reductive cleavage of Ar–X bonds (Scheme 162a).^784^ This phenalenyl-based photocatalytic system was successfully used to functionalize aryl halides through hydrodehalogenation, coupling with unactivated arenes, and C–P and C–B bond formation. According to the results of electrochemical, spectroscopic, and DFT studies (Scheme 162b), the phenalenyl photocatalyst forms a complex with DBU, and subsequent photoexcitation and SET generate a solvated radical pair. The second photoexcitation of phenalenyl^•–^ generates an excited state (phenalenyl^•–^*) with a higher potential for ArX reduction. The thus generated aryl radical reacts with the coupling partner to afford the desired product, whereas the starting photocatalyst is obtained from phenalenyl^•–^* via SET.

**Scheme 163:**
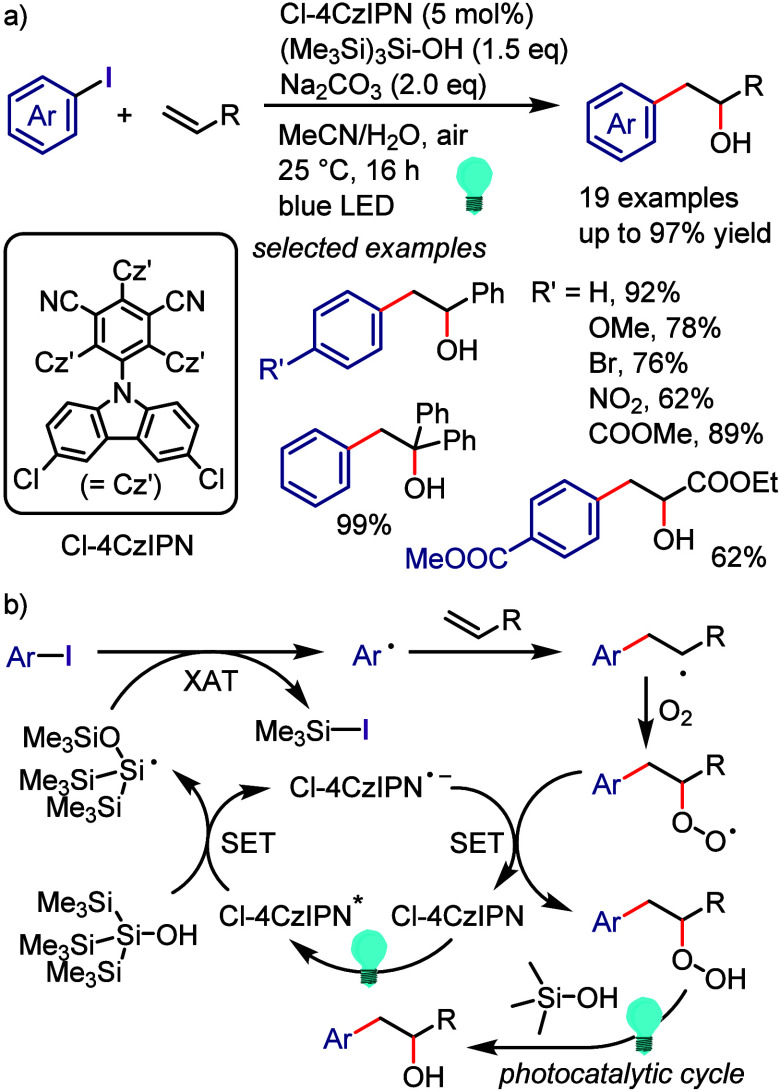
XAT-Based Three-Component Reaction of Aryl Iodides with Olefins and O_2_

Blakely et al. developed a photoredox multicomponent reaction of aryl iodides with olefins and O_2_ mediated by silyl radicals as halogen acceptors via XAT (Scheme 163a).^785^ The differential nucleophilicity of the *in situ* generated aryl radicals enabled the preferential reaction with olefins and O_2_ for sequential C–C and C–O bond formation, respectively. This protocol was applied to a wide range of alkenes to obtain the corresponding hydroxyarylated products. The hypothesized mechanism starts with the oxidation of silanol by the photoexcited Cl-4CzIPN to generate a silyl radical via the Brook rearrangement (Scheme 163b). XAT between the aryl halide and silyl radical affords an aryl radical, which adds to the olefin, and the subsequent reaction with triplet O_2_ affords a peroxy radical. The following single-electron reduction by the Cl-4CzIPN radical anion and reduction via a photocatalytic cycle induced by silanol afford the desired product.

**Scheme 164:**
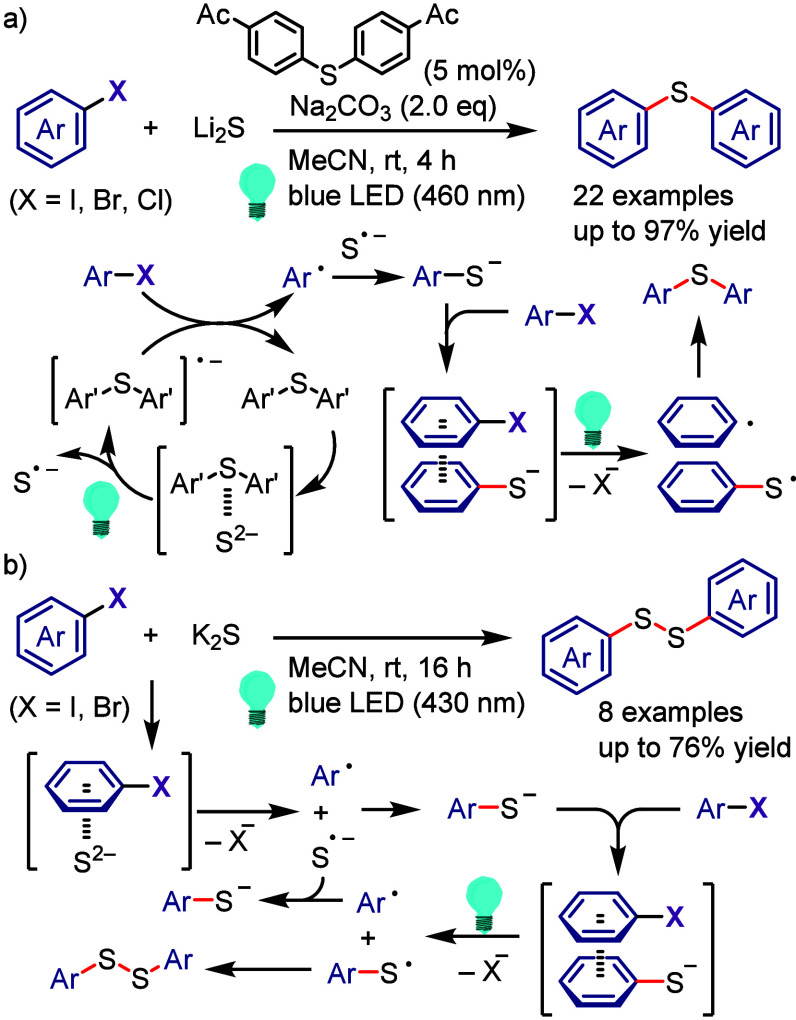
Sulfide Anion-Induced Photodissociation of Aryl Halides and C–S Bond Formation

Guo et al. used Li_2_S and K_2_S as sulfur sources and electron donors for the formation of EDA complexes, inducing the selective transformation of aryl halides into diaryl sulfides and diaryl disulfides under light irradiation (Scheme 164).^786^ In the presence of a diaryl sulfide photocatalyst and base, an EDA complex consisting of the photocatalyst and S^2–^ engages in SET upon irradiation to generate S^•–^ and a photocatalyst radical anion, which reduces the aryl halide via SET to form an aryl radical (Scheme 164a). The thus generated aryl radical reacts with S^•–^ to generate a thiolate anion, which forms an EDA complex with the aryl halide. Subsequent SET gives aryl and thiyl radicals, which undergo radical–radical coupling to afford the desired diaryl sulfide. When the reaction was conducted with excess K_2_S in the absence of a photocatalyst, diaryl disulfides were obtained instead of diaryl sulfides (Scheme 164b). The proposed mechanism starts with the formation of an EDA complex comprising S^2–^ and an aryl halide, with subsequent photoinduced SET affording an aryl radical and S^•–^. The following radical–radical coupling produces a thiolate anion, which interacts with the aryl halide to form an EDA complex that is converted into aryl and thiyl radicals under irradiation. The generated thiyl radical dimerizes to afford the diaryl disulfide, whereas the aryl radical is trapped by S^•–^ to form a thiolate anion.

### 4.3 Photoinduced Dissociation of Aryl–Iodonium Bonds

Light-induced chemical transformations have drawn considerable attention as elegant and ecofriendly approaches in organic chemistry.^787−792^ UV–vis light is a clean, green, convenient, and renewable energy source used to promote chemical reactions through the absorption-induced generation of reactive excited states that engage in reactions or are converted into products inaccessible under traditional thermal conditions.

The photolysis of diaryliodonium salts produces aryl radicals, which can participate in a series of transformations.^793−796^ We discuss the recently reported metal- and organometallic photocatalyst-free visible light-induced arylation reactions involving diaryliodonium salts and affording C–C and C–heteroatom bonds. The versatility of diaryliodonium salts as photoinitiators for the synthesis of polymers has been extensively reviewed and is outside the scope of the present work.^797−809^

#### 4.3.1 Photocatalyst-Free Dissociation of Aryl–Iodonium Bonds

The photochemical reactions of diaryliodonium salts are initiated by an interaction between the iodonium center and an electron donor, which affords an EDA complex with photochemical properties superior to those of the parent iodonium substrate. The photoexcitation of the EDA complex enables internal SET, which generates an aryl radical that subsequently undergoes radical–radical coupling or is trapped by a radical acceptor to give the desired product.

**Scheme 165:**
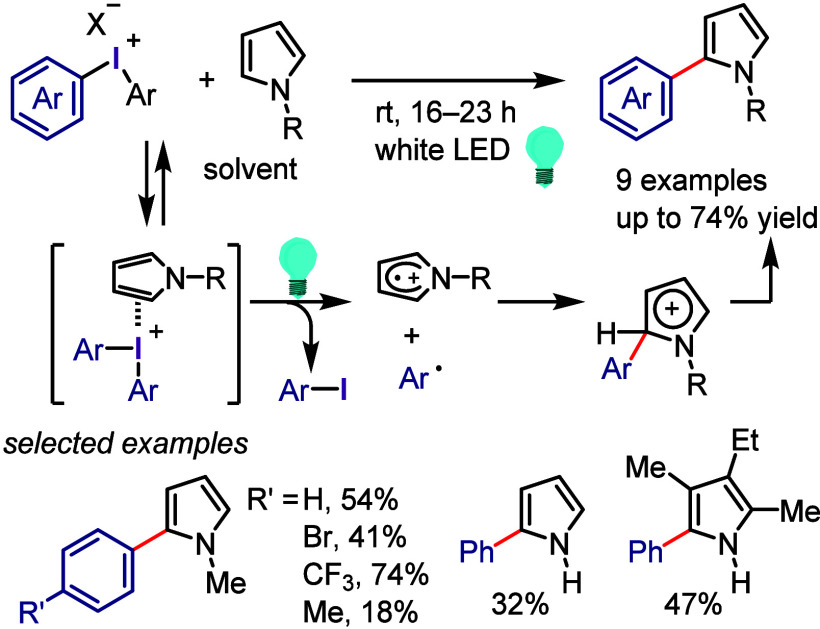
Photoinduced Arylation of Pyrroles with Diaryliodonium Salts

Tobisu and Chatani reported that the arylation of *N*-methylpyrrole with diaryliodonium salts affords 2-arylpyrroles under visible light in the absence of photocatalysts (Scheme 165).^810^ The mechanism proposed based on the results of UV–vis spectroscopic analysis indicates an interaction between pyrrole and the iodonium salt through the formation of a CT complex. The photoexcitation of this complex generates a pyrrole radical cation and an aryl radical along with an aryl iodide via SET. The subsequent coupling reaction affords a 2-arylpyrrole cation, which is deprotonated to furnish an arylated pyrrole.

**Scheme 166:**
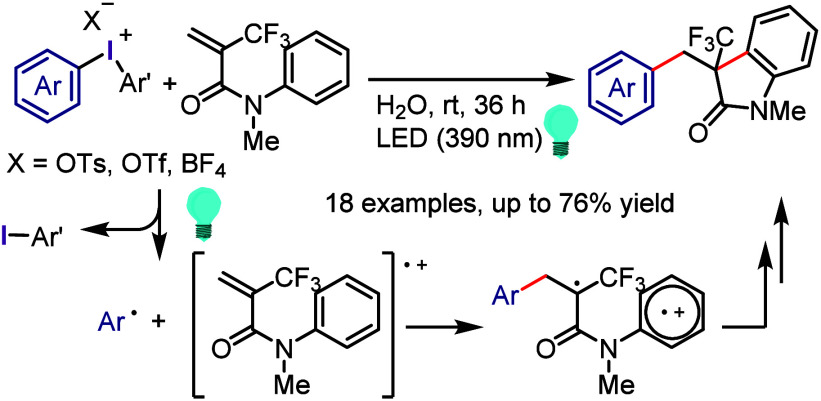
Visible Light-Induced Synthesis of Oxindoles Involving an Arylation–Cyclization Cascade

Yadav et al. synthesized quaternary CF_3_-substituted oxindoles via an arylation–cyclization cascade under visible-light irradiation, using diaryliodonium salts as aryl radical sources for the reaction with *N*-aryl-2-(trifluoromethyl)acrylamide (Scheme 166).^811^ According to the proposed mechanism, the photoexcitation of the iodonium salt followed by SET with the acrylamide generates an aryl radical and acrylamide radical cation. Coupling followed by cyclization and aromatization affords the desired product.

**Scheme 167:**
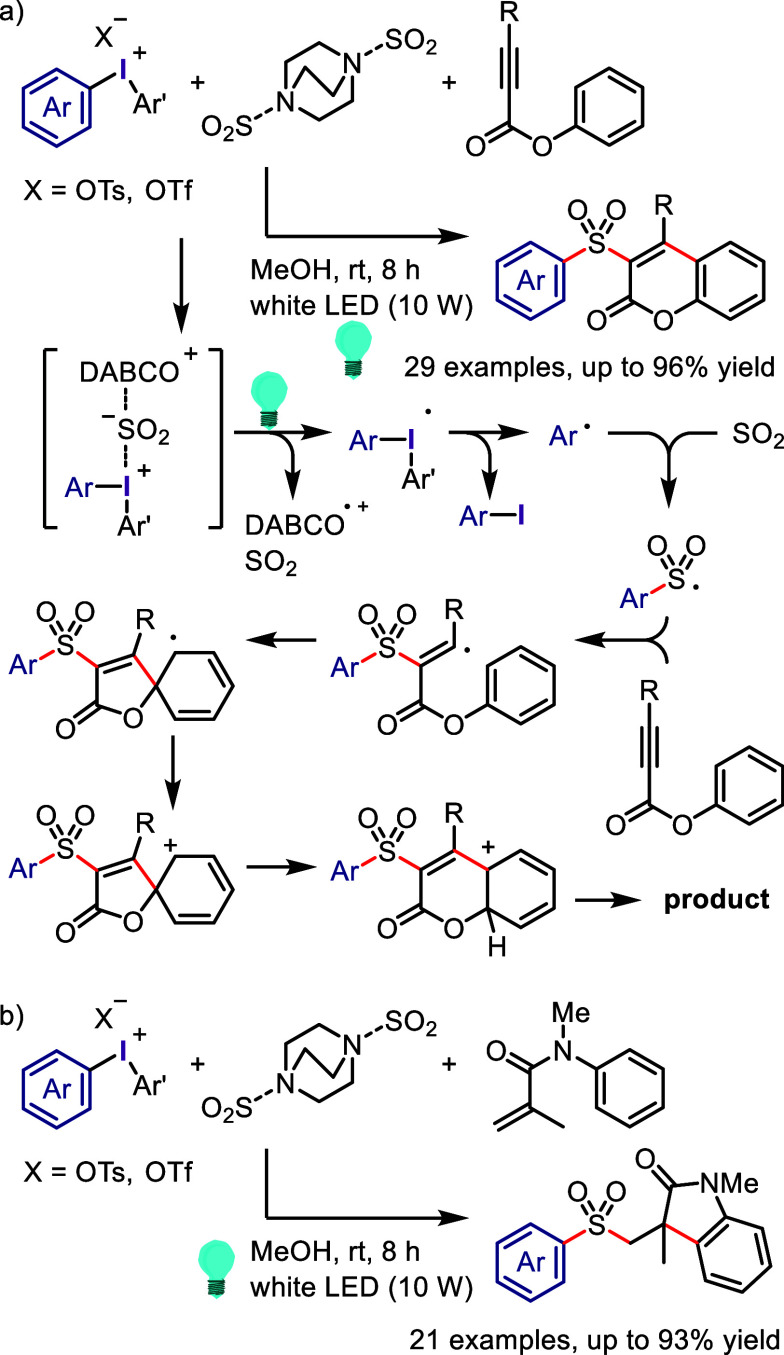
Photoinduced Three-Component Synthesis of Sulfonylated Coumarins and Oxindoles

Manolikakes et al. developed a visible light-induced three-component reaction between a diaryliodonium salt, an arylpropynoate, and DABSO affording the corresponding sulfonylated coumarin (Scheme 167a).^812^ Sunlight irradiation provided a comparable yield. In the proposed mechanism, DABSO serves as an SO_2_ source and diaryliodonium salt activator. The interaction between DABSO and the iodonium salt results in the formation of a CT complex, which engages in SET under irradiation to generate an aryl radical. This radical reacts with SO_2_ to generate an arylsulfonyl radical, which is intercepted by an alkynyl ester to generate an alkenyl radical that undergoes spirocyclization followed by oxidation to generate a cationic spirocyclized intermediate. The following 1,2-ester migration and sequential rearomatization and deprotonation afford the coumarin product. Analogously, the one-pot reaction of a diaryliodonium salt, *N*-arylacrylamide, and DABSO under visible-light irradiation delivered the corresponding sulfonylated oxindole (Scheme 167b).^813^

**Scheme 168:**
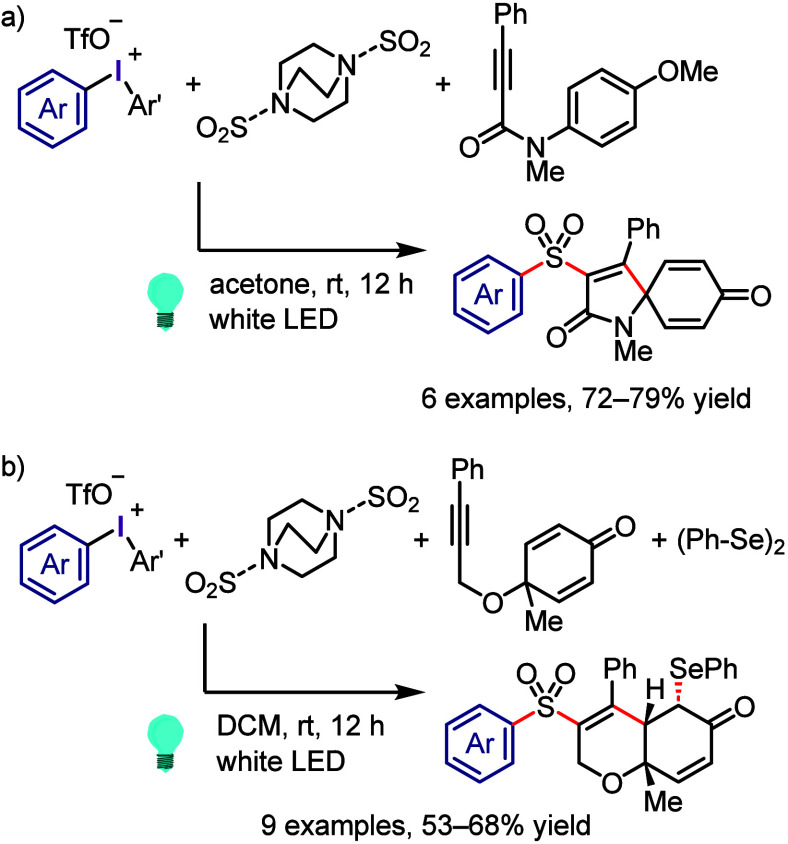
Photoinduced Multicomponent Synthesis of Sulfonylated Bicyclic Compounds

Volla et al. performed the spirocyclization of alkenyl and alkynyl amides using diaryliodonium salts and DABSO under white-light irradiation to generate the corresponding spirocycles (Scheme 168a).^814^ A four-component cascade reaction was reported by the same group. The reaction of diaryliodonium triflates, DABSO, an alkynyl cyclohexadienone, and diphenyldiselenide under white-light irradiation afforded substituted dihydrochromenones with high regio- and diastereoselectivities (Scheme 168b).^815^

**Scheme 169:**
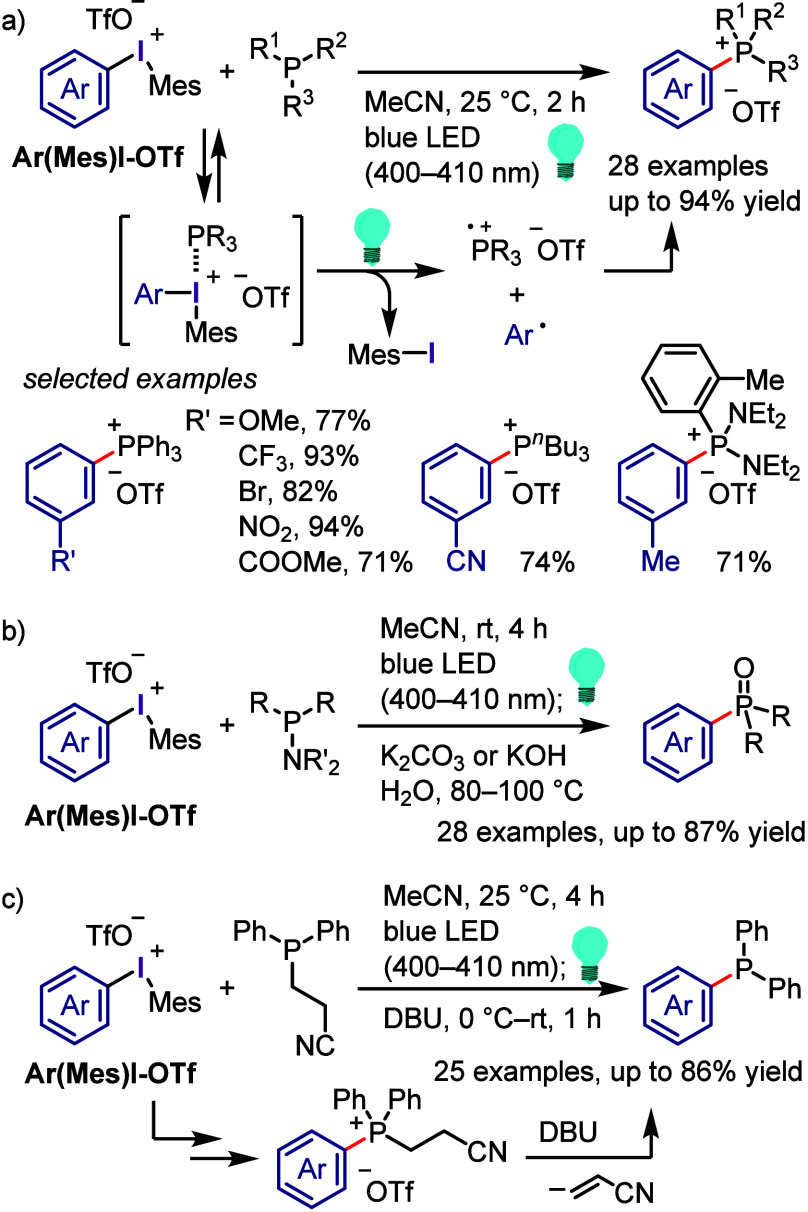
Photoinduced Arylation of Phosphines with Iodonium Salts

Karchava et al. reported the arylation of phosphine derivatives with aryl(Mes)iodonium triflate (**Ar(Mes)I-OTf**) affording arylphosphonium salts under visible-light irradiation (Scheme 169a).^816^ The proposed mechanism starts with the formation of an EDA complex between the Mes-iodonium salt and tertiary phosphine, which engages in SET under photoirradiation to generate aryl and phosphorus-centered radicals. The combination of these radicals affords the desired arylphosphonium salt. Further expansion was performed by carrying out the one-pot coupling of aminophosphines with **Ar(Mes)I-OTf** to generate phosphonium salts under blue-light irradiation, with subsequent hydrolysis affording the corresponding arylphosphine oxides (Scheme 169b).^817^ The same group developed an indirect route to unsymmetrical tertiary arylphosphines (Scheme 169c).^818^ The one-pot reaction of (2-cyanoethyl)diphenylphosphine with **Ar(Mes)I-OTf** under visible-light irradiation followed by the treatment of the thus generated quaternary phosphonium salts with DBU furnished the corresponding tertiary arylphosphines via a retro-Michael reaction.

**Scheme 170:**
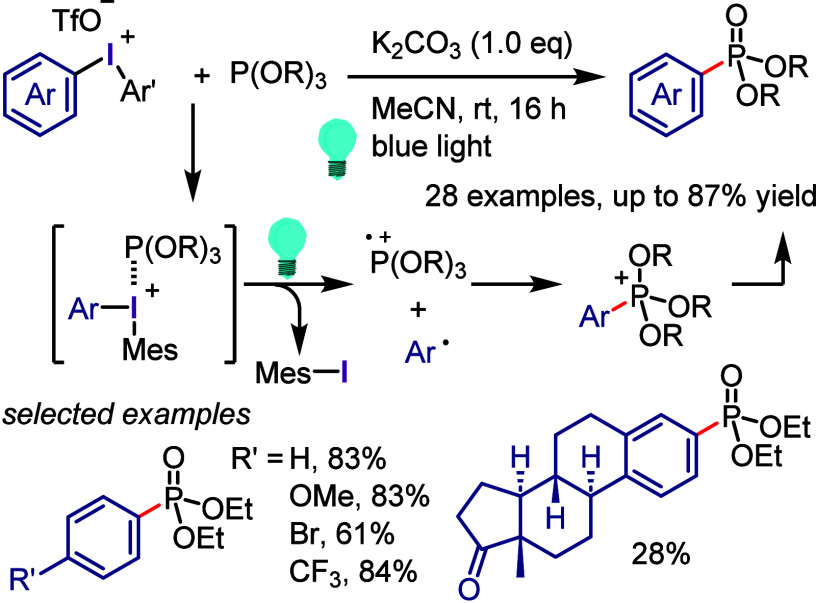
Light-Promoted Reaction of Iodonium Salts with Trialkylphosphites Affording Arylphosphonates

Lakhdar et al. synthesized arylphosphonates from diaryliodonium salts and trialkylphosphites under blue-light irradiation (Scheme 170).^819^ According to the mechanism proposed based on the results of experimental and theoretical studies, the low electron-donating ability of phosphites leads to the formation of weak EDA complexes with iodonium salts. SET under blue-light irradiation affords an aryl radical and a phosphorus-centered radical cation, which combine to generate arylphosphonium salts. Nucleophilic displacement in the presence of a base affords the desired product.

#### 4.3.2 Organophotocatalyzed Aryl–Iodonium Bond Dissociation

**Scheme 171:**
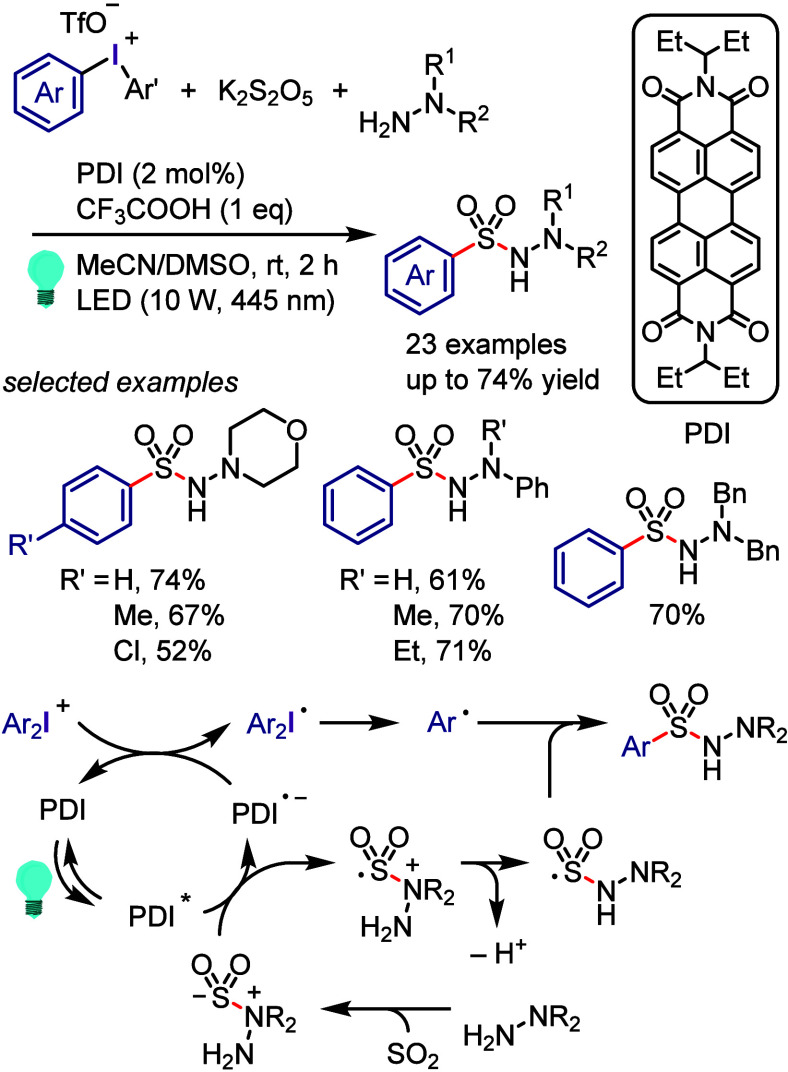
Perylenediimide-Promoted Reactions of Iodonium Salts with Hydrazine and SO_2_ under Visible-Light Irradiation

Manolikakes et al. synthesized *N*-aminosulfonamides via the photoinduced three-component reactions of diaryliodonium salts, substituted hydrazines, and SO_2_ surrogates (DABSO or K_2_S_2_O_5_/TFA) in the presence of perylenediimide (PDI) as a photoredox organocatalyst (Scheme 171).^820^ The reaction mechanism starts with the photoexcitation of PDI to generate PDI*. Concomitantly, the liberated SO_2_ interacts with hydrazine to afford a zwitterion, which is oxidized by PDI* via SET and deprotonated to generate a sulfonyl radical. SET from the reduced catalyst (PDI^•–^) to the aryl halide affords an aryl radical, which undergoes radical–radical coupling with the sulfonyl radical to afford the desired product.

**Scheme 172:**
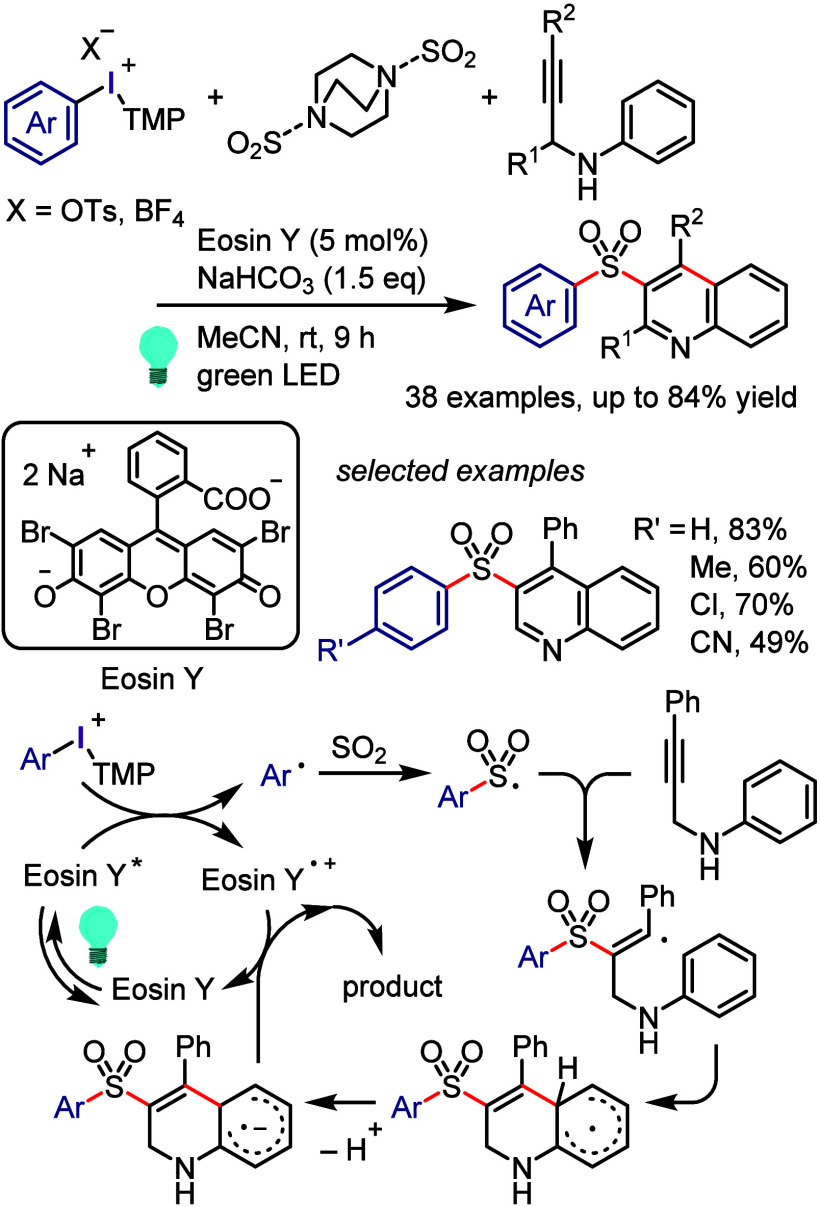
Photocatalytic Synthesis of 3-Arylsulfonylquinolines under Green-Light Irradiation

Zhang et al. developed a three-component cascade reaction of diaryliodonium salts with *N*-propargyl aromatic amines and DABSO catalyzed by Eosin Y under visible-light irradiation for the synthesis of 3-arylsulfonylquinolines (Scheme 172).^821^ According to the proposed mechanism, Eosin Y is photoexcited upon irradiation with green LED light to produce Eosin Y*, which engages in SET with an iodonium salt to generate an aryl radical and Eosin Y^•+^. The aryl radical reacts with DABSO to give an arylsulfonyl radical, which is regioselectively trapped by the triple bond of propargylamine to generate the corresponding alkenyl radical. Intramolecular cyclization followed by deprotonation furnishes a radical anion, which is oxidized by Eosin Y^•+^ or the iodonium salt to regenerate Eosin Y or the aryl radical and yield the desired product.

**Scheme 173:**
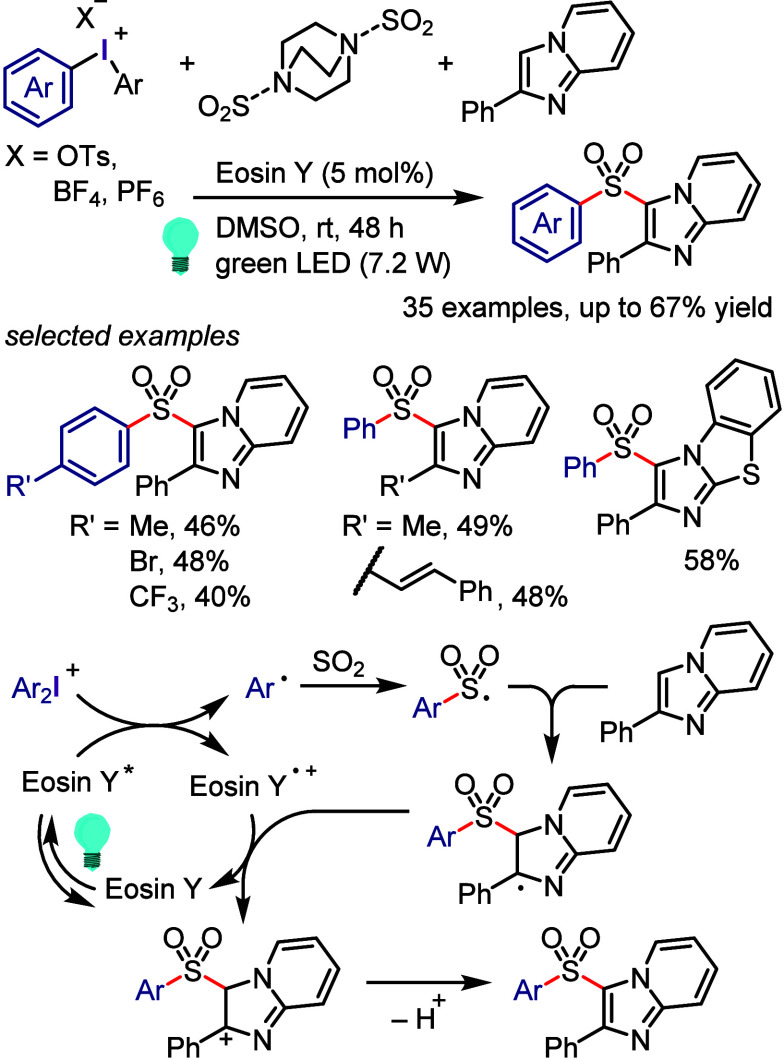
Photoinduced Selective C–H Arylsulfonylation of Imidazoheterocycles

Piguel et al. reported a three-component reaction of iodonium salts with DABSO and an imidazoheterocycle in the presence of EosinY under green-light irradiation, wherein the C-3 sulfonylation of the imidazoheterocycles proceeded via C–H arylsulfonylation (Scheme 173).^822^ The reaction mechanism is analogous to that shown in Scheme 171. An arylsulfonyl radical generated by Eosin Y* reacts with the imidazoheterocycle at C-3 to form a radical intermediate. SET with Eosin Y^•+^ and subsequent deprotonation afford the desired product.

**Scheme 174:**
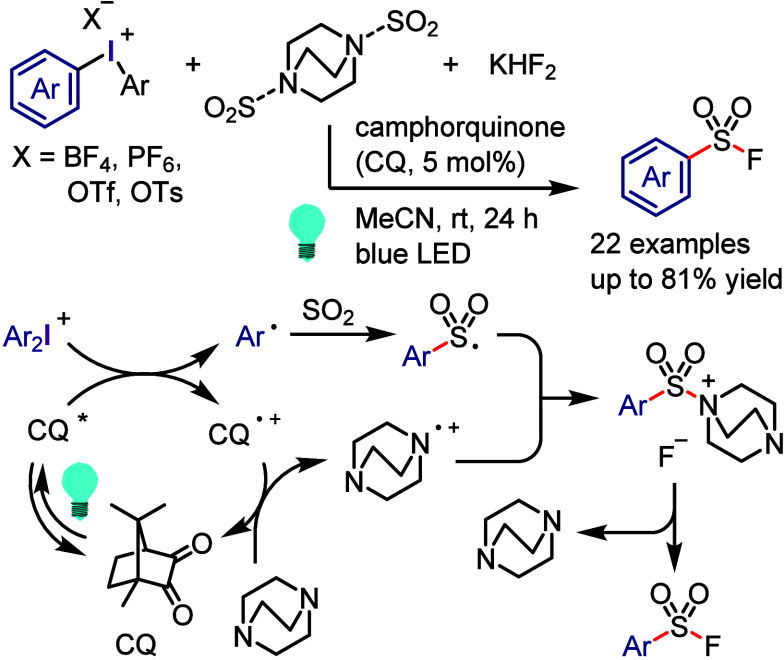
Photocatalytic Synthesis of Arylsulfonyl Fluorides from Iodonium Salts, DABSO, and KHF_2_

Ma et al. applied arylsulfonyl radical generation upon irradiation to the fluorosulfonylation of diaryliodonium salts using DABSO and KHF_2_ in the presence of camphorquinone (CQ) as an organophotocatalyst (Scheme 174).^823^ According to the proposed mechanism, blue-LED irradiation converts CQ to an excited state, which reduces the employed iodonium salt to an aryl radical and generates CQ^•+^. The coupling of the aryl radical with SO_2_ from DABSO affords an arylsulfonyl radical, whereas CQ^•+^ reduces DABCO to the DABCO radical cation. These species combine in the presence of KHF_2_ to form a sulfonate intermediate, which loses DABCO to generate an arylsulfonyl fluoride.

**Scheme 175:**
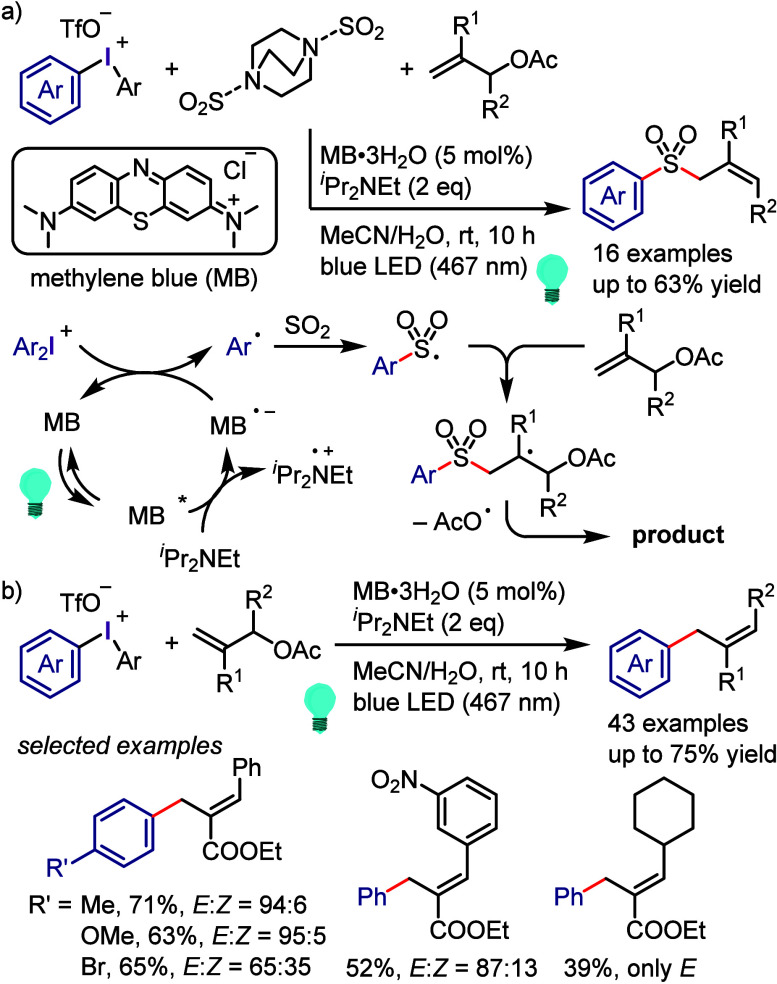
Photoinduced Arylsulfonation and Arylation of Allyl Acetate with Diaryliodonium Triflates

Murarka et al. used methylene blue trihydrate as an efficient photoredox organocatalyst for the arylsulfonylation of Morita–Baylis–Hillman acetates by diaryliodonium triflates in the presence of DABSO (Scheme 175a).^824^ The proposed mechanism starts with the photoexcitation of the catalyst, which is followed by reductive quenching with DIPEA via SET to generate the highly reducing MB^•–^ radical anion, which reduces the diaryliodonium salt to regenerate MB and form an aryl radical. The sequential addition of this radical to SO_2_ and the double bond of allyl acetate affords the corresponding alkyl radical, which expels an acetate radical to afford the desired product. When the reaction was carried out in the absence of DABSO, the aryl radical directly reacted with allyl acetate to generate the corresponding allylarene (Scheme 175b).

**Scheme 176:**
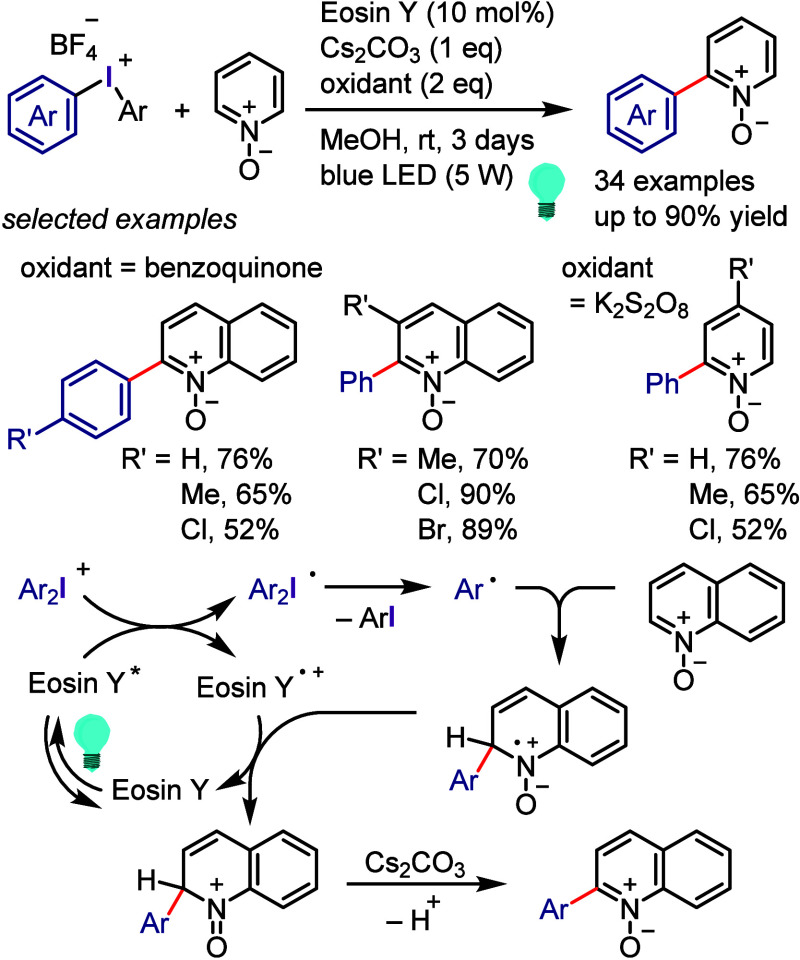
Photoinduced Arylation of Quinoline and Pyridine *N*-Oxides with Diaryliodonium Salts

Quinoline and pyridine *N*-oxides were selectively arylated at C-2 with diaryliodonium tetrafluoroborates in the presence of Eosin Y as a photocatalyst under visible light to afford the corresponding *N*-heterobiaryls (Scheme 176).^825^ The proposed mechanism starts with the excitation of Eosin Y by blue light, and the thus generated Eosin Y* is converted into an aryl radical and EosinY^•+^. The addition of the aryl radical to quinoline *N*-oxide followed by oxidation with EosinY^•+^ generates an *N*-oxide cation that is deprotonated to generate the desired 2-arylated product. 1,4-Benzoquinone and K_2_S_2_O_8_ were found to be important additives promoting deprotonation and oxidizing EosinY, respectively.

**Scheme 177:**
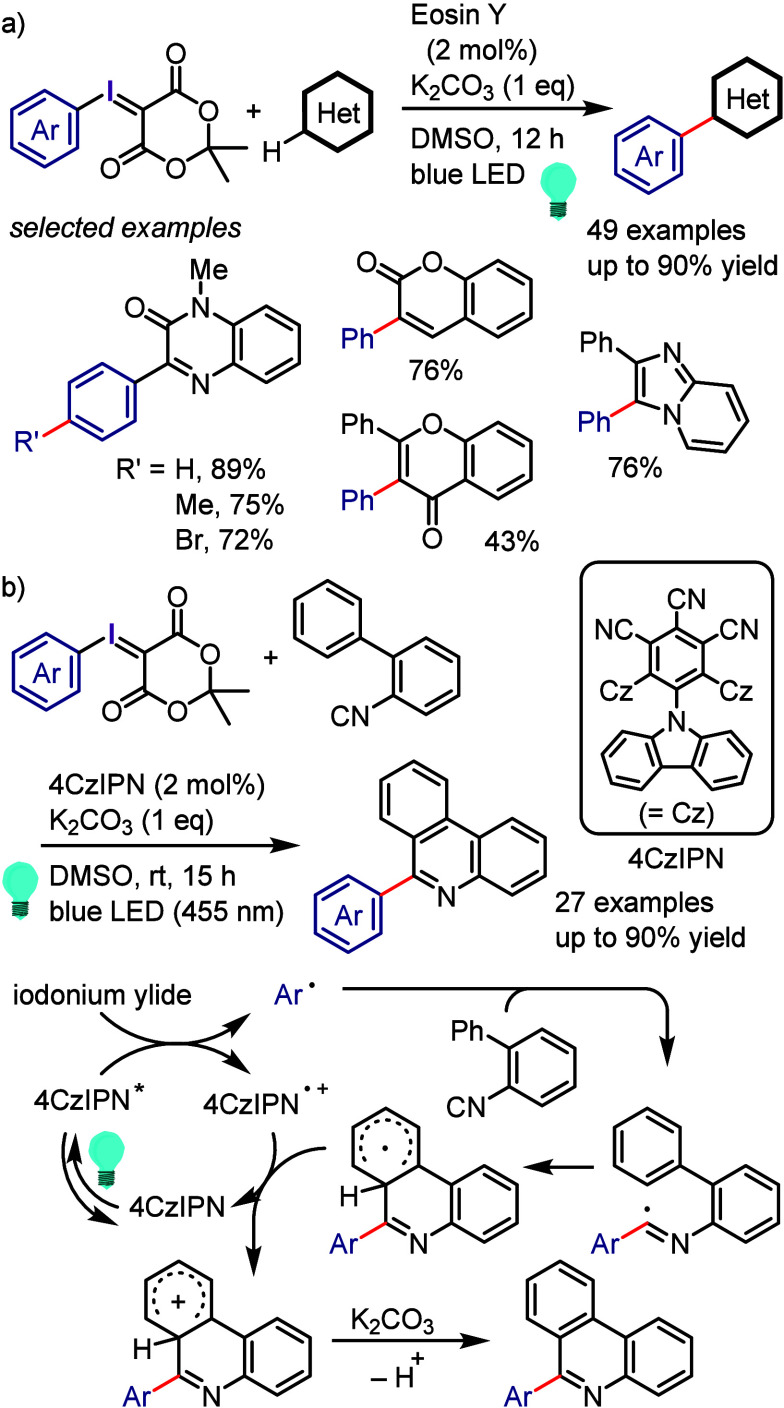
Generation of Aryl Radicals from Iodonium Ylides under Photoredox Conditions

Aryliodonium ylides can also generate aryl radicals^18,826−828^ under irradiation in the presence of EosinY, which was applied to the direct C(sp^2^)-H arylation of diverse heterocycles (Scheme 177a).^829^ Aryliodonium ylide screening indicated the broad applicability of this reaction and its excellent compatibility with diverse functional groups and bioactive compounds. The ability of 4CzIPN to induce the generation of aryl radicals from aryliodonium ylides under irradiation was used for the sequential arylation/cyclization of 2-isocyanobiaryls to generate 6-arylated phenanthridines (Scheme 177b).^830^ The proposed mechanism starts with the photoexcitation of 4CzIPN, with the subsequent SET generating an aryl radical and 4CzIPN^•+^. The addition of the aryl radical to the isocyano group affords an imidoyl radical, which is converted to a cyclized radical. The following oxidation by 4CzIPN^•+^ via SET affords the desired product after deprotonation.

**Scheme 178:**
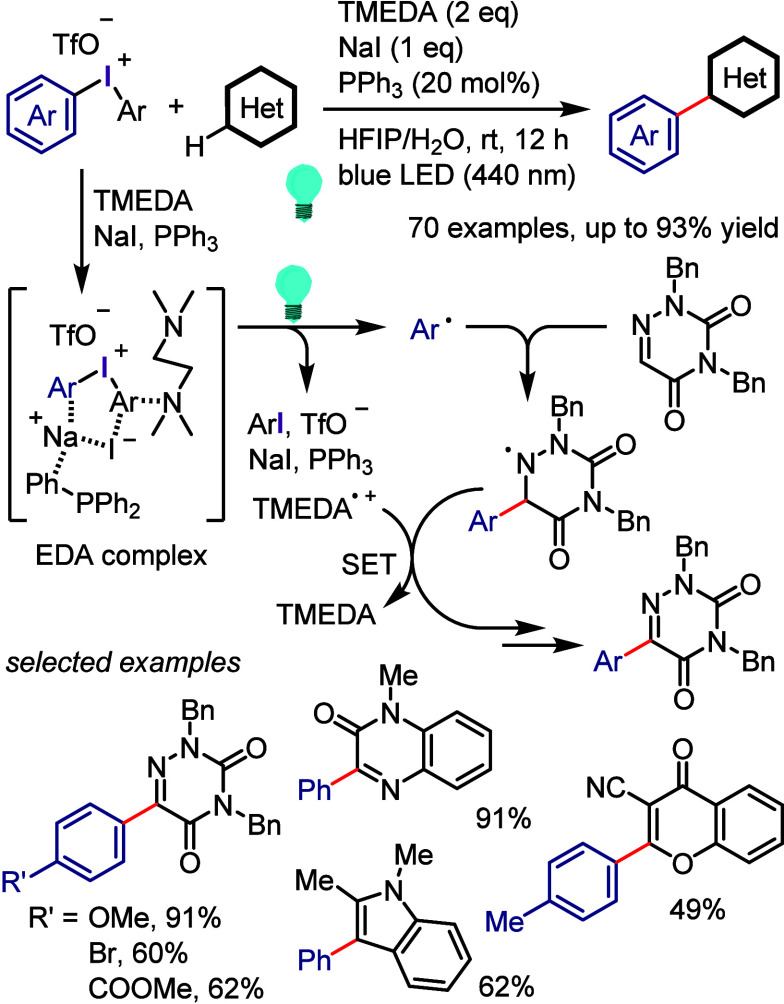
Photoactivation of Iodonium Salts by an Electron-Donor Triad (Na/Ph_3_P/*N*,*N*,*N*′,*N*′-Tetramethylethylenediamine (TMEDA)) for C–H Arylation

Roy et al. designed a general photoredox system comprising NaI, Ph_3_P, and *N*,*N*,*N*′,*N*′-tetramethylethylenediamine (TMEDA) for the activation of diaryliodonium salts and generation of aryl radicals, which engaged in the C–H arylation of heterocycles (Scheme 178).^831,832^ This protocol was compatible with a broad array of aromatic and nonaromatic heterocycles, i.e., azauracils, quinoxaline-2-ones, cinnolinones, imidazopyridines, indazoles, pyrazinones, pyrazines, quinoline *N*-oxides, isoquinolines, and indoles. Detailed mechanistic investigations indicated the formation of a tetrameric EDA complex between the diaryliodonium salt, NaI, Ph_3_P, and TMEDA, with the irradiation-based excitation of this complex followed by SET generating an aryl radical, NaI, Ph_3_P, and TMEDA^•+^. The solvent (HFIP/H_2_O)-assisted nucleophilic addition of the aryl radical to azauracil affords a radical intermediate that engages in SET with NaI, Ph_3_P and TMEDA^•+^ to regenerate the photoredox system and produce a cationic intermediate. The deprotonation of the latter in the presence of the triflate anion gives the desired product.

## 5 Electrochemical Dissociation of Ar–I Bonds

**Scheme 179:**
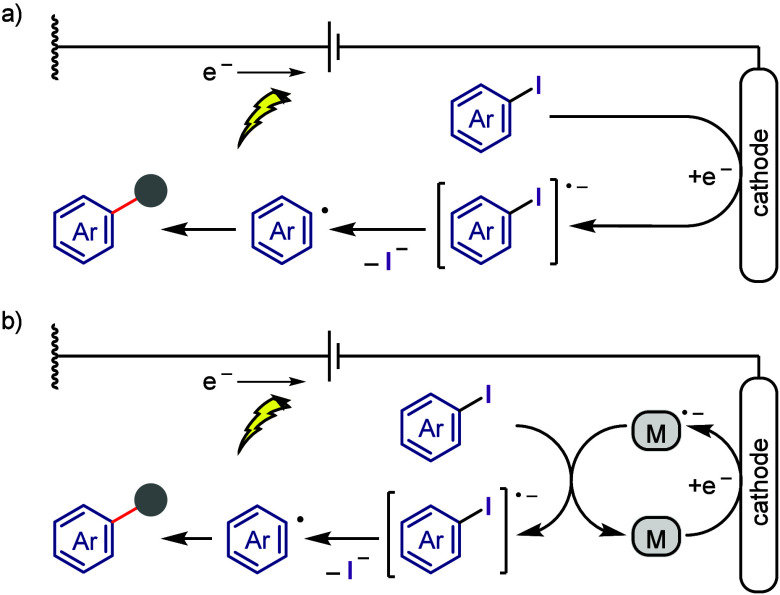
Electrochemical Processes for Aryl Halide Activation

The conventional chemical transformations used to realize arylation with aryl halides suffer from a narrow substrate scope and the use of expensive metals and/or ligands or strong bases beside other environmental and practical concerns. Hence, efficient, green, and sustainable alternative synthetic transformations under benign conditions are urgently required. In this context, electrochemistry attracts growing interest and has emerged as a versatile and green strategy for broadening the scope of and complementing the current arylation approaches to realize challenging arylation reactions by applying electric current as a driving force.^624,625,736,833−844^ The high tunability of electrochemical processes via applied current or potential adjustment makes them well suited for numerous redox reactions. These processes are ecofriendly, as they employ electrons as green redox reagents instead of the environmentally deleterious chemical reagents, which lowers the associated risks, costs, and waste production while increasing the atom/step economy.^845−853^ Classical electrochemical reactions are initiated by SET from the electrode surface to the substrate in a heterogeneous process (direct electrolysis) (Scheme 179). Alternatively, a chemical substance (mediator, M) can be used in catalytic amounts as a redox reagent to shuttle SET (through a homogeneous process) to the substrate and initiate the electrochemical transformation (indirect electrolysis). Indirect electrolysis is superior to its direct counterpart, allowing one to (i) avoid substrate overreduction and mitigate electrode passivation, (ii) eliminate kinetic inhibition and overpotentials and thus accelerate the electrochemical process, (iii) achieve a high and/or different selectivity, and (iv) conduct electrolysis at a potential lower than the redox potential of the substrate, which leads to the reaction proceeding under milder conditions and exhibiting a broad functional group tolerance.

The reduction potential, bond dissociation energy, polarizability, and Ar–X bond cleavage mechanism^484^ of iodoarenes favor the dissociation of Ar–I bonds under mild conditions, which results in a broad substrate scope and sustainability. In this part of the review, we demonstrate the crucial role of electric current as a green energy source for aryl iodide reduction/activation at the cathode and the generation of aryl radicals suitable for further functionalization.^844,854^

### 5.1 Direct Dissociation of Ar–X Bonds

The electrochemical reductive transformations of aryl halides can be achieved by direct electrolysis or indirect electrolysis mediated by redox catalysts.^624,625,845,849,850^ Direct electrolysis is facilitated by the cathode-to-ArX SET and generation of highly reactive radical ions and radical intermediates, requiring the subsequent oxidation of a sacrificial reductant (i.e., anode erosion or use of cheap reductants with lower potentials, such as Et_3_N) to maintain the charge balance.

**Scheme 180:**
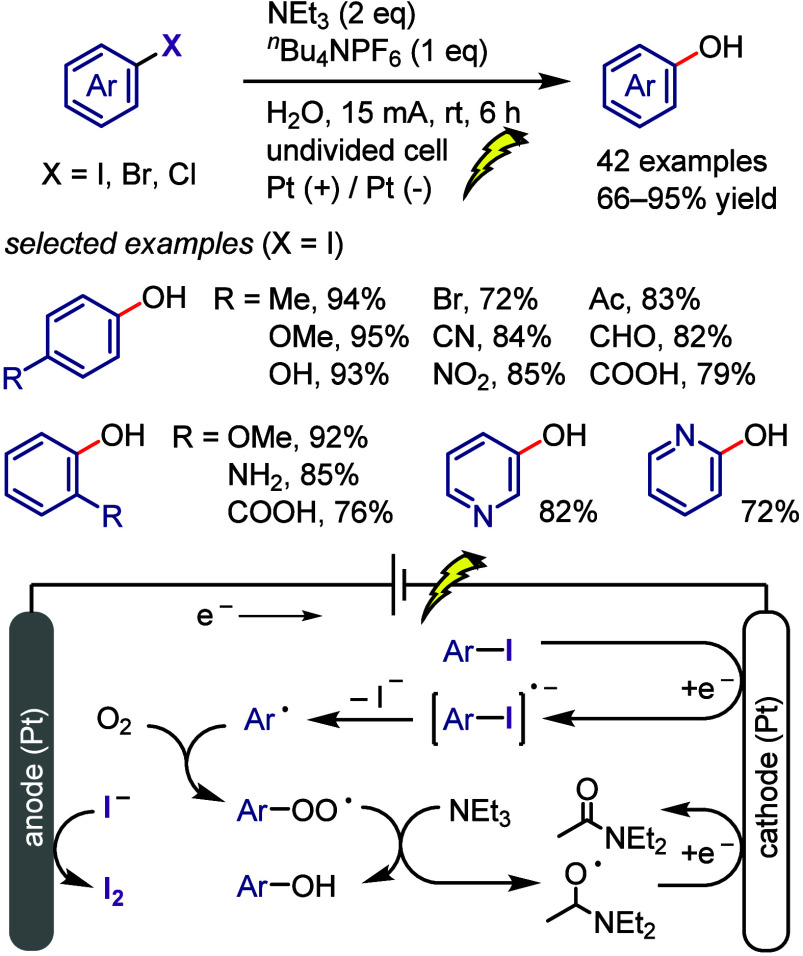
Electrochemical Hydroxylation of Aryl Halides

Ke et al. synthesized phenols via electrochemically driven Ar–X bond dissociation in the presence of H_2_O/Et_3_N/air (Scheme 180).^855^ The electrochemical process is hypothesized to start with the cathodic reduction of an aryl iodide to generate a radical anion, which undergoes Ar–I bond mesolysis to generate an aryl radical and liberate an iodide anion. The aryl radical reacts with molecular oxygen to form a peroxy radical, which is converted into the corresponding phenol in the presence of Et_3_N as a sacrificial reductant. The anodic oxidation of iodide affords molecular iodine.

**Scheme 181:**
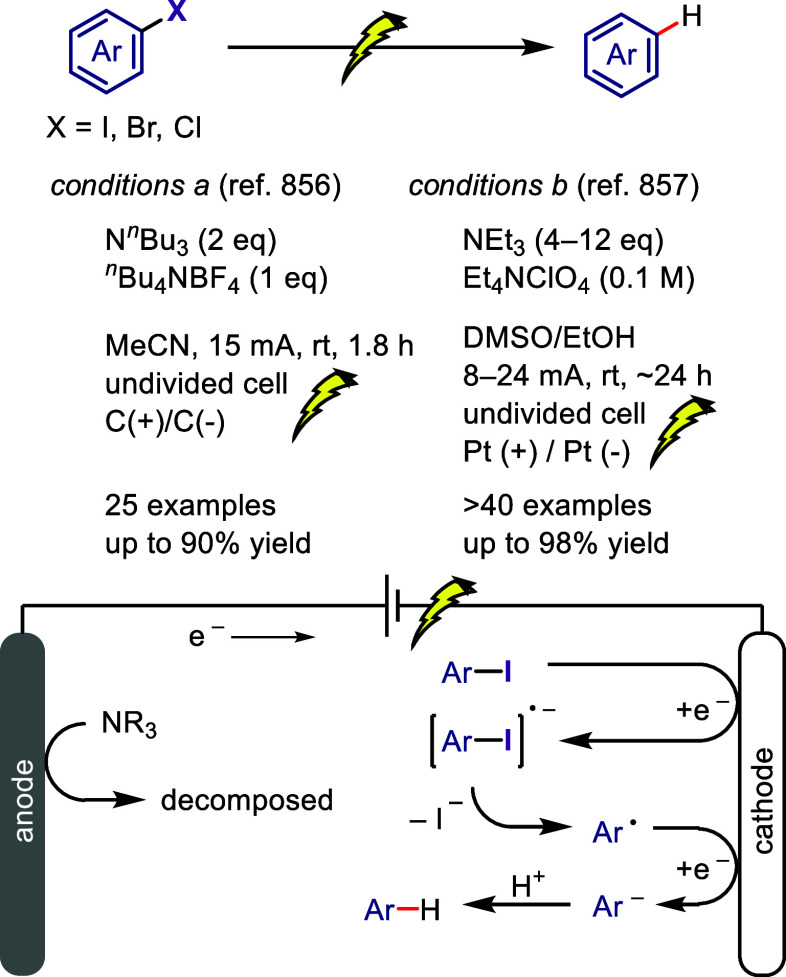
Electrochemical Hydrodehalogenation of Aryl Halides

The Pan and Chi group reported the electrochemical hydrodehalogenation of aryl halides, showing that unlike that described previously, it did not require a divided cell and metallic electrode but featured a narrow substrate scope (Scheme 181, conditions a).^856^ Guo et al. designed a more applicable and controllable electrolysis protocol for haloarene hydrodehalogenation (conditions b).^857^ Ethanol, added as a cosolvent, competed with electrosensitive substrate moieties and consequently prevented overreduction and decomposition. In both reaction systems, the main feature of electrolysis on the cathode is the reduction of the aryl iodide to a radical anion, which undergoes Ar–I bond mesolysis to generate an aryl radical. The aryl radical is preferentially reduced to the corresponding aryl anion, which abstracts a proton from the solvent, Et_3_N, or moisture to afford the hydrogenated product. At the anode, the tertiary amine acts as a sacrificial reductant and provides electrons for the cathodic reduction.

**Scheme 182:**
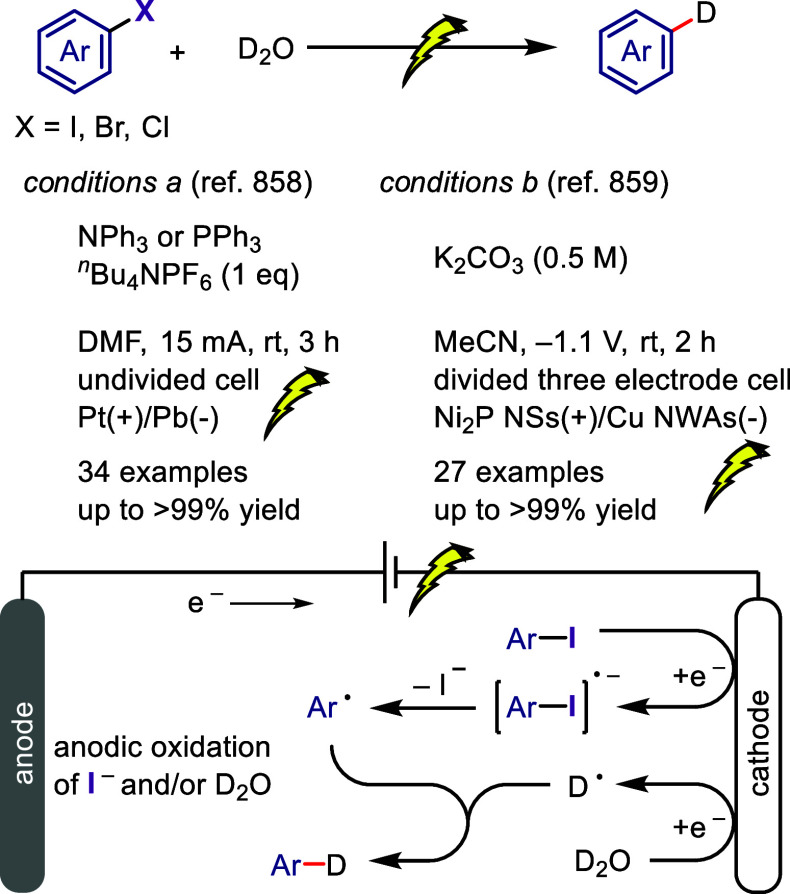
Electrochemical Dehalogenative Deuteration of Aryl Halides with D_2_O

This electrochemical hydrodehalogenation of aryl halides can be applied to reductive deuteration (Scheme 182). Lei et al. used an undivided cell with Pt and Pb electrodes (conditions a).^858^ In this case, the proposed mechanism involves the concomitant reduction of an aryl halide and D_2_O at the cathode to generate aryl and deuterium radicals, respectively, which undergo radical–radical coupling to afford a deuterated arene. Zhang et al. fabricated a copper nanowire array (NWA) cathode in situ through the electrochemical reduction of CuO NWAs on Cu foil and used it for the electrochemical deuteration of aryl halides with D_2_O as the sole deuterium source (conditions b).^859^ In view of its large surface area, the Cu NWA cathode outperformed Cu foil, Pt, carbon paper, and Ni foam cathodes. Interestingly, the one-pot conversion of Ar–H bonds to Ar–D bonds through a sequence of halogenation and deuterodehalogenation was efficiently applied to the site-specific deuteration of C–H (hetero)arenes and pharmaceuticals. Furthermore, paired reactions at the cathode and anode without additional oxidants are also possible.

**Scheme 183:**
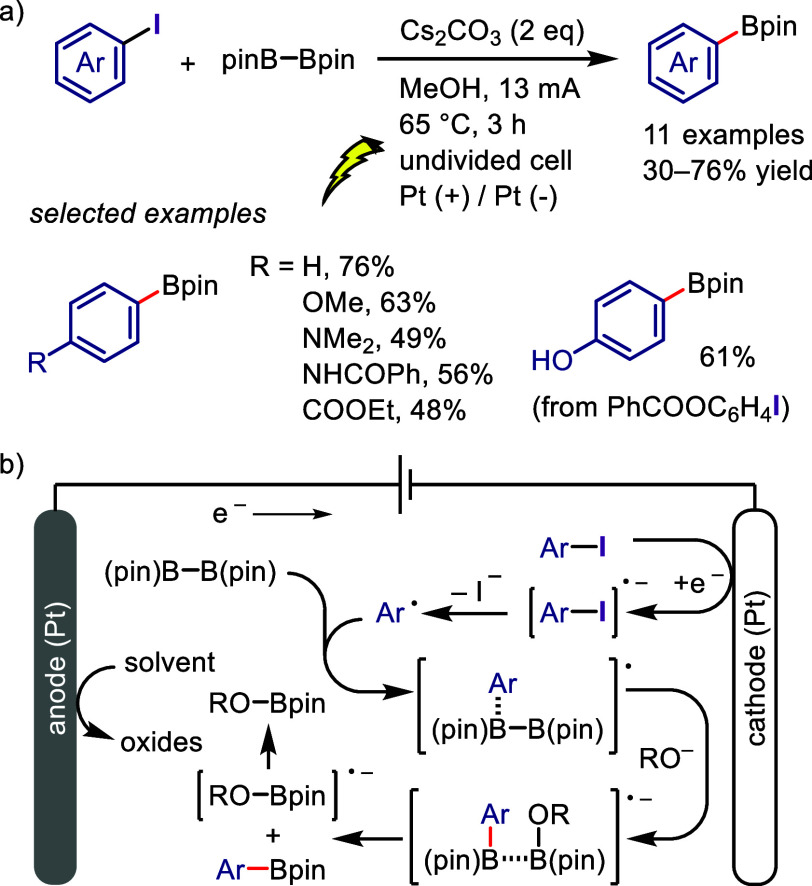
Electrochemical Borylation of Aryl Iodides

Mo et al. borylated aryl iodides through the electrochemical *in situ* generation of aryl radicals as key intermediates (Scheme 183a).^860^ A plausible radical mechanism was suggested based on electron paramagnetic resonance and cyclic voltammetry indicating the generation of aryl radicals (Scheme 183b). According to this mechanism, the aryl iodide is reduced at the cathode to form an aryl radical, which reacts with B_2_pin_2_ to form a C–B bond in the presence of a base, with subsequent B–B bond dissociation generating Ar–Bpin and a borate radical anion.

**Scheme 184:**
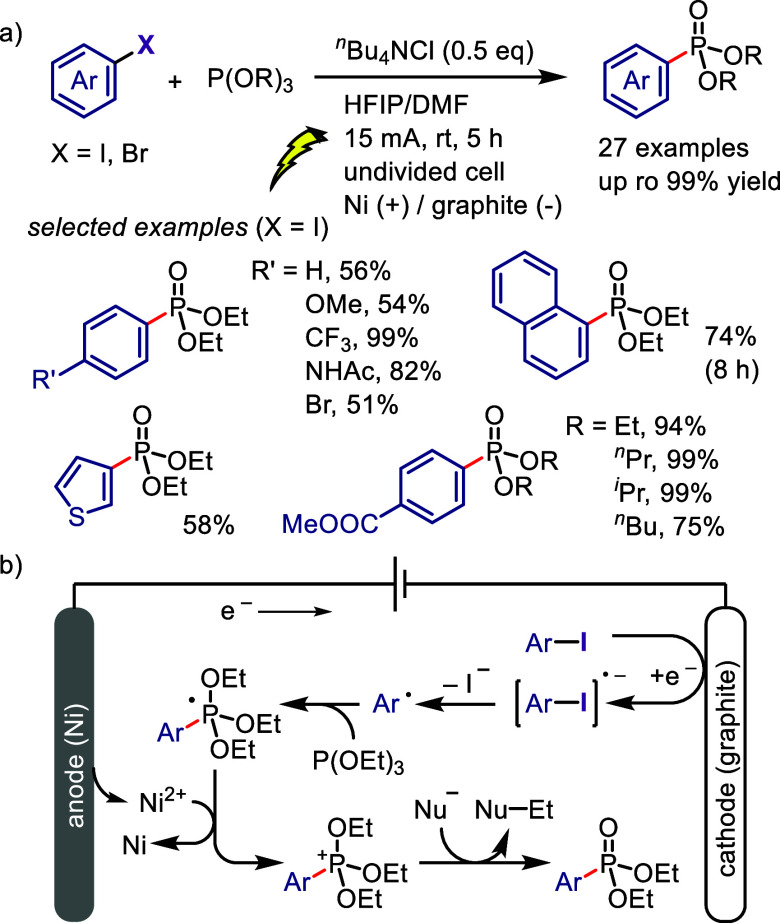
Electrochemical Phosphorylation of Aryl Halides

Wang et al. electrochemically phosphorylated aryl halides by trialkylphosphites in an undivided cell with a graphite cathode and Ni sacrificial anode (Scheme 184a).^861^ Trialkylphosphites worked well, although no reaction was observed for trimethyl- and triphenylphosphites. Mechanistic studies ruled out the possibility of Ni(II)/Ni(0) and Ni(III)/Ni(I) cycles, which are the key processes in Ni-catalyzed electrochemical reactions.^862,863^ According to a plausible mechanism, the reaction starts with the reduction of an aryl halide at the cathode to generate an aryl radical, which couples with a phosphite to form a phosphoranyl radical (Scheme 184b). The oxidation of the phosphoryl radical by Ni(II) generated through the erosion of the Ni anode followed by the reaction with nucleophiles in the reaction medium affords the desired phosphorylation product.

### 5.2 Indirect Dissociation of Ar–X Bonds

The direct electrolysis of aryl halides, particularly complex ones, can be hindered by reactivity and selectivity problems. When direct electrolysis is not efficient, an alternative electrochemical strategy that involves the use of soluble redox mediators to shuttle electrons from the cathode to the aryl halide in the solution, becomes more appropriate. Therefore, the use of redox-active organic mediators (catalysts) can make electrosynthetic transformations proceed under more benign conditions via unique mechanistic pathways and thus result in a high reactivity, selectivity, and functional group compatibility, broad substrate scope, etc.^624,625,845,849,850^

**Scheme 185:**
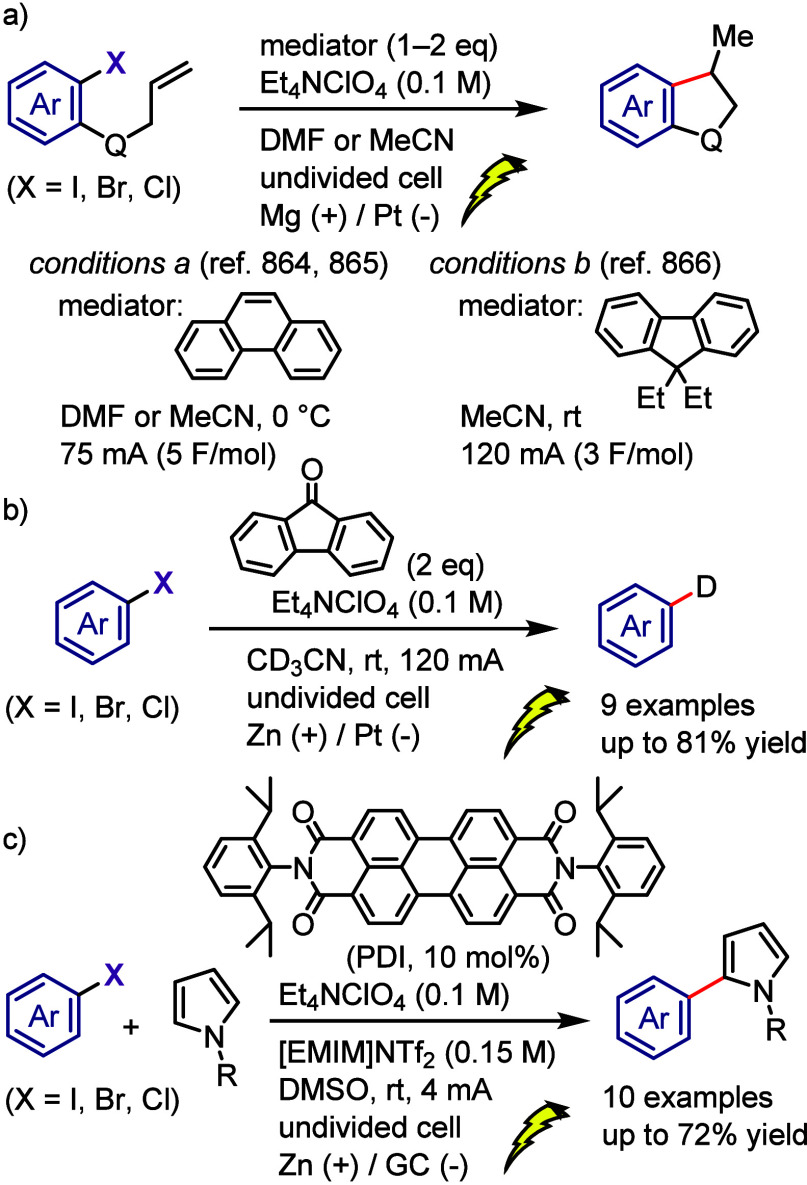
Electroreductive Functionalization of Aryl Halides in the Presence of Redox Mediators

Early examples of organic mediators include the use of phenanthrene and 9,9-disubstituted fluorenes for the reductive radical cyclization of *ortho* alkenylhaloarenes to the related five- and six-membered fused skeletons in an undivided cell equipped with a Pt cathode and Mg sacrificial anode (Scheme 185a).^864−866^ In these reactions, stoichiometric amounts of redox mediators were used. Mitsudo et al. reported the electroreductive deuteration of haloarenes in the presence of 9-fluorenone as a mediator using an undivided cell equipped with a Pt cathode and Zn sacrificial anode (Scheme 185b).^867^ In contrast, Zhu et al. demonstrated that a catalytic amount of perylene bisimide serves as indirect mediator for the direct coupling of aryl halides with pyrroles to generate heterobiaryls (Scheme 185c).^868^ The reaction worked well with 1-ethyl-3-methylimidazolium bis((trifluoromethyl)sulfonyl)imide ([EMIM]NTf_2_) as an important electrolyte in an undivided cell equipped with a glassy carbon cathode and Zn sacrificial anode.

**Scheme 186:**
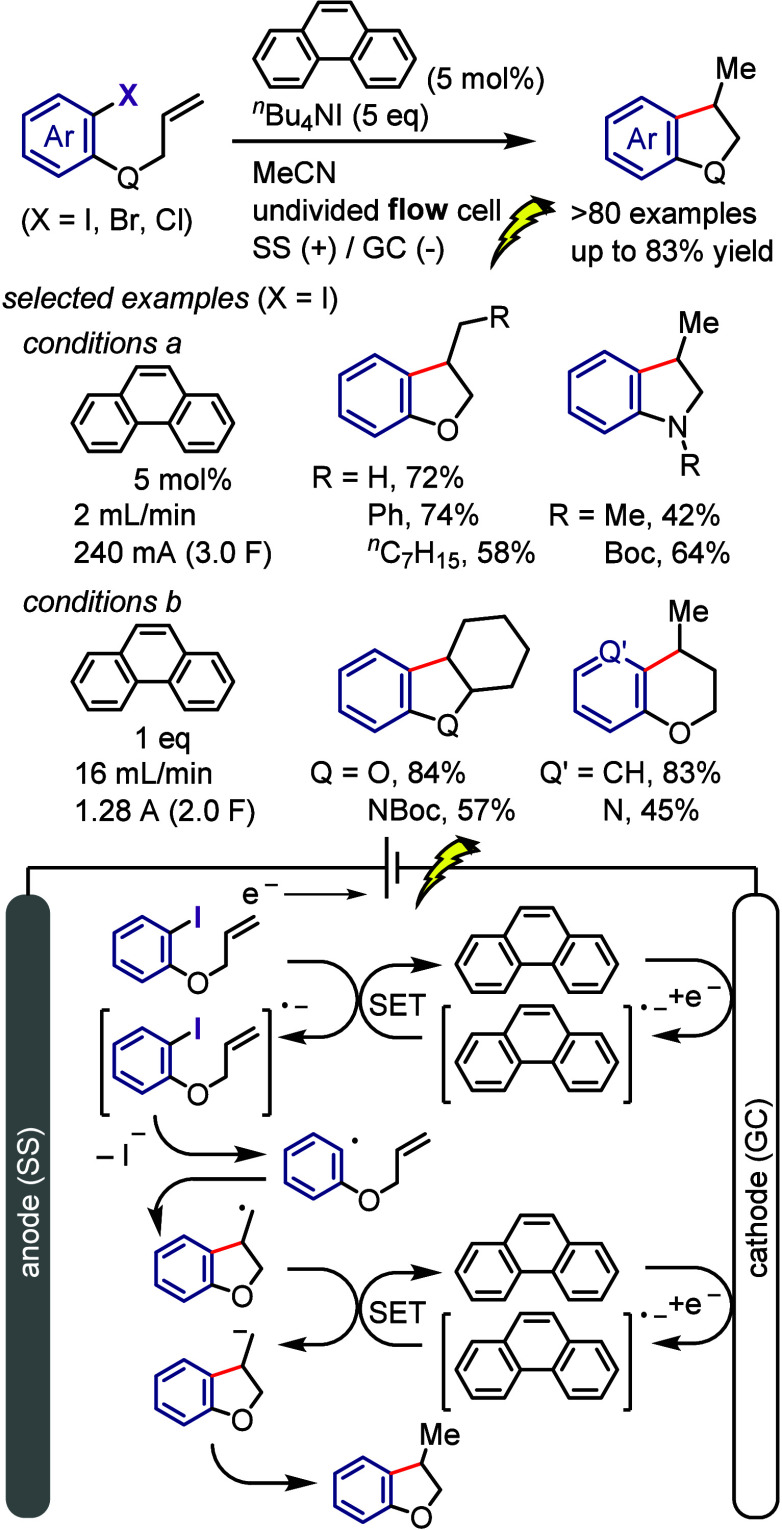
Phenanthrene-Catalyzed Electroreductive Radical Cyclization

Brown et al. realized an electroreductive radical cyclization using an undivided electrochemical flow cell equipped with a stainless steel (SS) anode and glassy carbon (GC) cathode (Scheme 186).^869^ This reaction system had the advantages of not requiring a sacrificial anode, operating with catalytic mediator amounts, and suitability for gram-scale synthesis. The proposed mechanism starts with the one-electron reduction of phenanthrene on the cathode to generate the phenanthrene radical anion, which reduces the aryl halide via SET to form an aryl radical. After cyclization with an alkenyl moiety, the reduction of the resulting radical by the phenanthrene radical anion followed by protonation affords the desired product. The presence of phenanthrene (mediator) and its radical anion results in the formation of a detached reaction layer between the cathode and Ar–X. In this layer, the mediator acts as a charge shuttle by fluxing the phenanthrene radical anion outward from the cathode to induce SET to Ar–X and thus obtain Ar–X^•–^. Thus, the direct two-electron reduction of Ar–X to Ar–H is prevented.

**Scheme 187:**
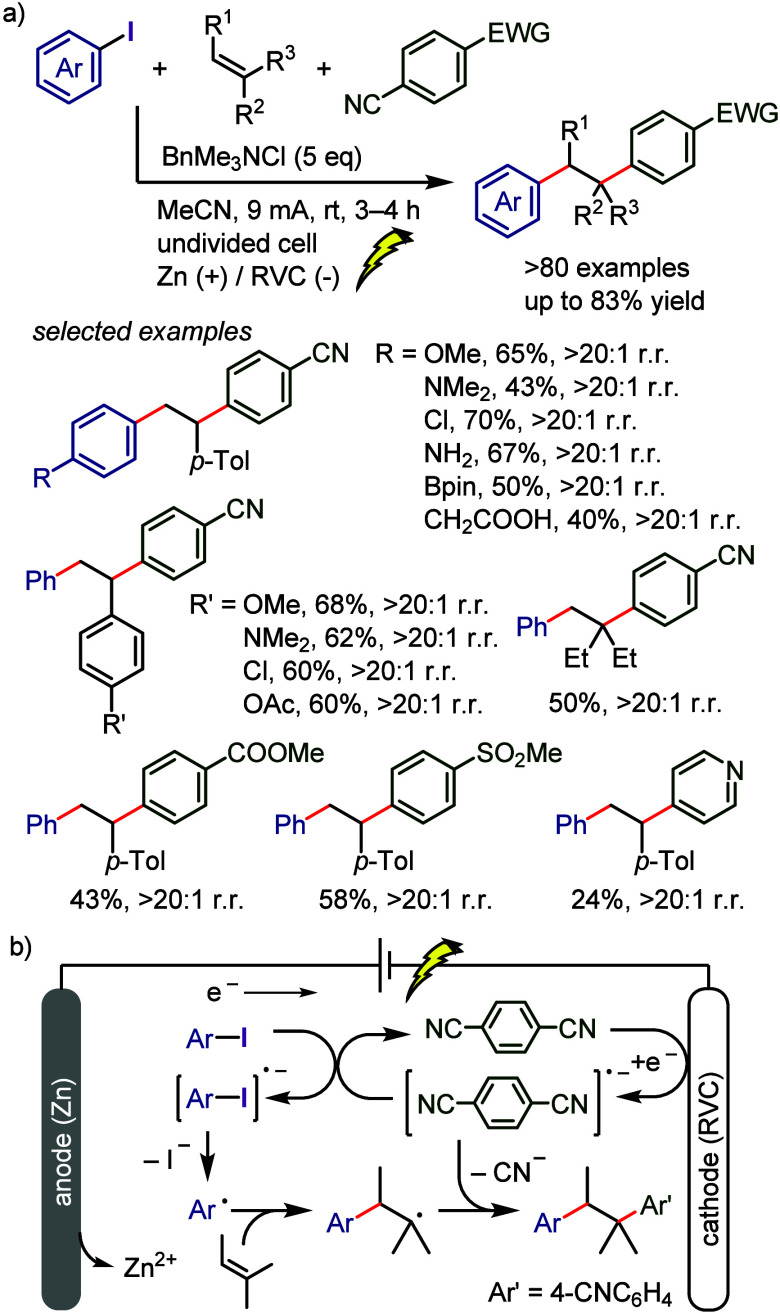
Regioselective 1,2-Diarylation of Alkenes with Aryl Halides and Aryl Nitriles

Wang et al. employed cyanoarenes as aryl radical precursors and redox mediators for the electroreductive 1,2-diarylation of alkenes using a Zn sacrificial anode and reticulated vitreous carbon (RVC) cathode (Scheme 187).^870^ This protocol was compatible with various electron-withdrawing group–substituted cyanoarenes and the gram-scale synthesis and late-stage functionalization of natural and bioactive compounds. According to the mechanism proposed based on the results of DFT calculations, dicyanoarene reduction on the cathode generates a dicyanoarene radical anion, which reduces the employed aryl halide to form an aryl radical via SET. The anti-Markovnikov addition of the aryl radical to an alkene generates the corresponding alkyl radical, which combines with the dicyanoarene radical anion to afford the desired product after cyanide expulsion.

**Scheme 188:**
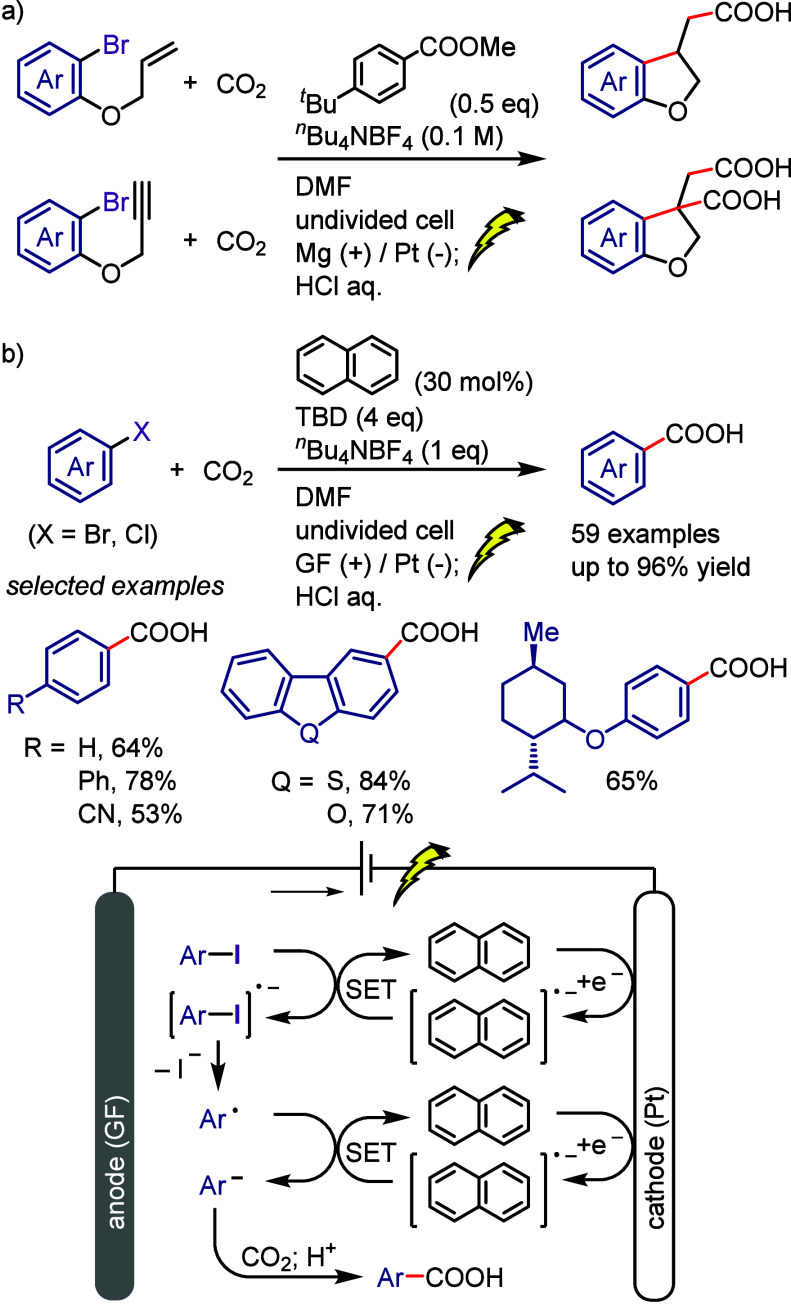
Electroreductive Radical Generation Followed by CO_2_ Trapping

The trapping of aryl or alkyl radicals by CO_2_ followed by protonation affords the corresponding carboxylic acids. Senboku et al. developed an electroreductive radical cyclization induced by substoichiometric amounts of methyl *tert*-butylbenzoate as a redox mediator, with the subsequent CO_2_ trapping affording bicyclic carboxylic acids (Scheme 188a).^871,872^ The reaction with *ortho*-alkenylbromoarenes generated monocarboxylic acids, whereas dicarboxylic acids were obtained when *ortho*-alkynylbromoarenes were used. In these cascade reactions, two or three electrons were sequentially transferred to bromoarenes for multiple bond formation. Xue et al. demonstrated the electroreductive carboxylation of aryl halides with CO_2_ generating carboxylic acid derivatives in the presence of 1,5,7-triazabicyclo[4.4.0]dec-5-ene (TBD), wherein a catalytic amount of naphthalene was used as a mediator (Scheme 188b).^873^ Electronically and sterically diverse aryl halides were tolerated, and the late-stage carboxylation of aryl halides derived from natural products and drugs could be realized. The proposed mechanism is initiated by the one-electron reduction of naphthalene on the cathode, and the thus generated naphthalene radical anion engages in SET to the aryl halide to afford an aryl radical. The reduction of the aryl radical by the naphthalene radical anion or cathode generates a radical anion, which reacts with CO_2_ to furnish the corresponding benzoic acid derivative after protonation.

## 6 Electrophotochemical Activation of Aryl Halides

**Scheme 189:**
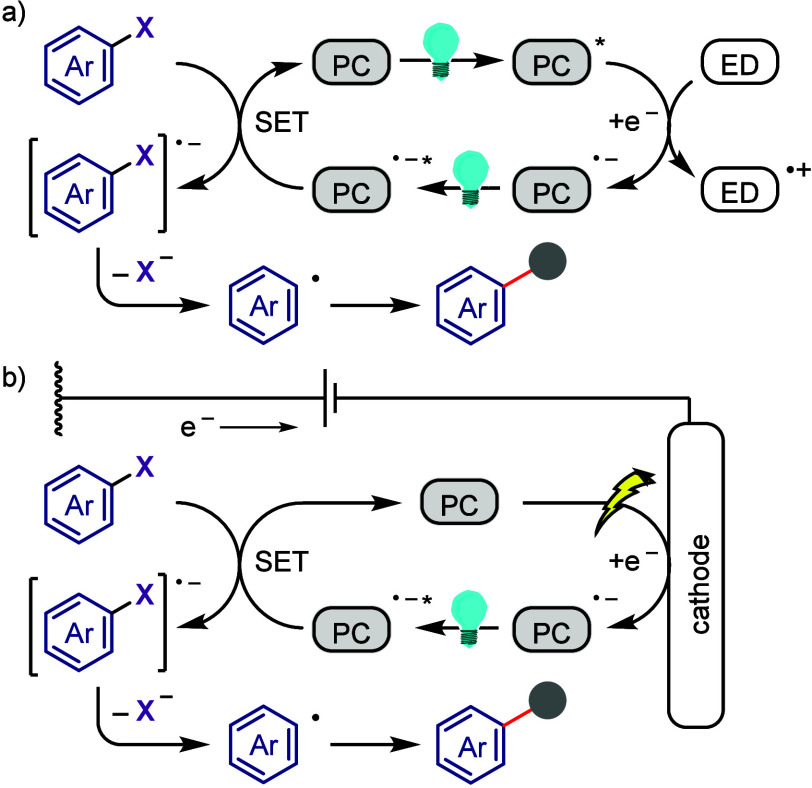
(a) Consecutive Photoinduced Electron Transfer and (b) Electrophotochemical Activation of Aryl Halides

The benefit and power of electricity and light can be merged in elegant electrophotocatalytic (or photoelectrocatalytic) strategies to solve the critical problems of chemical reactions and enable unprecedented chemical transformations.^624,625,874−885^ During consecutive photoinduced electron transfer (conPET), the photocatalyst is photoexcited and then reduced by an additional electron donor to generate a photocatalyst radical anion (PC^•–^), which is further photoexcited to afford a strongly reducing excited radical anion (PC^•–^*) (Scheme 189a).^490,746,764,886−896^ The sequential generation of PC^•–^* from the ground state of the photocatalyst can be replaced with the combination of an electrochemical process and photoirradiation; the one-electron reduction of the photocatalyst proceeds at the electrode surface to generate PC^•–^, which is photoexcited to afford PC^•–^* (Scheme 189b). This synergistic strategy, i.e., the electrophotochemical activation of aryl halides, avoids the use of additional sacrificial reagents or strong bases and, hence, side reactions. In addition, the scope of potential catalysts is considerably broadened, as a limited number of compounds can absorb light and act as photocatalysts in their neutral states, whereas their radical ions are more suitable for this process. Therefore, the electrophotochemical strategy offers a general platform for transcending the limitations of electro- and photochemical protocols. This elegant hypothesis was successfully applied to the Ar–X bond activation of aryl halides with very negative reduction potentials.

**Scheme 190:**
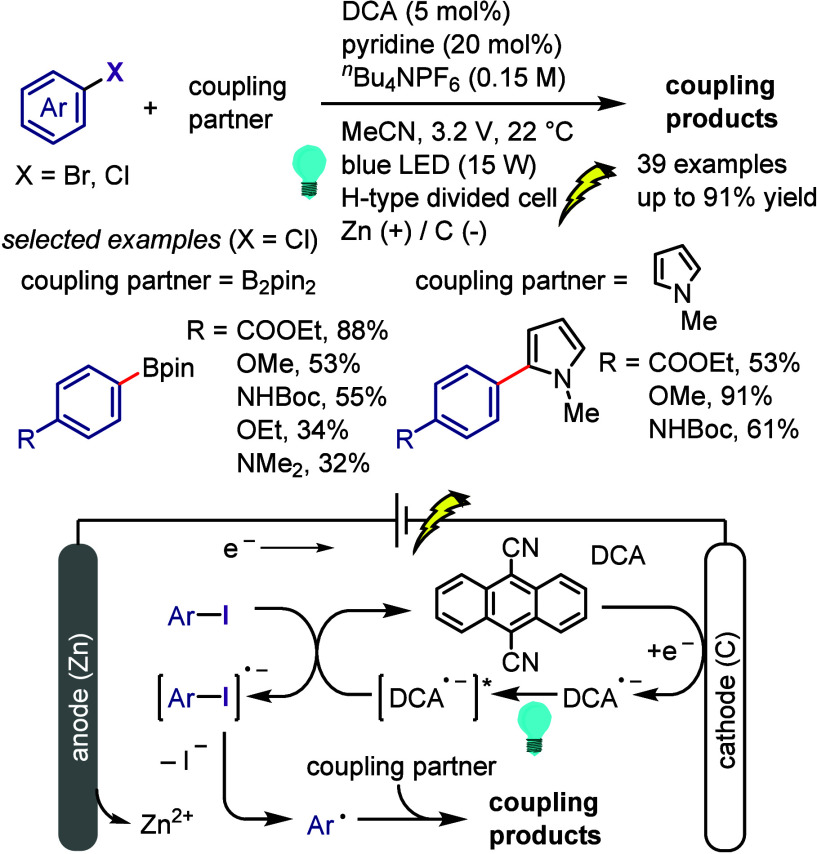
Electrophotocatalytic Functionalization of Aryl Halides Induced by Dicyanoanthracene (DCA)

Lambert et al. developed a powerful reductive electrophotocatalytic strategy mediated by dicyanoanthracene (DCA) as an organocatalyst (Scheme 190).^897^ The electrochemical reduction of DCA at the cathode generates a photoactive radical anion, DCA^•–^, which is excited by visible light to form a strongly reducing radical anion, DCA^•–^* (*E*_red_ = −3.2 V vs SCE). This protocol was applied to the reductive borylation, stannylation, and (hetero)arylation of aryl halides with very negative reduction potentials under blue-light irradiation using an H-type divided cell equipped with a carbon foam cathode and Zn-plate sacrificial anode. Challenging (hetero)aryl halides with reduction potentials of −1.9 to −2.9 V and potentially sensitive functional groups were tolerated. The reduction of the employed aryl halide by DCA^•–^* via SET leads to the regeneration of DCA and formation of an aryl radical, which is trapped by a radical acceptor (i.e., coupling partner) to afford the desired product. Mechanistic studies ruled out the formation of EDA complexes from DCA^•–^ and aryl halides, and a dual photocatalytic mechanism was also found to be unlikely, as DCA is not excited by blue-LED light.

**Scheme 191:**
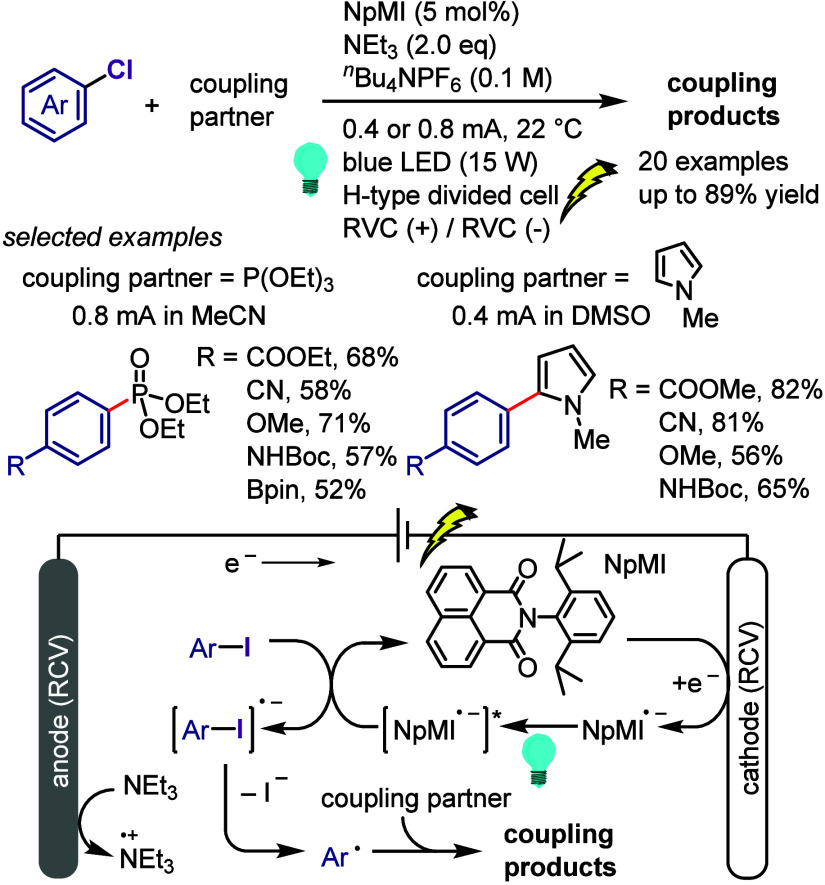
Electrophotocatalytic Reduction of Aryl Chlorides through Electron-Primed *N*-Aryl-1,8-naphthalimide (NpMI) as a Photoredox Catalyst

Wickens et al. developed an electrophotocatalytic process promoted by *N*-aryl-1,8-naphthalimide (NpMI) for the reductive hydrodehalogenation, phosphorylation, and (hetero)arylation of challenging aryl chlorides (Scheme 191).^898^ According to the proposed mechanism, the priming of NpMI with electrons before visible-light excitation leads to the generation of the strongly reducing NpMI^•–^*, which has a reduction potential beyond that of Na^0^ and comparable with that of Li^0^. This approach enabled the reduction of highly electron-rich aryl chlorides (*E*_red_ = −3.4 V vs SCE) and was compatible with various aryl chlorides bearing potentially sensitive functional groups. Moreover, the adopted strategy showed robust reactivity and selectivity compared with the traditional photochemical and electrochemical approaches to the activation of aryl chlorides and radical cross-coupling with pyrrole over hydrodehalogenation.

**Scheme 192:**
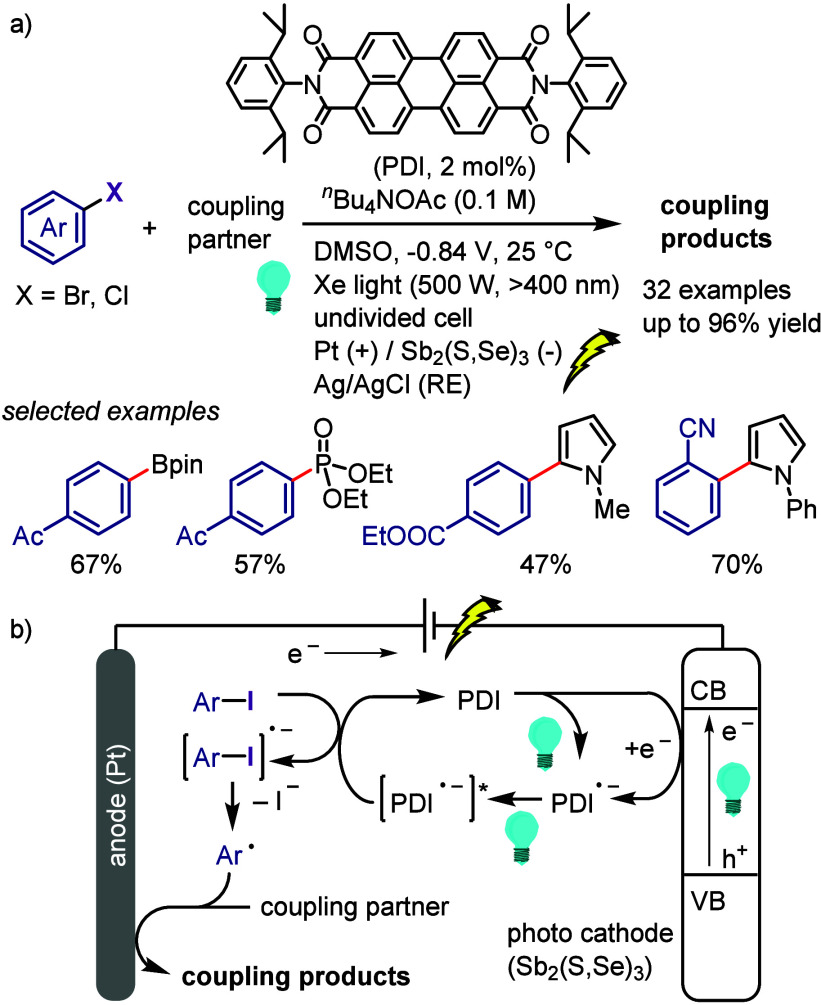
Tandem Photoelectrochemical/Photoredox Reductive Functionalization of Aryl Halides

Wu et al. designed a coupled photoelectrochemical/photoredox strategy for the efficient and selective reductive functionalization of aryl halides under visible–near-infrared (NIR) light in the presence of a perylene-based (PDI) photocatalyst in an undivided cell equipped with an Sb_2_(S,Se)_3_ photocathode, Pt anode, Ag/AgCl reference electrode, and ^*n*^Bu_4_NOAc electrolyte (Scheme 192a).^899^ PDI, the photocathode, vis–NIR light, and the acetate anion of the electrolyte were required for the transformation to take place and maximize the yield of the coupling product. A broad range of electron-deficient (hetero)aryl halides could be coupled with diverse pyrrole derivatives, P(OEt)_3_, and B_2_pin_2_ for the chemoselective construction of C–C, C–P, and C–B bonds, respectively, in preference to the competitive hydrodehalogenation. Cyclic voltammetry and spectroscopic experiments supported the mechanism shown in Scheme 192b. According to this mechanism, the vis–NIR photoexcitation of the Sb_2_(S,Se)_3_ photocathode generates electron–hole pairs, and the electrons reach the cathode surface and reduce PDI to PDI^•–^. The acetate anion of the electrolyte interacts with the π-acceptor PDI via anion−π interactions and accelerates light capture and SET to generate PDI^•–^. The second photoexcitation of PDI^•–^ affords the strongly reducing PDI^•–^* (*E*_red_ = −1.86 V vs SCE), which reduces the aryl halide to an aryl radical and is converted into PDI. The thus generated aryl radical is trapped by the coupling partner to afford the cross-coupling product.

**Scheme 193:**
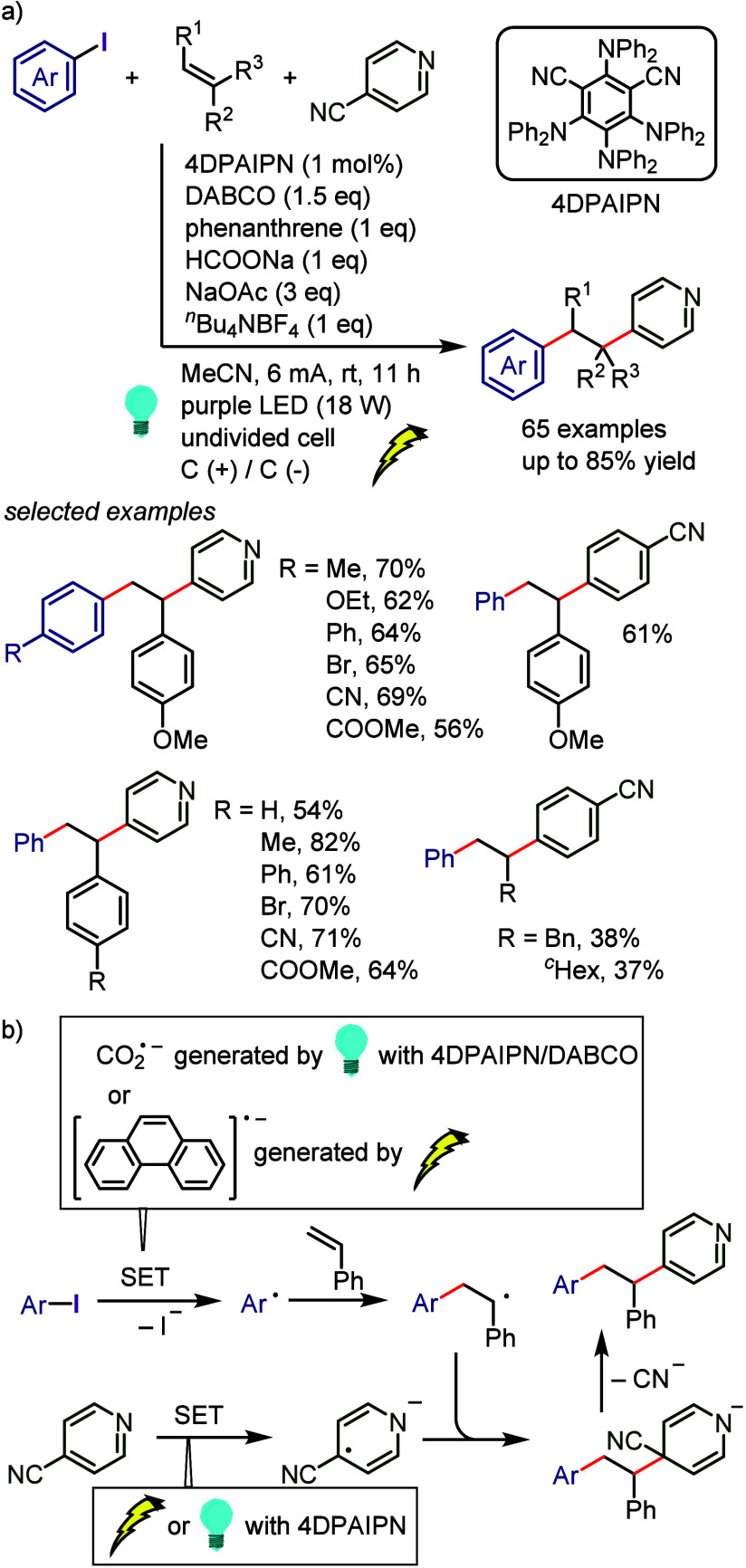
Electrophotocatalytic Three-Component Cascade Reaction Used for the 1,2-Diarylation of Alkenes

Li et al. envisaged a novel multicomponent protocol for the electrophotocatalytic 1,2-diarylation of alkenes with aryl halides and cyanoaromatics (Scheme 193a).^900^ The synergistic nature of the electrophotocatalytic process enabled the reductive generation of ArX^•–^ and ArCN^•–^ for alkene 1,2-diarylation and process termination. According to the mechanism proposed based on the results of mechanistic studies and DFT calculations (Scheme 193b), the cathodic reduction of phenanthrene generates the corresponding radical anion, which engages in SET to form an aryl radical. Analogously, the DABCO radical cation generated by the photoexcited 4-DPAIPN via SET abstracts hydrogen from HCO_2_Na to afford the reactive CO_2_^•–^, which reacts further to generate an aryl radical. The trapping of this aryl radical by an alkene followed by coupling with ArCN^•–^, which is generated through the cathodic and photocatalytic reduction of ArCN, affords three-component coupling products after decyanation.

**Scheme 194:**
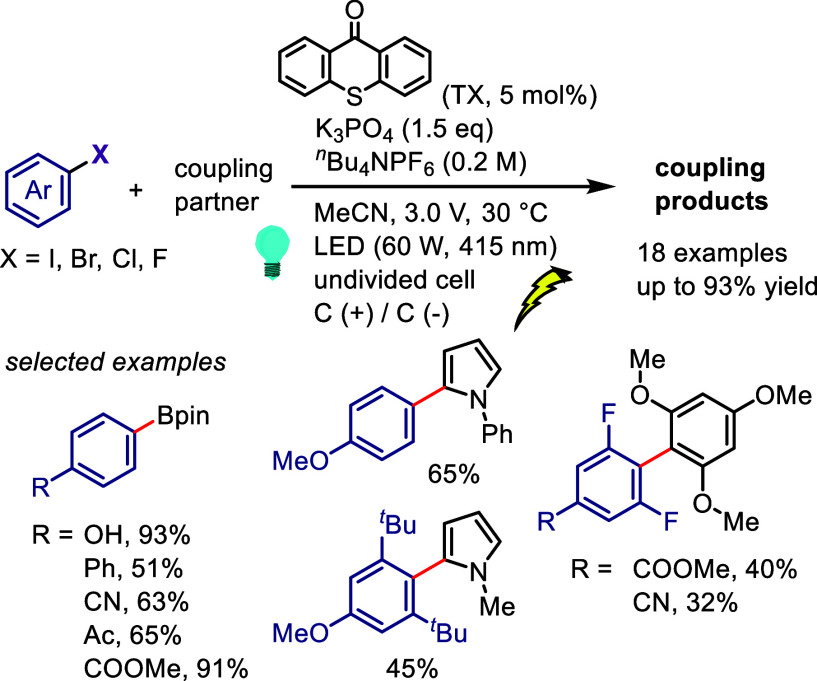
Electrophotocatalytic Reductive Functionalization of Aryl Halides

Zhang et al. used thioxanthone as a precatalyst for the electrophotocatalytic hydrogenation of imines and reductive derivatization of aryl halides (Scheme 194).^901^ The addition of TfOH to the catalytic system enabled the efficient hydrogenation of imines at low potentials and thus precluded competitive reactions. In the absence of TfOH, the catalytic system generated a strongly reducing active species with a potency comparable with that of Na^0^ and Li^0^ and therefore enabled the photocatalytic reductive functionalization of aryl halides with very negative reduction potentials. The catalytic system enabled the hydrogenation, borylation, stannylation, and arylation of exceptionally challenging aryl halides and construction of Csp^2^–H, Csp^2^–B, Csp^2^–Sn, and Csp^2^–Csp^2^ bonds. These transformations were performed in an undivided cell under reductant-free conditions (the generated radical intermediates were used as sacrificial reagents) and exhibited high faradaic efficiencies.

## 7 Assessing the Features of Iodoarene Transformations

Green chemistry is defined as the “design of chemical products and processes to reduce or eliminate the use and generation of hazardous substances.”^902−905^ To contribute to this goal, synthetic chemists have developed chemical processes that utilize low-toxicity abundant resources, employ sustainable and renewable feedstocks, minimize waste and hazardous product formation, and use smaller amounts of energy. Anastas and Warner defined the 12 principles of green chemistry in 1998 as (1) waste prevention, (2) atom economy, (3) less hazardous chemical syntheses, (4) designing safer chemicals, (5) safer solvents and auxiliaries, (6) design for energy efficiency, (7) use of renewable feedstocks, (8) reduce derivatives, (9) catalysis, (10) design for degradation, (11) real-time analysis for pollution prevention, (12) inherently safer chemistry for accident prevention.

**Figure 3:**
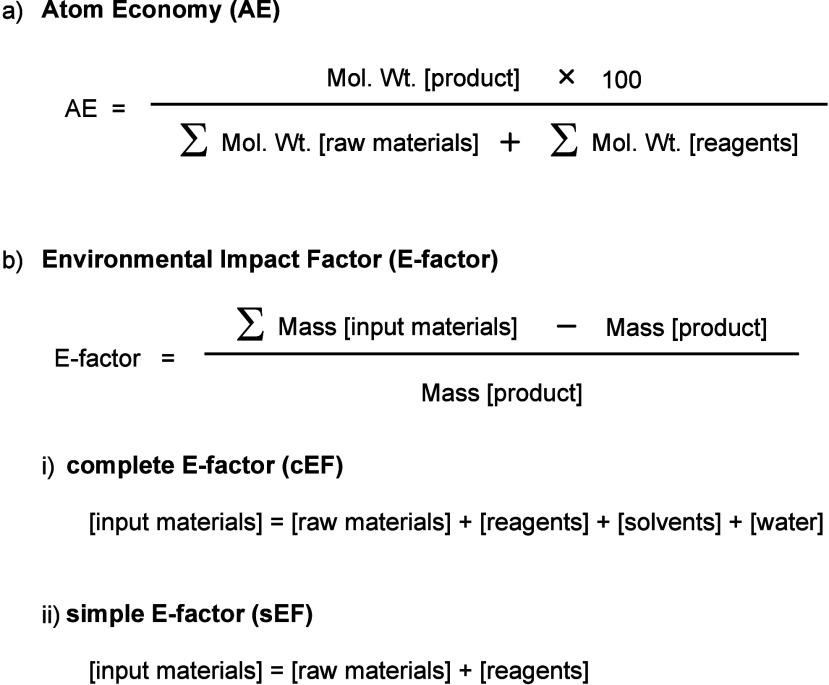
Green metrics. (a) Atom Economy (AE) and (b) Environmental Impact Factor (E-factor).

To evaluate the chemical transformations from a green chemistry perspective, Atom Economy (AE) and Environmental Impact Factor (E-factor) are the simplest and most widely adopted green metrics in both industrial and academic chemistry (Figure 3).^906,907^ AE, proposed by Trost in 1991, is calculated as the molecular weight of the desired product divided by the total molecular weight of all reactants appearing in the stoichiometric equation, expressed as a percentage (Figure 3a).^908,909^ This metric assumes the stoichiometric use of starting materials and a theoretical chemical yield, making it a practical tool for assessing alternative synthetic routes to a target molecule without requiring experimental data. In contrast, the E-factor, proposed by Sheldon in 1992, quantifies the actual waste generated in a process and is determined by dividing the total mass of waste by the mass of the final product (Figure 3b).^910,911^ Thus, an ideal E-factor is zero, with higher values indicating greater waste generation and environmental impact. The E-factor values differ depending on the industry segment, as described in the literature by Sheldon (Table 1). Waste is broadly defined as anything that is not the desired product, including all auxiliary materials such as solvents and chemicals used in workup. Originally, water was excluded from waste calculations because the use of water was considered unlikely to cause an environmental impact. However, as water disposal or reuse often necessitates pretreatment, the current trend includes water as part of the waste. A major source of waste in chemical manufacture is solvent and water losses. Therefore, the use of new related E-factors, complete E-factors (cEF) and simple E-factors (sEF), has been suggested.^906^ The cEF accounts for all process materials including solvents and water, whereas the sEF metric excludes water and solvents from the calculation (Figure 3a-i). The sEF is particularly suited for evaluating processes during the early development stage, where solvent and water use may not yet be optimized (Figure 3a-ii).

**Table 1:** E-Factors in the Chemical Industry Described by Sheldon.^906,907,910,911^

industry segment	product tonnage	E-factor (kg waste/kg product)
oil refining	10^6^–10^8^	<0.1
bulk chemicals	10^4^–10^6^	<1–5
fine chemicals	10^2^–10^2^	5–>50
pharmaceuticals	10–10^3^	25–>100

The loss of iodine in reactions using aryl iodides as the starting materials results in waste generation and reduced atom economy because of the large atomic weight of iodine (∼160). Nevertheless, aryl iodides are more reactive than other aryl halides, such as aryl bromides and chlorides, and enable various transformations even in the absence of transition metal catalysts, thus helping avoid exposure to rare and toxic metal species. Aryl iodides are oxidized to generate hypervalent iodine reagents, which can be used as nonmetal oxidants. Diaryliodonium salts are stable and easily accessible compounds that can be converted into various products via ligand coupling or aryne generation. In addition to their oxidative transformations, aryl iodides can be activated by relatively low-energy techniques, such as irradiation or electronic treatment, to promote the generation of aryl radical intermediates.

In this section, we select representative bond formations using iodoarenes as starting materials and introduce the developments concerning green sustainable chemistry. Specifically, we compare the related preparation methods and efficiency of phenol *O*-arylation, the benefits of using diaryliodonium salts as aryne precursors, and the sequential double functionalization affording benzofuran skeletons. For the direct activation of iodoarenes inducing bond-forming reactions, we selected borylation and hydroxylation initiated by irradiation or electric stimuli and compared their conditions from a green chemistry perspective. The reactions using diaryliodonium salts or iodoarenes described here contribute to safer processes, waste reduction, high yields, selective transformations, short-step synthesis, energy efficiency, and green solvent use.

### 7.1 Iodoarene Syntheses Based on Green Sustainability

**Scheme 195:**
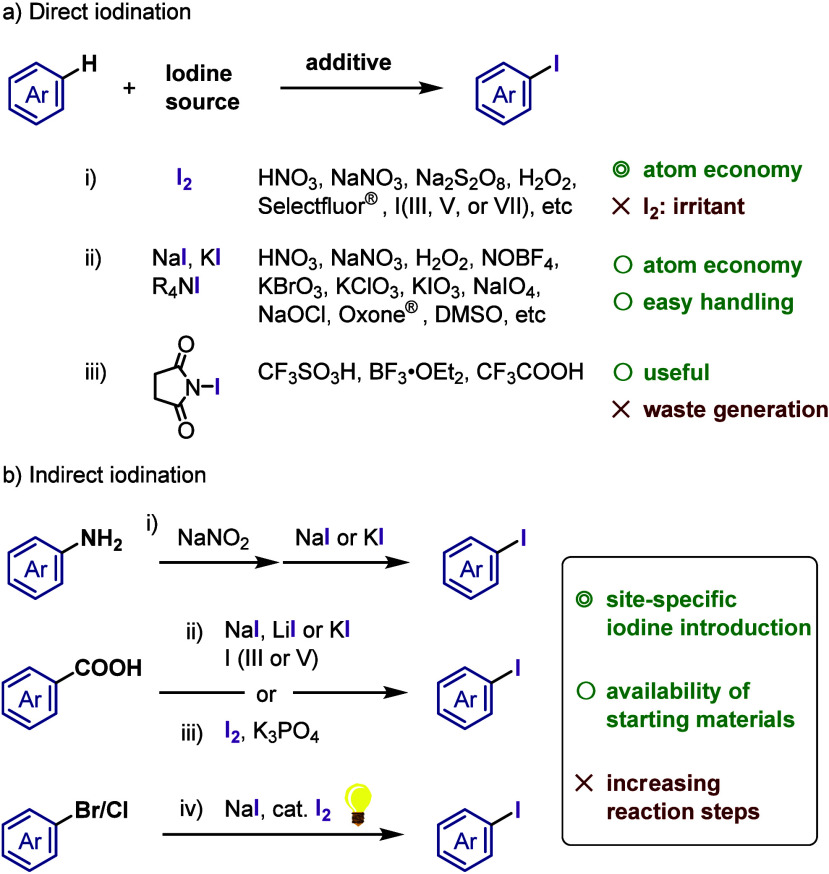
Transition Metal-Free Synthesis of Iodoarenes

Numerous methods for aryl iodination have been developed to date.^912−914^ Although various combinations of iodine sources with activating additives have been employed, this review highlights selected methods emphasizing green sustainability without the use of transition metals (Scheme 195).

For the iodination of the aryl C–H bond, electrophilic aromatic substitution of arenes has been recognized as a fundamental and widely accessible approach (Scheme 195a).^914,915^ Elemental iodine, as a cost-effective and atom-economical iodine source, is advantageous (Scheme 195a-i). However, its low reactivity renders it incapable of iodinating aromatic rings on its own, and it generates hydrogen iodide as a byproduct during the reaction. To enhance its reactivity, various acids and/or oxidizing reagents are typically employed. Acids promote the polarization of elemental iodine to increase its electrophilicity, whereas oxidants generate highly reactive iodonium cation-like species. Under oxidative conditions, iodide anions released during the reaction are further oxidized into active species, improving iodine atom economy. Heavy metal-based oxidants, including compounds of silver,^916,917^ mercury,^918,919^ lead,^920^ chromium,^921^ cerium,^922^ iron,^923,924^ and copper,^925^ have been commonly used. However, from the perspective of green and sustainable chemistry, these reagents should be avoided. Instead, nonmetallic or earth-abundant alkali metal oxidants, such as nitric acid,^926^ sodium nitrates (NaNO_3_),^927^ sodium peroxosulfates (Na_2_S_2_O_8_),^928,929^ hydrogen peroxide,^930−932^ Selectfluor,^933,934^ and iodine(III, V, or VII) compounds,^935−937^ have been employed as alternatives. Unsubstituted aromatic hydrocarbons and electron-rich aromatic compounds undergo iodination in the presence of elemental iodine and these oxidants. In contrast, electron-deficient arenes bearing nitro and carbonyl groups require severe reaction conditions using concentrated sulfuric acid due to their low nucleophilicity.^938,939^ Similarly, the iodination of heteroarenes depends on their electronic density, and the reaction conditions are adjusted accordingly.

Iodide salts, such as alkali metal or ammonium iodides, are also effective iodine sources generating iodonium cationic-species under oxidative conditions (Scheme 195a-ii). These salts are advantageous because of their ease of handling and lower volatility compared to sublimable iodine.^914,915^ In addition to common oxidants,^940−942^ nitrosonium tetrafluoroborate (NOBF_4_),^943^ potassium bromate (KBrO_3_),^944^ potassium chlorate (KClO_3_),^945^ potassium iodate (KIO_3_),^946^ sodium hypochlorite (NaOCl),^947^ Oxone,^948^ and dimethyl sulfoxide^949^ have been employed to oxidize iodide salts.

*N*-Iodo compounds, such as *N*-iodosuccinimide (NIS)^950−954^ and 1,3-diiodo-5,5-dimethylhydantoin,^955−957^ serve as efficient iodine sources, though they generate stoichiometric amounts of waste during the iodination process. The combination of NIS with suitable acids, such as triflic acid (CF_3_SO_3_H), boron trifluoride etherate (BF_3_·OEt_2_), or trifluoroacetic acid, enables the iodination of electron-deficient arenes under milder conditions compared to methods involving elemental iodine or iodide salts (Scheme 195a-iii).^951−953^ In addition to these iodinating agents, other electrophilic iodinating reagents, such as iodochloride,^958^ bis(pyridine)iodonium tetrafluoroborate,^959,960^ and *N*-iodosaccharin,^961^ have also been developed for aryl C–H bond iodinations. However, these reagents suffer from drawbacks, including high energy consumption during their preparation and the generation of substantial amounts of waste during the reaction.

Aryl iodides can be also synthesized through the substitution of other functional groups with iodine atoms, starting from prefunctionalized aromatic compounds (Scheme 195b). This approach allows for the introduction of iodine atoms based on the substituent positions, thereby enabling the synthesis of a diverse range of iodoarenes. For instance, treatment of arylamines with nitrate leads to Griess diazotization, producing aryl diazonium salts that react with alkali metal iodides to furnish the corresponding aryl iodides in a one-pot procedure (Scheme 195b-i).^962−965^ Aromatic carboxylic acids undergo decarboxylative iodination to form aryl iodides. Although heavy-metal (mercury or lead) reagents have been employed in the past,^966,967^ this transformation can be induced by nonmetallic reagents, such as Burton ester^968^ and hypervalent iodines (Scheme 195b-ii),^969−971^ albeit with significant amounts of waste. Moreover, decarboxylative iodination under simple conditions using elemental iodine and inorganic bases has been reported (Scheme 195b-iii).^972^ Halogen exchange reactions, such as the substitution of aryl bromides or chlorides, offer another efficient route to iodoarenes. Nucleophilic aromatic substitution^973^ and the transition metal-catalyzed Finkelstein reaction^974−976^ provide the iodoarene synthesis, although these methods suffer from substrate limitation or reliance on transition metals. Recent advances demonstrated a photoinduced Finkelstein reaction using sodium iodide and catalytic amount of elemental iodine, providing a more sustainable alternative (Scheme 195b-iv).^977^ In addition, aryl boronic acids,^978,979^ trifluoroborate,^980^ and triflate^981^ are effective precursors for aryl iodide synthesis, although their synthesis may consume energy and exhibit low atom economy during the iodination process.

Direct iodination of aromatic compounds using elemental iodine presents the most straightforward and cost-effective approach, offering ideal highly atom-economic transformation. However, elemental iodine is a known irritant to the eyes, skin, and respiratory tract,^982^ and its activation requires strong acids and/or oxidants. In contrast, iodide salts are easy-to-handle iodine sources that generate iodonium cation-like species in the presence of oxidants. Despite these challenges, the industrial production of various iodoarenes has been established, making them commercially available. NIS serves as a complementary iodinating agent for less reactive arenes and is available commercially, even though it generates waste and thus exhibits a low atom economy. Indirect iodination of prefunctionalized arenes also provides an efficient approach for synthesizing diverse iodoarenes, particularly when the starting materials are easily accessible, though this method requires additional reaction steps.

### 7.2 Reagent Design for Efficient Diaryliodonium Salts

#### 7.2.1 Ligand Control for Highly Reactive and Selective Arylation

**Scheme 196:**
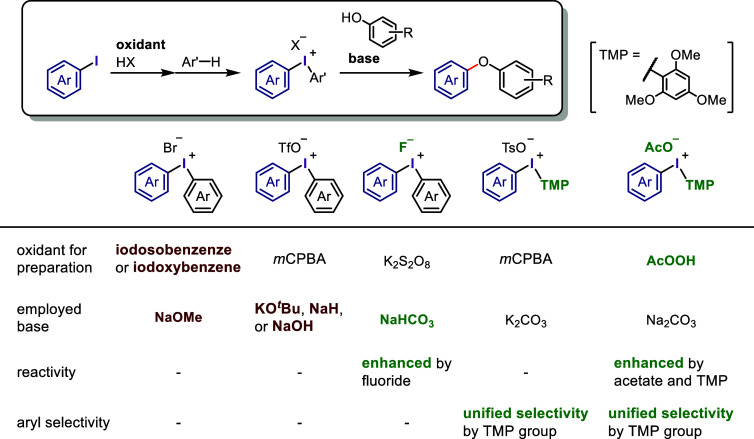
Comparison of Various Diaryliodonium Salts: Preparation and Phenol *O*-Arylation

Diaryliodonium salts are efficient arylation reagents for various nucleophiles to form aryl–carbon or aryl–heteroatom bonds in the absence of transition metal catalysts. These reactions involve ligand exchange between the nucleophile and diaryliodonium salt followed by ligand coupling between the nucleophile and one aryl group. These types of reactions were first reported in the 1950s, as exemplified by the reaction of diphenyliodonium bromide with sodium phenoxide to afford diphenyl ether.^103,104^ In this section, we compare the reported *O*-arylations of phenols with various types of diaryliodonium salts and their preparation methods (Scheme 196).

Diaryliodonium bromides, which were employed in early reports, are unstable and can be prepared using iodosobenzene or iodoxybenzene, which are explosive oxidants.^103^ Numerous works have aimed to develop easily accessible and stable diaryliodonium salts bearing various counteranions and protocols for the efficient arylation of various nucleophiles under mild conditions. Olofsson et al. reported the *O*-arylation of phenol derivatives with diaryliodonium triflates or tetrafluoroborates in the presence of bases, such as KO^*t*^Bu, NaH, or NaOH.^195−197^ These iodonium salts are relatively stable and can be prepared using a mild oxidant, *m*CPBA. Gaunt et al. used fluoride as the counteranion of diaryliodonium salts, revealing that the reaction of diaryliodonium fluoride with phenol derivatives proceeds effectively even in the presence of a weak base, NaHCO_3_ and suggesting that the fluoride anion assists the activation of hydroxy groups.^198^ When the diaryliodonium salts have two different aryl groups, both of them can participate in bond formation during arylation. To achieve the unified selectivity of aryl transfer, TMP group-bearing iodonium salts have been used.^190,219^ In this case, the other aryl group is transferred during arylation, and the TMP group is converted to TMP–I. The synthesis of TMP-iodonium salts has been well studied and can be achieved using one-pot procedures.^156,161,162^ Kita and Dohi achieved a highly reactive arylation using TMP-iodonium acetates, wherein the TMP ligand and acetate anion assisted the activation of a hydroxy group and accelerated the following ligand coupling.^220^ Diaryliodonium acetates can be prepared using 9% peracetic acid in acetic acid as a green oxidant rather than *m*CPBA; peracetic acid generates acetic acid upon oxidation, whereas *m*CPBA forms chlorobenzoic acid as waste.^170,221^

#### 7.2.2 Easily Accessible Aryne Precursors

**Scheme 197:**
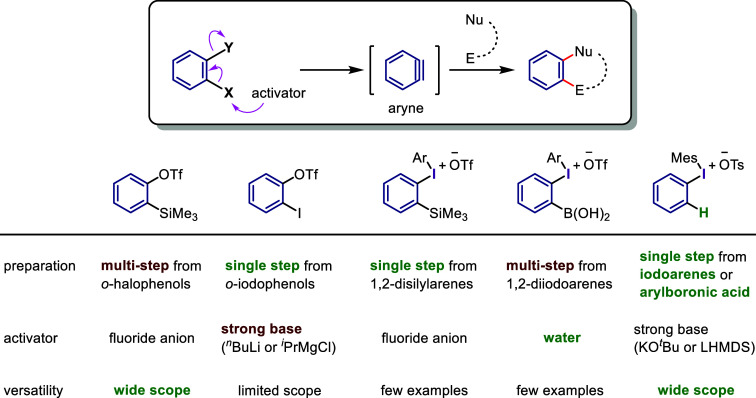
Comparison of Various Aryne Precursors: Generation and Versatility

Arynes are useful synthetic intermediates for constructing functionalized aromatic rings via carbon–carbon or carbon–heteroatom bond formation (Scheme 197).^983^ Among the available aryne generation methods, those relying on *o*-silylaryl triflates are most frequently employed, generating aryne intermediates under almost neutral conditions.^984^ Nevertheless, the preparation of these precursors generally requires multistep reactions involving the halogen–metal exchange of *o*-halophenols or *ortho*-metalation of phenol derivatives. In addition, both silyl and triflate groups serve as auxiliaries for aryne generation, which results in the production of considerable amounts of waste. Various aryne precursors have been developed to improve preparation accessibility and reduce waste. Iodoarenes and diaryliodonium salts also serve as aryne precursors. *o*-Iodoaryl sulfonates are converted into arynes via halogen–metal exchange with *n*-BuLi or *i*-PrMgCl followed by the elimination of sulfonate anion^985,986^ and can be prepared in one step from the corresponding 2-iodophenols, although the use of strong bases imposes limitations on substrate diversification. In contrast, when diaryliodonium salts are employed as aryne precursors, the iodonium moieties serve as leaving groups after *ortho* activation. Diaryliodonium salts bearing *ortho*-silyl or boryl groups generate arynes in the presence of the fluoride anion or water as activators, respectively.^418,987^ However, the syntheses of these precursors require multiple steps. Diaryliodonium salts without *ortho* functional groups also generate arynes via *ortho* deprotonation by appropriate bases, such as LDA or KO^*t*^Bu.^414^ In this case, aryne precursors can be prepared from relatively simple arenes or iodoarenes without the introduction of activating groups. Stuart et al. used Mes-iodonium salts as stable aryne precursors easily accessible by well-established methods.^237,423^ In addition, the iodomesitylene generated during aryne formation can be reused in the preparation of aryne precursors. This approach provides the step economy and waste reduction process for the synthesis of highly functionalized arenes.

### 7.2.3 Double Functionalization of Diaryliodonium Salts

**Scheme 198:**
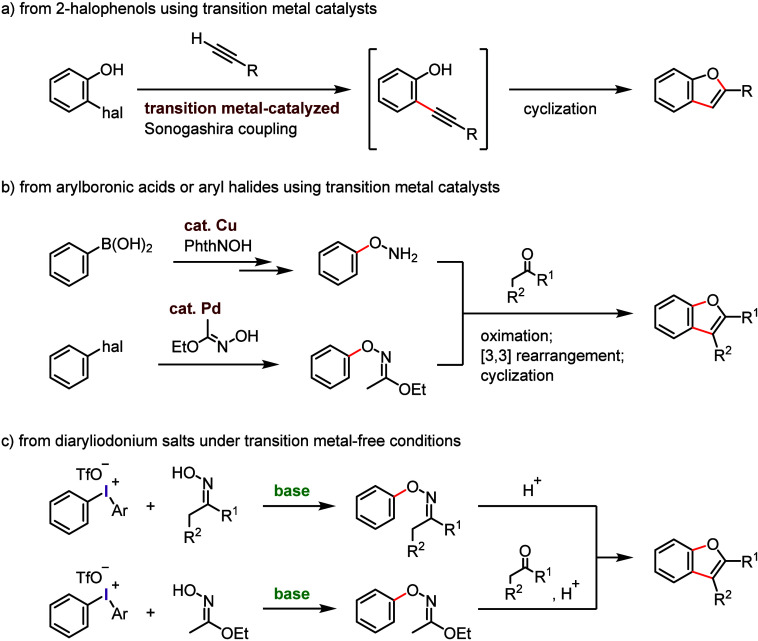
Comparison of Benzofuran Synthesis Methods

The above-mentioned aryne generation from Mes-iodonium salts represents the double functionalization of aryliodines(III) and *ortho* aryl–H bonds attached to one aryl group of diaryliodonium salts,^120^ which provides a step-economical synthesis of multifunctionalized arene derivatives. In this section, we take the synthesis of the benzofuran skeleton as an example and compare it with other approaches. Benzofurans are important motifs found in various bioactive compounds and can be synthesized via several approaches.^988−993^ When constructing benzofurans from benzene rings, one needs to incorporate carbon and oxygen atoms in mutual *ortho* positions. 2-Halophenols are useful starting materials, featuring a preinstalled oxygen atom and aryl halide moieties enabling aryl–carbon bond formation. For example, the most adopted approach involves transition metal–catalyzed coupling with terminal alkynes generating 2-alkynylphenols followed by intramolecular cyclization,^994−1001^ which has also been applied to a one-pot procedure (Scheme 198a).^1002−1009^ Relatively simple phenols not bearing halogen atoms at *ortho* positions can be also converted into benzofuran derivatives via transition metal–catalyzed aryl C–H bond functionalization (Scheme 198b).^1010−1012^ When arylboronic acids or haloarenes are employed as the starting materials, sequential transformations involving the generation of *O*-aryl oximes via transition metal–catalyzed coupling, [3,3]-sigmatropic rearrangements, and cyclization result in efficient benzofuran construction.^1013−1015^ In these approaches, the starting materials can be prepared using established and step-economical methods, and numerous benzofuran derivatives are therefore accessible. The use of diaryliodonium salts as starting materials allows the initial *O*-aryl oxime formation to be carried out under transition metal–free conditions (Scheme 198c). In addition, these sequential transformations can be realized in one pot, as independently reported by three groups at almost the same time.^211−213^

### 7.3 Development of Aryl Iodide Borylation Methods

**Scheme 199:**
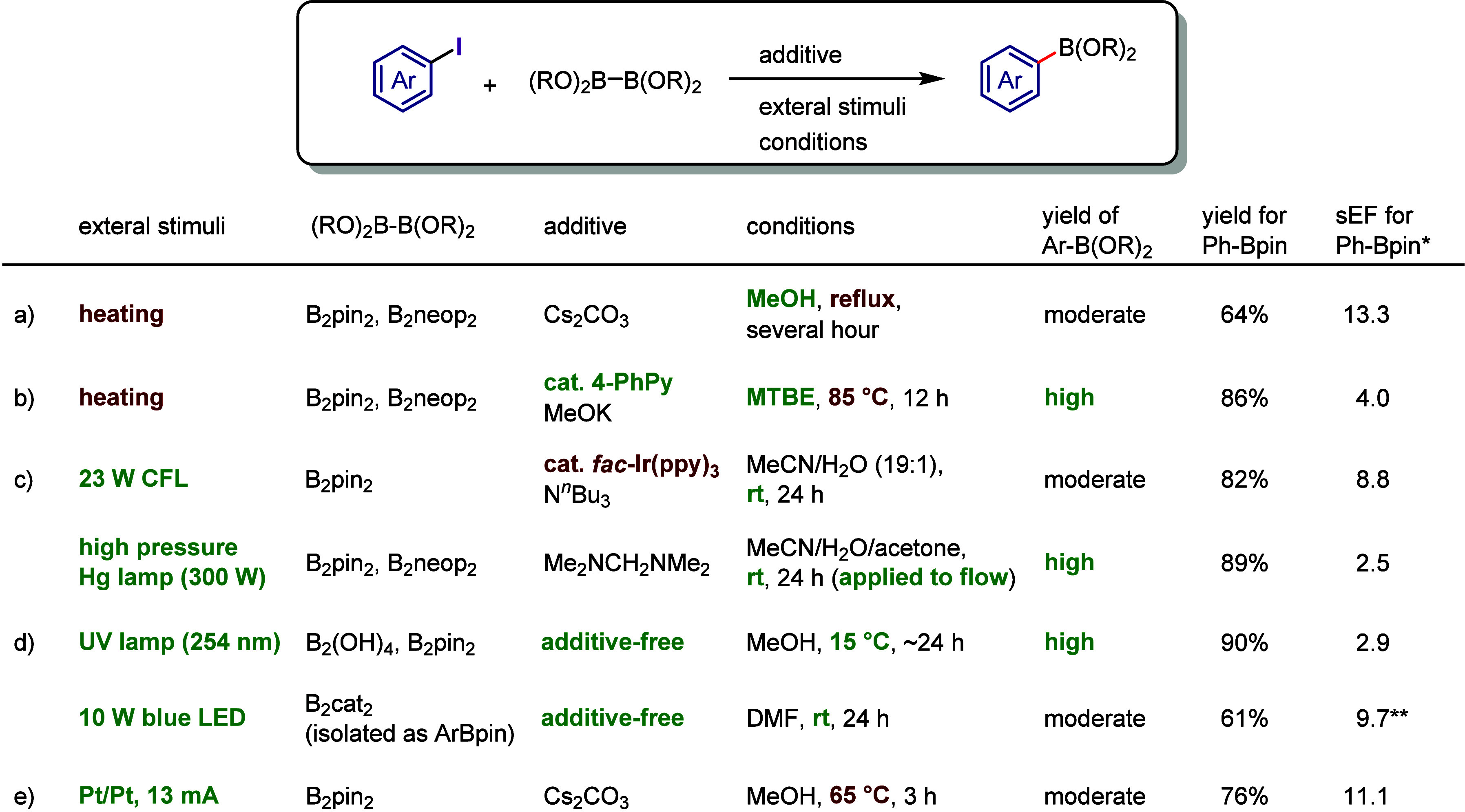
Comparison of Synthetic Approaches to Arylboronic Esters The sEF values were calculated based on the information provided in the literature. Reagents required for work-up were excluded. Estimated values for Ph-Bcat intermediate.

Arylboronates are robust and stable building blocks that can be converted into functionalized arenes via transition metal-catalyzed coupling and other reactions.^6,1016−1020^ These are typically prepared via the generation of aryl metal species followed by their reaction with trialkyl borates.^1021−1023^ Transition metal-catalyzed borylations, developed as an alternative method, require a stoichiometric amount of highly reactive organometallics or expensive and toxic transition metals.^1024−1031^ Considerable efforts have been devoted to developing cost-effective green sustainable methods for synthesizing arylboronates. Based on the breakthrough discoveries of the boron–boron bond cleavage by Hoveyda^1032^ and Fernández,^1033^ several transition metal-free borylation reactions using diboron compounds have been developed. The reaction of aryl iodides with pinacol diboron (4.0 equiv) in the presence of inorganic bases, such as cesium carbonate, in MeOH as a green solvent under reflux conditions affords arylboronic acid pinacol esters, with the sEF value was calculated to be 13.3 (Scheme 199a).^577^ The addition of a 4-phenylpyridine catalyst improves the corresponding yields by stabilizing the generated boryl intermediates, with the sEF value decreasing to 4.0 owing to the reduced amount of B_2_pin_2_ (2.0 equiv) (Scheme 199b).^580,581^

To construct an energy-effective system without heating conditions, light energy can activate aryl halides and/or diboron reagents at lower temperatures.^1034^ The combination of compact fluorescent light (CFL) with a metal-based photocatalyst^1035^ or high-pressure mercury lamp induces borylation in the presence of organic bases with low sEF values (Scheme 199c).^726,727^ In the latter case, the reaction was applied to a continuous processing system. Furthermore, additive-free photoinduced borylations were demonstrated under UV (254 nm) or blue LED irradiation, which reduced final waste (Scheme 199d).^724,728,729^ The former (using only 2.0 equiv of B_2_pin_2_ and resulting in a high yield) exhibits a lower sEF value, whereas the latter (using 4 equiv of B_2_cat_2_ and leading to a moderate yield) shows a higher sEF value. The electrochemical borylation of aryl iodides was also reported, although the addition of an inorganic base and heating conditions were still required (Scheme 199e).^860^ In this case, 4.0 equiv of B_2_pin_2_ and cesium carbonate were employed, resulting in an increased sEF value of 11.1.

### 7.4 Development of Aryl Iodide Hydroxylation Methods

**Scheme 200:**
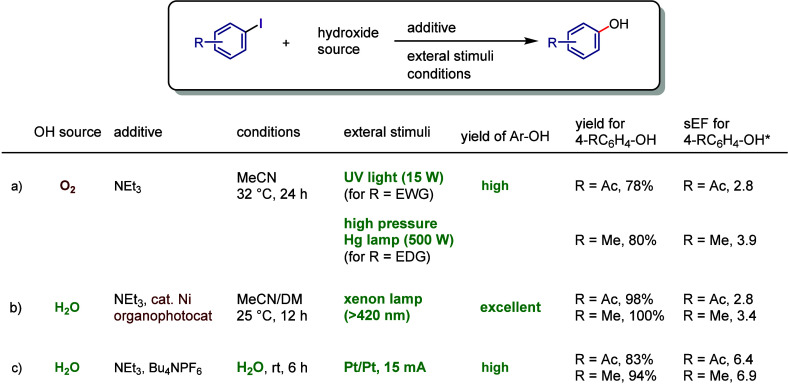
Comparison of Synthetic Approaches to Hydroxyarenes The sEF values were calculated based on the information provided in the literature. Reagents required for the work-up were excluded.

Phenol derivatives are one of the fundamental motifs of natural products, bioactive compounds, and organic functional materials;^1036−1038^ thus, various synthetic approaches to these derivatives have been developed.^1039,1040^ The transition metal-catalyzed coupling reactions of aryl halides with hydroxide anions from inorganic bases or hydroxide surrogates are robust approach,^1041−1048^ although the use of strong bases, such as the hydroxide anion, results in undesirable side reactions, whereas the reactions of hydroxide surrogates generate chemical waste. Considering the principles of green and sustainable chemistry, effective hydroxylation using alternative hydroxide sources under milder conditions is desired. Molecular oxygen was used as an alternative hydroxide source in the photoinduced hydroxylation of aryl iodides based on the photoinduced homolytic cleavage of aryl–iodine bonds used in borylation and phosphinylation reactions (Scheme 200a).^721^ In this reaction, aryl radicals were generated and trapped by molecular oxygen to generate aryl peroxyl radicals, which were converted into hydroxyarenes in the presence of triethylamine. Triethylamine is the only additive, resulting in low sEF values.

Furthermore, hydroxylation methods using water as a hydroxide source were developed, providing a safer protocol for accident prevention by avoiding the use of combustion-supporting oxygen gas. Aryl halides reacted with water to generate hydroxyarenes in a hybrid catalytic system comprising graphitic carbon nitride and a nickel complex under xenon lamp irradiation via the activation of both aryl halides and water (Scheme 200b).^1049^ Under these reaction conditions, although the number of additives increases, high reaction yields result in low sEF values. This semiheterogeneous catalytic system could potentially be applied to continuous processing. The electrochemical hydroxylation of a broad variety of aryl iodides with water was also reported (Scheme 200c).^855^ The sEF value is slightly higher due to the addition of ammonium hexafluorophosphate. Nevertheless, this reaction proceeded in water, the most recommended green solvent.

## 8 Summary and Outlook

Transition metal–catalyzed arylations suffer from several drawbacks, including their environmental and economic impacts. Therefore, considerable attention has been drawn to the development of green and sustainable strategies for constructing diverse aromatic frameworks under mild conditions. This review discusses the rapidly growing fields of (hetero)aryl halide activation for the direct (hetero)arylation of trapping reagents and the construction of chemical bonds under benign conditions, focusing on the popular strategies applicable to the activation of aryl halides. The hypervalent activation of iodoarenes via the formation of diaryliodonium salts provides a unique strategy for dissociating Ar–I bonds and generating highly reactive intermediates. The reactivity of diaryliodonium salts is attributed to the presence of σ-hole(s) on the I(III) surface, which allows these salts to act as Lewis acids in addition to being reactive arylating reagents. The feasibility of Ar–I(III) bond cleavage and the generation of formal aryl cation, aryl radical, and aryne intermediates enables the construction of diverse C–C and C–heteroatom bonds under transition metal-free benign (thermal and photochemical) conditions. Symmetrical diaryliodonium salts were first used as aryl transfer reagents to increase chemoselectivity. Interestingly, diaryliodonium salts bearing sterically congested and/or electron-rich aryl groups as dummy (auxiliary) ligands were found to be suitable for achieving the highly chemoselective transfer of the aryl electrophile. In view of their exceptionally high reactivity in S_N_Ar reactions of diaryliodonium salts can become common arylating reagents for the synthesis of radiolabeled molecules for biomolecular imaging (PET).

Despite the usefulness of this transition metal-free direct arylation protocol, it suffers from the production of aryl iodide waste (and therefore exhibits a low atom economy of 10–20%) and potential purification problems. Therefore, we discussed the recent successful strategies for tackling this issue and maximizing the arylation atom efficiency. One strategy makes use of the two aryl groups of the iodonium salt and their incorporation into the final product through one-pot cascade reactions. Alternatively, one can recycle the aryl iodide byproduct. The recycling of iodoarene waste can not only solve the main problem of this approach and provide an attractive transition metal-free strategy for the C–H functionalization of unactivated (hetero)arenes but also enable the use of aryl iodides in catalytic amounts. Thus, we hope that this review will be of interest to researchers aiming to develop new methods of solving the problems associated with this piece of chemistry.

Base-induced Ar–I bond dissociation through SET from a ground-state electron-donating intermediate under organic promoter and organic promoter–free conditions was overviewed. The addition of an organic promoter significantly improves the rate of the transformation through the cascade reaction of a base (normally KO^*t*^Bu) and this promoter to *in situ* generate strong electron donors capable of reducing Ar–X through SET. Under organic promoter–free conditions, the electrons could originate from the combinations of base/high reaction temperature, reaction of a base with the solvent, and generation of strongly electron- donating intermediates or base-activated halogen bond–assisted Ar–X bond cleavage. Notably, the generation of strong electron donors even in very low yields is sufficient to initiate the chain mechanism. These protocols employ unique organic electron donors with tunable reduction ability for the conversion of aryl halides into the highly reactive aryl radical and aryl anion intermediates, which participate in the formation of diverse bonds and heterocyclic skeletons under benign transition metal-free conditions. The advantages of this sustainable chemistry will promote further studies to (a) fully understand the reaction mechanism and provide a full structural picture of the *in situ* generated organic electron-donating intermediates to guide the design of more powerful promoters; (b) overcome the drawbacks of regioselectivity and the use of substrates in large molar excess (as solvents); (c) investigate other ways of accessing the SET process; (d) establish general methods with broad substrates scope for the highly chemo- and regioselective syntheses of highly functionalized products; (e) develop novel organic electron donors from easily accessible starting materials capable of reducing not only the electronically and sterically diverse aryl iodides but also the more challenging aryl bromides, chlorides, and fluorides.

In addition to the ground-sat thermal ways of activations, the situation is more attractive for the excited-state reactions, with photoinduced aryl–halogen bond cleavage playing a vital role as a complementary/alternative sustainable strategy of transition metal–catalyzed processes for the derivatization of haloarenes under mild conditions. In the context of this review, we highlighted the possible transition metal-free sustainable strategies for the photoactivation of haloarenes. The most convenient approach to light-induced aryl–iodine bond cleavage under metal- and photocatalyst-free conditions is the direct excitation of aryl iodides with UV light or SET. For SET to occur under photocatalyst-free conditions, the substrate should act as a photoreductant and transfer a single electron to the desired aryl halide under irradiation; alternatively, the aryl iodide can interact with an electron donor and form an EDA complex capable of absorbing visible light and afford an excited state where SET takes place. Another transition metal-free photochemical strategy for activating aryl halides relies on organic photocatalysts. In such reactions, the excited photocatalysts can reduce aryl halides via SET. Moreover, XAT activation is a useful methodology employed for Ar–I bond cleavage with no need for strong reductants. These activation reactions result in Ar–X bond cleavage and generate aryl radicals for C–C and C–heteroatom bond formation with high functional group tolerance under environmentally benign conditions.

Despite the progress in and advantages of the transition metal-free photoactivation of aryl halides, further studies in the following directions should develop valuable alternatives to transition metal–catalyzed processes: (a) Expand the present strategies and discover more tactics for the metal- and photocatalyst-free photoactivation of aryl halides; (b) design new transition metal-free organic photocatalysts with suitable redox potentials for improving the catalytic performance of photochemical transformations, overcome the challenges of aryl halide substrates, and enhance the scope and diversity of the photochemical process; (c) develop novel photochemical transformations applicable to the synthesis of challenging products, including natural products and pharmaceuticals; (d) understand the mechanistic details of aryl halide photoactivation to design efficient photochemical cross-couplings; (e) expand the light absorption range to enable visible light–driven reactions; (f) improve the quantum efficiency of the time-consuming photochemical processes; (g) avoid the use of sacrificial electron donors, (h) reduce the molar excess, improve the chemo- and regioselectivity, and broaden the scope of arenes in the C–H arylation of unactivated arenes with aryl halide.

Electrochemical synthesis has emerged as an efficient and green method of achieving challenging transformations using electric current as a driving force. In this case, electrons are used as a potent, controllable, traceless, and green redox reagent instead of the environmentally hazardous chemical substances, which allows one to lower the associated risks and costs, reduce waste generation, hinder side reactions, and elevates the atom and step economy of the process. Electrochemical reductive functionalization at the cathode is relatively rare compared with oxidative reactions at the anode. Considering the aim of this review, we highlighted two possible ways of inducing the electrochemical dissociation of aryl halides at the cathode and generating aryl radicals as very reactive intermediates suitable for various cross-couplings. Direct electrolysis involves direct SET from the surface of the cathode to the aryl halide and suffers from reactivity and selectivity problems. Therefore, a more convenient indirect electrolysis strategy was developed for the electroreductive functionalization of aryl halides under more benign conditions with greater reactivity, selectivity, substrate scope, functional group tolerance, etc. In this case, an organic mediator acts as a redox-active catalyst to shuttle the electrons from the cathode to aryl halide.

As reductive functionalization at the cathode is underdeveloped, we expect the synthetic community to make efforts at broadening the reaction scope, overcoming the challenges associated with the reductive dissociation of aryl–halogen bonds, and developing more selective, efficient, sustainable, and practical electrochemical approaches. These targets could be fulfilled by, i.e., (a) designing benign electroreductive strategies through, e.g., the fabrication of more efficient electrode materials and/or use of new organic mediators with suitable redox potentials; (b) designing novel organocatalysts with unique catalytic pathways and discovering new catalytic concepts; (c) avoiding the use of sacrificial reagents and electrodes through the development of paired electrolysis systems for the activation of aryl halides; (d) developing mild electrochemical processes for the late-stage functionalization of complex substrates.

Similar to conPET methodologies for the reductive functionalization of haloarenes but with no need for sacrificial electron-donating additives, electrophotochemical activation strategies were developed for haloarenes with extremely negative reduction potentials. This new strategy enables the further expansion of the electro- and photochemical approaches by combining the two chemistries in one step while maximizing their advantages and minimizing their limitations. As the electrophotochemical strategy is still in its infancy, we expect more discoveries in the near future, particularly in relation to the development of new commercially available, cheap, and potent organic mediators.

In general, we highlighted the pioneering works and discussed the recent progress in the activation of haloarenes via hypervalency, bases, and organocatalysts under the action of heat, radiation, and electricity. Further studies should deal with weaknesses of these processes to make them competitive sustainable alternatives to traditional and metal-catalyzed approaches.
